# Self-adaptive layered oxide cathodes driven by charge reconfiguration for sodium-ion batteries

**DOI:** 10.1038/s41467-026-78040-8

**Published:** 2026-09-28

**Authors:** Jia-Yang Li, Ying Guo, Xin Wang, Wei Kong Pang, Hai-Yan Hu, Yan-Fang Zhu, Diancheng Chen, Xin-Yu Liu, Qin-Fen Gu, Bernt Johannessen, Xiaobo Zheng, Yang Sun, Shaobo Cheng, Yifei Yuan, Chao Wu, Jia-Zhao Wang, Shi-Xue Dou, Yao Xiao

**Affiliations:** 1 College of Chemistry and Materials Engineering, Wenzhou University, Wenzhou, P. R. China; 2 Institute for Superconducting and Electronic Materials, Australian Institute for Innovative Materials, Innovation Campus, University of Wollongong, North Wollongong, Australia; 3 Henan Key Laboratory of Diamond Materials and Devices, Key Laboratory of Integrated Circuit, Ministry of Education, School of Physics, Zhengzhou University, Zhengzhou, China; 4 School of Materials, Sun Yat-sen University, Shenzhen, China; 5 Australian Synchrotron, ANSTO, Clayton, Australia; 6 Centre for Clean Energy Technology, School of Mathematical and Physical Sciences, Faculty of Science, University of Technology Sydney, Sydney, Australia; 7 Institute of Energy Materials Science (IEMS), University of Shanghai for Science and Technology, Shanghai, China; 8 State Key Laboratory of New Textile Materials and Advanced Processing, Wuhan Textile University, Wuhan, P. R. China

**Keywords:** Batteries, Batteries, Energy, Batteries

## Abstract

Layered oxide cathodes have attracted worldwide attention due to environmentally friendliness, structural diversity, and economic benefits. However, poor long-term cycling and unsatisfactory rate performance hinder the practical application. Hence, an oxide cathode with self-adaptive volume has been designed, achieving stable capacity retention of 82% after 1000 cycles at 2 C. The enhanced long-term performance is attributed to its dynamically adjustable volume reducing the acceleration of stress, enabled by a tunable interlayer distance that expands at the beginning of the charging process and then compresses at the highest voltage. Uniform electric field distribution is depicted by 4-dimensional scanning transmission electron microscopy showing even Coulombic interactions between the oxide layers leading to high mechanical integrity. Such self-adaptable behavior originates from the reduced repulsive force between interlayered oxygen due to the charge reconfiguration of intralayer oxygen and transition metal ions. Additionally, the charge compensation behavior is detected by in situ X-ray absorption spectra, which would be beneficial to high-rate performance. The self-adaptive volume change driven by charge reconfiguration provides a pathway toward durable materials for sustainable sodium-ion energy storage.

## Abstract (web summary)

Layered oxide positive electrode materials for Na-ion batteries suffer from poor cycling stability and rate capability. Here, authors develop a multi-element doped high-entropy layered oxide cathode that exhibits self-adaptive volume changes during charging, enabling stable capacity retention.

## Introduction

Layered oxide cathodes (Na_x_TMO_2_, where TM = transition metal) have attracted worldwide attention due to environmentally friendliness, structural diversity, compositional tunability and economic benefits caused by non-toxic and abundant transition metals, which are potential commercial cathode candidates for sodium ion batteries (SIBs)^1–4^. However, stress accumulation resulting from complex phase transformations and interlayer slipping deteriorate structural integrity by promoting crack propagation, which would ultimately lead to cell failure^5–9^. To address the aforementioned issue, considerable efforts have been devoted to suppressing phase transitions and enhancing the mechanical robustness of layered oxide cathode materials.

Among these, the design of specific crystal structures is a widely adopted strategy. For instance, Sun et al. reported a tailored P2-type structure achieved by introducing cation vacancies, which effectively inhibited phase transitions and consequently improved structural stability^10^. Moreover, constructing composite phases such as P2/O3 intergrowth structure could combine the advantages of both phases, offering enhanced electrochemical performance as well^11,12^. However, these solutions could mitigate the stress variations originated from phase transitions and enhance structural stability to a certain degree, the repeated insertion and extraction of sodium ions inevitably induce lattice expansion and contraction, leading to the continuous generation of internal mechanical stress. Thus, a fundamental solution to stress-related degradation might depend on the development of self-adaptive materials that can dynamically respond to mechanical stress by reconfiguring their structure to eliminate early stage stress accumulation. The advantageous properties of high-entropy materials such as enhanced phase stability and chemical versatility have been applied to various research fields^13–15^. As a result, an increasing number of high-entropy materials have been explored as cathodes, offering the potential to achieve self-adaptive volume change cathodes through the synergistic effects among different constituent elements^16–19^. Despite the expected improvements in electrochemical performance achieved through the introduction of the high-entropy concept, these enhancements are generally attributed to the high-entropy cocktail or synergistic effect. The individual roles and mechanistic contributions of each specific component remain insufficiently elucidated and have rarely been investigated systematically^20,21^. If self-adaptive structure can be obtained based on the high-entropy concept, and the functional contributions of each element can be elucidated, it would provide valuable guidance for the design of highly stable oxide cathodes.

Therefore, we design a layered oxide Na_2/3_Ni_1/6_Li_1/18_Cu_1/18_Mg_1/18_Mn_1/3_Ti_1/3_O_2_ (NLCMMTO) based on high-entropy concept of multiple element substitution, aiming to achieve enhanced long-life sustainable cathodes. Such NLCMMTO exhibits self-adaptive structural behavior, which could effectively accommodate volume fluctuations that interlayer spacing first expands due to O–O repulsion and then contracts at high voltage due to charge reconfiguration in the TM–O framework. Additionally, the roles of individual elements have been studied via advanced techniques. The introduction of Cu would enhance the redox activity of oxygen forming bridged TM–O–TM framework with delocalized oxygen hole states, which reduces the repulsive force between interlayer oxygen leading to compression of layer distance during charging process and acceleration in electron and ion movement. The Li plays an important role in suppressing the overoxidation of oxygen with stabilizing bridged TM–O–TM framework and then strengthening the structural framework and preserving mechanical integrity. The charge reconfiguration driven self-adaptive volume change provides valuable insights into the design of long-life and structurally stable cathodes for sustainable SIBs.

## Results and discussion

### Physical characterization

**Fig. 1:**
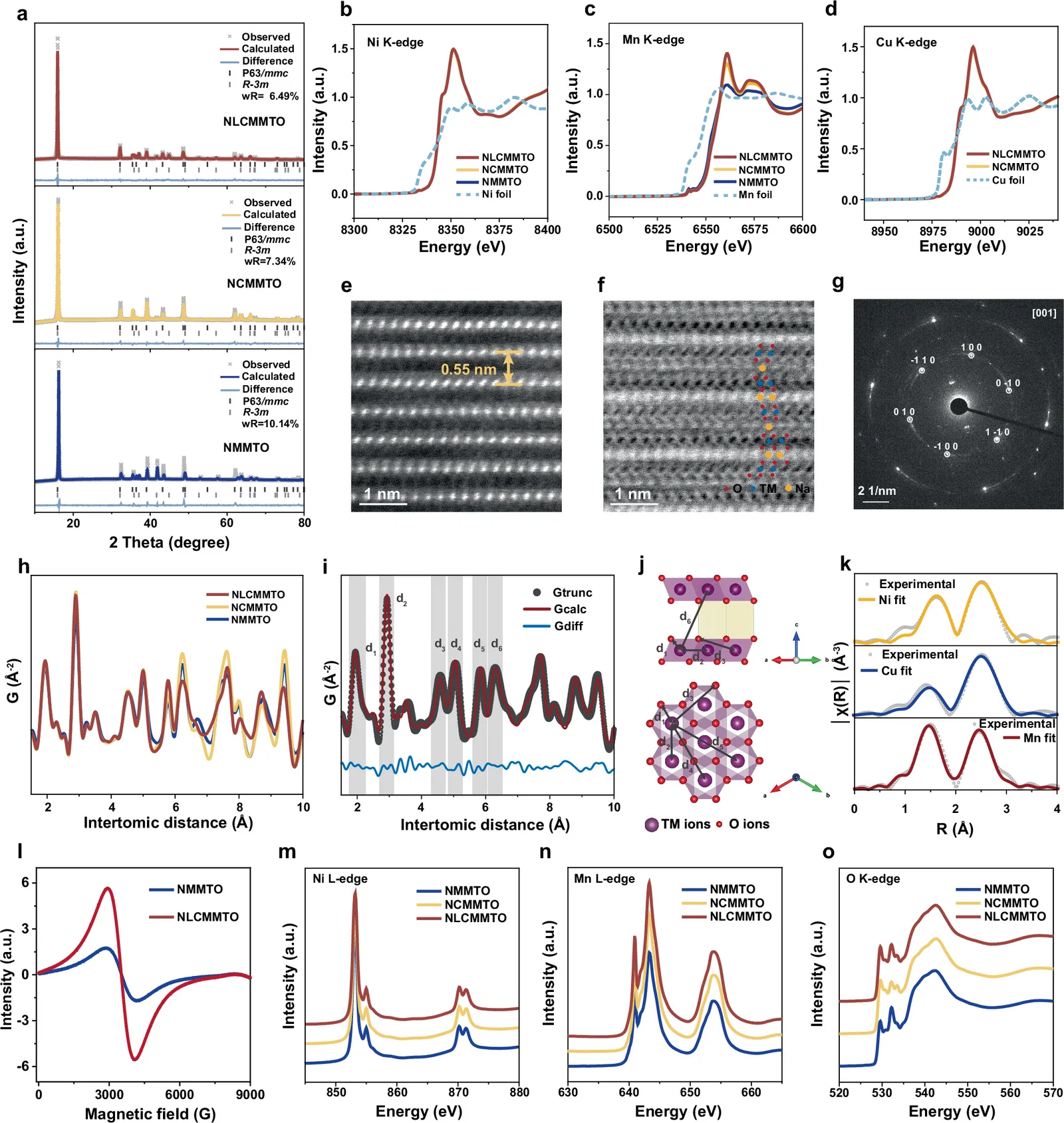
Physical characterization of Na_2/3_Ni_1/6_Mg_1/6_Mn_1/3_Ti_1/3_O_2_(NMMTO), Na_2/3_Ni_1/6_Cu_1/9_Mg_1/18_Mn_1/3_Ti_1/3_O_2_(NCMMTO), Na_2/3_Ni_1/6_Li_1/18_Cu_1/18_Mg_1/18_Mn_1/3_Ti_1/3_O_2_(NLCMMTO) samples. **a** XRD patterns of NMMTO, NCMMTO and NLCMMTO. **b** Ni K-edge; **c** Mn K-edge; **d** Cu K-edge of NMMTO, NCMMTO and NLCMMTO. **e** HAADF-STEM image, **f** ABF-STEM image of NLCMMTO along the [110] zone axis. **g** SAED pattern of NLCMMTO along the [001] zone axis. **h** PDF of NMMTO, NCMMTO and NLCMMTO. **i** RMC-fitted NLCMMTO, **j** ideal structural model of NLCMMTO. **k** FT-EXAFS analysis of Ni Mn and Cu K-edge for NLCMMTO. **l** EPR of NMMTO and NLCMMTO. **m**–**o** Ni, Mn, L-edges O K-edges for NMMTO, NCMMTO and NLCMMTO.

Three samples with different entropy values have been obtained as designed, Na_2/3_Ni_1/6_Mg_1/6_Mn_1/3_Ti_1/3_O_2_, Na_2/3_Ni_1/6_Cu_1/9_Mg_1/18_Mn_1/3_Ti_1/3_O_2_, Na_2/3_Ni_1/6_Li_1/18_Cu_1/18_Mg_1/18_Mn_1/3_Ti_1/3_O_2_, denoted as NMMTO, NCMMTO and NLCMMTO, respectively, by substituting different elements and contents. All of them exhibit similar X-ray diffraction (XRD) patterns (Fig. 1a). Most of the characteristic XRD peaks can be indexed based on P6_3_/*mmc* space group and extra peaks could be assigned to *R-*3*m* symmetry showing the main P2 with less O3 combination phase constitutes in three samples^8,22^. Besides, after introducing Li, a slight increase in the O3 phase content is observed. X-ray absorption near-edge structure (XANES) has been used to analyze the oxidation state of Ni, Mn and Cu in the three samples. The nearly unchanged absorption edge positions indicate that there is no obvious valence change by element substitution in three samples (Fig. 1b–d). However, a slight difference in the Mn spectral feature, particularly in peak intensity, is observed for NMMTO. This variation is attributed to the subtle change of the local coordination environment (Fig. S1). The morphologies of NMMTO, NCMMTO and NLCMMTO were characterized by scanning electron microscopy (SEM) and transmission electron microscopy (TEM) in Figs. S2–S5. Interestingly, NLCMMTO particles consist of multiple-layer oriented stacking nanosheets which are beneficial for facilitating Na^+^ transport^23^. Furthermore, in order to demonstrate the crystal matching of P2 and O3 on an atomic scale, high-angle annular dark field (HAADF) and annular bright field (ABF) spherical aberration-corrected transmission electron microscopy (STEM) were performed on the NLCMMTO material. The bright dots in HAADF-STEM (Fig. 1e) and the black dots in ABF-STEM (Fig. 1f) represent transition metal atoms. Meanwhile, the faint black dots in ABF-STEM correspond to Na and O atoms. The HAADF-STEM and ABF-STEM maps along the [010] crystallographic direction clearly show the adjacent crystal planes. The lattice spacing of 0.55 nm is assigned to the (002) planes of the P2 phase, indicating that P2 phase is the dominant configuration^24^. The selected-area electron diffraction (SAED) along [001] provides information on the crystalline plane of NLCMMTO as shown in Fig. 1g. Pair distribution function (PDF) analysis (Fig. 1h) shows structural distortions at the local scale, as evidenced by peak broadening and subtle shifts in the interatomic distances within the 3-9 Å range. These observations suggest that the introduction of Li and Cu in NLCMMTO leads to a defect-rich local environment, which might facilitate sodium ion transport while potentially inducing adaptive lattice responses during cycling. The Reverse Monte Carlo (RMC)-fitted PDF (Fig. 1i) reveals the average first shell TM–O (d_1_ in Fig. 1j) and second shell TM-TM (d_2_ in Fig. 1j) correlations. The extended X-ray absorption fine structure (EXAFS) spectra of transition metals in three samples were also collected to study the local environment of specific elements. The Ni, Cu and Mn Fourier-Transform (FT)-EXAFS spectra of NLCMMTO are shown in Fig. 1k, where the two main peaks correspond to the TM–O first coordination shell and the TM-TM second coordination shell, respectively. In addition, similar spectral features and close atomic distances suggest a homogeneous distribution of transition metal ions within a comparable chemical environment, consistent with the XANES results. Electron paramagnetic resonance (EPR) spectra (Fig. 1l) exhibit a pronounced increase in signal intensity for the NLCMMTO sample compared to NMMTO, indicating a higher concentration of paramagnetic centers^25,26^. This enhancement likely arises from oxygen vacancies and localized electronic states induced by charge redistribution after substituting Li and Cu. To analyze the elemental valence, the L-edges of transition metal elements were collected via soft X-ray absorption spectroscopy (SXR) in Figs. 1m, n and S6, showing that the transition metal elements in the three samples exhibit the same valence state. There is a slight difference in the O K-edge spectra of these samples as shown in Fig. 1o that, after introducing Cu, the peak intensity at 533 eV related to out-of-plane O π* increases, which might be associated with Jahn-Teller distortion introduced by Cu^27,28^. In contrast, Li incorporation in NLCMMTO slightly suppresses this peak, suggesting alleviated interlayer deformation stress in the TM layers. The energy dispersive X-ray spectroscopy (EDS-STEM) mapping of NLCMMTO indicates that elements such as Na, Ni, Cu, Mg, Mn, Ti and O are uniformly distributed within the sample (Fig. S7). Additionally, XPS spectra of NLCMMTO shown in Fig. S8 were collected, which also present the chemical valence of transition metals that is consistent with the result of SXR.

**Fig. 2:**
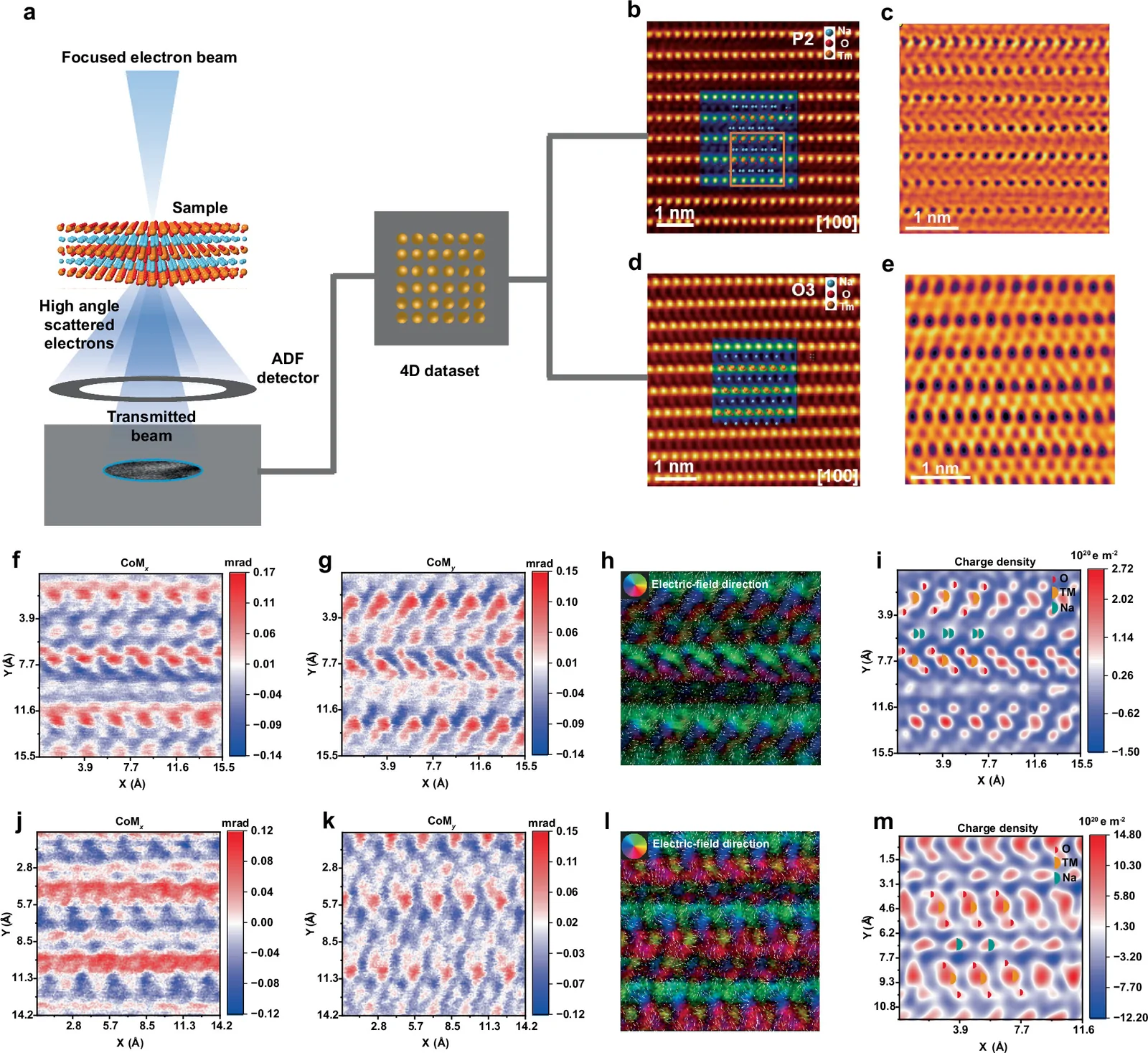
4D-STEM for NLCMMTO. **a** Schematic of the 4D-STEM working mechanism. **b**, **c** HAADF-STEM and ABF-STEM images of P2 phase in NLCMMTO. **d**, **e** HAADF-STEM and ABF-STEM images of O3 phase in NLCMMTO. **f**, **g** Centroid along the *x*-direction and along the *y*-direction for the P2 phase. **h** Atomic-scale electric field distribution for the P2. **i** Atomic-level distribution of charge density of the P2 phase. **j**, **k** Centroid along the *x*-direction and along the *y*-direction for the O3 phase. **l** Atomic-scale electric field distribution for the O3. **m** Atomic-level distribution of charge density of the O3 phase.

Four-dimensional scanning transmission electron microscopy (4D-STEM) was employed to investigate the atomic-scale electrostatic characteristics of the P2 and O3 domains in NLCMMTO. During raster scanning, a focused electron probe was sequentially positioned across the specimen, while a pixelated detector simultaneously collected position-resolved diffraction signals throughout the scan (Fig. 2a). The center of mass (CoM) of each diffraction pattern was then determined along two orthogonal directions. Within the framework derived from Ehrenfest’s theorem, the measured CoM displacement reflects the momentum transfer between the incident electrons and the specimen and can be related to the projected in-plane electric field after accounting for probe response^29,30^. Accordingly, the two-dimensional projected electric field distribution can be reconstructed from the CoM components, while the corresponding projected charge density distribution is obtained from the divergence of the electric field according to Gauss’ law. The focused ion beam (FIB) lamella-preparation procedure for STEM and the cross-sectional characterization of the specimen are provided in Figs. S9 and S10, respectively, and additional 4D-STEM-derived maps are presented in Figs. S11–S14 and Supplementary Videos 1–4. HAADF-STEM and ABF-STEM images were used to identify the atomic arrangements of the P2 and O3 domains (Fig. 2b–e). The experimentally determined CoM components along the *x*- and *y*-directions (CoM_*x*_ and CoM_*y*_) are shown in Fig. 2f, g for the P2 phase and in Fig. 2j, k for the O3 phase, respectively. Because the diffraction signals were acquired using a high-speed hybrid-pixel electron detector, rapid data collection was achieved for the beam-sensitive layered-oxide cathode material. The CoM components were subsequently converted into the projected electric field maps shown in Fig. 2h, l using the appropriate physical relationship^29,30^. The color field maps describe the local electric field direction, and both phases exhibit a periodic field distribution correlated with the atomic column arrangement, with the field generally diverging from the positively charged atomic column regions. Based on the reconstructed electric fields, the projected charge density maps were further calculated for the P2 and O3 phases (Fig. 2i, m). For both domains, the reconstructed charge density contrast alternates spatially between the atomic-column positions and the surrounding interstitial regions, showing positive and negative signals, respectively. These features represent the probe-convolved net projected charge density and contain contributions from both the nuclear and electronic charge distributions. To further evaluate the Coulombic interactions between neighboring layers in the layered oxide, the *y*-component of the electric field was extracted, and line-scan integrations were performed across four oxygen atomic layers in the P2 and O3 regions (Fig. S15). The integrated profiles show only small variations among equivalent oxygen layers in each phase, indicating a relatively uniform electrostatic distribution across the oxygen sublattice. This result suggests that the interlayer electrostatic potential landscape is globally smooth and homogeneous, with limited local fluctuations in Coulombic interactions between neighboring transition metal oxide slabs. Consistently, the projected charge density maps in Fig. 2i, m do not show pronounced localized charge accumulation across the oxygen sublattice in either phase. Such homogeneous charge distribution is important for layered oxides because spatially nonuniform interlayer electrostatic repulsion can generate localized mechanical imbalance, which may provide nucleation sites for stress concentration, layer gliding, and subsequent phase transitions. In contrast, the relatively uniform in-plane charge distribution observed here implies more even Coulombic interactions between adjacent oxide layers and therefore a reduced tendency toward localized electrostatic and mechanical perturbations. The absence of pronounced local charge localization may consequently mitigate the development of stress-concentration sites and reduce the driving force for deleterious layer gliding and phase transformations during Na^+^ extraction and insertion. Thus, the homogeneous electrostatic environment revealed by 4D-STEM provides atomic-scale evidence supporting the high mechanical integrity of NLCMMTO and may contribute to maintaining its structural stability during repeated electrochemical cycling.

### Electrochemical performance and adaptive volume change in NLCMMTO

**Fig. 3:**
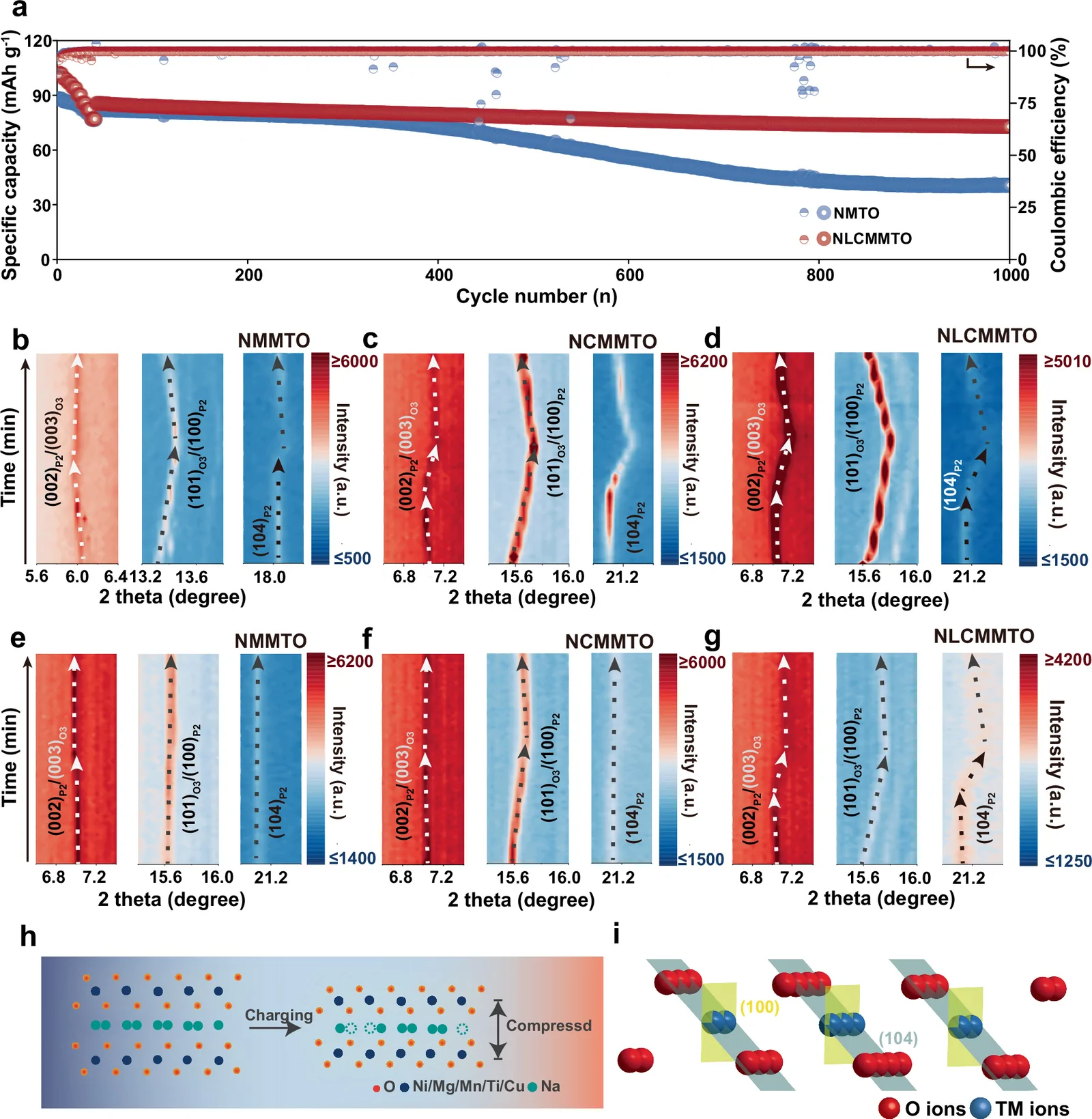
Electrochemical performance and self-adaptive volume behavior of NLCMMTO. **a** Long-term performance of NLCMMTO and NMTO coin cells at room temperature (25 ± 2 °C). (1C= 120 mA g^−1^) **b**–**d** selected in situ synchrotron-based XRD patterns collected during the first charge/discharge in the voltage range of 2.5–4.15 V at 20 mA g^−1^ of NMMTO, NCMMTO, and NLCMMTO at room temperature (25 ± 2 °C). **e**–**g** selected in situ synchrotron-based XRD pattern collected during the second charge/discharge in the voltage range of 2.5–4.15 V at 120 mA g^−1^ of NMMTO, NCMMTO, and NLCMMTO at room temperature (25 ± 2 °C). **h** Schematic picture of self-adaptive volume behavior in NLCMMTO. **i** (100) and (104) crystal plane diagram.

The electrochemical performance of Na_2/3_Ni_1/3_Mn_1/3_Ti_1/3_O_2_ (NMTO), NMMTO, NCMMTO, and NLCMMTO was systematically evaluated in Na half-coin cells over a voltage range of 2.5–4.15 V (versus Na/Na^+^) as shown in Fig. S16. As shown in Fig. 3a, the rate and long-term performance of NMTO and NLCMMTO cathodes were tested at current rates ranging from 0.1 to 5 C. Long-term cycling stability was also assessed. After 1000 cycles at 2C, the capacity retentions of NMTO and NLCMMTO are 59% (from 80.6 to 47.9 mAhg^−1^) and 82% (from 88.6 to 72.8 mAhg^−1^), respectively. NLCMMTO exhibits better cycling performance with lower capacity decay, demonstrating its improved structural and electrochemical stability. In Fig. S17–S20, the charge-discharge curves of NMTO, NMMTO, NCMMTO, and NLCMMTO reveal smoothed voltage plateaus. The differential capacity (dQ/dV) plots (Fig. S21) further confirm this observation, showing smoothed peaks, which suggest fewer and more reversible phase transitions during cycling in NLCMMTO. This structural stability is attributed to the synergistic effects of element specific functionalities that might be enabled by rational compositional design combined with high-entropy effect. The galvanostatic intermittent titration technique (GITT) was employed to further analyze the sodium-ion diffusion kinetics. As shown in Fig. S22, the sodium-ion diffusion coefficient of NLCMMTO is higher than those of the other samples, highlighting its favorable sodium-ion diffusion kinetic properties. This enhancement is beneficial for achieving high-rate performance and efficient sodium storage. In order to further understand the ion transport performance of the material, we used the bond valence site energy (BVSE) method to simulate the Na^+^ migration pathways in NLCMMTO. The simulation results are shown in Fig. S23. The sodium ions exhibit two-dimensional diffusion pathways with low migration energy barriers in both phases, indicating that the material has good ion transport performance in both phases.

Phase transformation was monitored via in situ synchrotron-based XRD over a voltage range of 2.5 V-4.15 V at 20 mA g^−1^ (Fig. 3b–d, S24, S25, S28, S29, S32 and S33). Moreover, the high resolution and intensity provided by the synchrotron beamline enables rapid data collection for in situ studies (within 1–3 min), allowing the investigation of phase evolution at an enhanced specific current of 120 mA g^−1^ (Fig. 3e–g, S26, S27, S30, S31, S34 and S35). All diffraction peaks are largely retained with slight shifts compared to the pristine state, indicating negligible structural changes during cycling. In general, (002) peak shifts towards lower angles continuously during the charging process, which is a consequence of the electrostatic repulsion between oxygen atoms after Na^+^ extraction^31^. And then, (002) peak moves back to the original position gradually during discharging. The normal behavior has been shown in NMMTO cathodes at both 20 mA g^−1^ and 120 mA g^−1^. Interestingly, after Cu substitution, at specific current of 20 mA g^−1^ NLCMMTO and NCMMTO present abnormal structural evolution that (002) left shifts at beginning of charging and then reverts to its previous position at the high voltage of 4.15 V. Such adaptive volume behavior could also be observed at higher specific current in NLCMMTO while disappearing in NCMMTO at 120 mAg^−1^. Additionally, since the position and intensity of (10 *l*) reflections are closely related to the TM-TM distances and stacking fault generation caused by the glide of TMO_2_ slabs, the (10 *l*) peaks should be carefully considered^32^. All (100) peak shifts of three samples exhibit similar trends at 20 mAg^−1^ and 120 mAg^−1^, shifting toward higher angles at the end of charging and returning to lower angles during discharge. Since the (100) plane consists solely of TM ions, the oxidation and reduction of these ions will cause a shortening or lengthening of TM-TM distances, thereby altering the lattice spacing. Even though the shift of the (100) reflection is subtle, the limited shift may be associated with sluggish redox kinetics, which is consistent with the lowest capacity observed at elevated current density in NMMTO cathodes. Notably, the (104) peaks of the three samples present different characteristics (Fig. S24–S35). At 20 mA g^−1^, no significant shift is observed throughout the entire process, except for the broadening of the (104) peak in NMMTO. In contrast, in NCMMTO and NLCMMTO, there are visible right shifts in both samples along with broadening (104) peaks. Considering that the (104) plane is composed of TM and oxygen ions, shown in Fig. 3i, it is believed that the TM–O and O–O bonds undergo changes during the electrochemical process. However, the change in the (104) peak for NMMTO and NCMMTO at 120 mA g^-1^ is similar to that of NMMTO at 20 mA g^−1^, while only NLCMMTO continues to exhibit a peak shift, reflecting the existence of structural adjustment of TM–O and O–O bonds even at higher current. Based on the evolution of the (104) reflection, the adaptive volume change may reasonably be associated with the involvement of oxygen. The contraction of the interlayer spacing involving oxygen participation at high voltage effectively mitigates internal stress accumulation, establishing a structural basis for enhanced long-term cycling stability. In addition, the Tresca stress calculation further shows that the P2/O3 intergrowth structure has lower stress accumulation than the pure P2 structure (Fig. S36), suggesting that the intergrowth region may act as a possible contributor to stress accommodation during cycling. The spontaneous contraction of the interlayer spacing, which may be related to oxygen involved charge redistribution, could further reduce stress accumulation and contribute to structural stabilization during cycling.

### Structural evolution and stress distribution

**Fig. 4:**
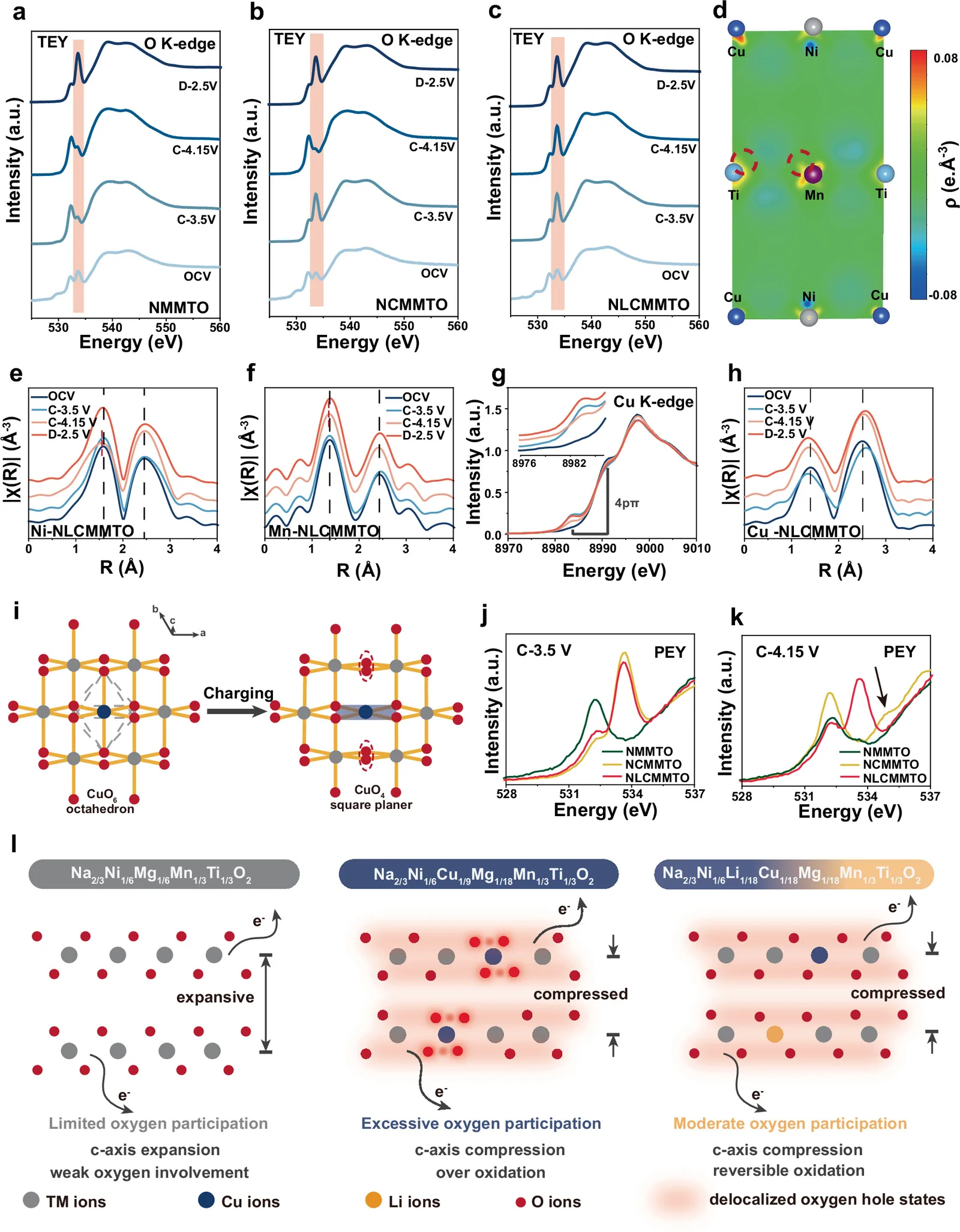
Regulated oxygen redox and lattice evolution in NLCMMTO. TEY O K-edge XAS of **a** NMMTO, **b** NCMMTO, **c** NLCMMTO electrodes collected at the pristine state (OCV), charging to 3.5 V (C-3.5), charging to 4.15 V (C-4.15), and discharging to 2.5 V (D-2.5). **d** Calculation of the electron localization function of NCMMTO. EXAFS spectra for **e** Ni, and **f** Mn at OCV, C-3.5, C-4.15 and D-2.5. **g** Normalized Cu K-edge XANES and **h** EXAFS in NLCMMTO at OCV, C-3.5, C-4.15 and D-2.5. **i** Schematic image of structure evolution of Cu. PEY O K-edge XAS of NMMTO, NCMMTO, and NLCMMTO upon charging to **j** 3.5 V; **k**, 4.15 V. **l** Schematic image of oxygen involved lattice evolution in layered oxides at highest voltage (4.15 V). All ex situ electrodes were cycled at 10 mA g^−1^ during the first cycle before disassembly and sampling at room temperature (25 ± 2 °C).

To obtain direct insight into the oxygen evolution behavior, SXR has been used to study the valence change and ligand environment by analyzing TM L-edges and O K-edge. As shown in Fig. 4a–c, NMMTO, NCMMTO, and NLCMMTO show similar O K-edges XAS spectra (TEY mode) at the original state. Upon charging to 3.5 V, a distinct peak emerges at ~533.6 eV in NCMMTO and NLCMMTO suggesting the participation of oxygen, which is associated with delocalized O 2*p* states arising from O 1 *s* → π* transitions, indicating the oxygen redox activity increases^33^. Such specific O 2*p* states could act as a “charge buffer” within the TM–O network^34^. This delocalization reduces the localization of electrons on transition metal d orbitals, might alleviate orbital degeneracy and facilitate TM–O hybridization, which could be helpful to stabilize the structural integrity^35^. As the voltage increases to 4.15 V, the 533.6 eV feature could remain in NLCMMTO, indicating the stabilization of that state upon further oxidation after introduction of Li. In contrast, the peak at 533.6 eV disappears for NCMMTO at the highest voltage. XPS is sensitive to valence electron, which is also carried out to analyze the valence states of NLCMMTO cathodes at different charge states. From Fig. S37, the M–(O–O) species have been detected at state of charging to 3.5 V further supporting the participation of oxygen^32,36–38^. The electron localization function was calculated as shown in Fig. 4d and S38. It can be seen that after Cu substitution, the electronic distribution is more delocalized, which may facilitate TM–O hybridization and charge redistribution. The function of Cu was also studied by XANES and EXAFS at different charge states. From Fig. S39–S42, due to the reduced electron density upon oxidation during the charging process, the TM-TM distance in NMMTO is shortened shown in Ni and Mn EXAFS spectra, while the TM–O bond length presents no obvious variation. Compared with NMMTO, the Ni–O/Mn–O and Ni-TM/Mn-TM distances in NLCMMTO change slightly, reflecting stable structure evolution during Na^+^ insertion and extraction. Besides, the shortening of TM–O bond length upon charging to 4.15 V suggests enhanced TM–O interaction at high states of charge, consistent with the involvement of oxygen in charge compensation during charging and discharging (Fig. 4e, f). There are obvious changes at different charge states in Cu XANES and EXAFS spectra (Fig. 4g, h). The XANES spectrum near the Cu K-edge is consistent with a CuO_6_ structure for primary state. With the charging process, the absence of the peaks at ∼8983 and 8991 eV which correlate with 1 *s* → 4*pπ* transitions verifies the generation of planar CuO_4_ characterized by stronger in-plane oxygen coordination which could be observed in NLCMMTO and NCMMTO (Fig. 4g and S43)^39,40^. The Cu EXAFS spectrum depicts the atomic local environment variation directly. As shown in Fig. 4h, during oxidation process the Cu–O bond length reduces accompanied by increasing Cu-TM distance, which could be related to increasingly square planar CuO_4_ local environment, possibly due to a weakening of the axial Cu–O interactions. A hypothetical structural evolution is schematically illustrated in Fig. 4i: during charging process, the axial Cu–O coordination may become weakened, which could allow local structural rearrangement accompanied by a reduced O–O distance (the region outlined by the dashed box) and altered TM–O–TM bond angles. Such changes might provide a structural basis for enhanced TM–O hybridization. Thus, the interlayer electrostatic repulsion caused by oxygen could be weakened, and then the interlayer distance will be reduced, which is also consistent with the evolution of (104) reflection in Fig. 3. To further understand the function of Li, PEY SXR spectra of NCMMTO and NLCMMTO electrodes at different states of charge were collected shown in Fig. 4j, k. In NCMMTO, the ~533.6 eV feature diminishes upon further charging to 4.15 V, accompanied by the emergence of a higher energy component ( ~ 534.9 eV), suggesting further oxidation of lattice oxygen accompanied by localized oxygen interactions which might be harmful to the structure integrity and Na^+^ transfer kinetics due to the irreversibility. For NLCMMTO, the ~533.6 eV feature persists even at 4.15 V without a significant shift to higher energy, which is often associated with higher reversibility. Beyond atomic scale structural evolution, the stress change in whole materials can also be compared by Tresca stress calculation of NMMTO and NLCMMTO in Fig. S44. Compared with the original state, charging up to 4.15 V both materials are subjected to greater stress. Although both of them exhibit similar stress distribution that stress concentration occurs at the edges, whole Tresca stress in NLCMMTO is lower and more uniform than that in NMMTO (Fig. S44). Figure 4l shows the functional mechanism of Cu and Li in regulating the structural and electronic evolution.

### The charge reconfiguration in NLCMMTO

**Fig. 5:**
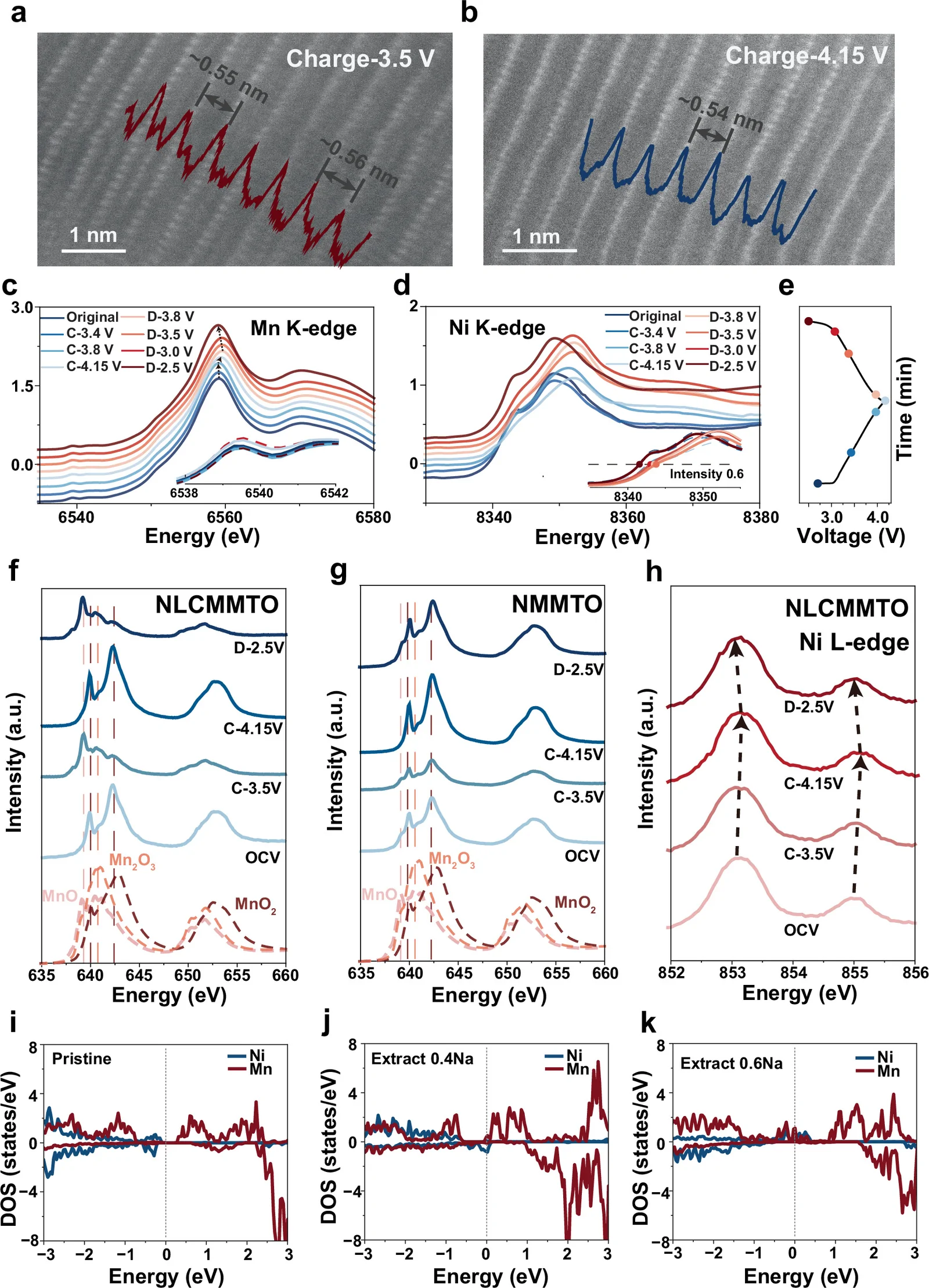
The charge reconfiguration in NLCMMTO. **a** HAADF-STEM pattern of NLCMMTO charging to **a** 3.5 V, **b** 4.15 V during first cycle at 10 mA g^−1^. **c** Mn K-edge, **d** Ni K-edge of NLCMMTO electrodes collected during the first cycle at 60 mA g^−1^ and paused at the indicated voltages at room temperature (25 ± 2 °C). **e**, Corresponding charge/discharge voltage profile. Mn L-edge of **f** NLCMMTO and **g** NMMTO and **h** Ni L_3_-edge spectra of NLCMMTO collected at selected states during the first cycle at 10 mA g^−1^. DOS calculation of simplified P2-based NLCMMTO model at **i** pristine state, **j** 0.4 Na extracted state and **k** 0.6 Na extracted state. (The structural models shown in Figs. 5i–k are provided in Supplementary Data 1, Supplementary Data 2 and Supplementary Data 3, respectively).

To make it easier to observe the self-adaptive volume change behavior visually, STEM was carried out to measure the lattice distance of NLCMMTO cathodes at 3.5 V and 4.15 V separately. As shown in Fig. 5a and 5b, the interlayer distance increases slightly after Na^+^ extraction at a charging voltage of 3.5 V. And then when charging to 4.15 V, the distance reduces. Since Cu and Ti show no obvious valence changes at 2.5–4.15 V operating window, evidenced by XAS showing no detectable absorption-edge energy shift across different states of charge (Fig. S45), Ni and Mn redox couples have been the focus of investigation. To investigate the charge compensation, ex situ XAS experiment was conducted via collecting the Ni and Mn spectra of NLCMMTO cathodes in battery at selected potentials. In general, the Mn pre-edge spectra reflecting the dip-forbidden 1*s*-3*d* transitions could partly reflect the valence state of Mn. As shown in Fig. 5c, the Mn pre-edge presents broad and weak indicating that Mn is primarily located in octahedral coordination sites. At the beginning of charging, there is a slight left shift at 6559 eV together with decreased intensity of C-3.4 V and C-3.8 V pre-edges, suggesting a subtle reduction of Mn. When charging to 4.15 V, the intensity of pre-edge peak is increased. During the following discharging process, the intensity of peak decreases with reducing Mn valence. During the entire Na⁺ insertion and extraction process, the coordination environment of Mn remains stable evidenced by the similar shapes of pre-edge features observed at different charge states. Ni K-edge XANES spectra reveal changes in the Ni environment at 3.8 V, evidenced by a continuous change in the shape of the absorption edge. Additionally, Ni XANES spectrum at the end of discharge returns to nearly the same position as that of the pristine material, showing the highly reversible ability of Ni. Notably, the spectrum of Ni at D-3.5 V seems like be in a higher oxidation state than its D-3.8 V during the reduction process due to its slight right shift. Considering that the L-edge of transition metals provides greater sensitivity to variations in valence electron state and that the weak pre-edge intensity of octahedrally coordinated Mn at the K-edge limits accurate evaluation of subtle valence changes, the SXR and XPS spectra were also collected in Fig. 5f, g and S46. It can be seen that the evolution of Mn L-edge XAS spectra of NLCMMTO and NMMTO (Fig. 5f, g) clearly reflects changes in Mn valence states across different electrochemical states. Compared with NMMTO, where the Mn peak shape and intensity remain nearly unchanged, NLCMMTO exhibits a Mn reduction trend during charging. In contrast to the relatively high Mn oxidation state in the pristine state (OCV), the spectral weight redistribution at C-3.5 V suggests an overall evolution of Mn toward lower oxidation states, followed by oxidation to a higher oxidation state at 4.15 V. Upon discharge, Mn of D-2.5 V is subsequently reduced again. The abnormal redox behavior of Mn observed during charging, as shown in Fig. 5f appears to correlate with the anomalous Ni redox behavior during discharge in Fig. 5d, implying a potential charge compensation relationship between Ni and Mn, which will be discussed later.

Ni TEY SXR L_3_-edge (Fig. 5h) and Ni 2p XPS spectra (Fig. S47) were collected to reveal the valence evolution of nickel during electrochemical cycling. The features of L_3_-edge shown in Fig. 5h shift towards higher energy at charging process, showing the oxidation of Ni and move back to the original position at the end of cycling. At OCV, the Ni 2*p*_3/2_ peak is centered closer to the Ni^2+^ reference shown in Fig. S47. Upon charging to 4.15 V, the peak shifts toward higher binding energy, consistent with an increase in Ni oxidation state (toward Ni^3+^)^41^. This shift partially reverses after discharging to 2.5 V, suggesting a Ni reduction process which is consistent with the results of SXR. This partial irreversibility likely arises from surface reactions with the electrolyte, as XPS is inherently surface sensitive. The combined insights from Mn and Ni L-edge XAS spectra indicate that both Mn and Ni actively participate in the charge compensation mechanism during cycling. The density of states (DOS) was calculated for studying the charge compensation of NLCMMTO with the corresponding optimized atomic structures provided in Supplementary Data 1-6. The DOS near the Fermi level reflects the availability of electronically accessible states that can participate in charge compensation. As shown in the Figs. 5i–k and S48, the valence state of Ni ions in the initial structure is relatively close to the Fermi level, while the valence band of Mn ions gradually moves upward during the desodiation process. In the early stage of desodiation process, Ni is preferentially oxidized, and then Mn ions further participate in the entire redox process.

**Fig. 6:**
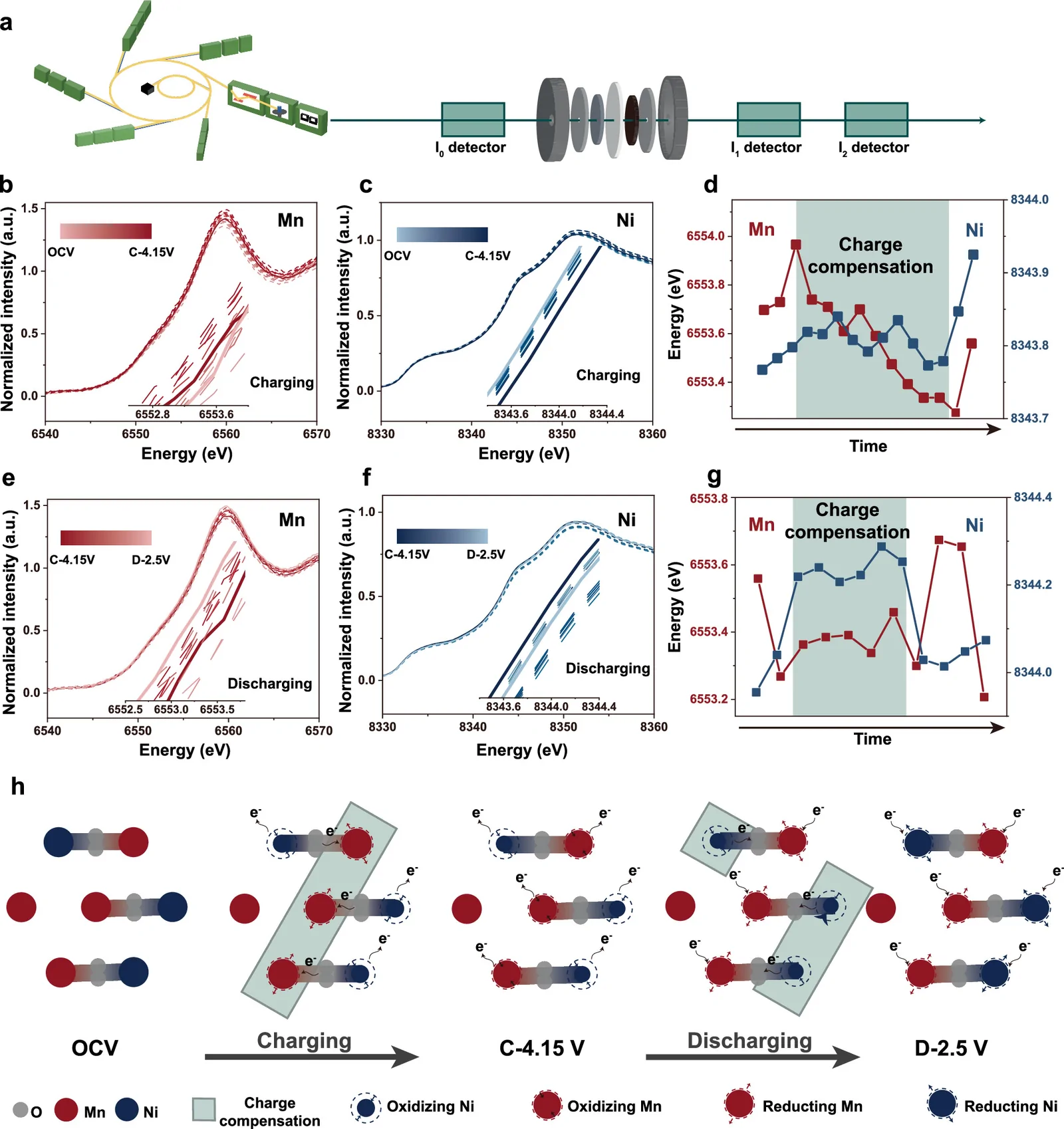
In situ XAS analysis of the charge compensation mechanism of Mn and Ni in NLCMMTO. **a** Schematic of the in situ battery XAS experiment set-up at ANSTO. In situ XANES spectra of (**b**) Mn (**c**) Ni K-edges of NLCMMTO collected during charging at 100 mA g^−1^ and room temperature (25 ± 2 °C). **d** The variation of energy for NLCMMTO during charging process. In situ XANES spectra of (**e**) Mn (**f**) Ni K-edges of NLCMMTO collected during discharging at 100 mA g^−1^ and room temperature (25 ± 2 °C). **g** The variation of energy for NLCMMTO during discharging process. **h** Schematic illustration of charge compensation between the TM ions.

To verify the charge compensation between Ni and Mn, an in situ XAS experiment has been carried out at ANSTO. To make the valence change clearer, the energy corresponding to normalized intensity of 0.6 for each spectrum has been selected. Figure 6a shows a schematic of the in situ XAS setup for batteries used to monitor real-time valence state changes of Mn and Ni during electrochemical cycling. Fig. 6b–g present the XANES spectra and edge shift trends for Mn and Ni during charging and discharging. Figure 6h further illustrates the evolution of the charge compensation mechanism via schematic diagrams. During charging, the K-edge position of Ni continues shifting right at 4.15 V cut-off (Figure 6c), indicating a continuous increase in the average oxidation state of Ni. In terms of Mn, the absorption edges shift towards lower energy at start of charging process, indicating reduction of Mn, which is consistent with the results of ex situ SXR. When the voltage reaches to 4.15 V the valence of Mn increases normally. Such fast and closely matched charge compensation possibly arises from Ni–O–Mn framework with delocalized O 2*p* states, which may enable delocalized charge compensation through the hybridized O 2*p*-TM band. This enhances the covalency of the TM–O bonds through stronger hybridization. Consequently, the modified bonding reduces the Na^+^ migration energy barrier and then achieves the improvement of rate performance. During discharge (Fig. 6e, f), the Mn K-edge shifts back toward lower energy, indicating the reduction of Mn. Simultaneously, Ni exhibits a slight shift toward higher energy at the beginning of discharging process and then moves towards lower energy, suggesting partial Ni reduction. The energy trends in Fig. 6g confirm that Mn reduces rapidly in the early stages of discharge, while Ni follows with a delayed response. This mirrors the behavior observed during charging, again highlighting the sequential and complementary nature of redox activity between Mn and Ni. The energy shifts of Mn and Ni K-edges in Fig. 6d, g clearly illustrate the cooperative charge compensation process involving both elements. In particular, the shaded regions show a coupled and complementary evolution of the Mn and Ni K-edge energies, with the increase in one generally accompanied by a decrease in the other, suggesting a redistribution of the relative redox contribution between the two species. Such synergy may help distribute the redox burden and could therefore contribute to enhanced structural stability and electrochemical reversibility.

Figure 6h visually summarizes this mechanism. As charging proceeds, electrons are progressively extracted from the structure, with Ni being oxidized first. Through the Ni–O–Mn bridging framework, partial electron transfer involving Mn occurs, and at the end of charging, Mn is also oxidized. Upon discharge, electrons re-enter the system, and Mn and Ni are reduced sequentially. The green area highlights the cooperative contribution of both elements to maintaining charge balance. The XAS data and schematic in this figure clearly reveal the dynamic valence changes of Ni and Mn during electrochemical cycling. This complementary behavior between Mn and Ni might be helpful to facilitate efficient charge compensation, helping to mitigate structural strain and improve cycling stability. Additionally, the charge reconfiguration in Ni–O–Mn could adjust the repulsive force between interlayer oxygen and stabilize the lattice framework. Real time tracking via in situ XAS spectra provides critical insights into the redox dynamics of complex electrode materials and supports the rational design of cathode materials for SIBs.

In summary, we have developed a layered oxide cathode with long-life cycling and satisfactory rate performance based on high-entropy concept, NLCMMTO, which exhibits self-adaptive volume regulation governed by the charge reconfiguration of intralayer oxygen and TM ions after generating the TM–O–TM framework with delocalized O 2*p* states, which suppresses interlayer repulsion and minimizes structural distortion under high voltage. The TM–O–TM framework with delocalized O 2*p* states boosts Na⁺ transport and redox kinetics, offering enhanced rate performance, which is verified by in situ XAS, SXR and XPS. Additionally, high mechanical integrity caused by even electric field distribution observed by 4D-STEM and reduced dynamic stress change confirmed by theoretical calculations offer enhanced long-cycling performance for sustainable SIBs. Structural evolution from the macroscopic to the microscopic, was systematically observed through advanced characterization methods and was rationalized by theoretical calculations. These combined features enable NLCMMTO to deliver long cycle life with minimal stress, offering a blueprint for developing next-generation oxide cathodes that link electrochemical stability, resource abundance, and scalability, reinforcing the role of SIBs in sustainable energy infrastructures.

## Methods

### Preparatinon of transition metal oxides

Na_2/3_Ni_1/3_Mn_1/3_Ti_1/3_O_2_, Na_2/3_Ni_1/6_Mg_1/6_Mn_1/3_Ti_1/3_O_2_, Na_2/3_Ni_1/6_Cu_1/9_Mg_1/18_Mn_1/3_Ti_1/3_O_2_, Na_2/3_Ni_1/6_Li_1/18_Cu_1/18_Mg_1/18_Mn_1/3_Ti_1/3_O_2_, were synthesized via solid-state reaction. Stoichiometric amounts of Na_2_CO_3_ (Aladdin, 99.5% purity), MgO (Aladdin, 99.0% purity), TiO_2_ (Aladdin, 99.0% purity), CuO (Aladdin, 99.0% purity), Li_2_CO_3_ (Aladdin, 99.0% purity), NiO (Aladdin, 99.0% purity), and Mn_2_O_3_ (Aladdin, 98.0% purity) were weighed, with a 10% excess of Na_2_CO_3_ to compensate for volatilization at high temperatures). The precursor materials were ground in an agate mortar for 45 min to ensure uniform mixing. After thorough grinding, the powder was compacted into pellets under a pressure of 16–19 MPa. The compacted material was then transferred to a muffle furnace and sintered at 1000 °C for 15 h in an air atmosphere, followed by natural cooling to room temperature to obtain the base material.

### FIB-STEM sample preparation

In the SEM window, a relatively flat surface area was identified. An electron-beam-induced deposition was then performed to deposit a W metal protective layer with dimensions of 10 µm in length, 2 µm in width, and 0.2 µm in height on the surface. Subsequently, an ion-beam-induced deposition was utilized to cover the same area with an additional 2 µm thick W protective layer. Next, Ga ion beams were employed to etch pits on both the upper and lower sides of the target region, followed by a U-cut. The target lamella was then extracted using a micromanipulator and welded onto a FIB-dedicated grid. Finally, the lamella was carefully thinned and polished using an ion beam to obtain a FIB-STEM sample that is amorphous-free and electron-beam transparent. The sample preparation process is shown in Figure S9.

### Characterizations

The samples were analyzed by X-ray diffraction (XRD) using a Bruker D8 Advance diffractometer with a Cu Kα radiation source (*λ* = 1.5418 Å) in the range of 2*θ* = 10°–80°. The crystal structure was analyzed by the Rietveld method using GSAS-‖ software. The morphological and structural features were characterized by scanning electron microscopy (SEM, JEOL SU-8020, 10 kV). In situ synchrotron-based X-ray diffraction and X-ray absorption spectroscopy (XAS) of battery experiments were conducted by modified standard CR2032 coin cell configuration. Circular apertures 5 mm in diameter were punched at the centers of both sides of the stainless-steel casing and sealed with Kapton film to provide X-ray transparent windows. The cathode composite was positioned within the X-ray beam path to enable direct probing of the active material during electrochemical cycling. Each cell contained one single-side coated cathode disc of 10 mm diameter, one sodium metal anode disc of 12 mm diameter and one glass fiber separator of 16 mm diameter. The cathode coating faced the separator. An electrolyte volume of 50 μL was added to each cell. The cell was galvanostatically cycled between 2.5–4.15 V at different rates using a battery testing system LANDdt. Synchrotron radiation XAS measurements were conducted at the wiggler XAS beamline (12ID) at the Australian Synchrotron in Melbourne, by using a set of liquid N_2_-cooled Si (311) monochromator crystals. The soft X-ray absorption spectroscopy (SXR) measurements were performed at the Australian Synchrotron in Melbourne. The morphological and structural peculiarities were characterized by transmission electron microscopy (TEM, JEOL JEM-2011, 200 kV). The HAADF-STEM, EDS, and 4D-STEM experiments were performed on a JEM-ARM300F2 instrument, which is equipped with a cold field emission gun and a 12-pole STEM monochromator corrector, and has a spatial resolution of ~0.6 Å. The instrument operates at 300 keV.

For ex situ XAS measurements, the cells were disassembled in an Ar-filled glovebox at room temperature. The electrodes were directly sealed between Kapton films inside the glovebox without washing and were stored under an inert atmosphere until transport. The sealed samples were transported to the beamline at room temperature and measured without deliberate air exposure before loading. For ex situ SXR measurements, the cells were disassembled in an Ar-filled glovebox at room temperature. The harvested electrodes were rinsed with DEC inside the glovebox, dried under the glovebox atmosphere, and mounted onto the dedicated ANSTO SXR sample holders. The mounted samples were sealed in vacuum bags, stored and transported at room temperature, and opened only immediately before loading into the beamline chamber.

During the HAADF image acquisition, the convergence angle was 21 mrad, and the camera length was 20 cm, and the corresponding collection angle ranged from 27 to 110 mrad. EDS spectra were collected using dual silicon drift detectors (SDDs). The 4D-STEM data acquisition was conducted at an acceleration voltage of 300 kV, with a dwell time of 100 µs. The probe size was set to 8 °C, and the camera length was 30 cm. A Dectris Arina 4D-STEM hybrid pixel electron detector (512 × 512 pixels) was employed for the measurements.

### Electrochemistry

In this manuscript we follow battery conventions and use “anode” and “cathode”, which correspond to the “negative electrode” and “positive electrode”, respectively. The electrochemical performance of half cells was evaluated by using coin cells (CR2032). The cells were fabricated in an argon-filled glovebox. (1) Electrode preparation: the cathode material, Super P carbon (TIMCAL Graphite & Carbon, 96%), and polyvinylidene fluoride (PVDF, Thermo Fisher Scientific, average molecular weight ranging from 250 and 450 kDa) binder were dispersed in N-methyl pyrrolidone (NMP, Aladdin, 99.9%) in a ratio of 7:2:1 by mass. Slurries were stirred for 30 mins in the MSK-SFM−12M Micro-vibration ball mill (300 rpm, Shenzhen Kejing). The resulting slurry was uniformly single-side coated onto the aluminum foil (15 μm, 99.6%, Hubei Fengsheng Electronic Technology Co., Ltd.) and transferred to a vacuum oven at 80 °C overnight. The electrodes were punched into 10 mm diameter disks using the MSK-T10 manual slicer (Shenzhen Kejing). The mass loading of the cathode material in the resulting cathode pellet was 2-3 mg/cm^2^. (2) Battery assembly: sodium metal sheet (99.9 %, Sigma-Aldrich) was used as a counter electrode, glass fibers was used as a diaphragm (Whatman GF/A, 260 μm thickness, pore size of 1.6 μm, punched into disks with a diameter of 16 mm), and 1.0 M NaClO_4_ (ethylene carbonate /diethyl carbonate = 1:1 + 5 vol % fluorinated ethylene carbonate) (Duoduo Chemical Technology Co., Ltd. NC-001) was used as electrolyte. Sodium metal sheet was prepared in an Ar-filled glovebox with H₂O and O₂ levels below 0.1 ppm. The surface oxide layer of the sodium metal was carefully removed using a clean blade, after which the freshly exposed sodium was pressed and punched into 12 mm diameter, ~0.5 mm thickness disks corresponding to an areal sodium loading of approximately 48 mg cm^−2^. Each Na disc was freshly prepared immediately before cell assembly. For the sodium-metal half cells, the massloading and the capacity ratio between the negative and positive electrodes were approximately 16–24 and 180–270, respectively. 100 μL of electrolyte was added to each conventional coin cell. (3) Battery testing: the coin cells were tested galvanostatically for charge/discharge on a NEWARE Battery Test System (MIHW-200–160CH, Shenzhen, China) in the range of 2.5–4.15 V (1 C = 120 mA g^–1^) at room temperature (25 ± 2 °C). GITT measurement was performed on the Neware test system.

### DFT calculations

All results of calculations in this work were performed in the framework of the density functional theory (DFT) with the projector augmented plane-wave method, as implemented in the Vienna ab initio simulation package (VASP)^42^. The valence electron and core ion interactions were described by the generalized gradient approximation (GGA) with the Perdew–Burke–Ernzerhof exchange–correlation function^43^. To mitigate the electron self-interaction, we adopted the GGA + U method with each *U* value calibrated for the corresponding oxides. For Mn and Ni, the U corrections are 3.9 eV and 6.2 eV, respectively^44^. The cut-off energy for a plane wave is set to 520 eV. The energy criterion is set to 10^−5^ eV in the iterative solution of the Kohn-Sham equation. The Brillouin zone integration is performed using a 5 × 5 × 2 k-mesh. All the structures are relaxed until the residual forces on the atoms have declined to less than 0.02 eV/Å. It should be noted that the present DFT calculations were performed using ideal periodic unit-cell models, which do not fully capture the multiphase interfaces, local disorder, or vacancies present in the experimental material. Therefore, the calculated DOS and energetic parameters are primarily used to identify relative trends and electronic-structure evolution among different configurations, rather than as absolute quantities for strict quantitative comparison with experiment.

### Bond valence site energy (BVSE) calculations

BVSE calculations were performed with the softBV program^45,46^ using structural models obtained from the Rietveld refinement. The energies of different Na sites in the crystal structure were calculated against a 3D grid of points with 0.1 Å resolution using the transferable Morse-type softBV force field. Na-ion migration pathways were identified with the regions of low BVSE.

## Supplementary information

Supplementary Information

Description of Additional Supplementary Files

Supplementary Data 1-6

Supplementary Video 1

Supplementary Video 2

Supplementary Video 3

Supplementary Video 4

Transparent Peer Review file

## Source data

Source Data

## Data Availability

The source data generated in this study are provided in the Source Data file. Source data are provided with this paper. The structural models used in the calculations are provided as Supplementary Data 1–6, and the associated supplementary videos are provided as Supplementary Videos 1–4. Source data are provided with this paper.
