# Supplementary material for: Self-adaptive layered oxide cathodes driven by charge reconfiguration for sodium-ion batteries
Source: Nat Commun. 2026 Sep 28;17:11197. doi: 10.1038/s41467-026-78040-8 (PMC13714294; doi:10.1038/s41467-026-78040-8)
Supplement: Supplementary file 1 [file 41467_2026_78040_MOESM1_ESM.pdf]

Self-adaptive Layered Oxide Cathodes Driven by Charge Reconfiguration for Sodium-ion Batteries

Supplementary Information

Jia-Yang Li[1,2](李佳阳), Ying Guo[3](郭英), Xin Wang[2](王鑫), Wei Kong Pang[2,*], Hai-Yan Hu[1](胡海燕), Yan-Fang Zhu[1,*](朱燕芳), Diancheng Chen[4](陈典诚), Xin-Yu Liu[1](刘欣雨), Qin-Fen Gu[5](顾勤奋), Bernt Johannessen[2,5], Xiaobo Zheng[6](郑小波), Yang Sun[4](孙洋), Shaobo Cheng[3,*](程少博), Yifei Yuan[1](袁一斐), Chao Wu[7,*](吴超), Jia-Zhao Wang[2](王佳兆), Shi-Xue Dou[7](窦世学) and Yao Xiao[1,8,*](肖遥)

[1]College of Chemistry and Materials Engineering, Wenzhou University, Wenzhou, 325035 P. R. China.
[2]Institute for Superconducting and Electronic Materials, Australian Institute for Innovative Materials, Innovation Campus, University of Wollongong, NSW 2500, Australia.
[3]Henan Key Laboratory of Diamond Materials and Devices, Key Laboratory of Integrated Circuit, Ministry of Education, School of Physics, Zhengzhou University, Zhengzhou 450052, China.
[4]School of Materials, Sun Yat-sen University, Shenzhen 518107, China.
[5]Australian Synchrotron, ANSTO, Clayton VIC 3168, Australia.
[6]Centre for Clean Energy Technology, School of Mathematical and Physical Sciences, Faculty of Science, University of Technology Sydney, Sydney, NSW 2007, Australia.
[7]Institute of Energy Materials Science (IEMS), University of Shanghai for Science and Technology, Shanghai 200093, China.
[8]State Key Laboratory of New Textile Materials and Advanced Processing, Wuhan Textile University, Wuhan 430200, P. R. China.

*Corresponding authors. E-mail: wkpang@uow.edu.au; yanfangzhu@wzu.edu.cn; chengshaobo@zzu.edu.cn; chaowu@usst.edu.cn; xiaoyao@wzu.edu.cn

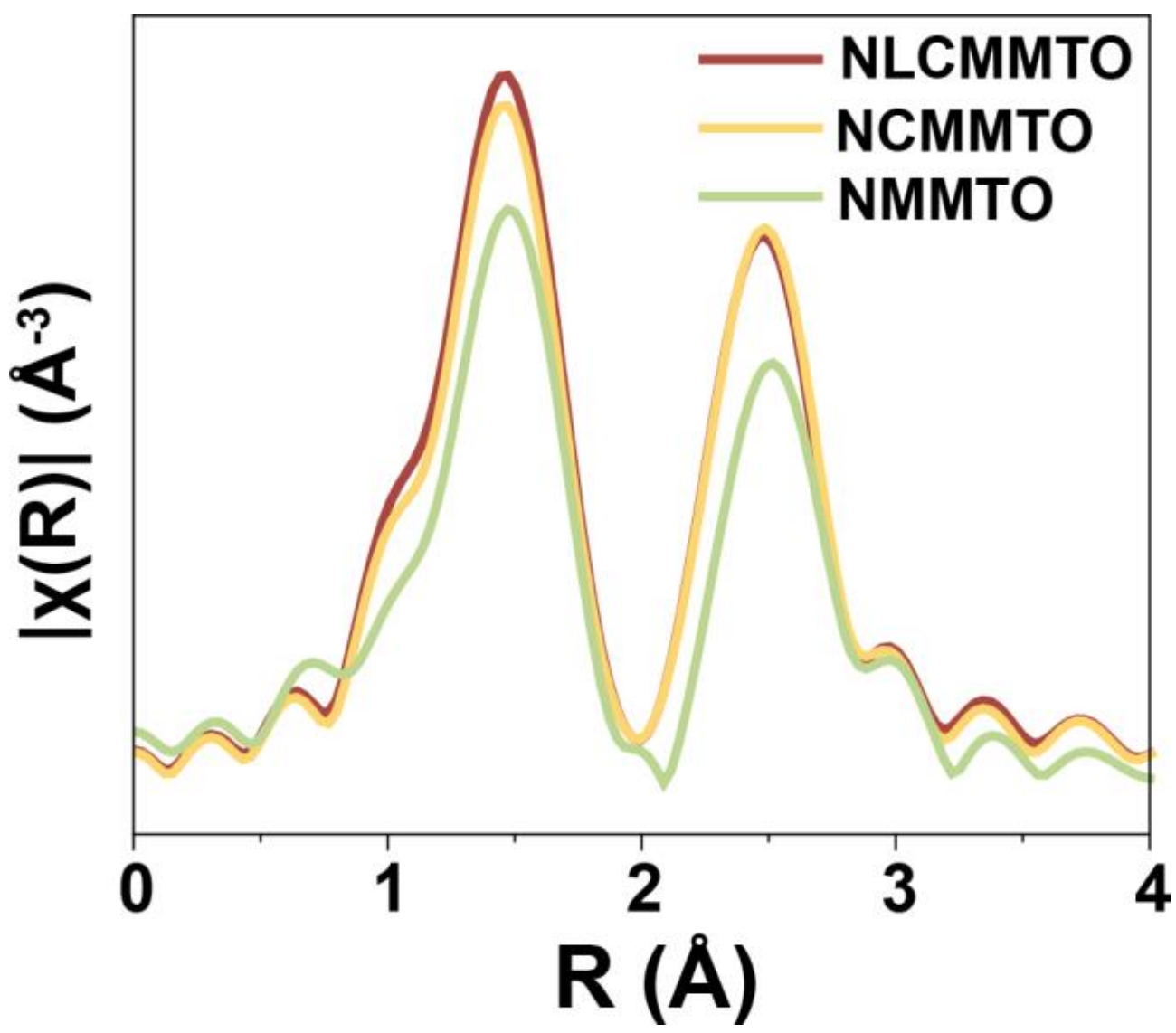

Supplementary Figure S1 Fourier-transform EXAFS spectra of Mn in R space in NMMTO, NCMMTO and NLCMMTO.

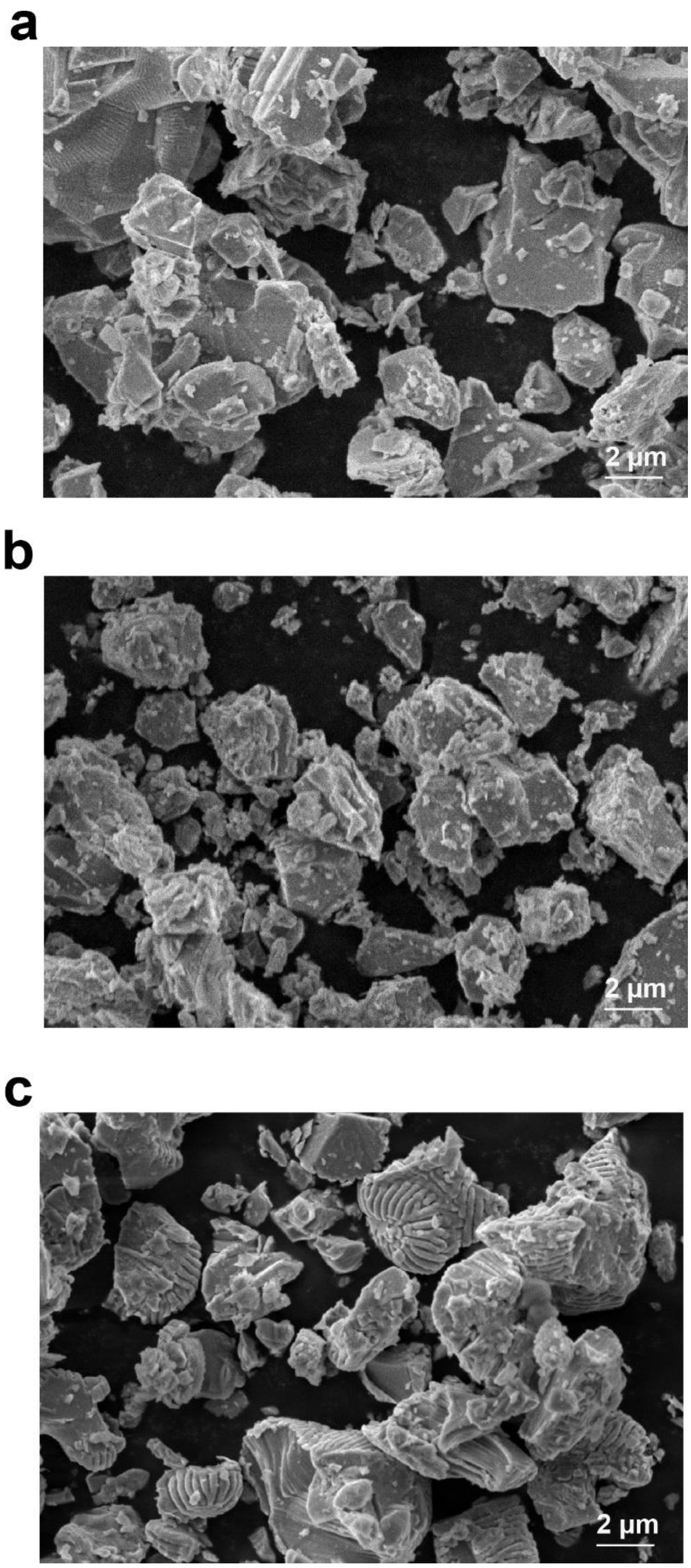

Supplementary Figure S2 SEM images of a) $Na_{2/3}Ni_{1/6}Mg_{1/6}Mn_{1/3}Ti_{1/3}O_2$ (NMMTO), b) $Na_{2/3}Ni_{1/6}Cu_{1/9}Mg_{1/18}Mn_{1/3}Ti_{1/3}O_2$ (NCMMTO), c) $Na_{2/3}Ni_{1/6}Li_{1/18}Cu_{1/18}Mg_{1/18}Mn_{1/3}Ti_{1/3}O_2$ (NLCMMTO).

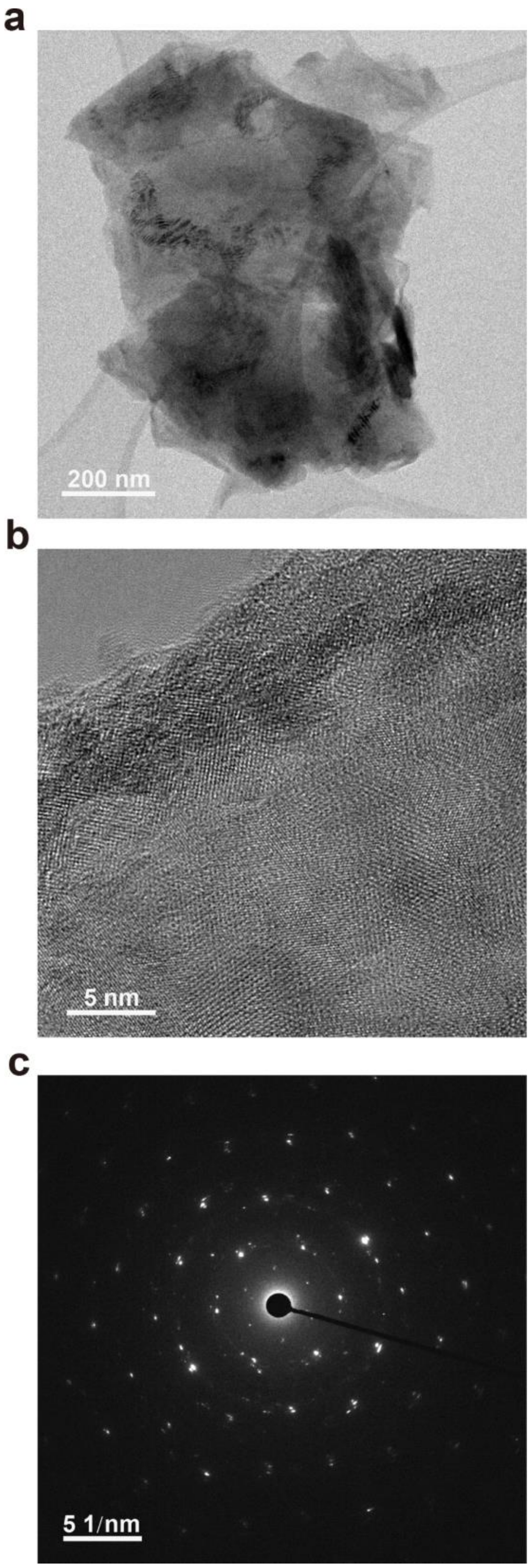

Supplementary Figure S3 a) TEM, b) HRTEM, and c) SAED images of NMMTO.

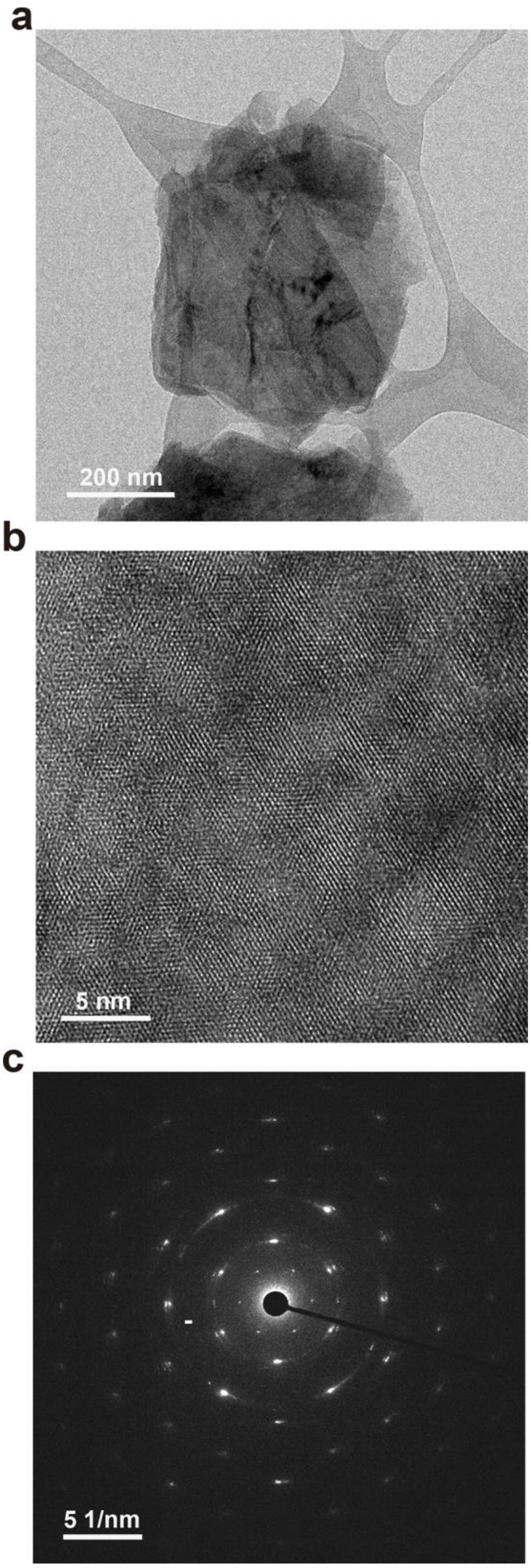

Supplementary Figure S4 a) TEM, b) HRTEM, and c) SAED images of NCMMTO.

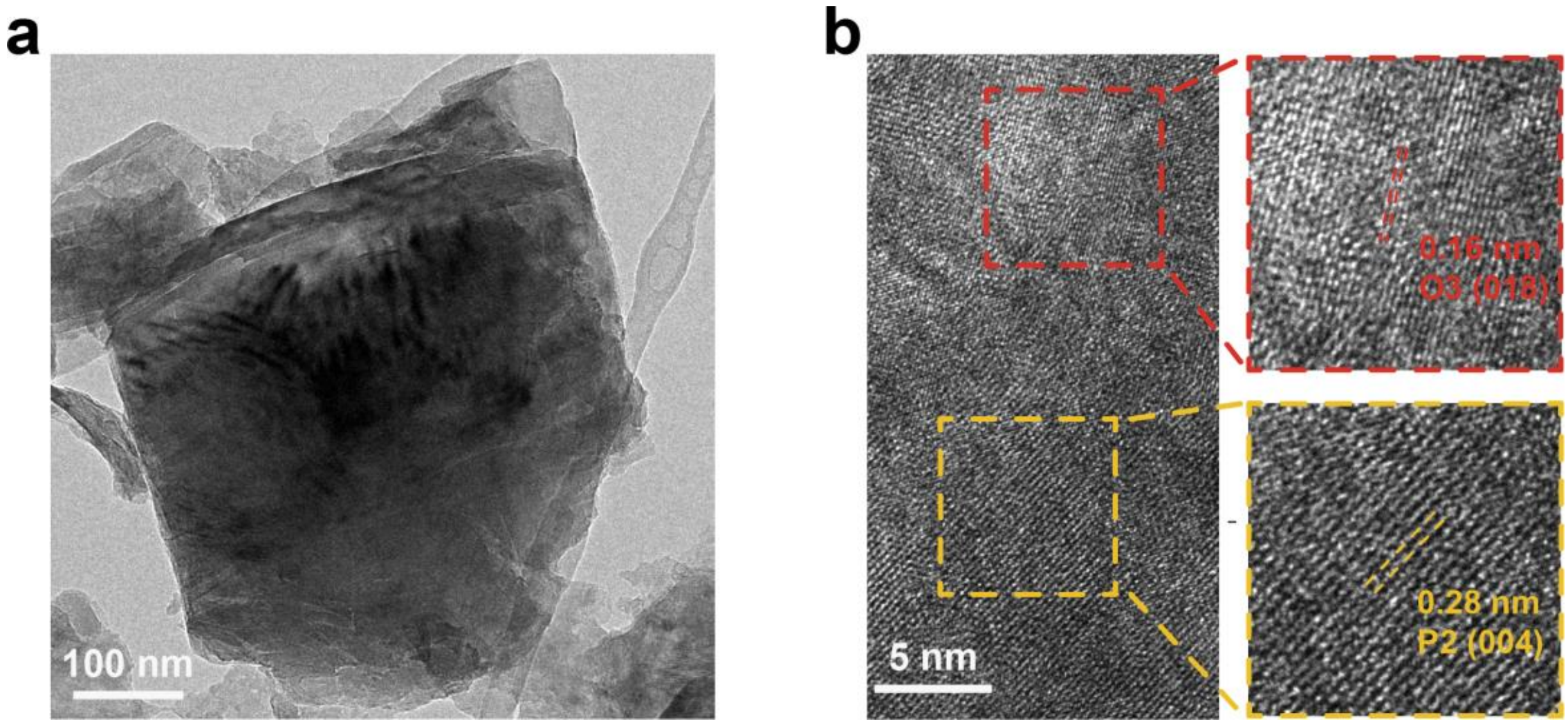

Supplementary Figure S5 a) TEM, b) HRTEM images of NLCMMTO.

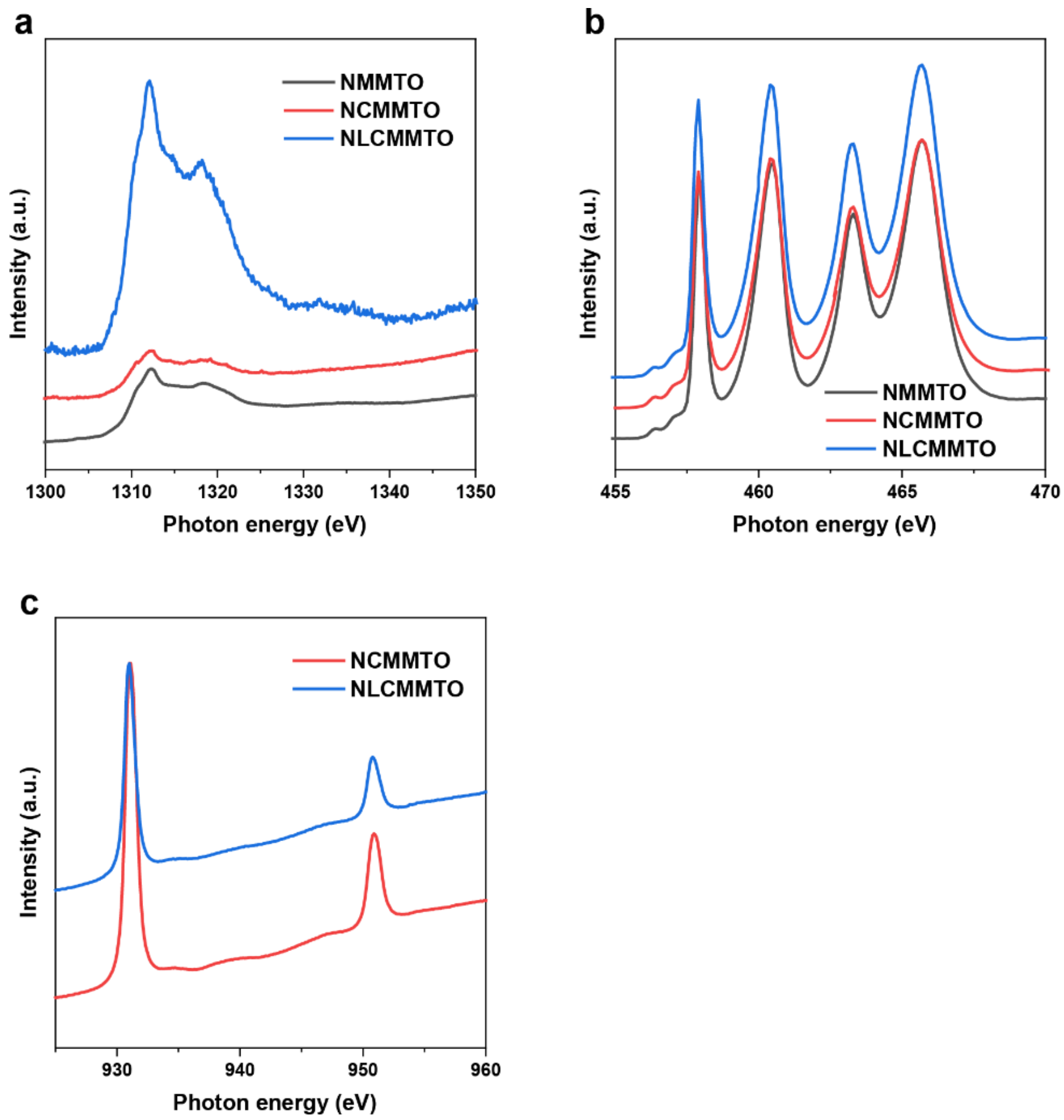

Supplementary Figure S6 SXR of a) Mg L-edges, b) Ti L-edges for NMMTO, NCMMTO, and NLCMMTO and c) Cu L-edges for NCMMTO, and NLCMMTO.

All three samples present similar SXR spectra, demonstrating that the substitution does not greatly alter their valence states.

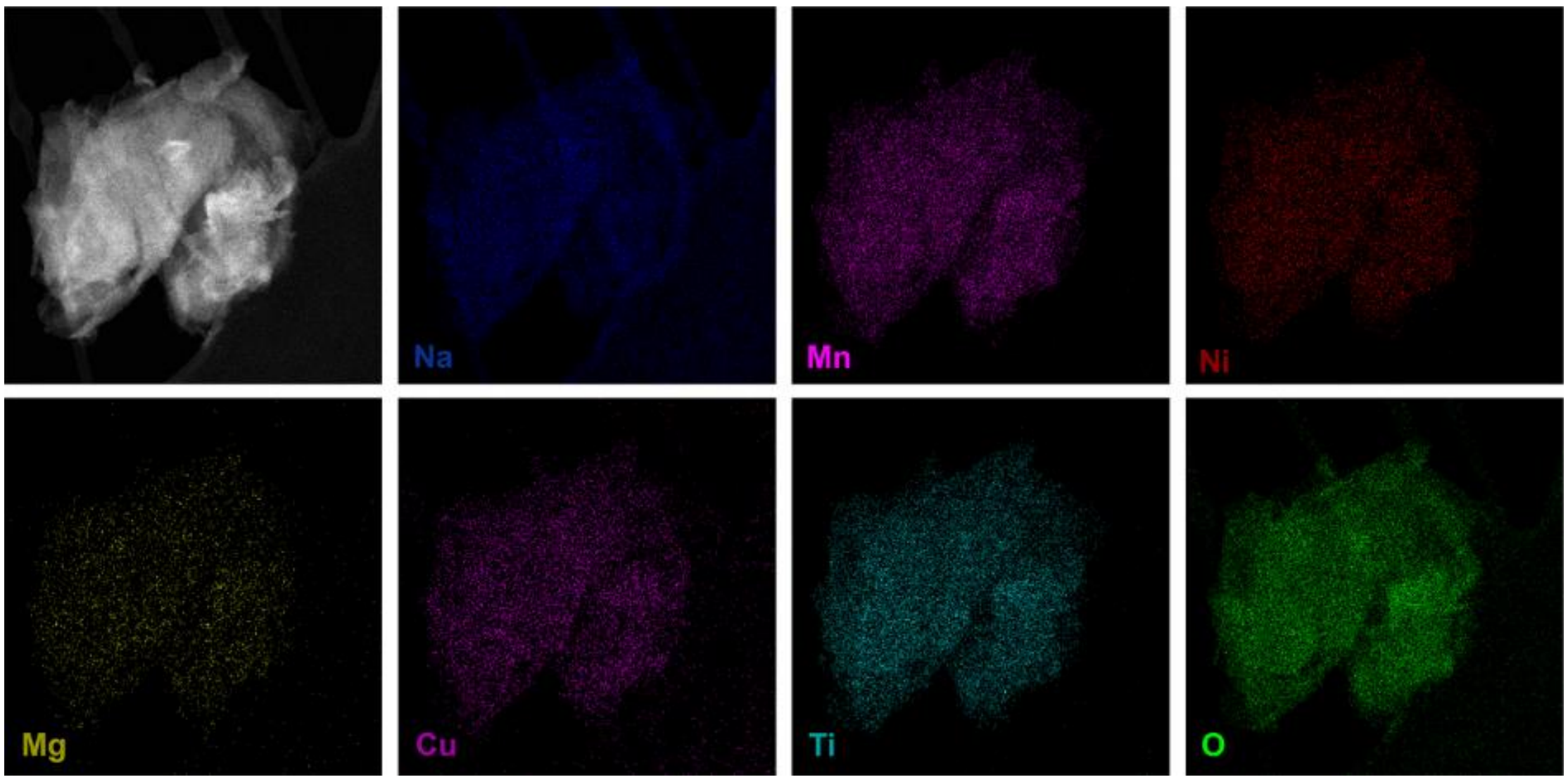

Supplementary Figure S7 TEM-mapping images of NLCMMTO.

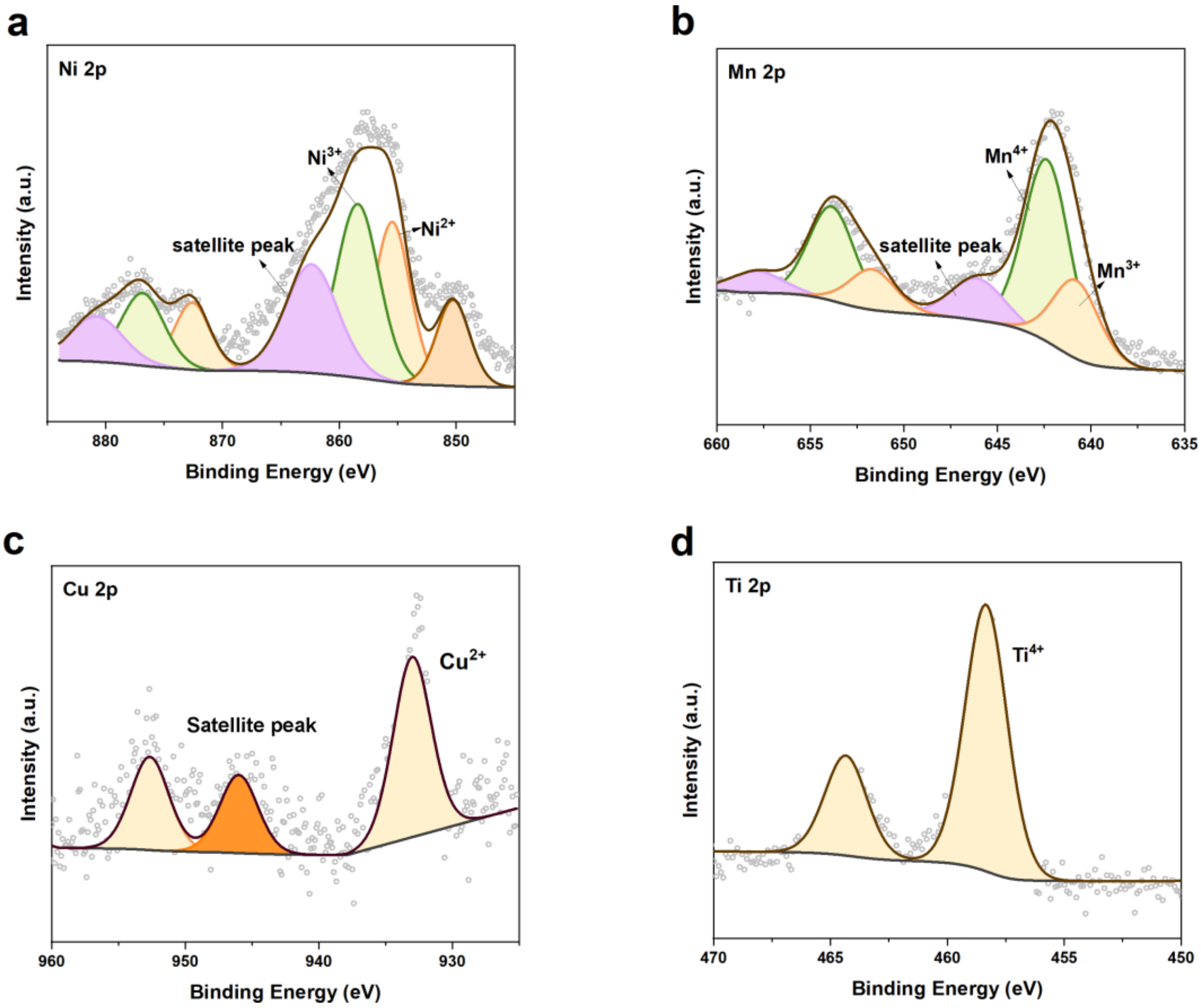

Supplementary Figure S8 XPS spectra of NLCMMTO. The fine spectra of a) Ni, b) Mn, c) Cu, and d) Ti.

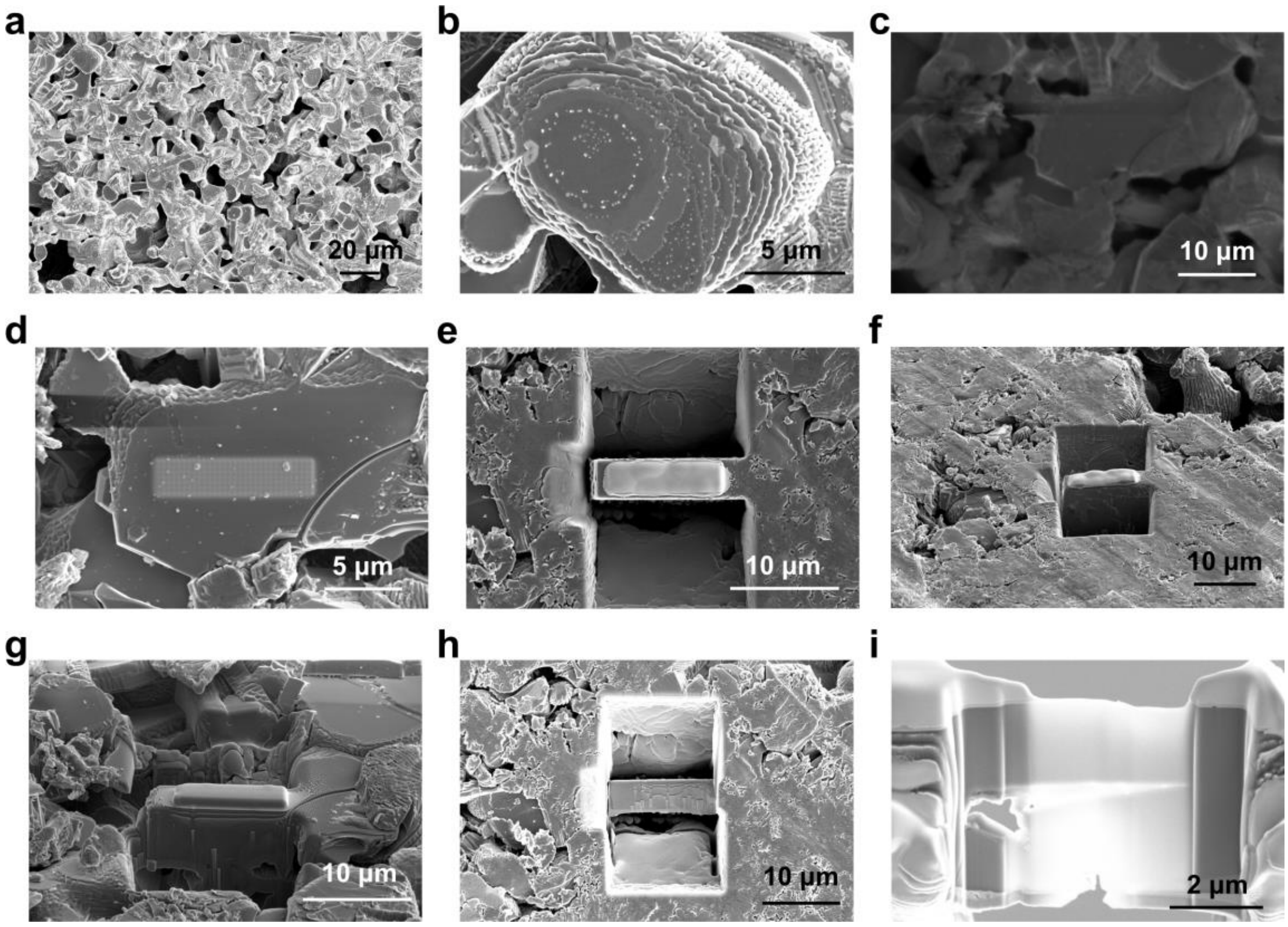

Supplementary Figure S9 The FIB-STEM sample preparation process. a) Low-magnification SEM image of NLCMMTO particles. b) Enlarged SEM image of the selected NLCMMTO particle with a relatively flat surface for FIB preparation. c) Electron-beam-induced deposition of a W protective layer on the selected region. d) Ion-beam-induced deposition of an additional W protective layer on the same region. e) Ga-ion-beam milling of trenches on both sides of the W-protected region. f) U-cut of the target region after trench milling. g) Side-view SEM image of the partially isolated lamella after U-cutting. h) Top-view SEM image of the isolated lamella prior to lift-out. i) Final thinning and polishing of the lamella to obtain an electron-transparent FIB-STEM specimen.

In the SEM window, a relatively flat surface area was identified. An electron-beam-induced deposition was then performed to deposit a W metal protective layer with dimensions of 10 μm in length, 2 μm in width, and 0.2 μm in height on the surface. Subsequently, an ion-beam-induced deposition was utilized to cover the same area with an additional 2 μm thick W protective layer. Next, Ga ion beams were employed to etch pits on both the upper and lower sides of the target region, followed by a U-cut. The target lamella was then extracted using a micromanipulator and welded onto a FIB-dedicated grid. Finally, the lamella was carefully thinned and polished using an ion beam to obtain a FIB-STEM sample that is amorphous-free and electron-beam transparent. The sample preparation process is shown in Figure S9

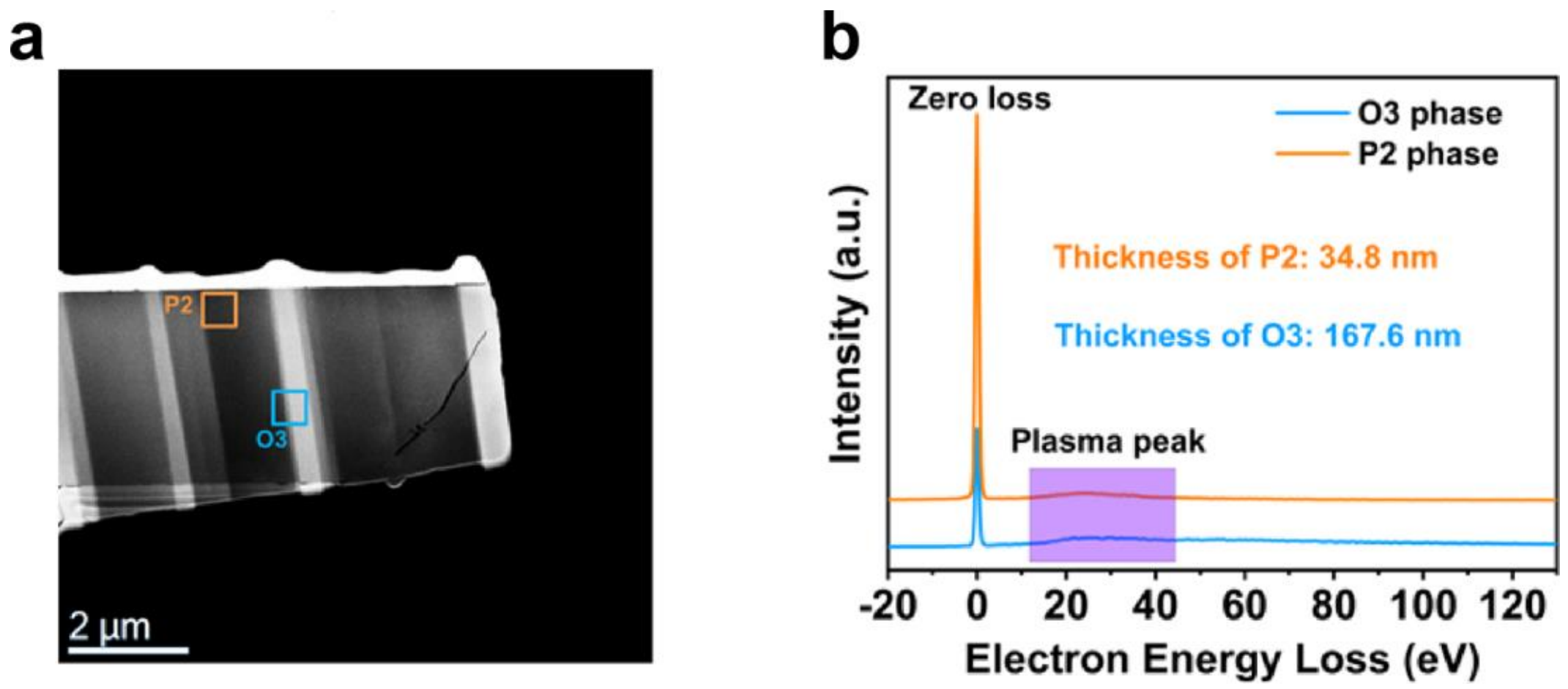

Supplementary Figure S10 a) Cross-sectional TEM image of NLCMMTO. b) Electron energy loss spectroscopy (EELS) spectra collected from the P2 and O3 regions.

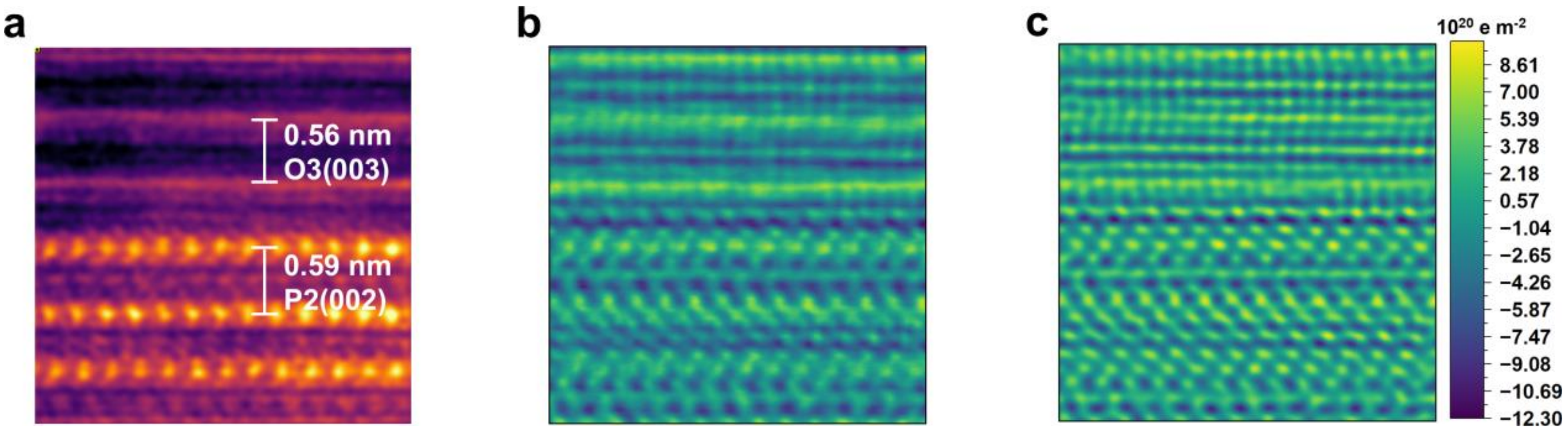

Supplementary Figure S11 a) BF-contrast inversion, b) phase-contrast image and c) calculated charge density of P2 and O3 intergrowth area in NLCMMTO.

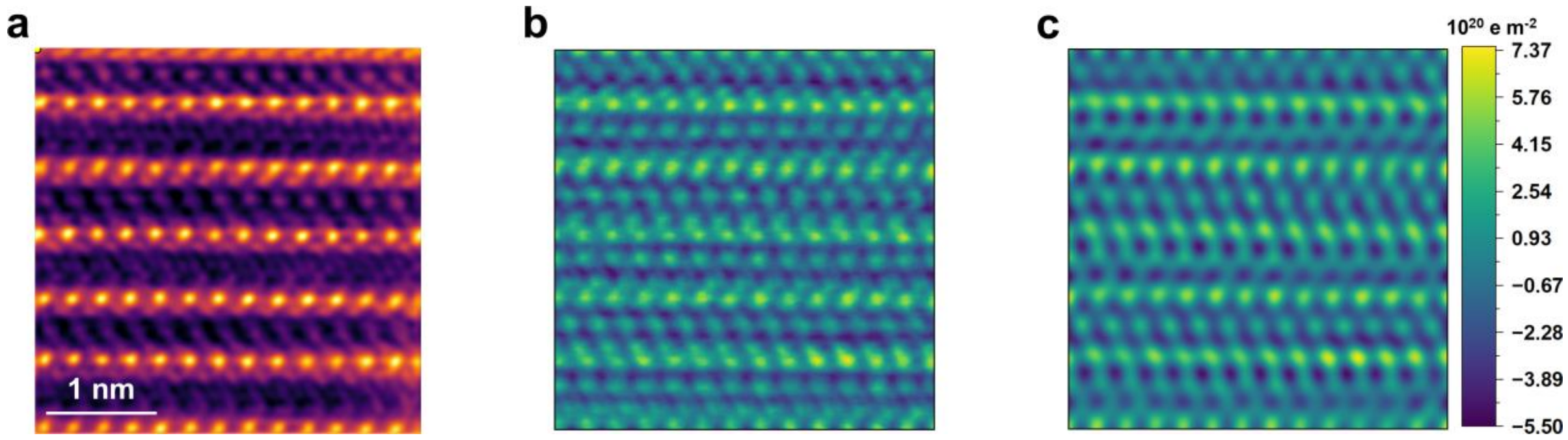

Supplementary Figure S12 a) BF-contrast inversion, b) phase-contrast image and c) calculated charge density of P2 area in NLCMMTO.

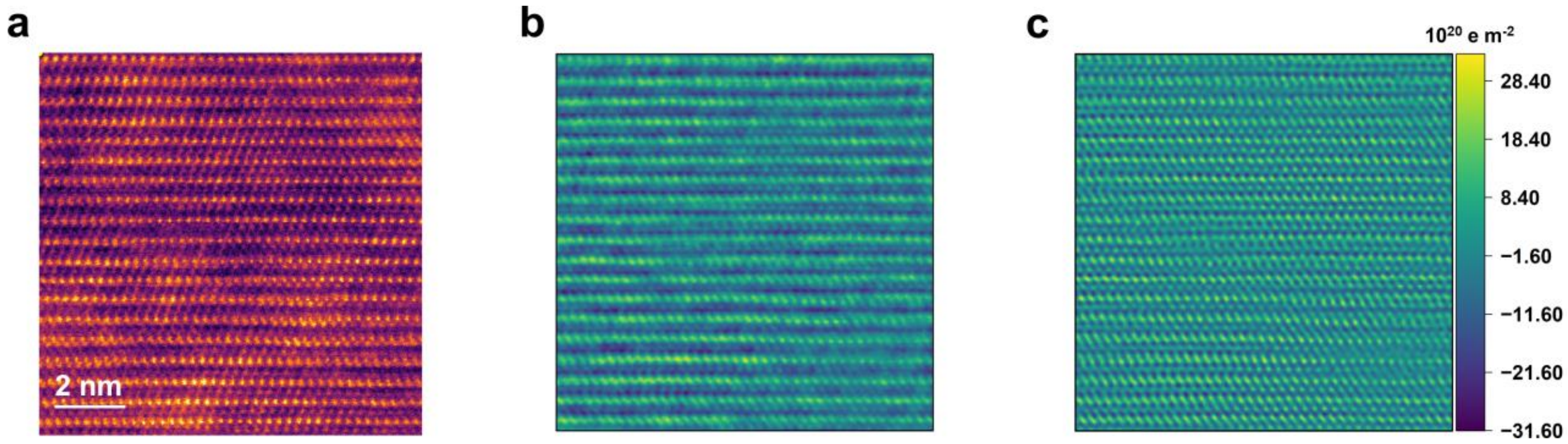

Supplementary Figure S13 a) BF-contrast inversion, b) phase-contrast image and c) calculated charge density of O3 area in NLCMMTO.

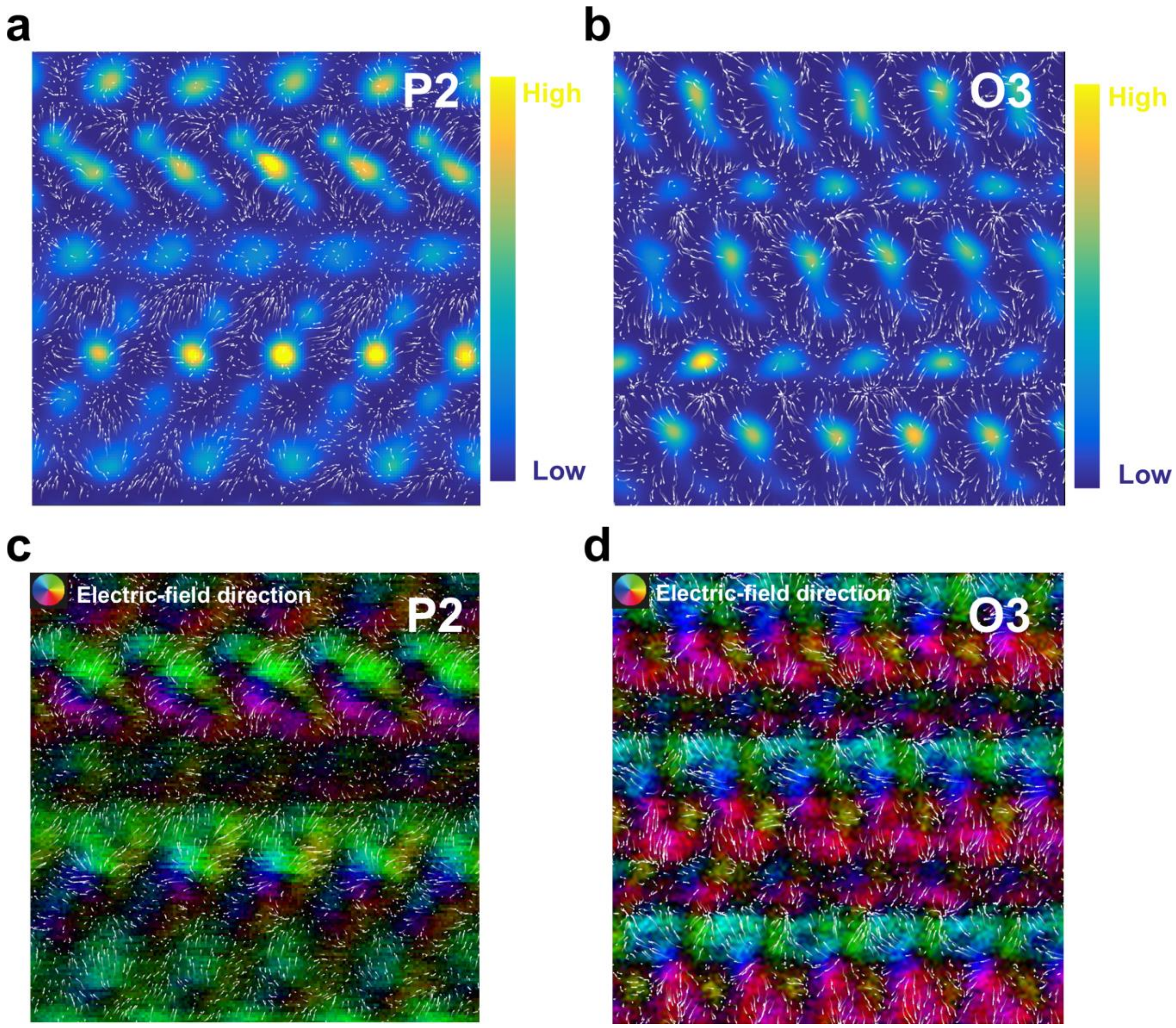

Supplementary Figure S14 a, b) Electrostatic potential distributions in the P2 and O3 regions of NLCMMTO, directly generated using Novena software, c, d) electric field distributions in P2 and O3 region in NLCMMTO.

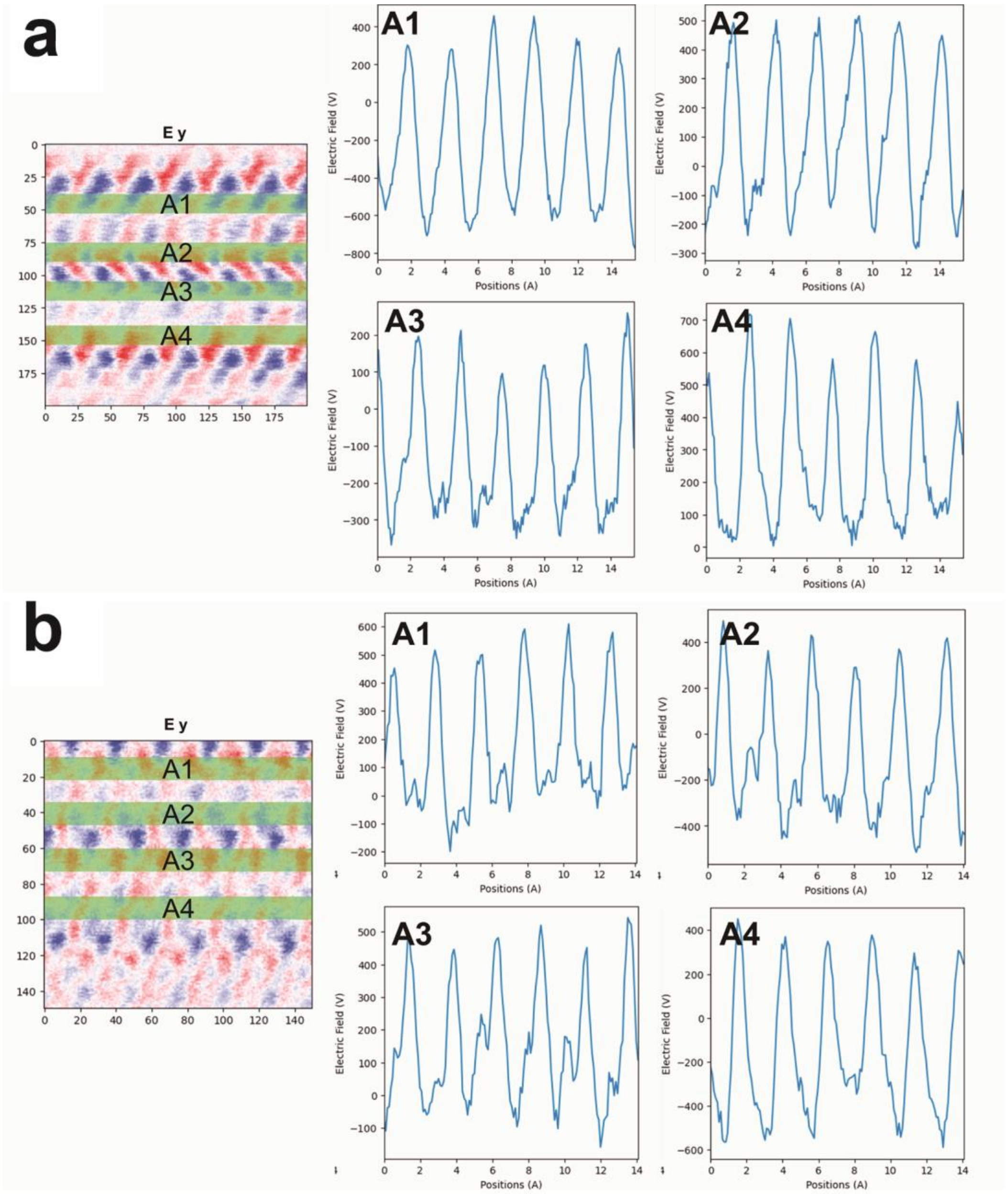

Supplementary Figure S15 a) The *y*-component of the electric field strength for the P2 phase and the integral of the electric field strength over four oxygen atom layers. b) The *y*-component of the electric field strength for the O3 phase and the integral of the electric field strength over four oxygen atom layers.

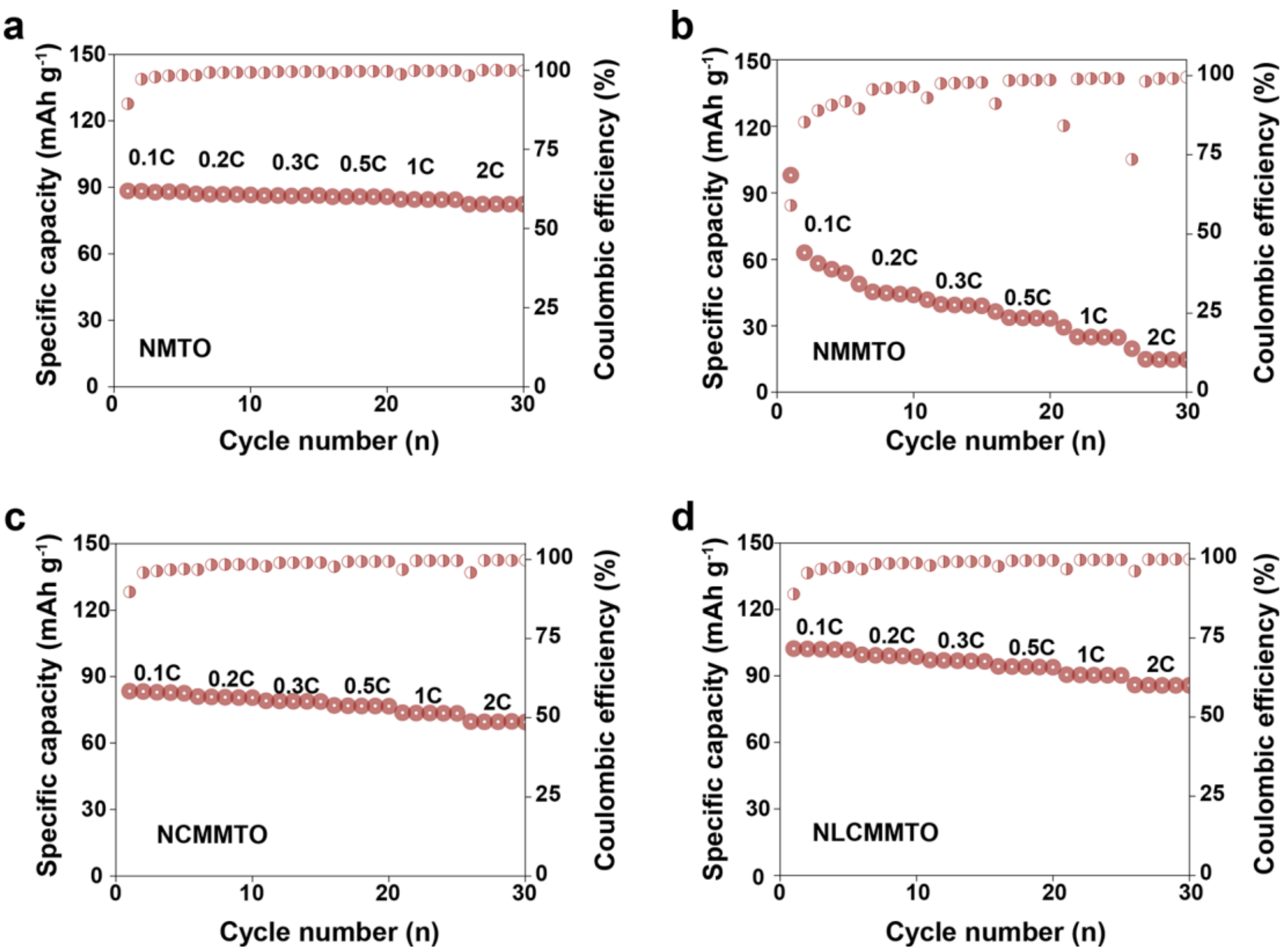

Supplementary Figure S16 The rate performance of a) NMTO, b) NMMTO, c) NCMMTO, and d) NLCMMTO at room temperature (25 ± 2°C) (1 C=120 mA g^{-1}).

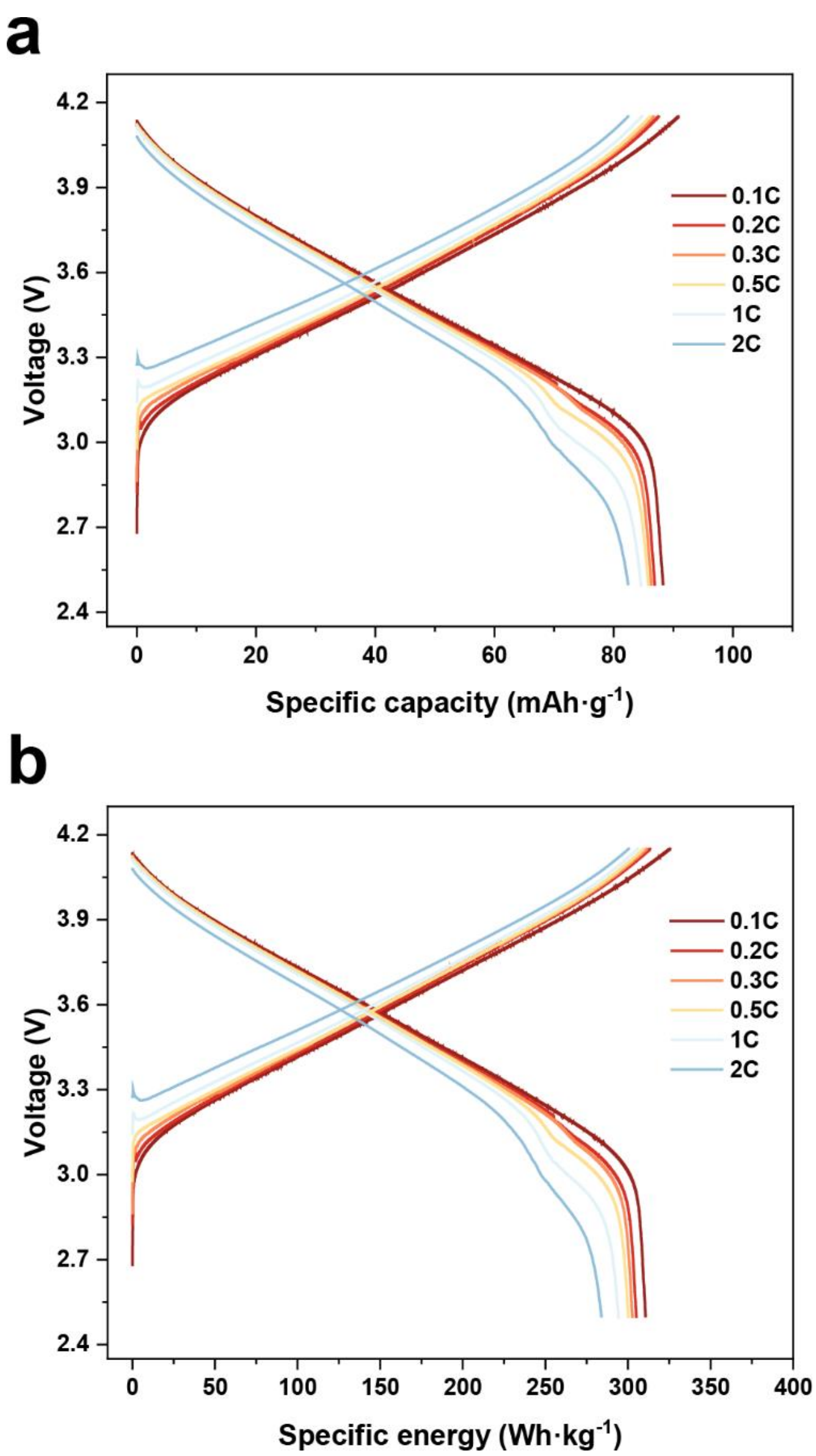

Supplementary Figure S17 NMTO a) Specific capacity and b) specific energy charge/discharge curves at different rates in 2.5 - 4.15 V and room temperature (25 ± 2 °C). (1 C = 120 mA g^{-1})

Figure S17b presents the specific energy in the second cycle at each rate, calculated based on the mass of the cathode active material (2.101 mg).

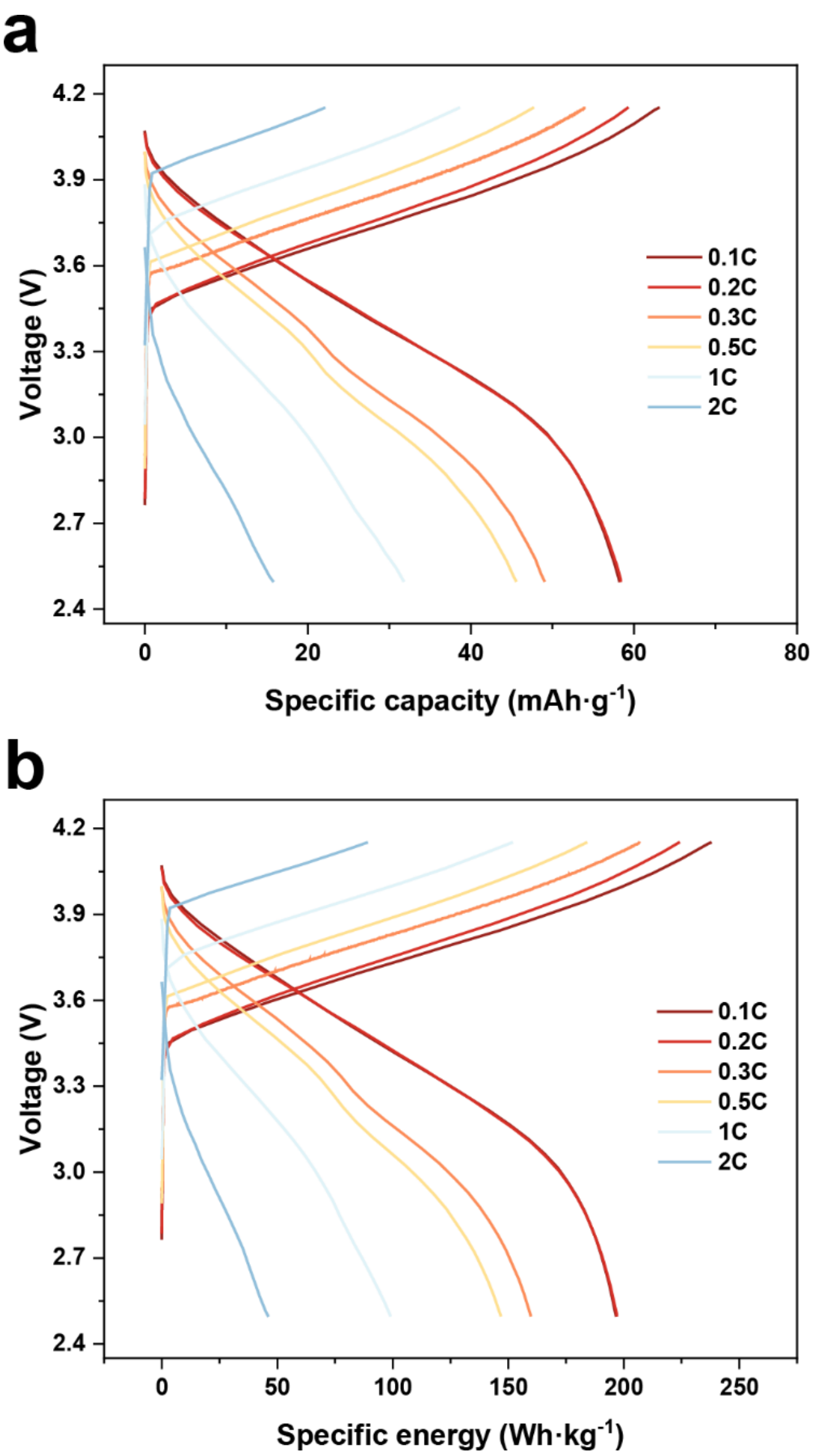

Supplementary Figure S18 NMMTO a) Specific capacity and b) specific energy charge/discharge curves at different rates in 2.5 - 4.15 V and room temperature (25 ± 2 °C). (1 C = 120 mA g^{-1})

Figure S18b presents the specific energy in the second cycle at each rate, calculated based on the mass of the cathode active material (1.051 mg).

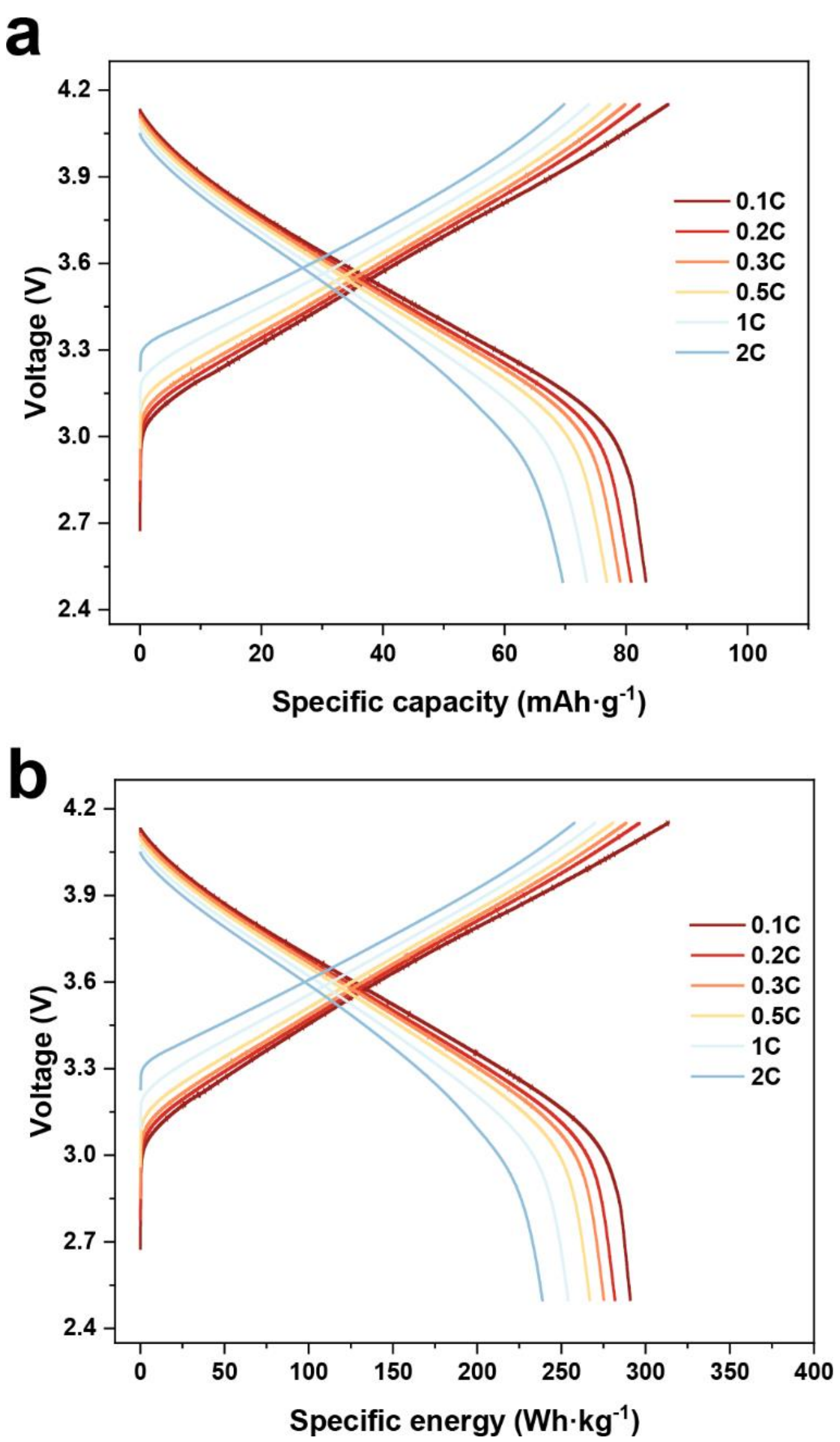

Supplementary Figure S19 NCMMTO a) Specific capacity and b) specific energy charge/discharge curves at different rates in 2.5 - 4.15 V and room temperature (25 ± 2°C). (1 C = 120 mA g^{-1})

Figure S19b presents the specific energy in the second cycle at each rate, calculated based on the mass of the cathode active material (1.128 mg).

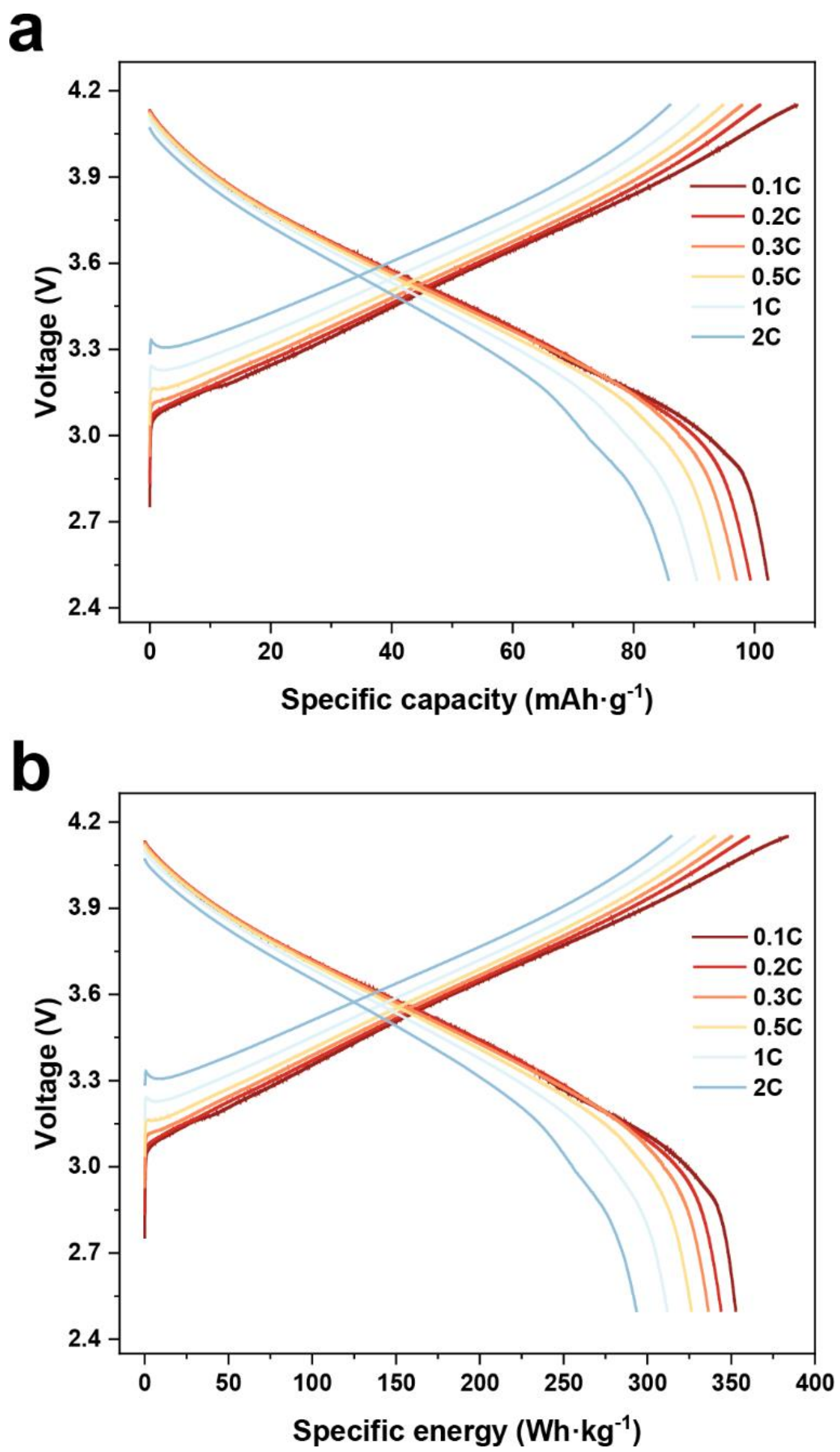

Supplementary Figure S20 NLCMMTO a) Specific capacity and b) specific energy charge/discharge curves at different rates in 2.5 - 4.15 V and room temperature (25 ± 2°C). (1 C = 120 mA g^{-1})

Figure S20b presents the specific energy in the second cycle at each rate, calculated based on the mass of the cathode active material (1.527mg).

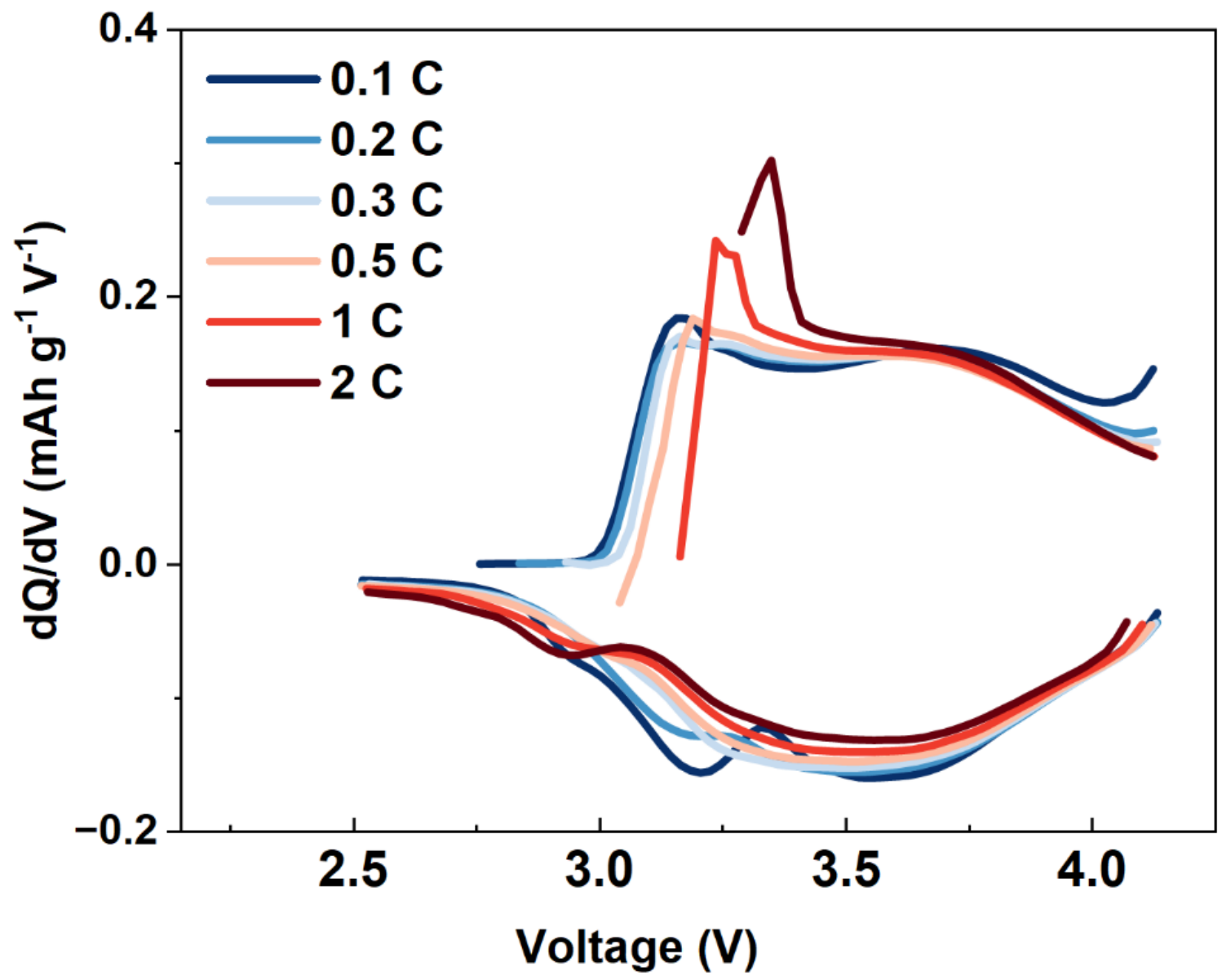

Supplementary Figure S21 dQ/dV curves of NLCMMTO in 2.5 - 4.15 V voltage regions at different rates and room temperature (25 ± 2°C). (1 C = 120 mA g^{-1})

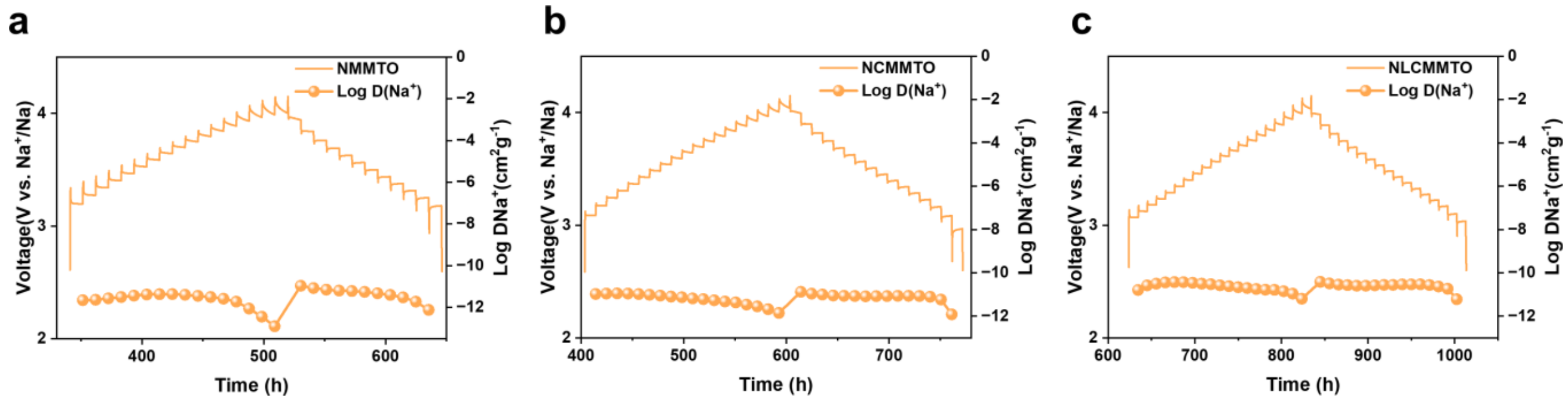

Supplementary Figure S22 GITT curves of a) NMMTO, b) NCMMTO and c) NLCMMTO within 2.5 - 4.15 V at 10 mA g^{-1} current and room temperature (25°C ± 2°C).

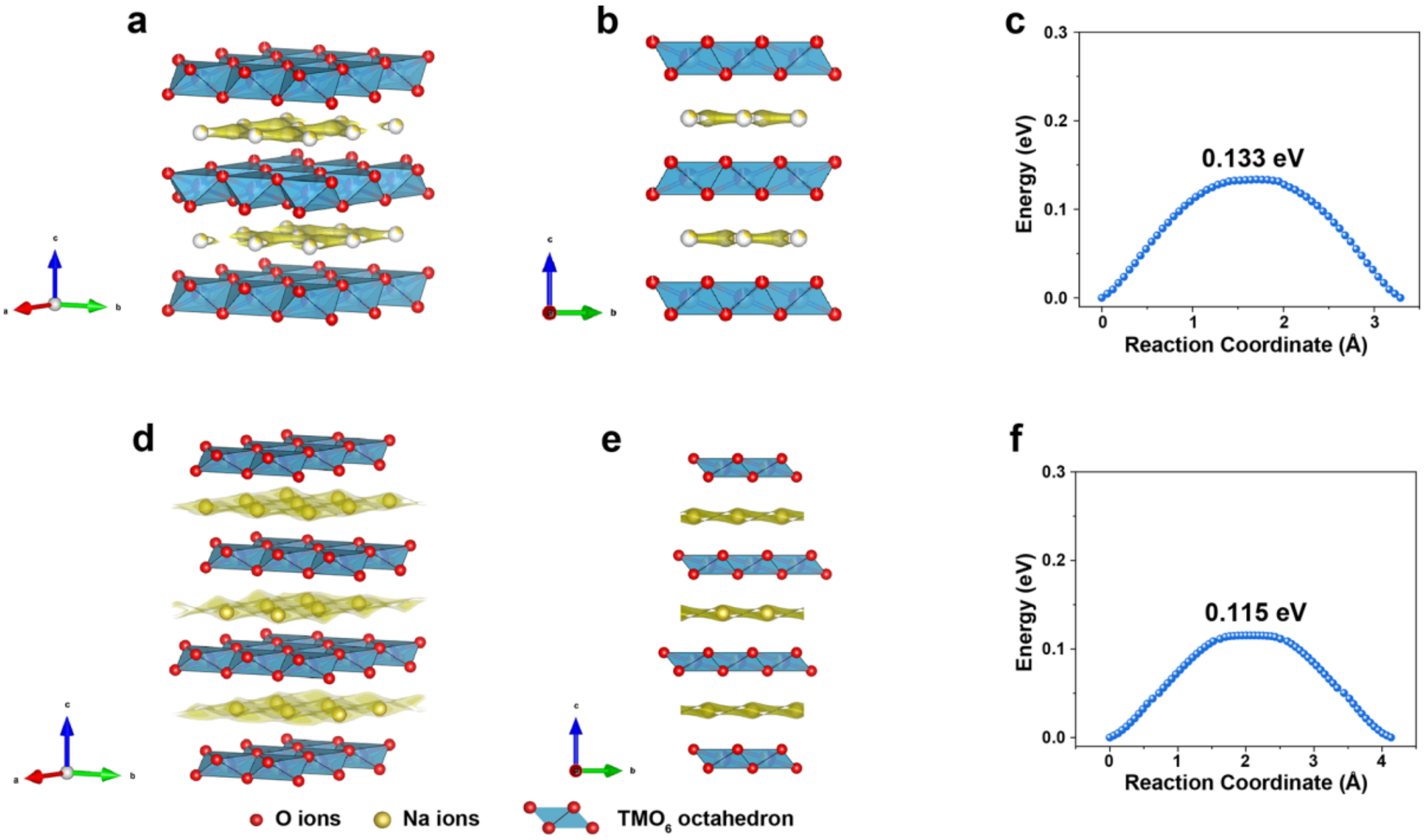

Supplementary Figure S23 Ion migration paths and energy barrier diagrams of a-c) NLCMMTO-P2 and d-f) NLCMMTO-O3 simulated by the BVSE method (the yellow projection is the diffusion path of sodium ions)

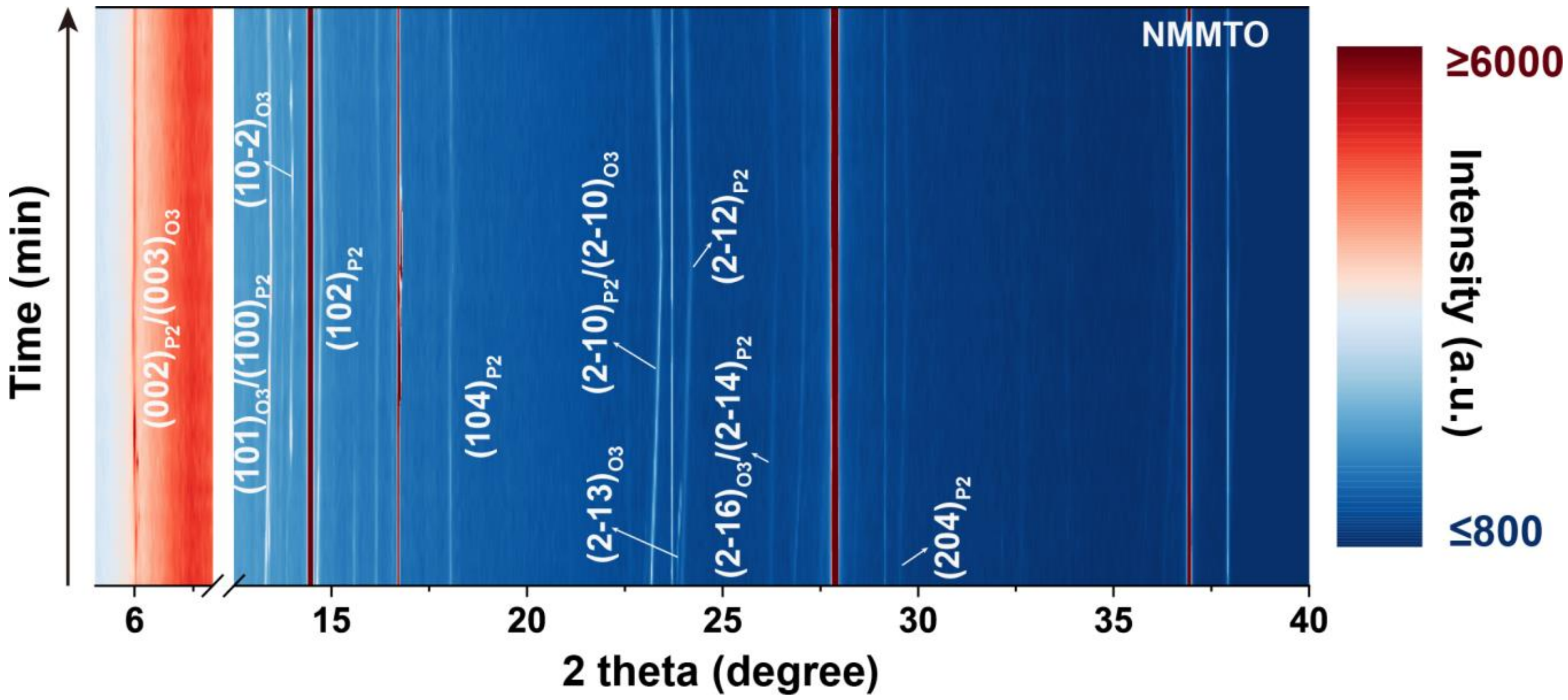

Supplementary Figure S24 Intensity contour map (bird's eye view) of the NMMTO during the first charge/discharge at 20 mA g^{-1} in the voltage range of 2.5 - 4.15 V at room temperature (25 ± 2°C).

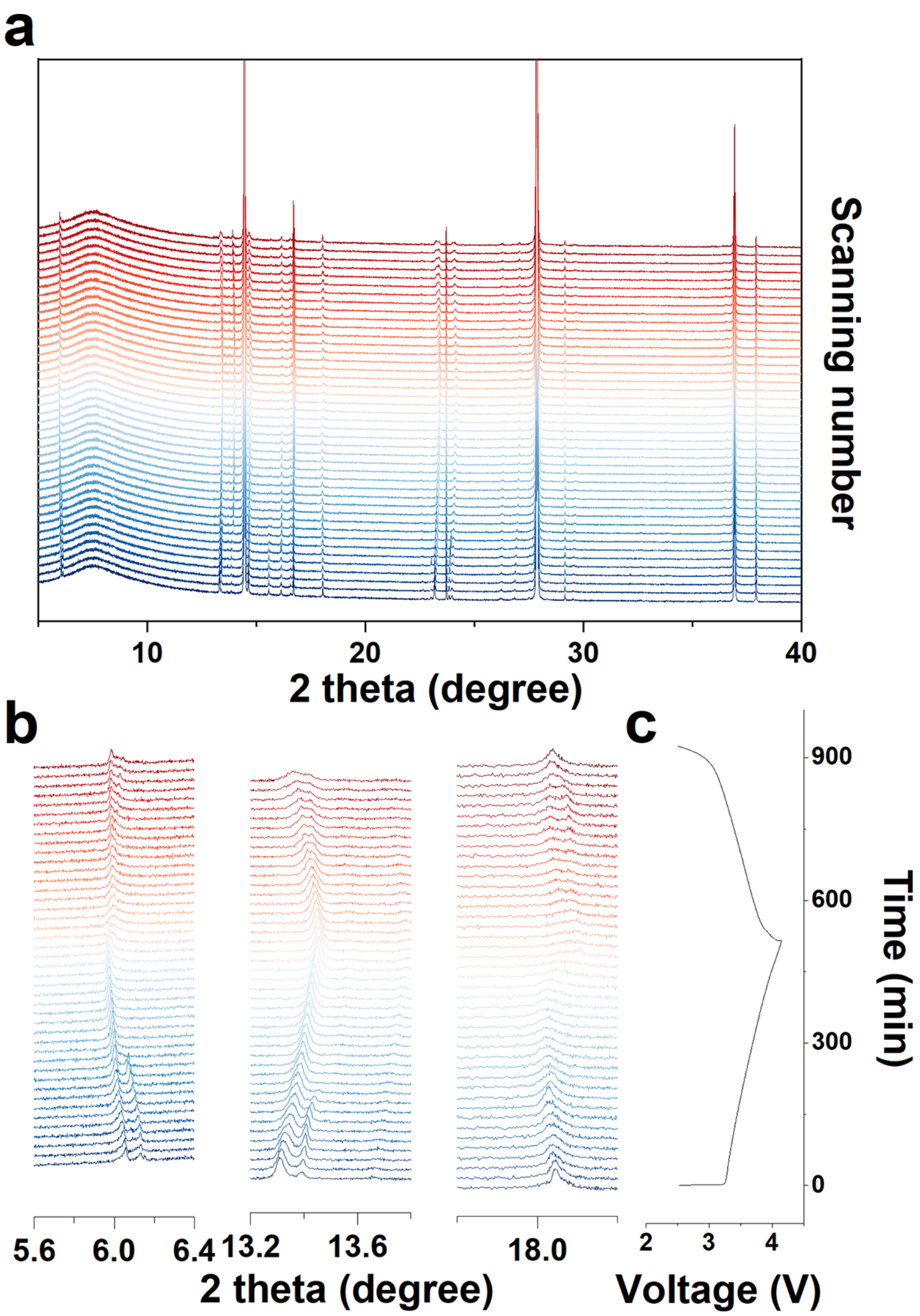

Supplementary Figure S25 a) In situ synchrotron-based XRD pattern of NMMTO during the first charge/discharge at 20 mA g^{-1} in the voltage range of 2.5-4.15 V. b) The selected area in Figure S25a. c) Profile of charging/discharging of NMMTO at 20 mA g^{-1} and room temperature (25 ± 2°C).

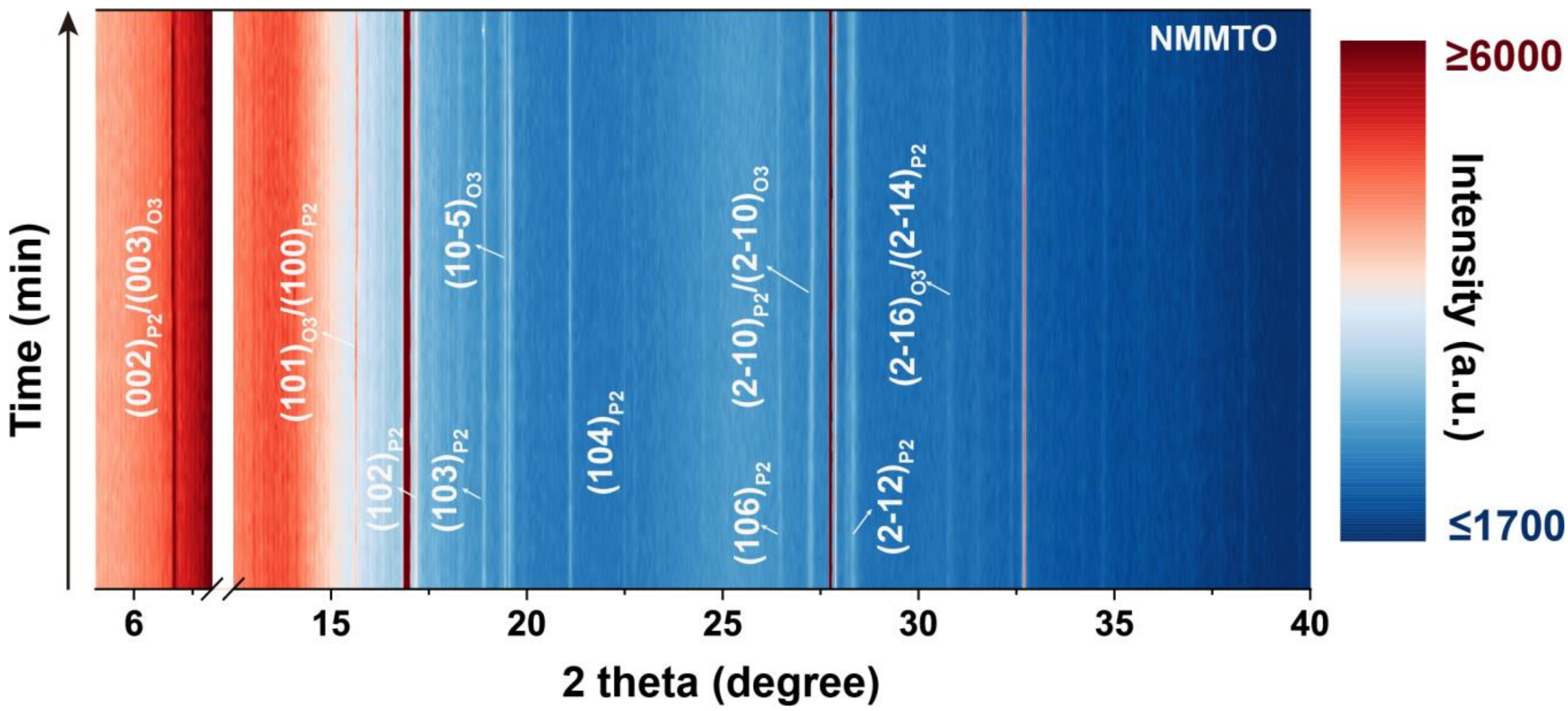

Supplementary Figure S26 Intensity contour map (bird's eye view) of the NMMTO during the second charge/discharge at 120 mA g^{-1} in the voltage range of 2.5 - 4.15 V at room temperature (25 ± 2°C).

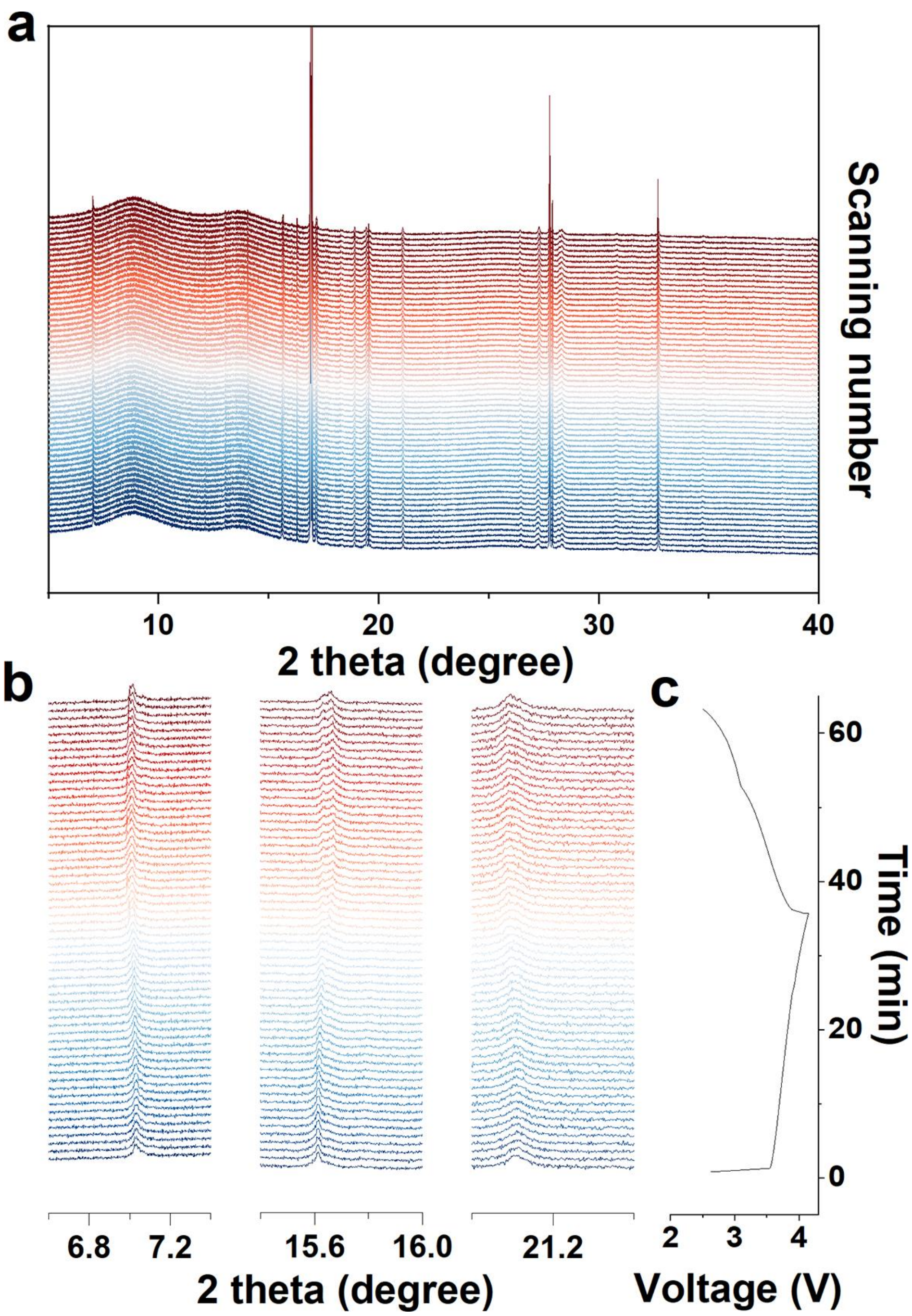

Supplementary Figure S27 a) In-situ synchrotron-based XRD pattern of NMMTO during the second charge/discharge at 120 mA g^{-1} in the voltage range of 2.5 - 4.15 V. b) The selected area in Figure S27a. c) Profile of charging/discharging of NMMTO at 120 mA g^{-1} and room temperature (25 ± 2°C).

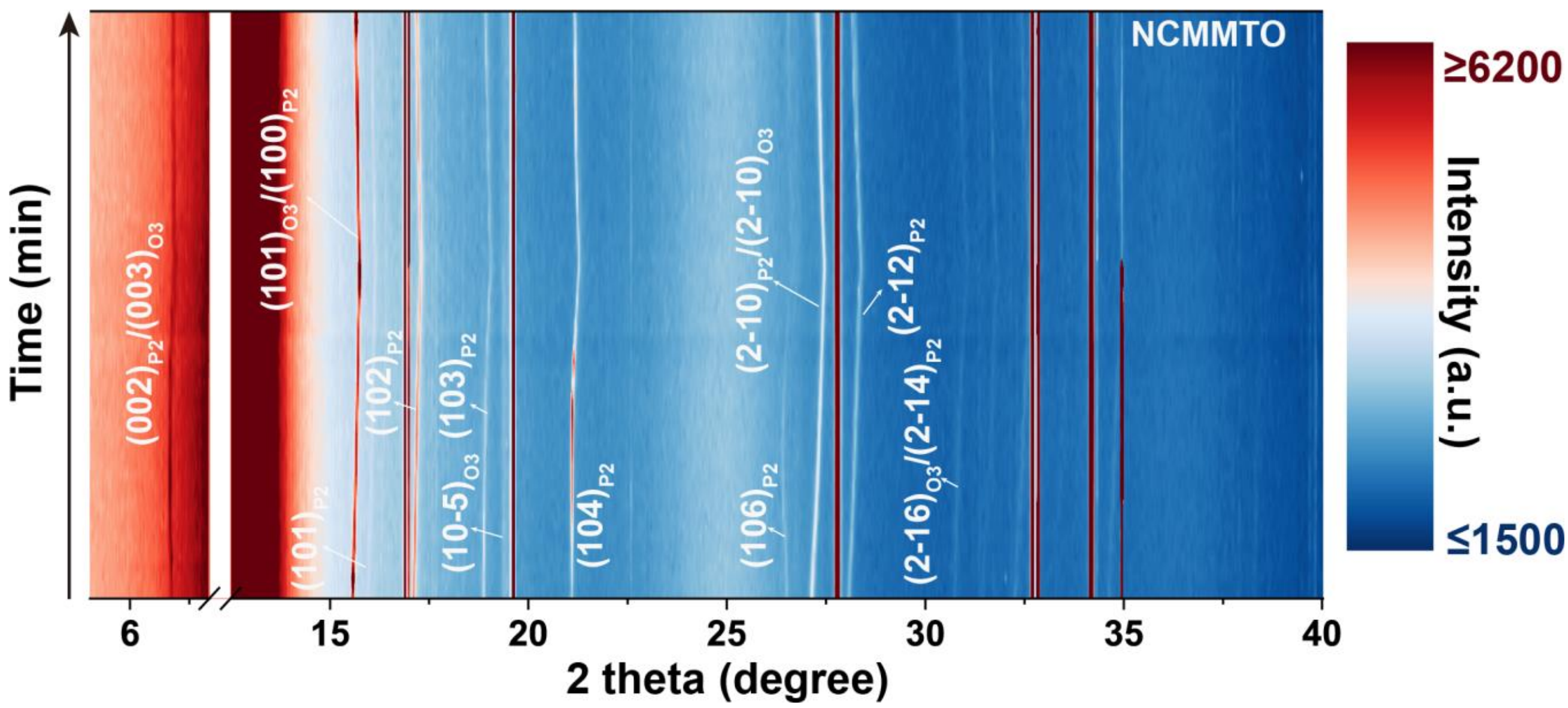

Supplementary Figure S28 Intensity contour map (bird's eye view) of the NCMMTO during the first charge/discharge at 20 mA g^{-1} in the voltage range of 2.5 - 4.15 V at room temperature (25 ± 2°C).

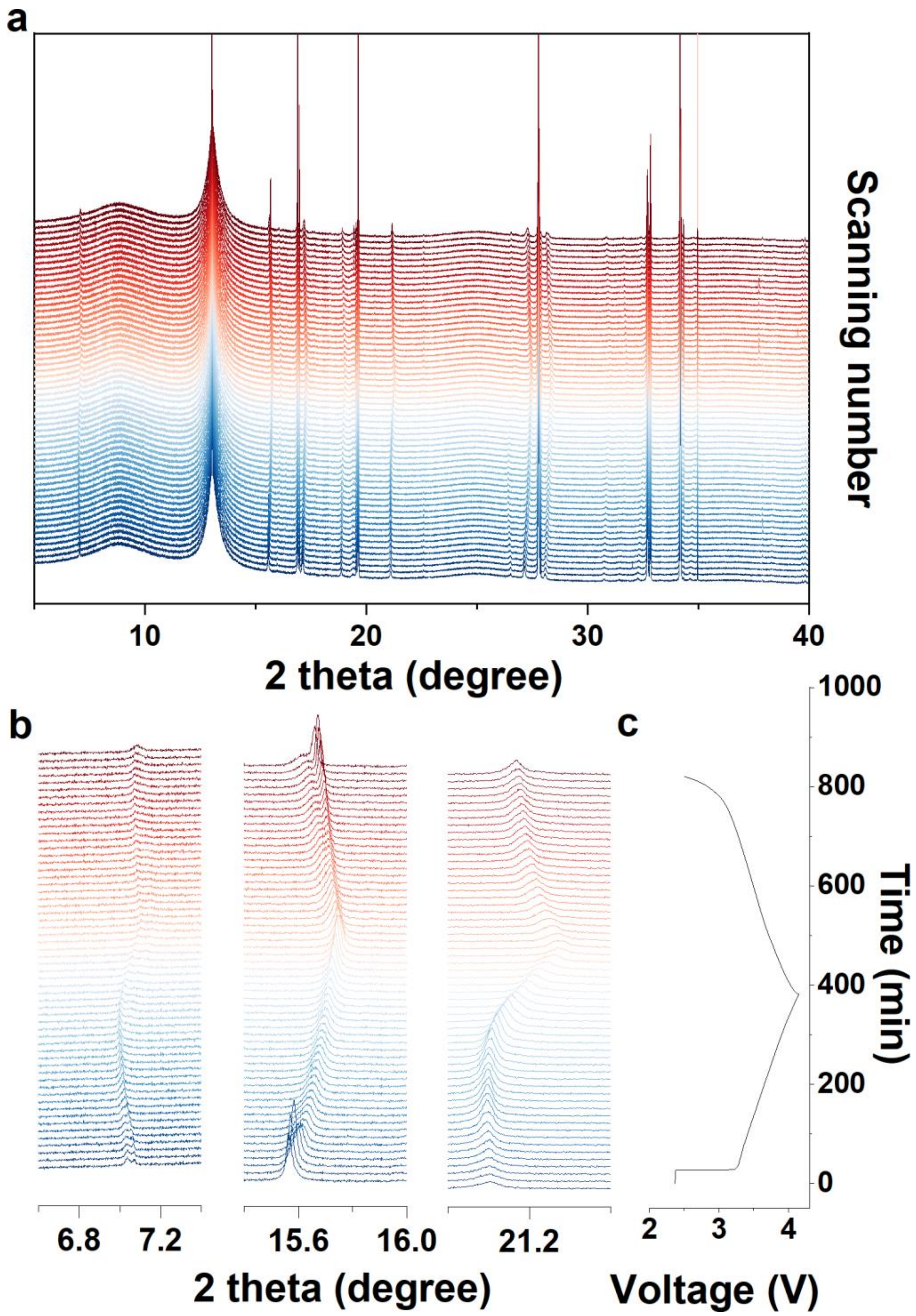

Supplementary Figure S29 a) In situ synchrotron-based XRD pattern of NCMMTO during the first charge/discharge at 20 mA g^{-1} in the voltage range of 2.5 - 4.15 V. b) The selected area in Figure S29a. c) Profile of charging/discharging of NCMMTO at 20 mA g^{-1} and room temperature (25 ± 2°C).

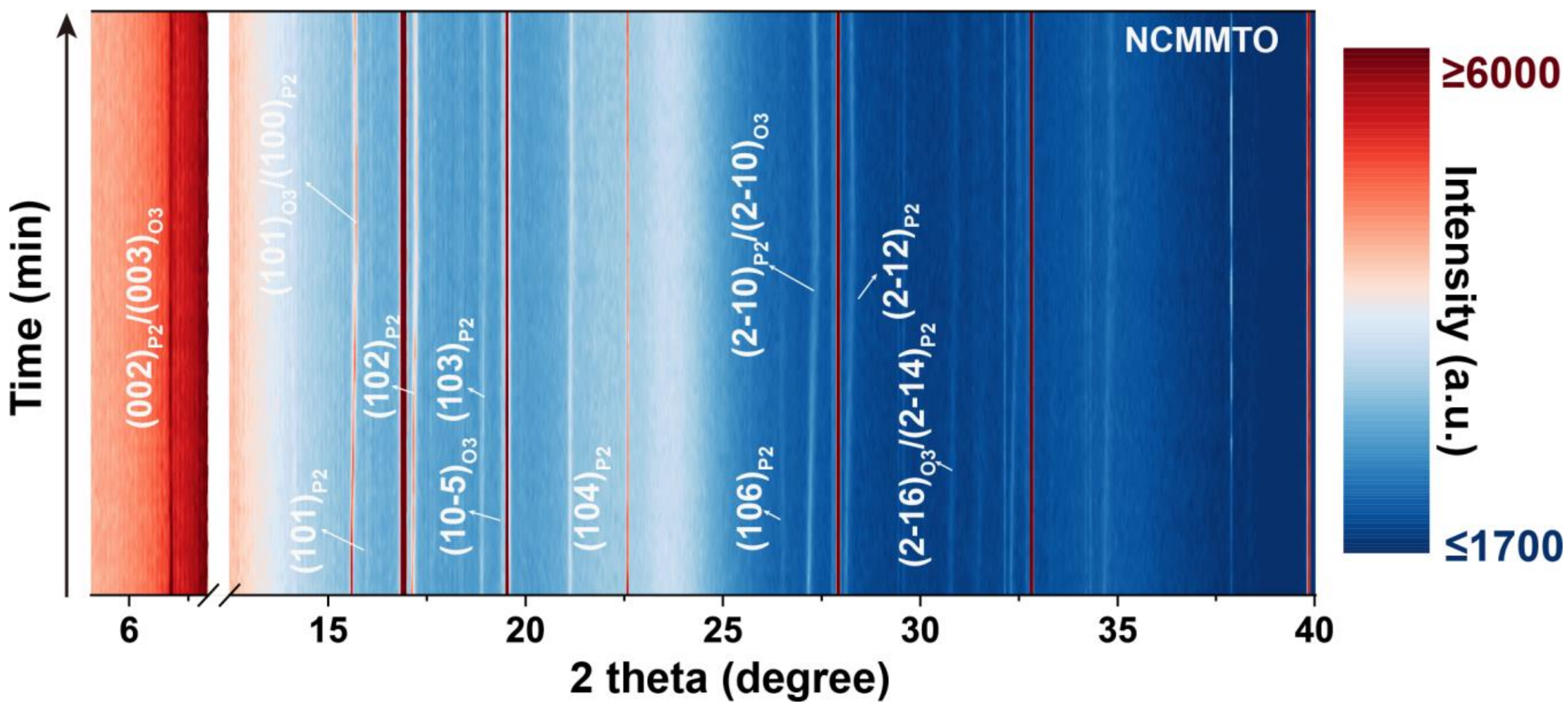

Supplementary Figure S30 Intensity contour map (bird's eye view) of the NCMMTO during the second charge/discharge at 120 mA g^{-1} in the voltage range of 2.5 - 4.15 V at room temperature (25 ± 2°C).

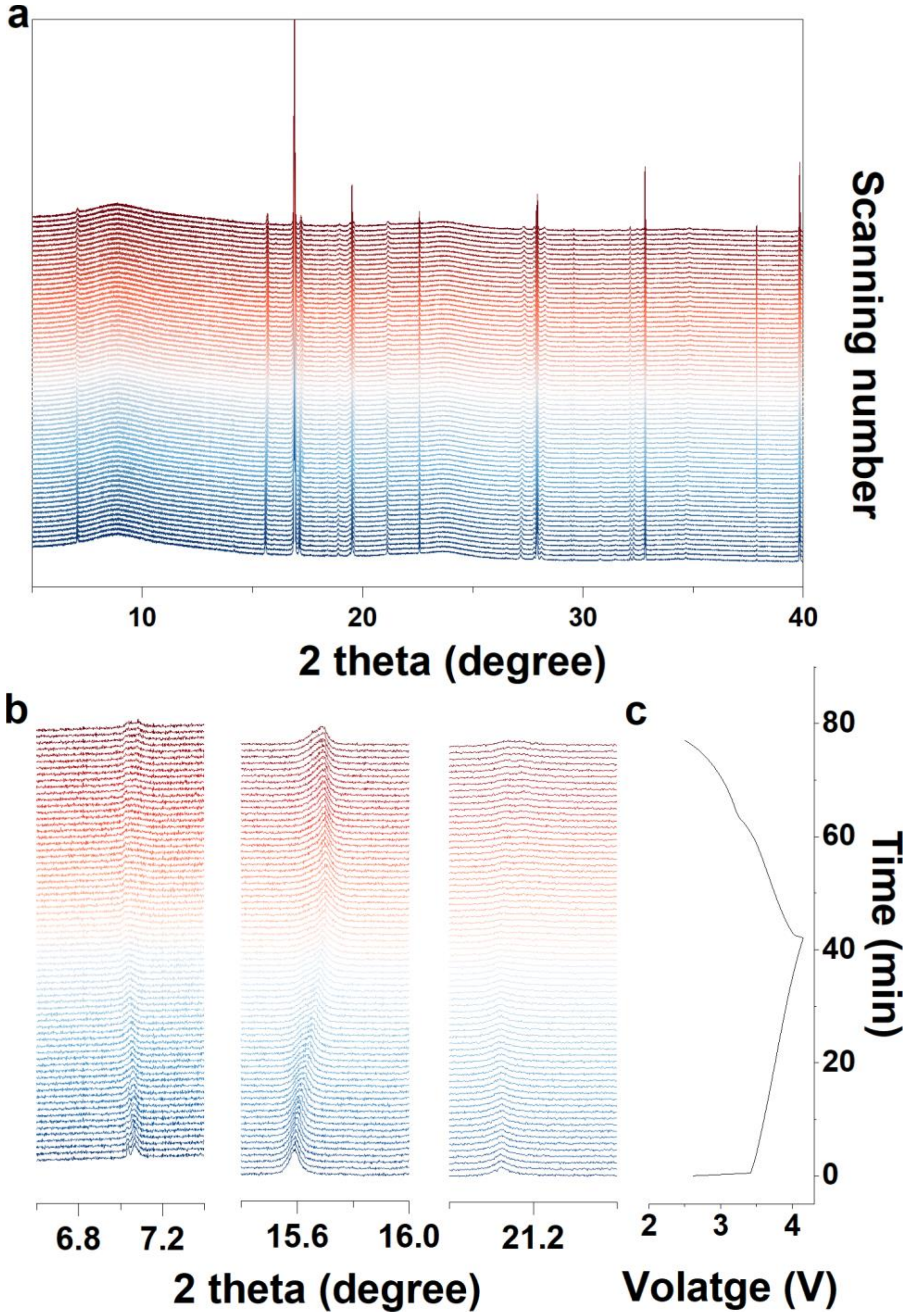

Supplementary Figure S31 a) In-situ synchrotron-based XRD pattern of NCMMTO during the second charge/discharge at 120 mA g^{-1} in the voltage range of 2.5-4.15 V. b) The selected area in Figure S31a. c) Profile of charging/discharging of NCMMTO at 120 mA g^{-1} and room temperature (25 ± 2°C).

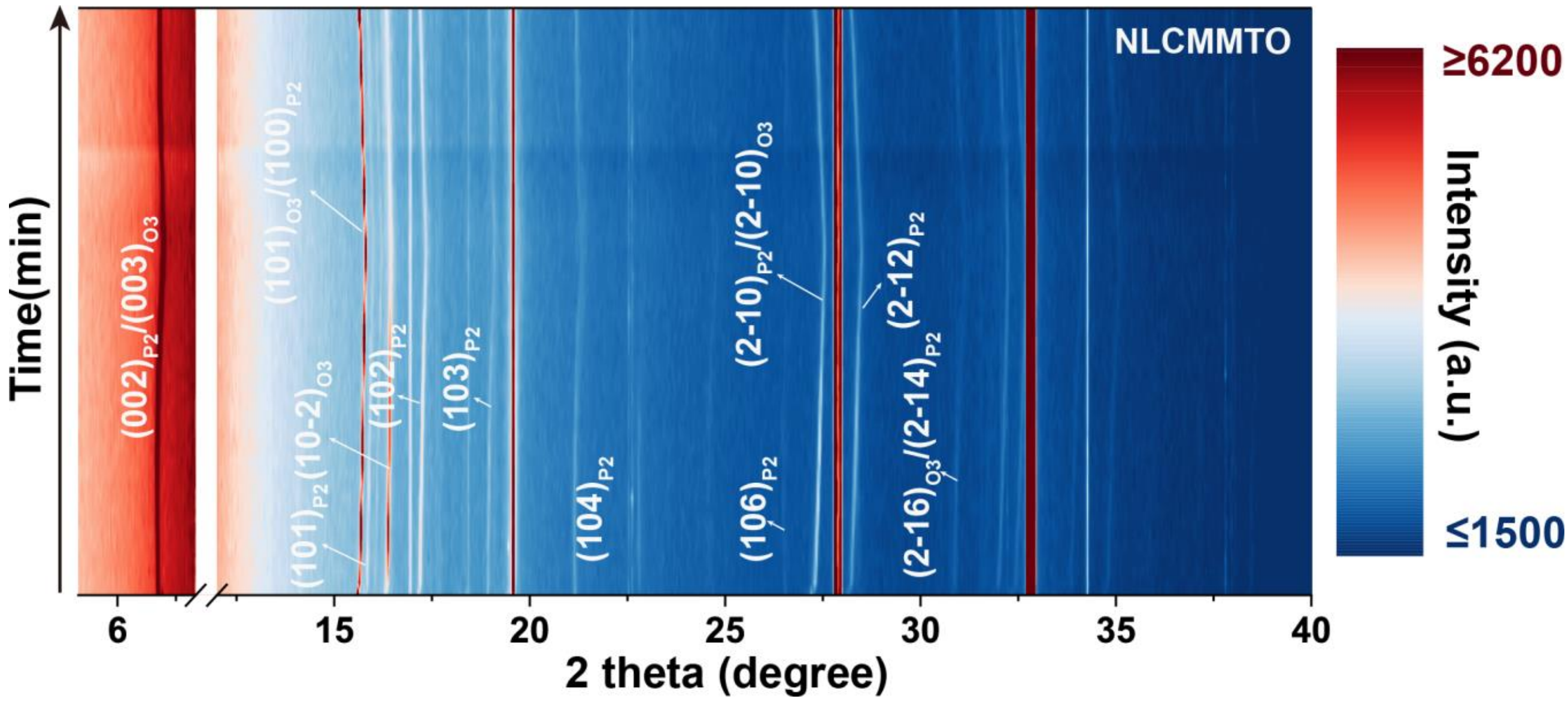

Supplementary Figure S32 Intensity contour map (bird's eye view) of the NLCMMTO during the first charge/discharge at 20 mA g^{-1} in the voltage range of 2.5-4.15 V at room temperature (25 ± 2°C).

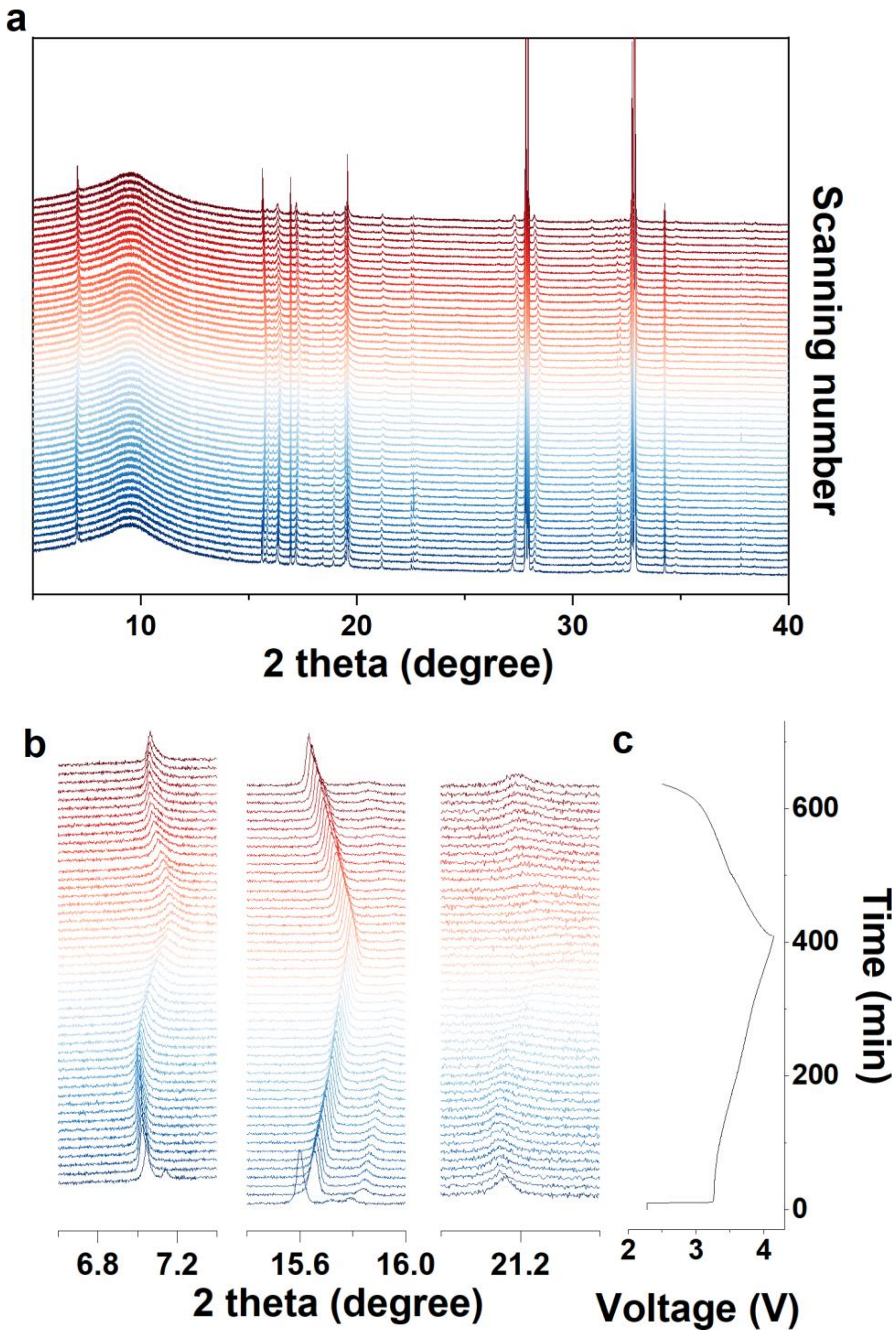

Supplementary Figure S33 a) In-situ synchrotron-based XRD pattern of NLCMMTO during the first charge/discharge at 20 mA g^{-1} in the voltage range of 2.5-4.15 V. b) The selected area in Figure S33a. c) Profile of charging/discharging of NLCMMTO at 20 mA g^{-1} and room temperature (25 ± 2°C).

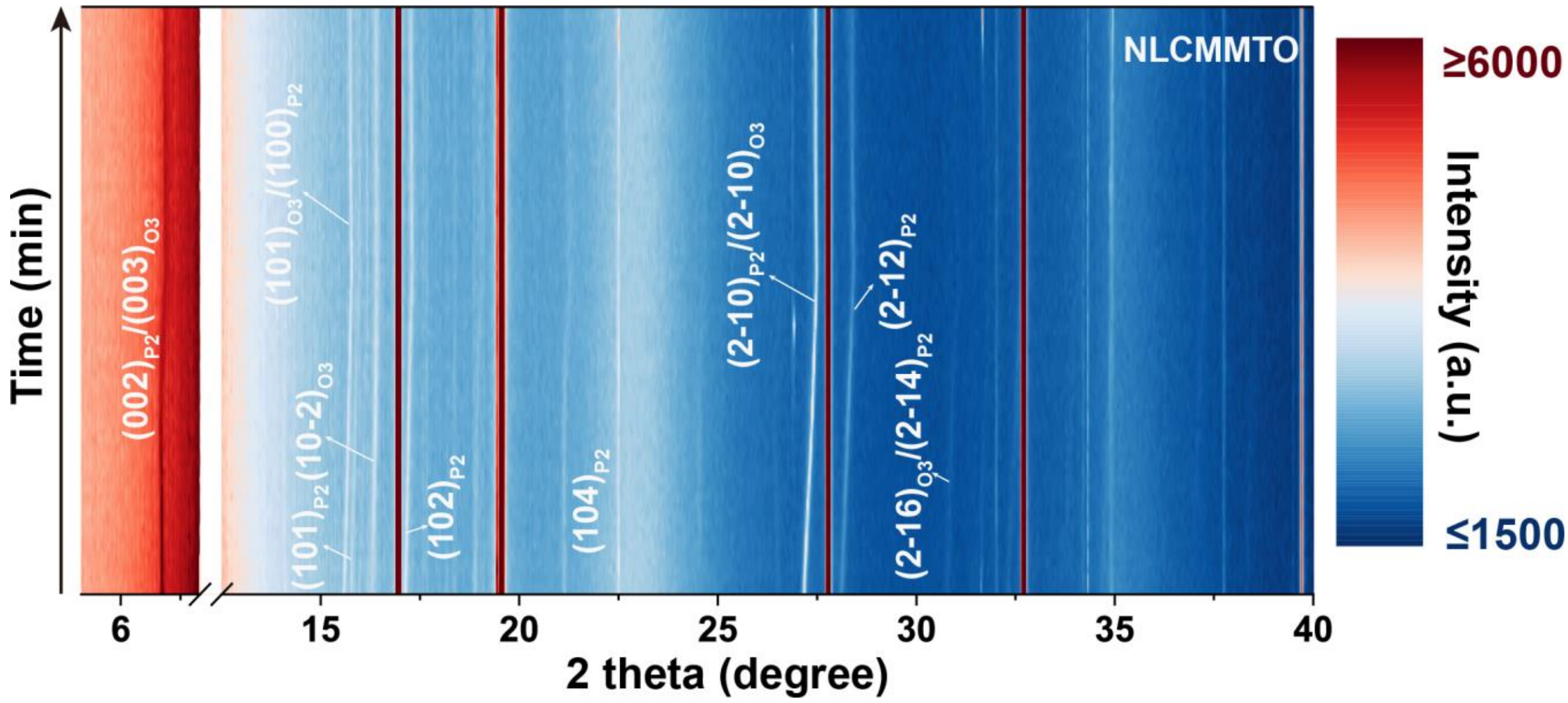

Supplementary Figure S34 Intensity contour map (bird's eye view) of the NLCMMTO during the second charge/discharge at 120 mA g^{-1} in the voltage range of 2.5- 4.15 V at room temperature (25 ± 2°C).

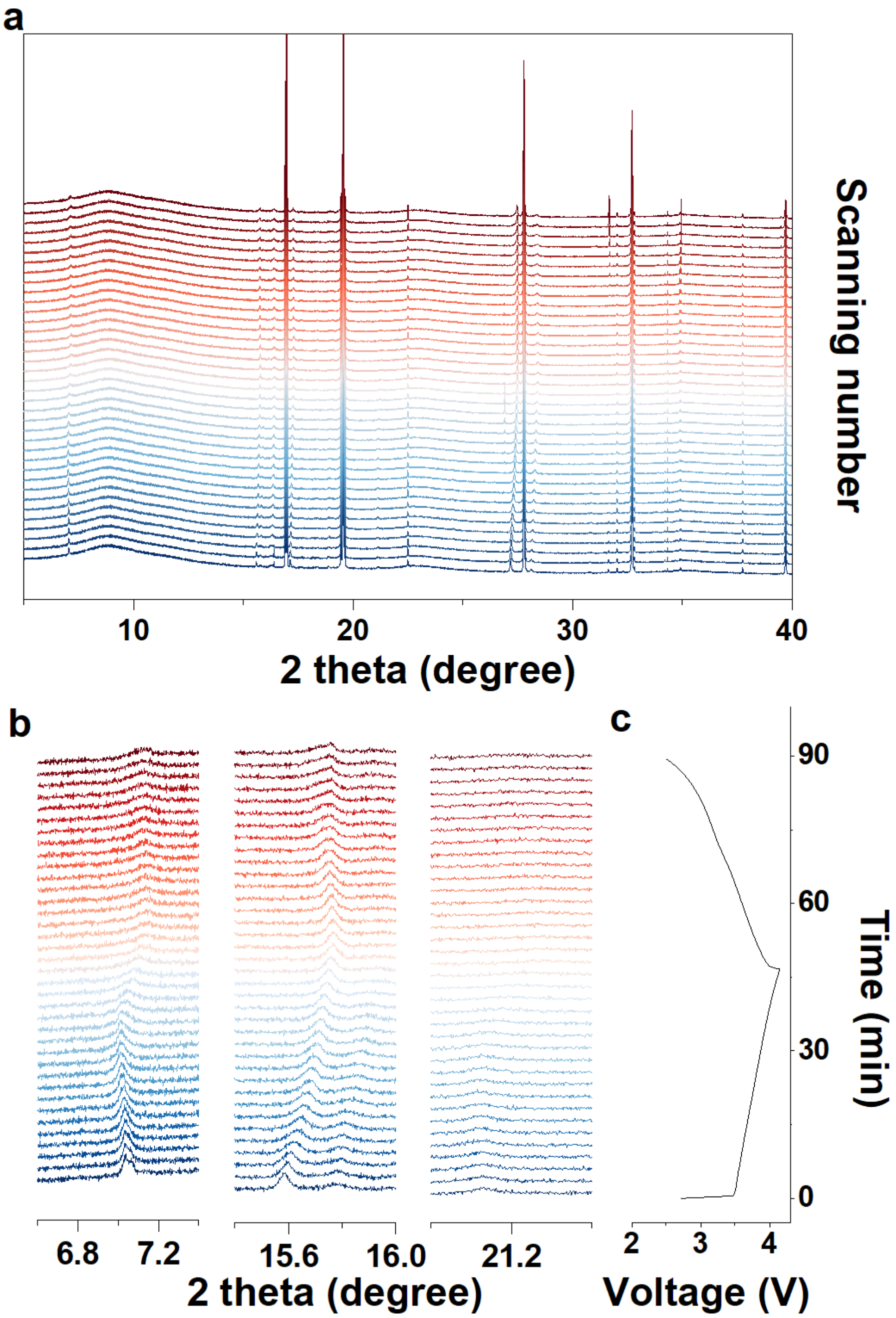

Supplementary Figure S35 a) In-situ synchrotron-based XRD pattern of NLCMMTO during the second charge/discharge at 120 mA g^{-1} in the voltage range of 2.5-4.15 V. b) The selected area in Figure S35a. c) Profile of charging/discharging of NLCMMTO at 120 mA g^{-1} and room temperature (25 ± 2°C).

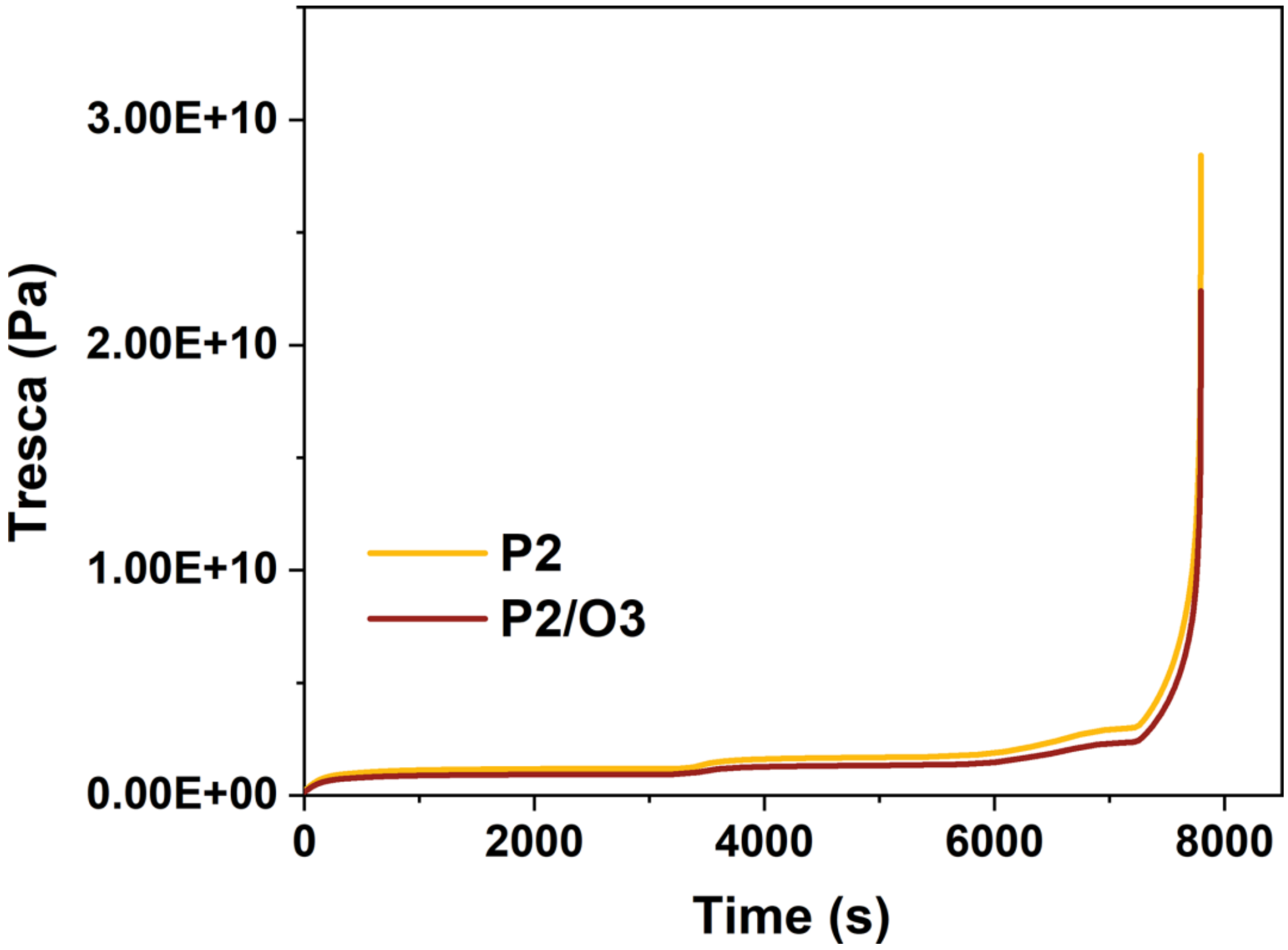

Supplementary Figure S36 Averaged stress calculation simulation results for different phase compositions of NLCMMTO, including pure P2 and P2+O3 mixed phases.

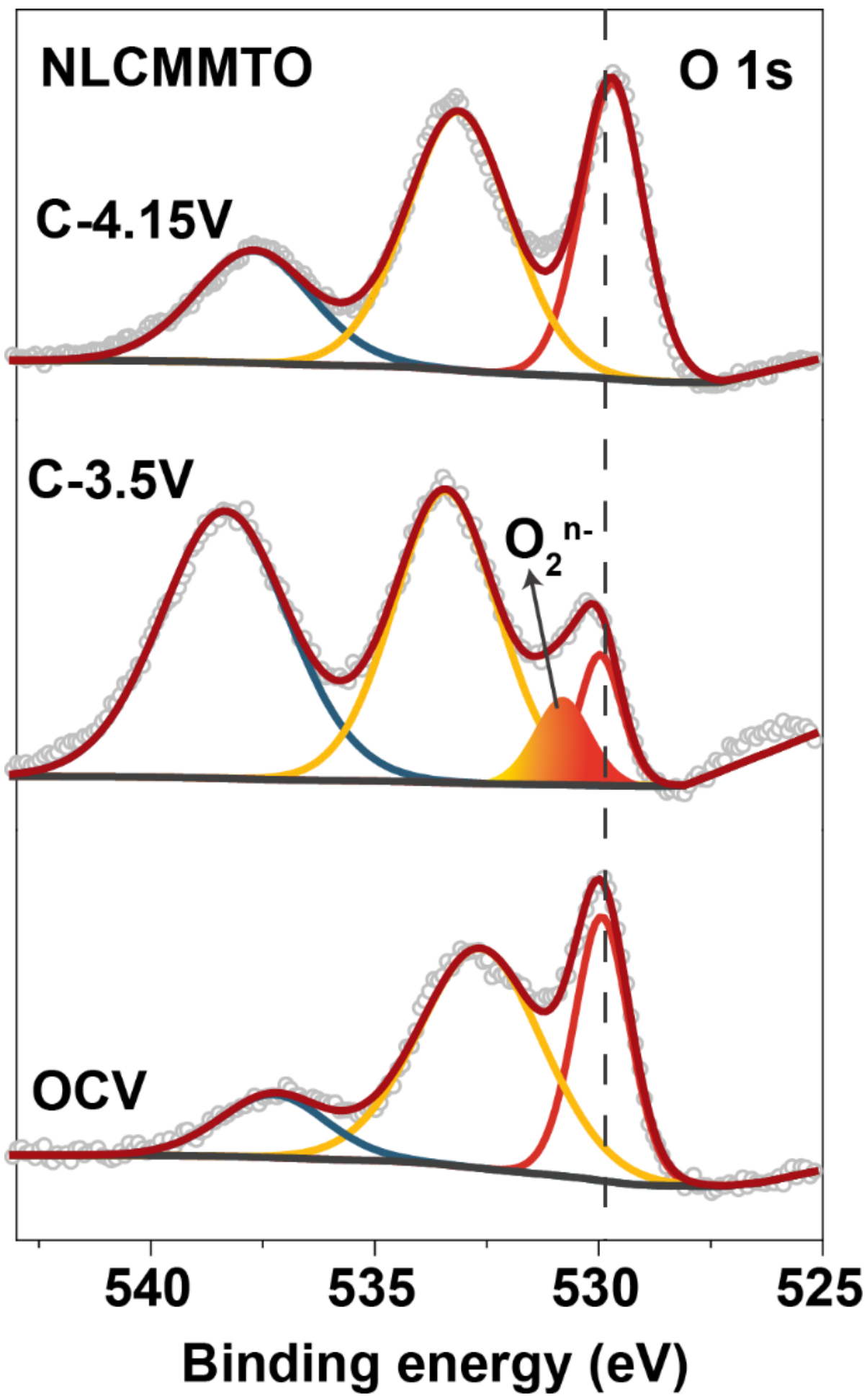

Supplementary Figure S37 XPS spectra of O 1s in NLCMMTO electrodes collected in specific voltage during the first charging/discharging cycle at 10 mA g^{-1} and room temperature (25 ± 2°C).

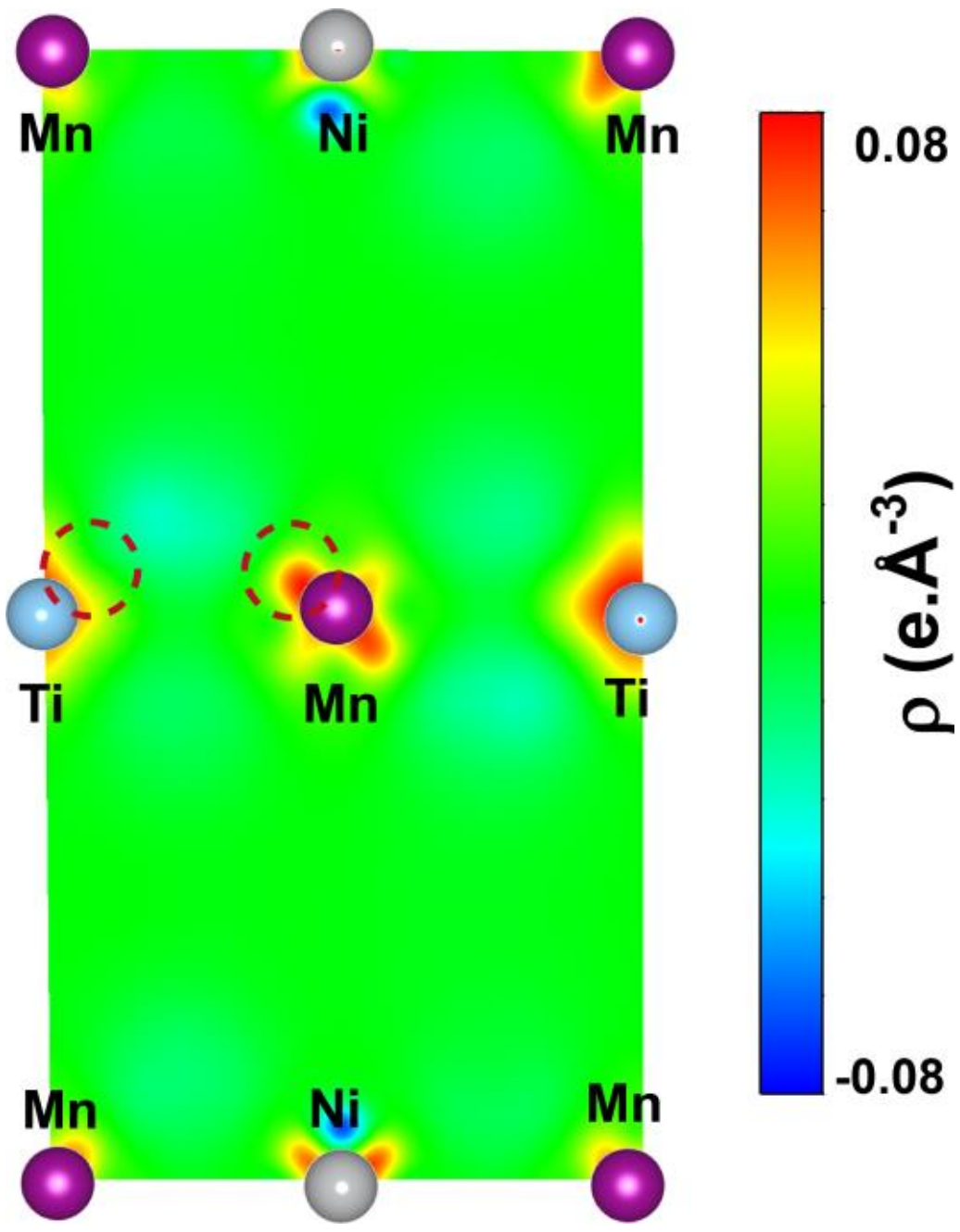

Supplementary Figure S38 Calculation of the electron localization function of NMMTO.

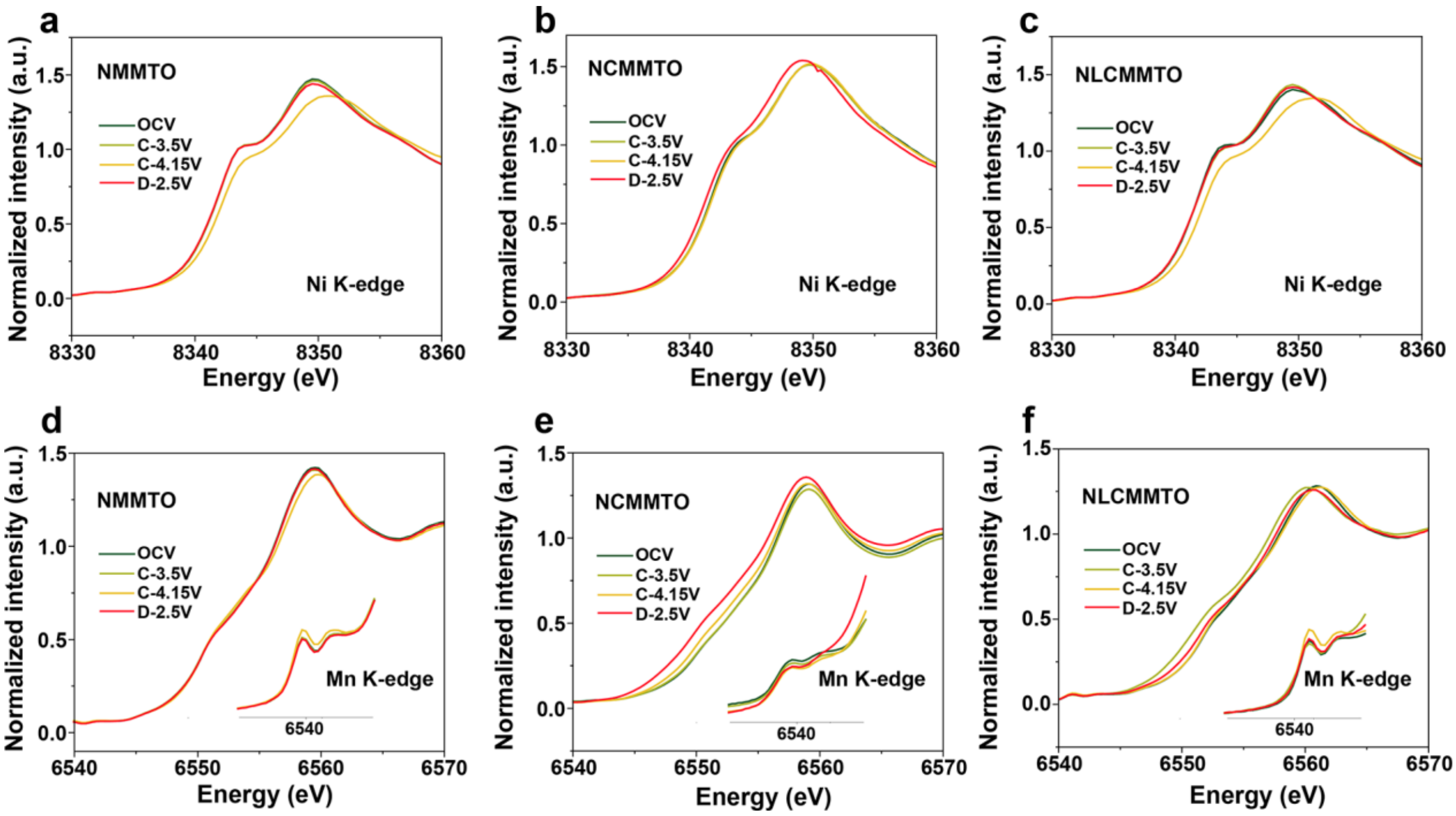

Supplementary Figure S39 a, d) Ni and Mn XANES of NMMTO ex situ electrodes, b, e) Ni and Mn XANES of NCMMTO ex situ electrodes, c, f) Ni and Mn XANES of NLCMMTO ex situ electrodes.

These electrode samples were collected by disassembling batteries at specific voltage during the first cycling at 10 mA g^{-1} and room temperature (25 ± 2°C).

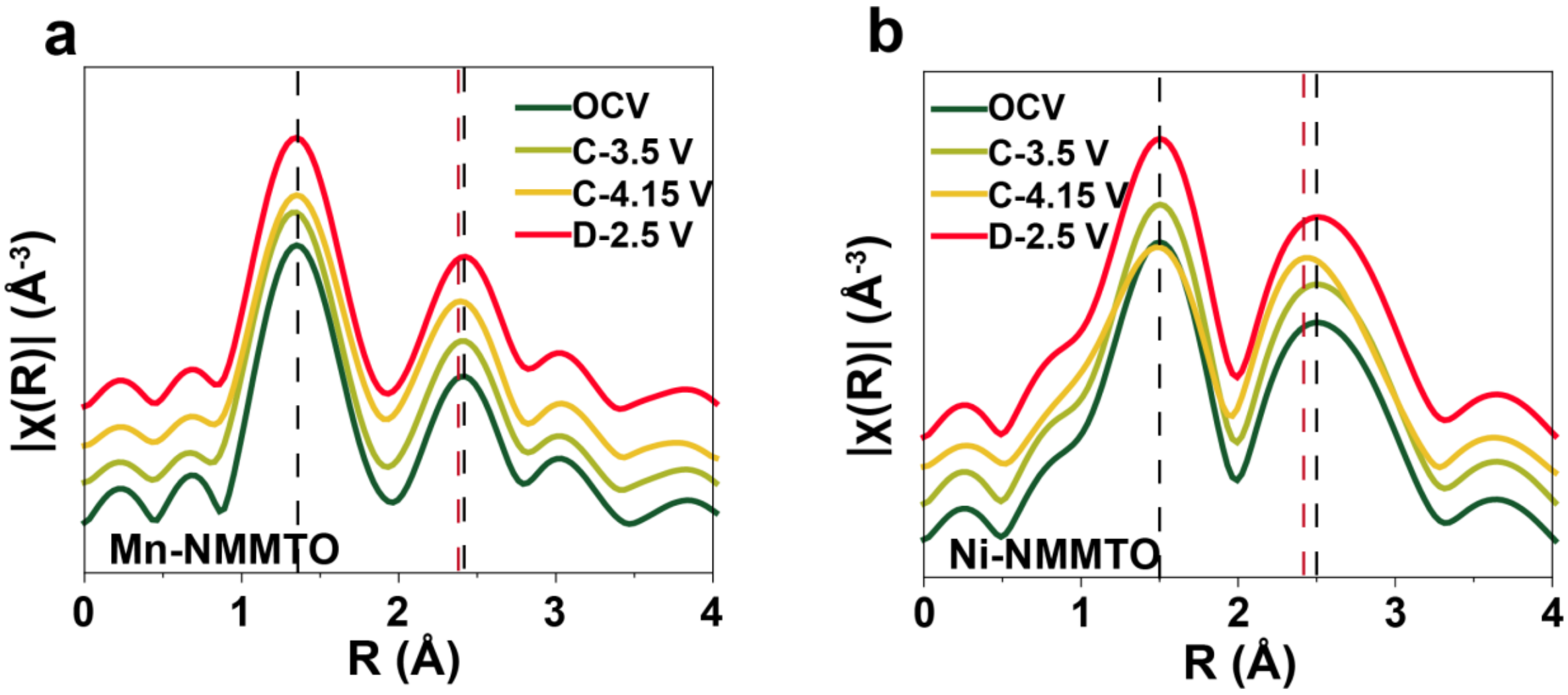

Supplementary Figure S40 a) Mn and b) Ni EXAFS in NMMTO at different charge states.

These electrode samples were collected by disassembling batteries at specific voltage during the first cycling at 10 mA g^{-1} and room temperature (25 ± 2°C). Due to Mn and Ni were oxidized, the TM-TM bond length shortens since their ionic radius decrease, like most publication mentioned.

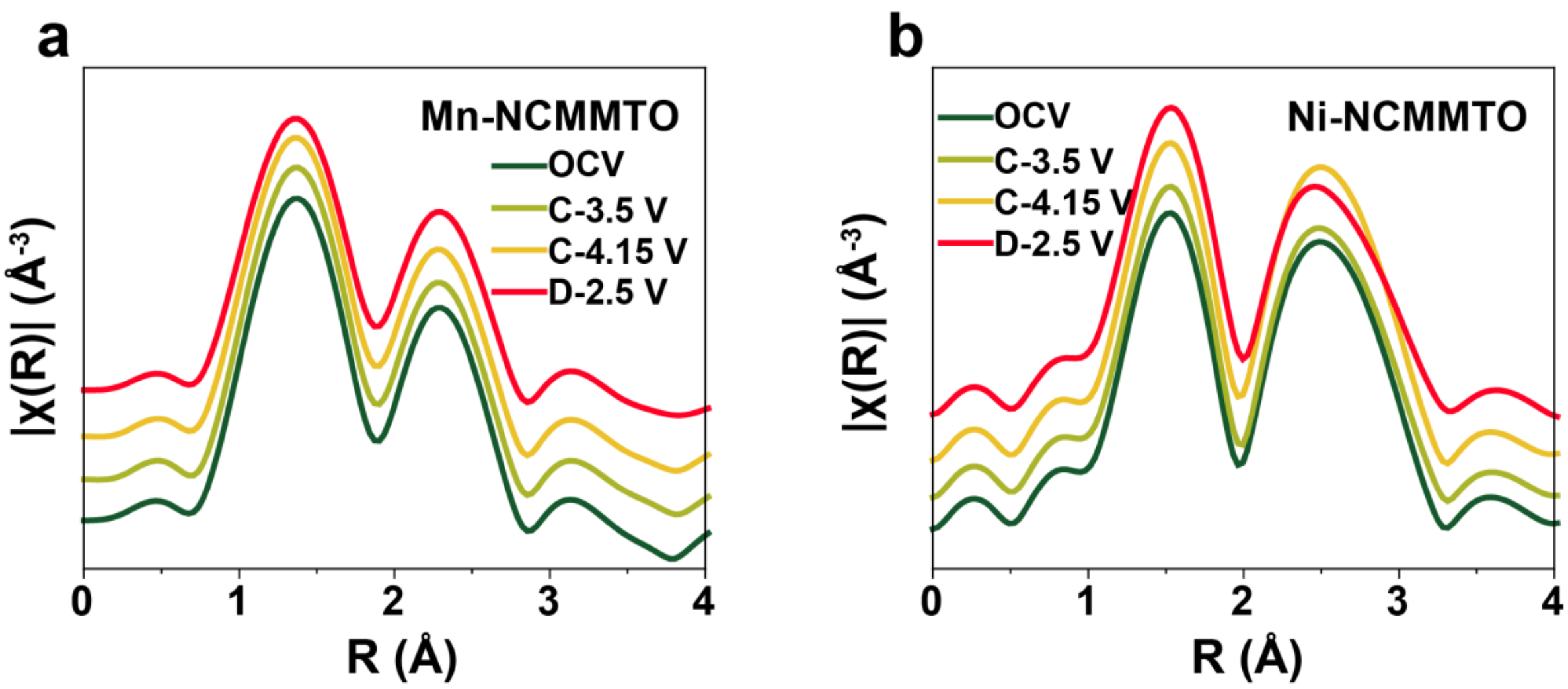

Supplementary Figure S41 a) Mn and b) Ni EXAFS in NCMMTO at different charge states.

These electrode samples were collected by disassembling batteries at specific voltage during the first cycling at 10 mA g^{-1} and room temperature (25 ± 2°C). After Cu induction, although Mn and Ni were oxidized, with the assistance of CuO_4, the TM-TM bond length could remain stable.

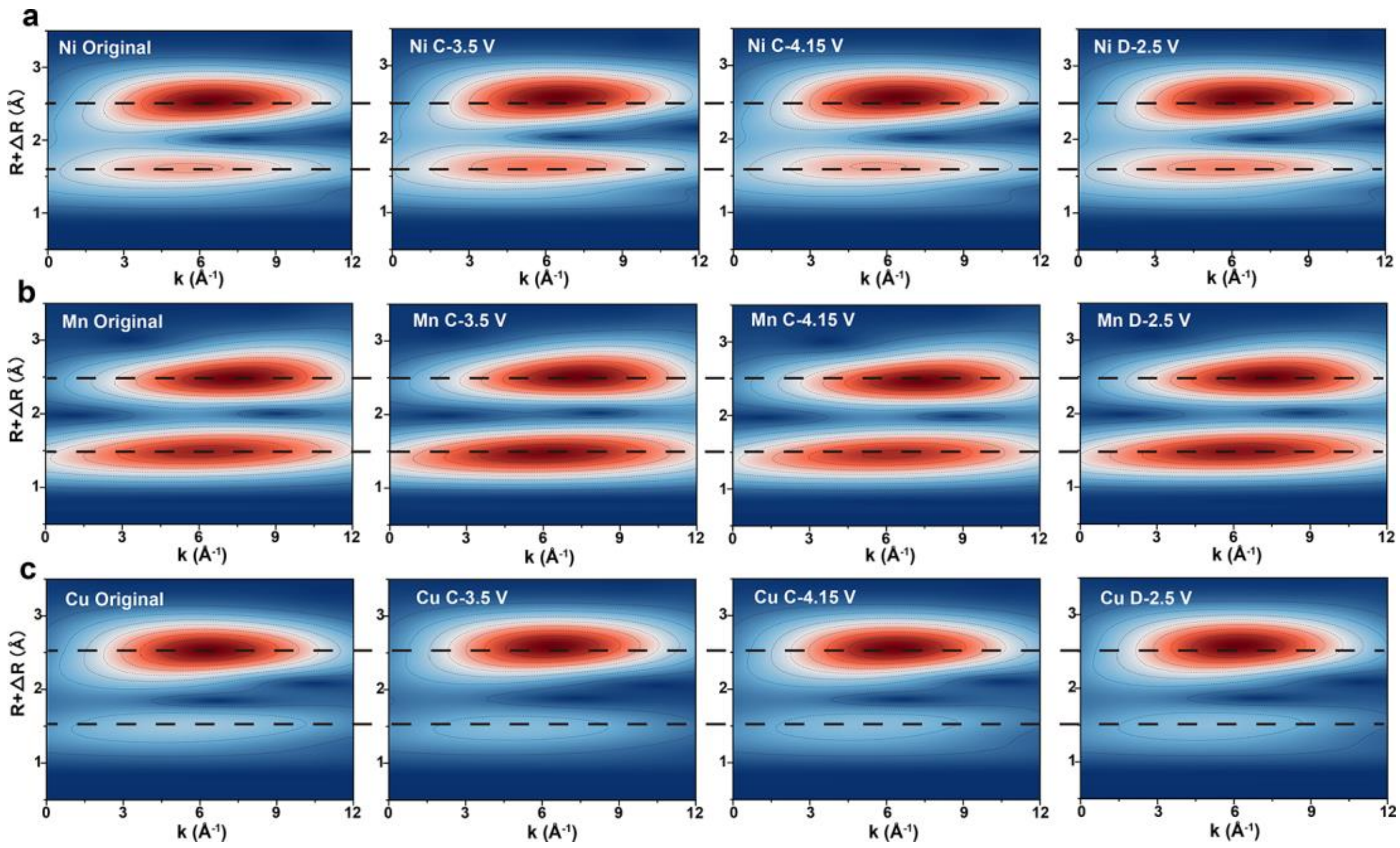

Supplementary Figure S42 a) Ni, b) Mn and c) Cu K-edges WT-EXAFS spectra of NLCMMTO at different voltage states.

These electrode samples were collected by disassembling batteries at specific voltage during the first cycling at 10 mA g^{-1} and room temperature (25 ± 2°C).

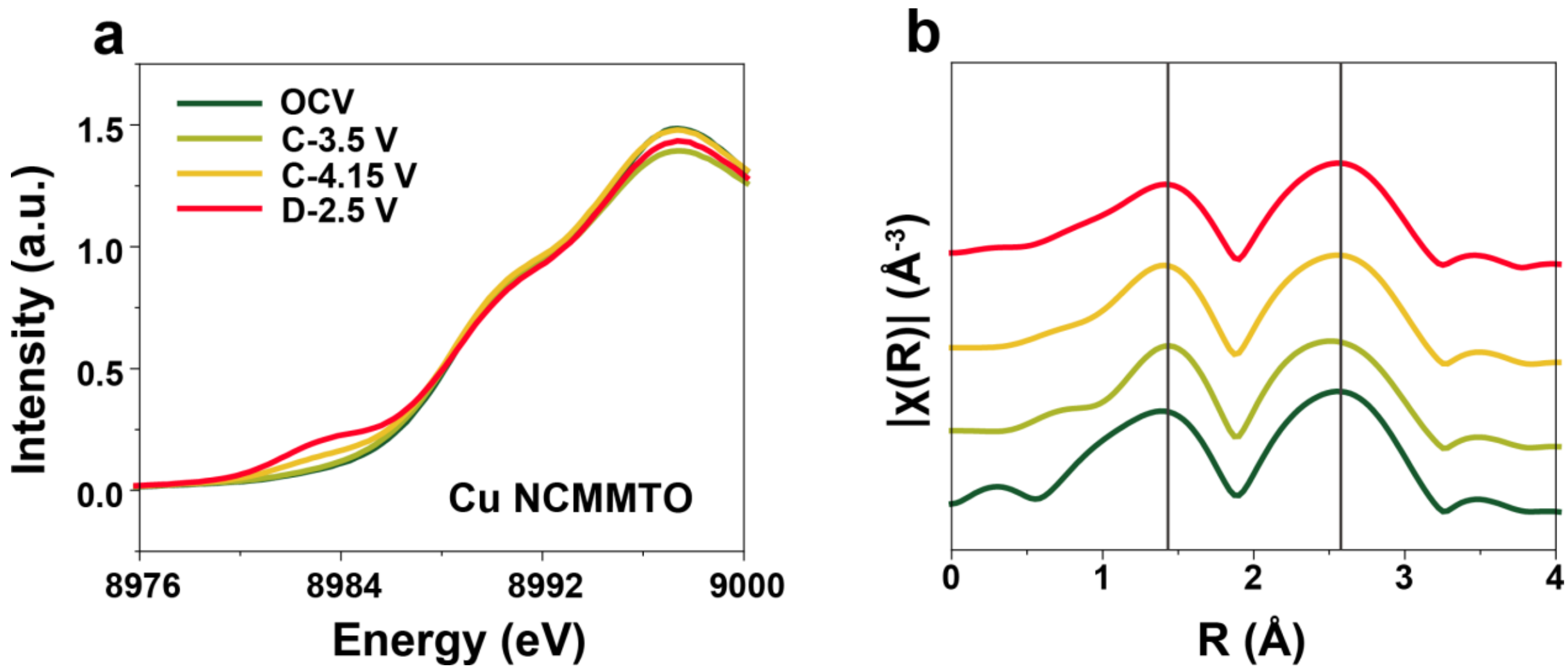

Supplementary Figure S43 a) XANES of Cu K-edge and b) EXAFS in NCMMTO at different charge states.

These electrode samples were collected by disassembling batteries at specific voltage during the first cycling at 10 mA g^{-1} and room temperature (25 ± 2°C).

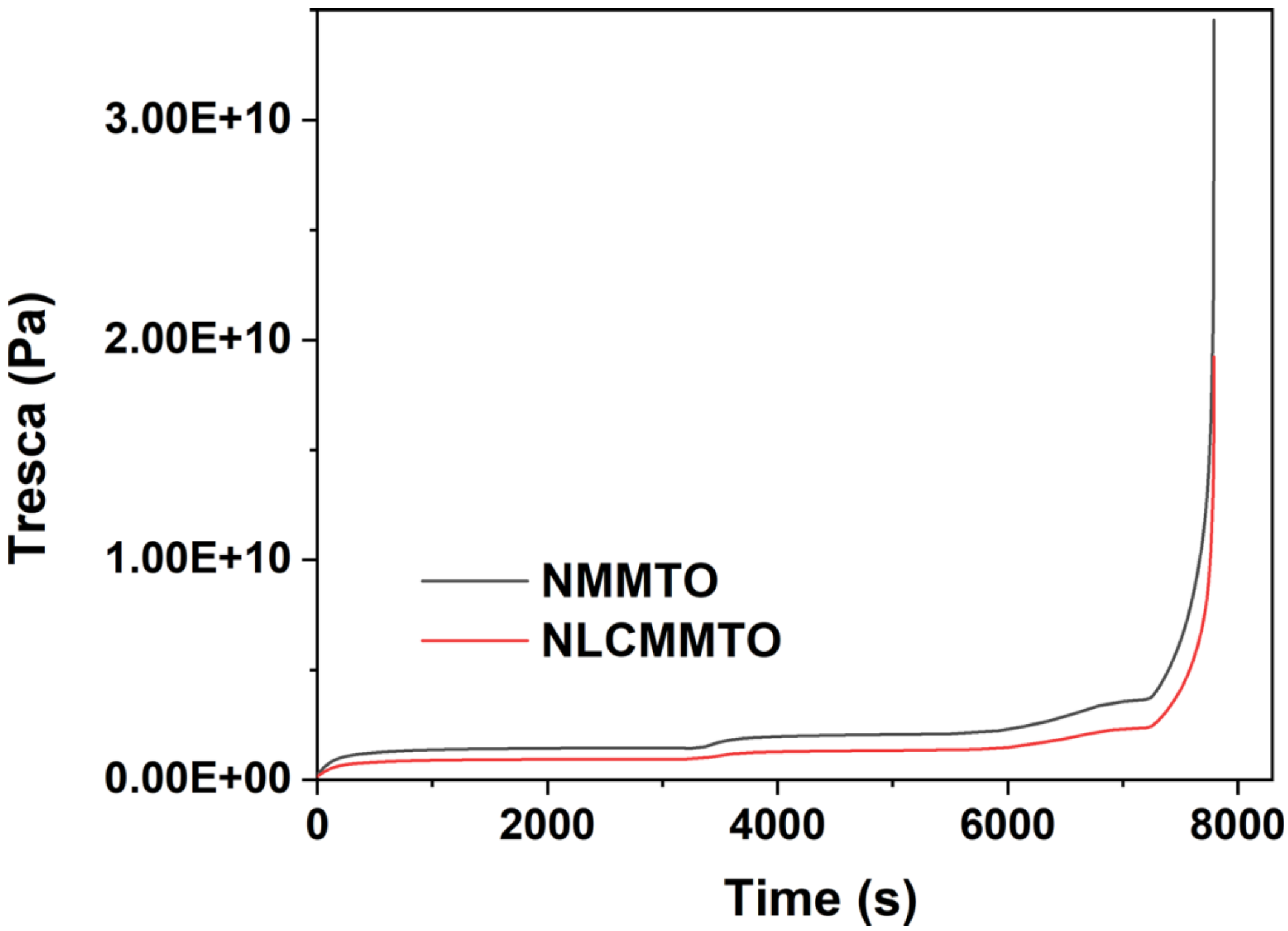

Supplementary Figure S44 Average simulation calculation results of NMMTO and NLCMMTO combinations.

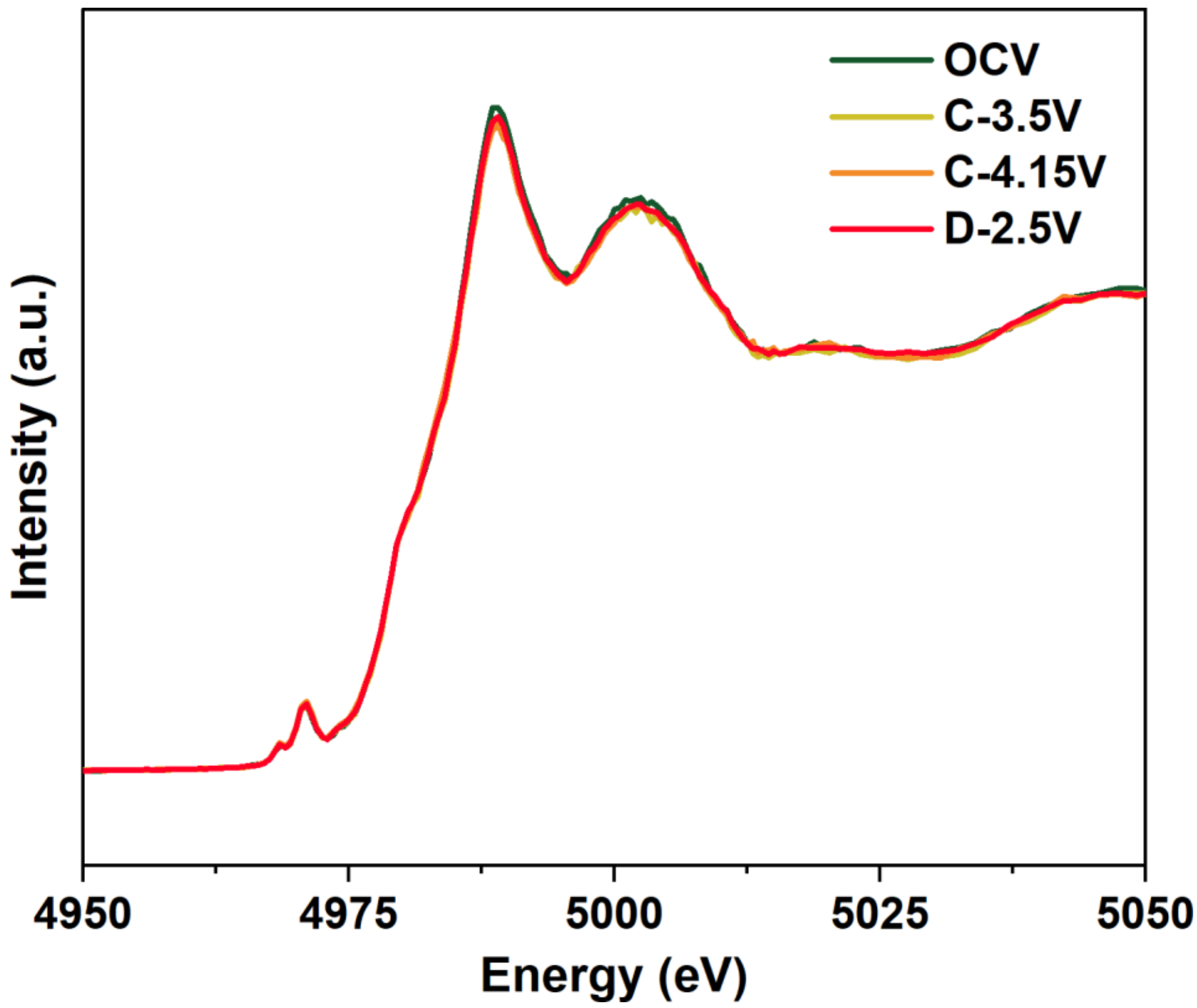

Supplementary Figure S45 Ti K-edge XAS spectra of NLCMMTO at different charging states. These electrode samples were collected by disassembling batteries at specific voltage during the first cycling at 10 mA g^{-1} and room temperature (25 ± 2°C).

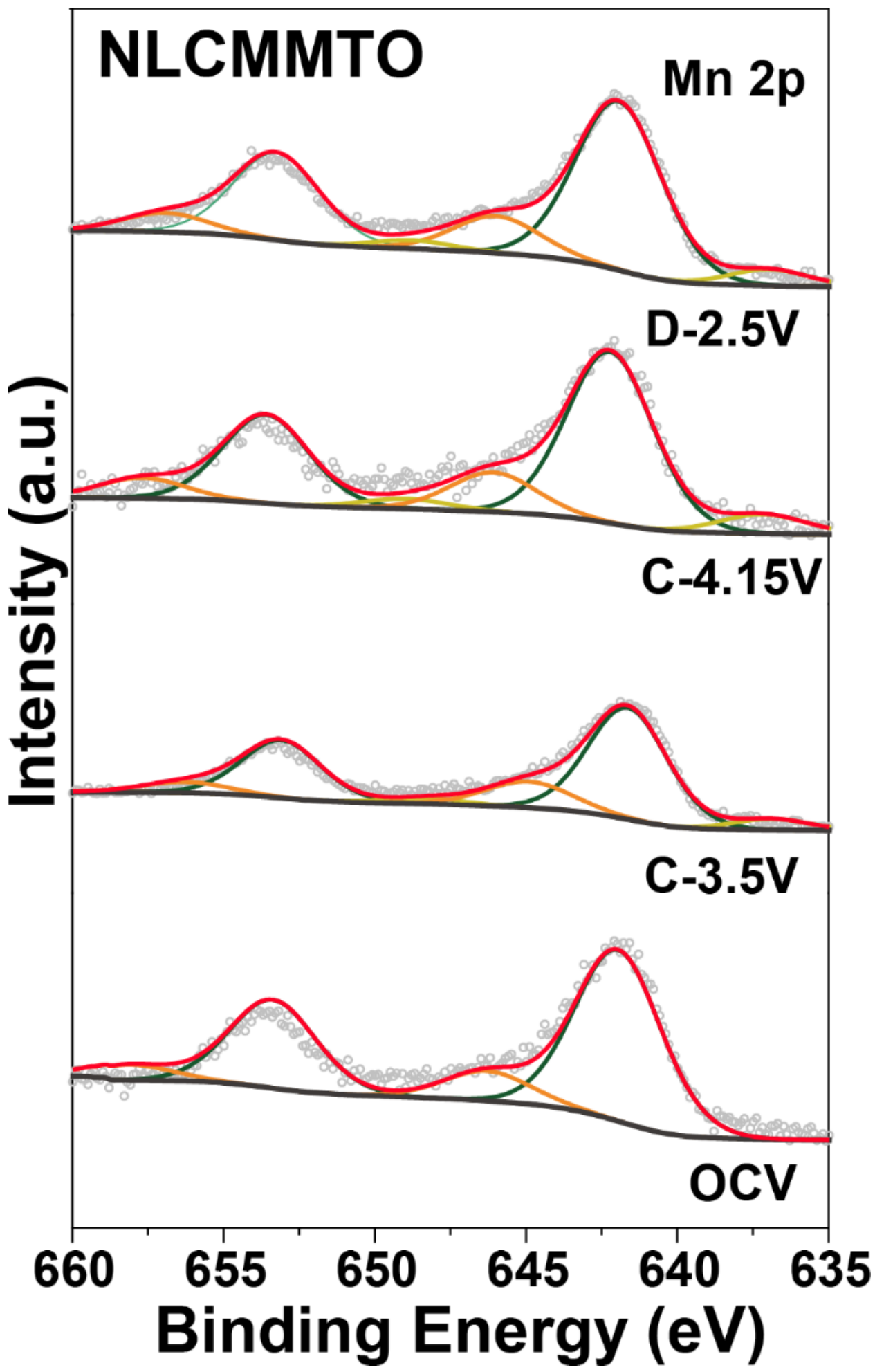

Supplementary Figure S46 Mn 2p XPS spectrum of NLCMMTO electrodes at different charging states.

These electrode samples were collected by disassembling batteries at specific voltage during the first cycling at 10 mA g^{-1} and room temperature (25 ± 2°C).

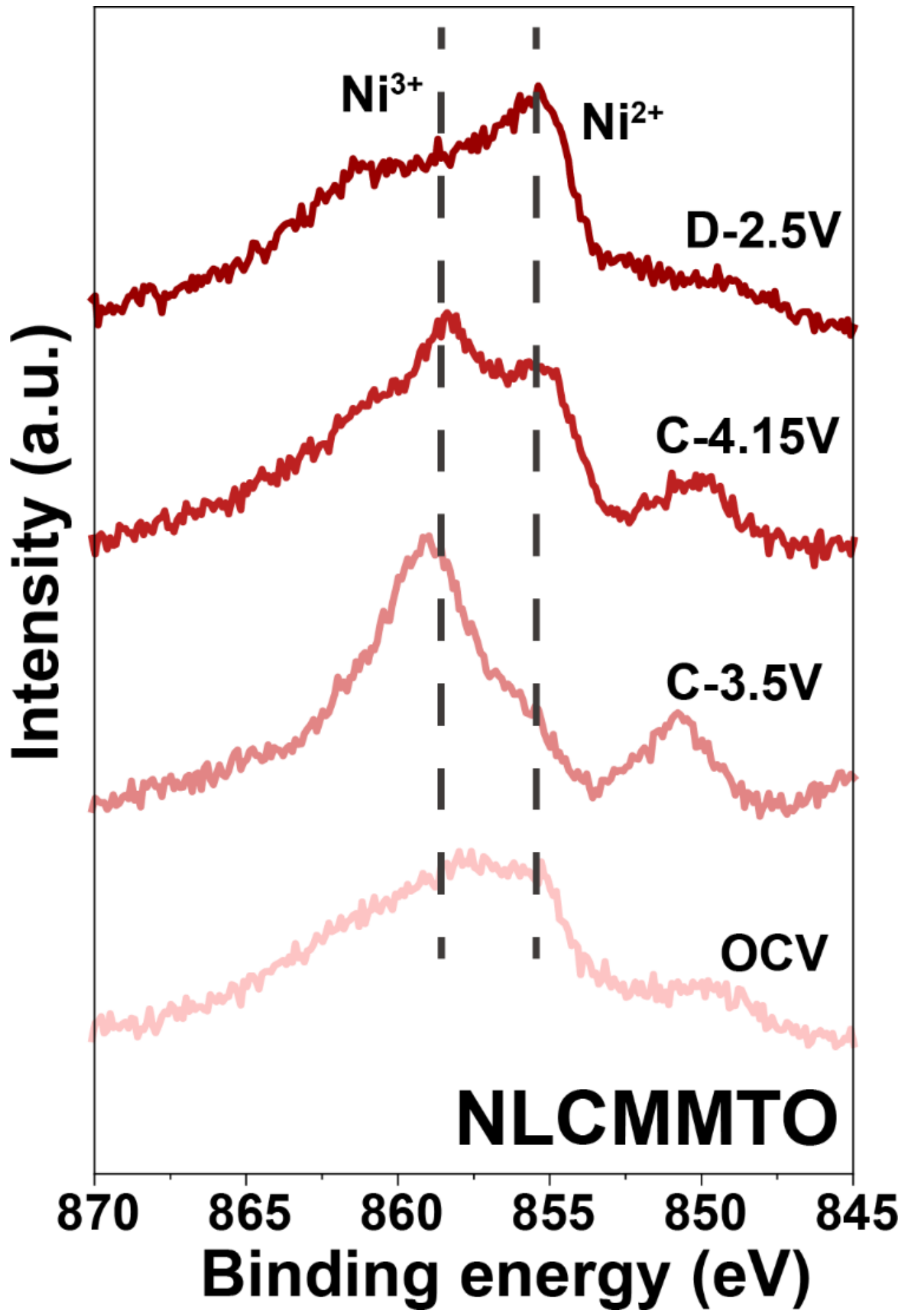

Supplementary Figure S47 Ni 2p XPS spectrum of NLCMMTO electrodes at different charging states.

These electrode samples were collected by disassembling batteries at specific voltage during the first cycling at 10 mA g^{-1} and room temperature (25 ± 2°C).

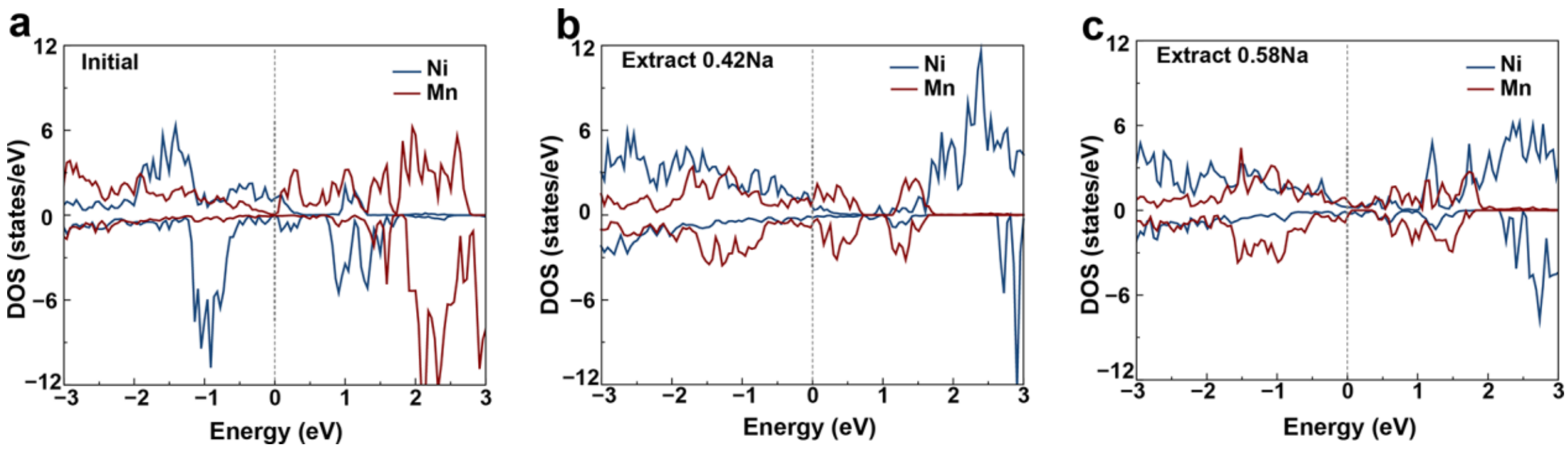

Supplementary Figure S48 DOS calculation of a) original, b) extract 0.42 Na and c) extract 0.58 Na of simplified O3-based in NLCMMTO. The structural models shown in Figure S48 are provided in Supplementary Data 4, Supplementary Data 5 and Supplementary Data 6, respectively.

Supplementary Table 1 | Crystallographic parameters of NLCMMTO refined by X-ray diffraction Rietveld refinement. Space group: $P6_3/mmc$, a = 2.92449 Å, c = 11.12987 Å, V = 82.437 Å^3, R-3m, a=2.92668 Å, c= 16.69332 Å, V = 123.829 Å^3 wR = 6.49 %

Atom	x	y	z	Frac	Uiso (Å^2)
Ni	0	0	0	0.164	0.07324
Cu	0	0	0	0.075	0.43555
Ti	0	0	0	0.3	0.87595
Mn	0	0	0	0.426	0.06365
O	0.6667	0.3333	0.07006	0.975	0.1204
Na1	0	0	0.25	0.073	0.05771
Na2	0.3333	0.6777	0.25	0.582	1.4663

Atom	x	y	z	Frac	Uiso (Å^2)
Na	0	0	0	0.108	0.97122
Mn	0	0	0.5	0.480	0.01267
Li	0	0	0.5	0.100	0.01267
Ni	0	0	0.5	0.200	0.01267
Mg	0	0	0.5	0.02	0.01267
O	0	0	0.147774	1	0.31056

Supplementary Table 2 | The weight fraction of P2 and O3 of NMMTO, NCMMTO, and NLCMMTO.

Cathode Material	P2	O3
NMMTO	0.7697	0.2303
NCMMTO	0.9759	0.0241
NLCMMTO	0.8201	0.1799
