# Supplementary material for: Self-adaptive layered oxide cathodes driven by charge reconfiguration for sodium-ion batteries
Source: Nat Commun. 2026 Sep 28;17:11197. doi: 10.1038/s41467-026-78040-8 (PMC13714294; doi:10.1038/s41467-026-78040-8)
Supplement: Supplementary file 2 [file 41467_2026_78040_MOESM2_ESM.pdf]

Description of Additional Supplementary Files

Supplementary Data 1: Atomic coordinates of the simplified pristine P2-based $Na_{2/3}Ni_{1/6}Li_{1/18}Cu_{1/18}Mg_{1/18}Mn_{1/3}Ti_{1/3}O_2$ computational structural model.

Supplementary Data 2: Atomic coordinates of the simplified P2-based $Na_{2/3}Ni_{1/6}Li_{1/18}Cu_{1/18}Mg_{1/18}Mn_{1/3}Ti_{1/3}O_2$ computational structural model after extraction of 0.4 Na.

Supplementary Data 3: Atomic coordinates of the simplified P2-based $Na_{2/3}Ni_{1/6}Li_{1/18}Cu_{1/18}Mg_{1/18}Mn_{1/3}Ti_{1/3}O_2$ computational structural model after extraction of 0.6 Na.

Supplementary Data 4: Atomic coordinates of the simplified pristine O3-based $Na_{2/3}Ni_{1/6}Li_{1/18}Cu_{1/18}Mg_{1/18}Mn_{1/3}Ti_{1/3}O_2$ computational structural model.

Supplementary Data 5: Atomic coordinates of the simplified O3-based $Na_{2/3}Ni_{1/6}Li_{1/18}Cu_{1/18}Mg_{1/18}Mn_{1/3}Ti_{1/3}O_2$ computational structural model after extraction of 0.42 Na.

Supplementary Data 6: Atomic coordinates of the simplified O3-based $Na_{2/3}Ni_{1/6}Li_{1/18}Cu_{1/18}Mg_{1/18}Mn_{1/3}Ti_{1/3}O_2$ computational structural model after extraction of 0.58 Na.

Supplementary Video 1: Electric filed distribution of CoM in O3 region of $Na_{2/3}Ni_{1/6}Li_{1/18}Cu_{1/18}Mg_{1/18}Mn_{1/3}Ti_{1/3}O_2$.

Supplementary Video 2: Electric filed distribution of CoM in P2 region of $Na_{2/3}Ni_{1/6}Li_{1/18}Cu_{1/18}Mg_{1/18}Mn_{1/3}Ti_{1/3}O_2$.

Supplementary Video 3: Electrostatic potential distribution in O3 region of $Na_{2/3}Ni_{1/6}Li_{1/18}Cu_{1/18}Mg_{1/18}Mn_{1/3}Ti_{1/3}O_2$.

Supplementary Video 4: Electrostatic potential distribution in P2 region of $Na_{2/3}Ni_{1/6}Li_{1/18}Cu_{1/18}Mg_{1/18}Mn_{1/3}Ti_{1/3}O_2$.
