# Supplementary material for: Self-adaptive layered oxide cathodes driven by charge reconfiguration for sodium-ion batteries
Source: Nat Commun. 2026 Sep 28;17:11197. doi: 10.1038/s41467-026-78040-8 (PMC13714294; doi:10.1038/s41467-026-78040-8)
Supplement: Supplementary file 3 [file 41467_2026_78040_MOESM8_ESM.pdf]

nature portfolio

Peer Review File

Self-adaptive Layered Oxide Cathodes Driven by Charge Reconfiguration for Sodium-ion Batteries

Corresponding Author: Professor Yao Xiao

This file contains all reviewer reports in order by version, followed by all author rebuttals in order by version.

Version 0:

Reviewer comments:

Reviewer #1

(Remarks to the Author)
This manuscript reports the design of a layered oxide cathode, Na2/3Ni1/6Li1/18Cu1/18Mg1/18Mn1/3Ti1/3O2, which exhibits stable electrochemical performance over prolonged cycling. The authors attribute the enhanced cycling stability to a self-adaptive volume regulation mechanism driven by charge reconfiguration, supported by a set of in situ and ex situ characterizations. They propose that Ni–O–Mn interactions play a critical role in mitigating stress accumulation and maintaining structural stability during cycling. In situ synchrotron XRD reveals unusual changes in the interlayer spacing, while 4D-STEM provides direct experimental evidence supporting this atypical structural evolution. Furthermore, the authors employ PDF analysis, XPS, and theoretical calculations to establish a mechanistic link between Ni-O2n--Mn superexchange interactions, stress redistribution, and long-term electrochemical stability. The role of each constituent element is systematically investigated, and the unconventional charge compensation mechanism is directly observed by in situ XAS. Overall, this work proposes a cathode chemistry based on earth-abundant transition metals and demonstrates durability under high current densities, which is important for the development of large-scale sodium-ion batteries.
Taken together, the intriguing evolution of interlayer spacing combined with the detailed mechanistic investigation makes this work potentially suitable for Nature Communications. However, several aspects related to phrasing, clarity, and presentation require further strengthening before the manuscript can fully meet the standards of this journal. Detailed comments are provided below.
1. The idea that adjustable repulsive force of O-O caused by charge reconfiguration leading to self-adaptive volume behaviour is innovative. Some researchers argue that the reduction of distance of TM layers at the high voltage might be consequence of the incorporation of Cu, what do authors think of this explanation, please discuss with this viewpoint.
2. It is impressive by the depth of the authors’ investigation into the role of Cu, which is both scientifically significant and thought-provoking. To further strengthen the clarity of the overall mechanism, the authors might consider expanding the explanation regarding Li to more clearly relate the function of each element to the enhanced electrochemical performance. This addition would undoubtedly strengthen the manuscript.
3. The structural evolution during charging and discharging was tracked by in-situ synchrotron XRD offering rich and important information on microstructural changes of the material in this draft. In especial, the real-time tracking of structural changes under high current densities was also be studied, which offer valuable design principles for commercially viable cathode materials capable of fast charging and discharging. Thus, authors should clarify the organic connection between structure characterization and electrochemical performance improvement and be more sharply articulated in the discussion.
4. The mechanism of charge compensation was directly observed by in-situ XAS, which is interesting and offers important scientific insights and serves as a guiding reference for other experiments seeking to probe the dynamic behavior of various elements. But it is not clear how the in-situ XAS measurements have been made. More experimental details on parameters are needed.
5. To make it clearer for readers, some subtitles and figure captions should be described more accurately. For example, the Fig 6h, “adaptive stress distribution driven by charge reconfiguration diagram” didn’t show the information on adaptive stress distribution; instead, it would be better to be described with “the fast charge transfer between the TM ions”.
6. The manuscript is figure-heavy and risks overwhelming readers. Consolidation of adding some clearer mechanistic schematics would improve readability.
7. We observe that both ex-situ XAS and in-situ XAS are used in the study. Since both techniques can probe the K-edge of transition metal elements, what is the fundamental difference between them, and why did the authors conduct both experiments?

Reviewer #2

(Remarks to the Author)
The manuscript presents a high-entropy layered oxide cathode (NLCMMTO) featuring "self-adaptive volume" behavior for Na-ion batteries, where Cu-driven O–O dimer formation and Ni–O–Mn superexchange are proposed to enhance stress management and cycling stability. The work leverages advanced characterization and aims to move beyond traditional high-entropy concepts by targeting specific electronic interactions. While the study is innovative and well-supported experimentally, strengthening the quantitative connection between mechanistic claims, structure–property relationships, and stress analysis would further clarify the manuscript's novelty and significance. Please see the below comments.

1. The long term cycling performance (82% capacity retention after 1000 cycles at 2C, from 88.6 to 72.8 mAh g^{-1}) is impressive in terms of stability, but the absolute capacity seems to be modest compared with recent state of the art layered Na cathodes in a similar voltage window. So. please place the performance of NLCMMTO in a more quantitative context by comparing specific capacity, average operating voltage, and energy density with relevant recent O3/P2 and high entropy Na layered oxides (including the works already cited in Refs. 1–4, 12, 18, 21, etc.). Also it would be helpful if the authors could calculate the theoretical capacity of NLCMMTO and discuss the fraction of capacity achieved at 0.1C and 2C.

2. I think a key novelty of the manuscript is the claim that Cu substitution promotes O–O dimer formation, which then drives charge reconfiguration and self adaptive volume behavior. The main experimental evidence seems to be the O 1s XPS feature assigned to M–(O–O) species at C 3.5 V (Fig. 4b), along with changes in O K edge spectra. While this is suggestive, O–O dimer assignments are often debated. So I suggest that authors should provide a clear justification for assigning this feature to O–O dimers, e.g., by: 1) Comparing to reference spectra of well defined peroxo/superoxo species or to previous reports (quantitatively, not only qualitatively). 2) Providing DFT derived O–O distances and charge analysis demonstrating peroxo/superoxo like species and reporting O–O bond lengths in the computed structures and clarifying whether they are in the typical range expected for peroxo like dimers. In addition, the Cu K edge XANES/EXAFS analysis (Fig. 4g–i) is used to support a transition from CuO_6 octahedra to planar CuO_4. To more deeply understand this phenomenon, I suggest author do quantify the Cu–O bond length changes upon charging (from EXAFS fitting) and report coordination numbers and clearly present the models used for the CuO_6 to CuO_4 conversion, and describe how this geometrical change makes O–O dimer creation possible. At the current version, the Cu evolution is described qualitatively; a more quantitative EXAFS analysis would significantly strengthen the claim that Cu is the active driver of O O dimer formation rather than a passive "filler."

3. NLCMMTO introduces both Cu and Li relative to NMMTO, and Cu relative to NMMTO in NCMMTO. To demonstrate that Cu is the key element responsible for O–O dimers and the related self adaptive behavior, the roles of Cu and Li need to be more carefully disentangled. For example, can the authors show that NCMMTO (Cu but no Li) already exhibits clear O–O dimer signatures, whereas Li primarily stabilizes the framework and mitigates Jahn–Teller distortions (as briefly suggested in the O K edge discussion)? A more systematic comparison of O K edge and O 1s spectra among NMTO, NMMTO, NCMMTO, and NLCMMTO at matched states of charge would be very helpful to understand the role of each element.

4. Authors state that the "self adaptive volume" and stress relief mechanism is attributed to Ni–O_2^{n}–Mn superexchange mediated by Cu induced O dimers. It is a key point of the manuscript, but at current version, it seems to be based mainly on inference rather than direct evidence. Although conducting entirely new experiments may be difficult, the authors should either: 1) provide some experimental analysis that directly supports the observed changes in magnetic interactions upon Cu substitution and charging, or 2) considerably qualify their conclusion and indicate that superexchange remains a possible, but not fully confirmed, mechanism.

5. The Tresca (spelled "Treca" in the manuscript) stress calculations show that NLCMMTO has lower and more uniform stress than NMMTO at 4.15 V. Please clarify how the calculated structures used for stress analysis were chosen (with or without O–O dimers, with which Na content, etc.), and how the stress distribution correlates with the observed lattice parameter evolution and with the measured electrochemical cycling stability. If possible, show a direct correlation between calculated stress and experimental $\Delta c/c$ or $\Delta V/V$ for the different compositions.

6. The manuscript briefly states that the O3 phase provides mechanical support, but this is not substantiated. Please either: 1) Provide some evidence that varying O3 content (e.g., via different synthesis conditions) changes cycling stability and/or lattice evolution, or 2) Reframe the statement more cautiously, acknowledging that the precise role of the O3 fraction is not fully understood. Also, if the interfaces between P2 and O3 domains are believed to contribute to stress accommodation or Na ion transport (as often suggested in P2/O3 intergrowth systems), this should be discussed explicitly, possibly with schematic or DFT insights.

7. Ti constitutes a large fraction of the transition metal sublattice ($Ti_{1/3}$), yet its functional role is only briefly mentioned. Please clarify whether Ti is entirely electrochemically inactive in the investigated voltage range (2.5–4.15 V) and provide evidence (e.g., Ti K edge XAS or indirect arguments) if available.

8. The configurational entropy argument is used to rationalize improved stability, but the manuscript also emphasizes very specific roles of individual elements (Cu → O dimers, Li → relieve interlayer deformation, Ti → framework strengthening, etc.). It would be useful to distinguish between the general stabilizing effect from high configurational entropy and the specific electronic or structural influences of individual elements. For instance, could the authors provide configurational entropy values (S_conf) for NMTO, NMMTO, NCMMTO, and NLCMMTO, and analyze whether performance aligns with these entropic values or if the contributions from Cu, Li, or Ti are more significant than the entropy itself?

9. Treca stress" should presumably be "Tresca stress." Please correct and briefly define the chosen stress criterion the first time it appears. The phrase 'self-adaptive volume behavior' is important to the manuscript but is somewhat vague. Providing a definition in the Introduction would make its meaning more precise.

10. In several main figures (e.g., in situ synchrotron XRD maps and in situ XAS), the scale bars, color bars, and labels are relatively small and difficult to read. And please carefully proofread the manuscript for minor grammatical errors and typos (e.g., "distorti00on", "Treca", "planer CuO4", etc.).

Reviewer #3

(Remarks to the Author)
The authors report the synthesis and advanced characterization of a layered oxide cathode material, Na2/3Ni1/6Li1/18Cu1/18Mg1/18Mn1/3Ti1/3O2 (NLCMMTO). They highlight NLCMMTO as a next-generation compound of interest for sustainable energy infrastructures, due to its electrochemical stability, resource abundance, and scalability. This material exhibits a self-adaptive volume regulation, with more attractive properties than the parent compound NMMTO, and NCMMTO. Several experimental techniques, some of which are supported by theoretical calculations, are used to provide a broad understanding of the structural and electronic properties of these compounds during charging and discharging, in line with their electrochemical properties. High quality X-ray absorption spectroscopy (ex situ and in situ), XPS, 4D-STEM, as well as high-resolution XRD provide a significant amount of information.

This is a thorough report, impressive by the large quantity of data reported, and the clear objective to systematically evaluate the evolution of the electrochemical properties through the structural, chemical , and electronic properties. Nevertheless, although the manuscript contains a very descriptive presentation of the results, the presentation and interpretation of some results could be clarified, and the discussion of the results in a broader context and their impact in the field is missing. One of the major issues in the manuscript is the lack of explanation for the role played by Li on the properties of NLCMMTO. Therefore, it is my understanding that the current version of the manuscript lacks the depth and impact expected to be considered for publication. I provide additional comments below.

• The manuscript would benefit from professional editing to correct English grammar, missing words and inconsistencies in the construction of sentences throughout the manuscript, including captions of figures in the main text and SI. Some examples:

- "However, in NLCMMTO that adding Li, the peak at 533 eV is slightly weaker demonstrating relieves the interlayer deformation stress in the TM layers."
- "The crystalline fact..."
- "… shell layer, the closely atomic…"
- "… all reflection peaks are kept combination with…"
- "… distorti00on…"
- "To gain the more direct the oxygen evolution behavior, the …"
- "Samples have also collected…"
- "A shape peak…"
- "was calculation in…"
- "planer CuO4…"
- "the function of Cu substitution"
- "…could partly present valence of Mn distinctly. From Figure 5c, the shape of Mn pre-edge shows broad and weak which represents that…"
- "desodium"

etc…

• The manuscript contains a lot of experimental data from multiple techniques covering various lengthscales, sometimes combined with theoretical calculations. The data provide evidence of different behaviours of NMMTO, NCMMTO, and NLCMMTO. This is factually underlined by the author at the end of the introduction (line 81-82): "The introduction of Li plays an important role in strengthening the structural framework and preserving mechanical integrity". The reported difference of electrochemical behaviour, and subsequent structural and chemical properties between NLCMMTO and NCMMTO is puzzling. Nevertheless, it is rather disappointing that the authors do not provide an explanation for the effect induced by Li incorporation in the structure on the material properties. This is a major weakness of the manuscript.

• The Mn-K edge of NMMTO in Fig. 1c is different compared to NLCMMTO and NCMMTO. The peak at ~6562 eV is much less intense, so is the peak at ~6575 eV, and the ratio of the two peaks seems to differ from the other compounds, contrary to what is mentioned in the text (line 102-104). This is in apparent contradiction with the Mn-L23 edge shown in Fig. 1n. Please comment.

• The 4D stem data of the P2 and O3 structures leads to electric field and charge density distributions that are visually different. It is surprising that the authors do not point this out. Specifically, the values of the charge density are much higher in O3 than P2, such difference seems unrealistic.
Furthermore, the authors claim (line 177-180) "The results indicate that, regardless of whether it is the P2 or O3 phase, there

is no significant variation in the electric field intensity within the oxygen atom layers. This suggests that the Coulombic interactions between the oxide layers are relatively uniform." It would be useful to overlay the atomic structure on the maps in Fig. 2f-m. The causal link between the two statements is also not obvious and should be explained further.

• From Fig. S22, the authors indicate that (line 206-207) The sodium ions all show the characteristics of three-dimensional diffusion… It is unclear why a planar geometry of the diffusion paths of Na ions shown in this figure is described as three-dimensional, as opposed to 2D.

• For all the XRD maps in Fig. 3, and in SI, please indicate the meaning of the vertical direction. One could guess that it corresponds to charging from the bottom, up to 4.15 V in the middle, then discharging up to the top, but this should be clearly indicated. In SI, "intensity" should not be indicated as the vertical axis label in the same type of maps since these are contour plots.

• The discussion of XRD peak positions from Fig. 3 maps and SI would benefit from maps over a narrower range of 2theta values to emphasize the variations of peaks. Thus far, the shifts are sometimes difficult to visualize. Furthermore, the data of the (104) peak could be included in the main text.

• Fig. 4i is not discussed at all in the text. What is its purpose?

• The O-K ELNES in Fig. 4 is discussed line 259-261: "Such a broad 3d peak caused by an integration of the octahedral crystal field, exchange and the Jahn-Teller distorti00on from MnO6 has been maintained during the whole charging and discharging process in NMMTO demonstrating the valence change is Mn3+/Mn4+." The meaning of this sentence and the rationale behind the demonstration of valence change is difficult to comprehend. Please clarify.

• In Fig. 5, atomic resolution HAADF images are shown at two charge states. Interplanar distances of 0.55-0.56 and 0.54 nm are indicated and mentioned later in the text. How meaningful are these values given that the cationic planes appear fuzzy and not as straight lines. It seems that these values are within the error.

• The Mn-K and Ni-K edges in Fig. 5 are difficult to visualize for all states of charge given that they overlap, which makes the description of the results line 307-337 difficult to follow. Please make sure that all spectra are visible.

• Line 332, it is indicated that the NLCMMTO exhibits an unexpected reduction of Mn from the +4 (OCV) to the +3(C-3.5V) upon charging to 3.5 V… Is it a typo for +2 valence at 3.5 V? The Mn-L3 edge in Fig. 5f shows a rather typical Mn 2+ fine structures at 3.5 V, which is also indicated line 268.

• The Mn-K edge shown in Fig. 5 shows limited potential to be interpreted in terms of valence and structural change. The Ni-K edge is better in this respect, but XPS is provided. It is surprising that the authors have not included data for the SXR Ni-L23 edge and XPS Mn 2p (was it recorded?), which would be useful to discuss the valence changes, complementarily to SXR Mn-L23, XPS Ni 2p and the XAS K edges, and support the discussion about the participation of Mn and Ni in the charge compensation mechanism.

• Line 341: "This shift partially reverses after discharging to 2.5 V, suggesting a quasi-reversible Ni redox process." The reversal is clearly partial and therefore the redox process cannot hardly be called quasi-reversible…

• The discussion about the DOS leads to the statement "the reduction activity of Ni ions is greater than that of Mn ions." I miss a justification for this interpretation from the DOS presented.

• Fig. 6c: the fine structure at ~8335 eV is not present in the Ni-K edges shown in Fig. 5. Why such difference?

• How is the oxidation state quantified from the Ni-K edge in Fig. 6 (line 363)?

• The link between covalency and bond strength could be explicited further (line 370).

Minor comments:
• Line 270: "…shows the oxygen going through structural change". The material can undergo structural changes, but not the oxygen atoms. Please rephrase.

• Line 274: the electron is more delocalized…" The electrons (or the electronic density) are more delocalized.

• In the schematic in Fig. 6h, a proper description of the corresponding states of charge is necessary to comprehend the meaning of the schematic.

• The colors of spectra in insets of Mn-K and Ni-K in Fig. 6 are difficult to distinguish. Maybe the dashes should be kept instead of full lines, and arrows indicating the shift of the spectra upon charging / discharging could be added.

Version 1:

Reviewer comments:

Reviewer #1

(Remarks to the Author)
My concerns have been well addressed, and I now recommend its publication.

Reviewer #2

(Remarks to the Author)
I appreciate the authors' detailed responses. Overall, most of my concerns have been addressed, particularly the more cautious interpretation of the O–O dimer and Ni–O–Mn superexchange mechanisms, as well as the additional discussion of Cu, Li, Ti, and configurational entropy.

However, the response to the role of the O3 phase and the P2/O3 intergrowth region remains only partially satisfactory. The added 4D-STEM phase and charge maps suggest local electrostatic/structural differences between P2 and O3 domains, but they do not directly demonstrate that the O3 phase provides mechanical support. Likewise, charge redistribution at the P2/O3 interface may indicate interfacial activity, but it is still indirect evidence for stress accommodation or enhanced Na^+ transport.

The statement that the higher O3 fraction in NLCMMTO leads to improved long-term cycling stability is also not fully justified, because Li incorporation simultaneously affects the O3 fraction, oxygen-redox behavior, local electronic structure, and charge redistribution. Therefore, the role of the O3 phase should be described more cautiously.

I suggest that the authors either provide quantitative P2/O3 phase fractions with uncertainties and correlate them with lattice strain parameters such as $\Delta c/c$, $\Delta V/V$, or strain anisotropy, or explicitly acknowledge that the precise contribution of the O3 phase remains unresolved. In the absence of such evidence, the P2/O3 interface should be described as a possible contributor to stress accommodation and Na^+ transport, rather than as a demonstrated origin of the improved cycling stability.

Reviewer #3

(Remarks to the Author)
I thank the authors for providing detailed answers to my comments. Several aspects of the study have been clarified in the revised version. Even though most of my comments have been adressed convincingly, some answers require further clarifications, as detailed below.

• Comment 3)

The authors provide an explanation for the Mn-K XANES intensity difference between NMMTO and the two other compounds NLCMMTO and NCMMTO. I suggest the authors consider including the proof of shortened Mn-O bond length (Figure R17 a) in the revised manuscript or at least in SI.
As it stands, the current modification to the revised manuscript is limited to changing "the well overlapped spectrum " to "the nearly identical spectra", which is a very light modification.

• Comment 4)

Here as well, the data regarding the specimen thickness, shown in the answer, would be of interest to the reader, at least in SI.

• Comment 9)

The spectra for NLCMMTO in the revised Figure 4c are drastically different of those in the original Figure 4a. Why? Have the data been acquired again?
Furthermore, various statements in the answer to this comment raise further questions.
First, referring to Figure R27a the peak at 529 eV in the O-K edge of NMMTO seems to disappear during the charging process, which is opposite to the authors' answer.
Second, the authors indicate that this remaining pre-peak at 529 eV of the O-K edge and the Mn-L edges shown in Figure R27b suggest that the Mn valence state shows negligible variation during cycling. This also appears very unlikely from the spectra given in Figure R27. A clear change of spectral weight is apparent in the Mn-L3 edge upon cycling, with a drastic red-shift at C-2.5 giving a Mn-L23 edge characteristic of low valence (~2+). Local geometrical effects, such as distortion, bond lengths, etc., can modify near-edge fine structures at L23 edges of transition metals. However, associating the spectral shifts in Figure R27b to a negligible variation of the Mn valence is surprising and requires solid proof (through e.g. multiplet calculations).

• Comment 10)

The authors indicate that the HAADF analysis is not used as a standalone quantitative method for determining subtle lattice changes. They agree that intrinsic uncertainty is is associated with directly measuring interplanar distances from these images. My initial question was about this uncertainty: how precise are the values 0.54, 0.55 and 0.56 nm? Thus far, the text has not been changed, and the statement (p. 324-326) does not reflect the uncertainty associated with such measurements: "[…] the interlayer distance still maintains 0.55 nm as pristine state, otherwise the distance could be enlarged to 0.56 nm after extraction Na+. When charging to 4.15V, the distance reduces to 0.54 nm without TM slabs sliding or disordering." Such statement does not indicate nor suggest any uncertainty to these values, which is misleading.

• Comment 12)

I disagree with the answer provided by the authors here.
Subtle effects like mixed-valence variations at the Mn-L23 XANES spectra of a complex structure like NLCMMTO cannot be interpreted solely by fingerprinting standard references like MnO, Mn2O3 and MnO2. Matching the energy of peaks in NLCMMTO with these standards does not mean that Mn2+, 3+ and 4+ are present in NLCMMTO. For example, pure Mn2+ compounds like MnO also have a Mn L-edge with intensity where the 3+ and 4+ "reference" peaks are marked, but it does not make MnO a mixed valence system.

The main spectral weight of the spectrum can be linked to the valence of Mn. But this is not the case for all the peaks and shoulders, which are associated with both the valence and local geometry effects. To discriminate between valence and geometry effects, multi-electronic calculations (e.g. multiplet, etc.) will provide a solid and quantitative answer, but not comparison with standards (with different local geometries and chemistries). Therefore, from the evidence provided and without appropriate calculations, only the overall valence and its trend could be mentioned. The Mn valence seems to go from an overall 4+ (OCV and -4.15) to 2+ (C-3.5 and C-2.5). Other valences cannot be excluded but cannot be supported either.

As it stands, the following statement is not supported: "[…] to the mixed valence of +2/+3/+4 upon charging to 3.5 V, which [...]".

• Comment 15)

I thank the authors for their answer. Maybe the point could be made for the reader, with a clear mention that "the DOS near the Fermi level reflects the availability of electronically accessible states that can participate in charge compensation."

Version 2:

Reviewer comments:

Reviewer #2

(Remarks to the Author)
The authors have adequately addressed my remaining concern. Although the additional stress calculation does not fully isolate the independent contribution of the O3 phase, the revised manuscript now presents the role of the P2/O3 intergrowth region more cautiously and appropriately describes it as a possible contributor to stress accommodation rather than as a demonstrated origin of improved cycling stability. Therefore, I am satisfied with the revision and recommend acceptance, subject to minor editorial checks such as figure numbering and language polishing. For example, the stress calculation is referred to as Supplementary Figure S37 in the response letter, whereas it appears as Figure S36 in the revised manuscript and Supporting Information. In addition, several Figure 2 panel references appear inconsistent with the figure caption, especially the assignments of the electric-field and charge-density maps. The caption of Figure R5 in the response letter should also be corrected, as it appears to describe O K-edge XAS rather than the HAADF-STEM/line-profile analysis shown.

Reviewer #3

(Remarks to the Author)
The authors have addressed all of my concerns. I now recommend this work for publication in Nature Communications.

Open Access This Peer Review File is licensed under a Creative Commons Attribution 4.0 International License, which permits use, sharing, adaptation, distribution and reproduction in any medium or format, as long as you give appropriate credit to the original author(s) and the source, provide a link to the Creative Commons license, and indicate if changes were made.
In cases where reviewers are anonymous, credit should be given to 'Anonymous Referee' and the source.
The images or other third party material in this Peer Review File are included in the article's Creative Commons license, unless indicated otherwise in a credit line to the material. If material is not included in the article's Creative Commons license and your intended use is not permitted by statutory regulation or exceeds the permitted use, you will need to obtain permission directly from the copyright holder.
To view a copy of this license, visit https://creativecommons.org/licenses/by/4.0/

Reviewer #1 (Remarks to the Author):

This manuscript reports the design of a layered oxide cathode, $Na_{2/3}Ni_{1/6}Li_{1/18}Cu_{1/18}Mg_{1/18}Mn_{1/3}Ti_{1/3}O_2$, which exhibits stable electrochemical performance over prolonged cycling. The authors attribute the enhanced cycling stability to a self-adaptive volume regulation mechanism driven by charge reconfiguration, supported by a set of in situ and ex situ characterizations. They propose that Ni-O-Mn interactions play a critical role in mitigating stress accumulation and maintaining structural stability during cycling. In situ synchrotron XRD reveals unusual changes in the interlayer spacing, while 4D-STEM provides direct experimental evidence supporting this atypical structural evolution. Furthermore, the authors employ PDF analysis, XPS, and theoretical calculations to establish a mechanistic link between Ni-O_{2n}^{-}-Mn superexchange interactions, stress redistribution, and long-term electrochemical stability. The role of each constituent element is systematically investigated, and the unconventional charge compensation mechanism is directly observed by in situ XAS. Overall, this work proposes a cathode chemistry based on earth-abundant transition metals and demonstrates durability under high current densities, which is important for the development of large-scale sodium-ion batteries.

Taken together, the intriguing evolution of interlayer spacing combined with the detailed mechanistic investigation makes this work potentially suitable for Nature Communications. However, several aspects related to phrasing, clarity, and presentation require further strengthening before the manuscript can fully meet the standards of this journal. Detailed comments are provided below.

Response: Thanks for your careful and comprehensive assessment of our manuscript and for recognizing the conceptual interest, methodological depth, and potential relevance of this work. We believe that the following responses adequately meet your expectations. Detailed point-by-point responses to the specific comments are provided below.

1. The idea that adjustable repulsive force of O-O caused by charge reconfiguration leading to self-adaptive volume behaviour is innovative. Some researchers argue that

the reduction of distance of TM layers at the high voltage might be consequence of the incorporation of Cu, what do authors think of this explanation, please discuss with this viewpoint.

Response: Thanks for your comment. Some studies suggest that the oxidation of Cu^{2+} to Cu^{3+} can alter the electronic distribution of Na-O-Cu, thereby reducing O-O repulsion and leading to changes in the interlayer spacing. However, based on the electrochemical behavior of our material, no obvious oxidation state change of Cu is observed. In addition, from **Figure R1** there is no noticeable shift being detected in the XAS spectra of Cu at different states of charge, indicating that Cu valence variation is not the origin of the change in O-O repulsion in our material.

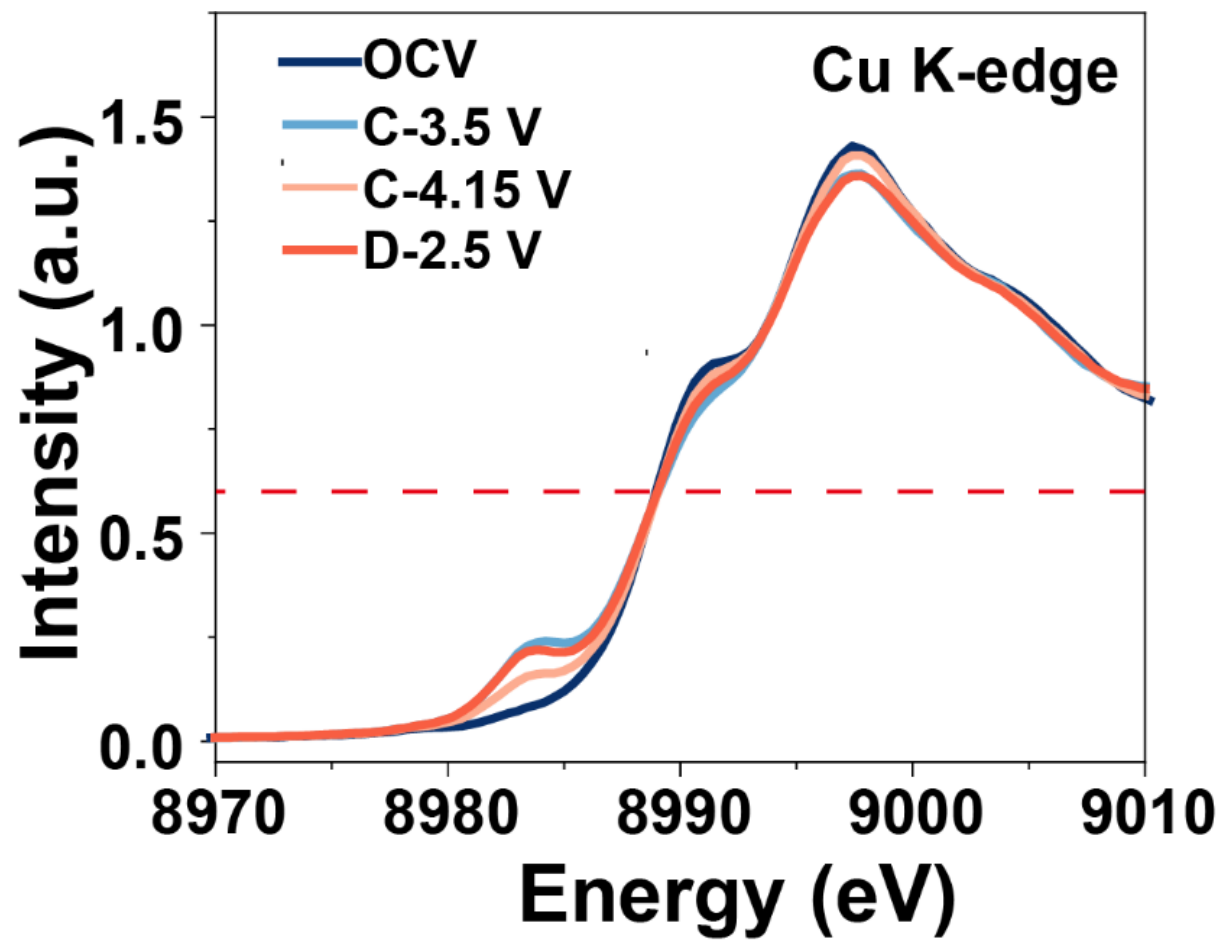

Figure R1 (Figure 4g) Cu K-edge XANES in NLCMMTO at different charge state.

However, the in-situ synchrotron based XRD reveals that, during charge and discharge, not only the (00l) reflections which are associated with adaptive adjustments of the TM layers, but also the (104) reflection exhibits a similar evolution shown in **Figure R2**. As the (104) plane involves both transition metal and oxygen atoms, this observation indicates that oxygen participates in regulating the interlayer spacing. Notably, at high voltage, a reduction in repulsive interactions leads to a contraction of the corresponding lattice spacing. This behavior cannot be explained solely by the redox activity of Cu. The similar evolution of the (104) and (00l) spacings reflects the coupled nature of interlayer and intralayer structural responses.

Thus, we believe that this behavior arises from the charge reconfiguration of intralayer oxygen and TM ions after introduction of Cu.

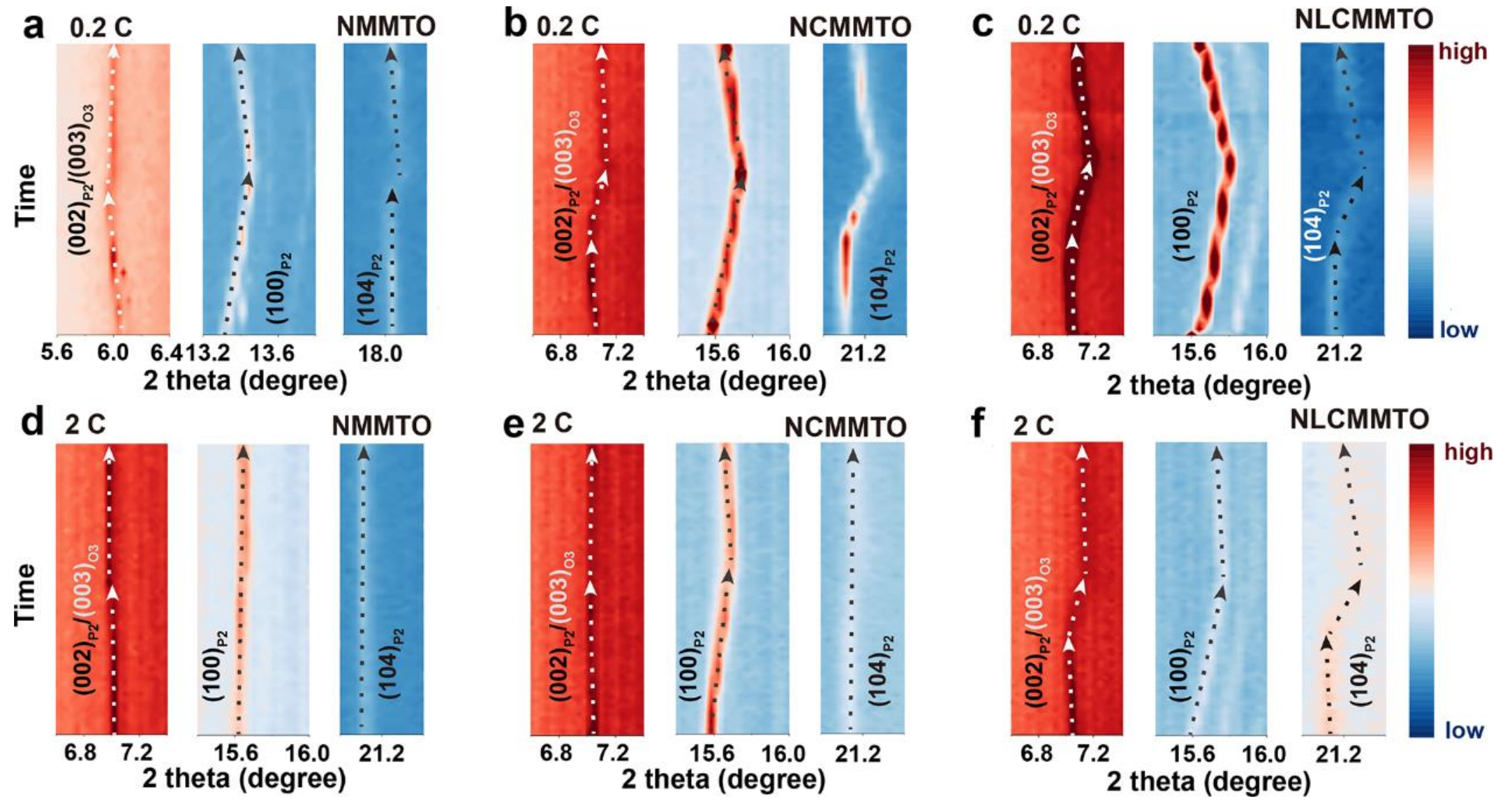

Figure R2 (Figure 1b-1g) a-c, selected in-situ synchrotron-based XRD pattern collected during the first charge/discharge in the voltage range of 2-4.15 V at 0.2C of NMMTO, NCMMTO, and NLCMMTO. **d-f**, selected in-situ synchrotron-based XRD pattern collected during the first charge/discharge in the voltage range of 2-4.15 V at 2C of NMMTO, NCMMTO, and NLCMMTO.

2. It is impressive by the depth of the authors' investigation into the role of Cu, which is both scientifically significant and thought-provoking. To further strengthen the clarity of the overall mechanism, the authors might consider expanding the explanation regarding Li to more clearly relate the function of each element to the enhanced electrochemical performance. This addition would undoubtedly strengthen the manuscript.

Response: Thanks for your valuable suggestions. Electrochemical performance most directly reflects the function of Li. It can be seen from **Figure R3a** that NLCMMTO exhibits higher specific capacity and better stability. To better understand the role of Li, soft X-ray absorption spectroscopy (SXR) was employed to the electrodes of NCMMTO and NLCMMTO at different states of charge. As shown in Figure R3b, NCMMTO and NLCMMTO exhibit a characteristic feature at ~533.6 eV when charged to 3.5 V, which is attributed to delocalized O 2p states associated with enhanced TM-O

hybridization,[1] likely arising from the formation of Ni-O-Mn framework, in which electron holes are distributed over the TM-O network rather than localized on specific sites.[2] Upon further charging, NLCMMTO retains this feature, indicating that the electron holes remain delocalized within the lattice. In contrast, the corresponding peak in NCMMTO gradually disappears and evolves into a higher energy shoulder at ~534.9 eV in **Figure R3c**, suggesting further oxidation of lattice oxygen accompanied by localized oxygen interactions which might be harmful to the structure integrity and Na^+ transfer kinetics due to the irreversibility. Such distinct behavior highlights the critical role of Li incorporation.

Specifically, such delocalized O 2p states could act as a "charge buffer" within the TM-O network. This delocalization reduces the localization of electrons on transition metal (Ni Mn) d orbitals, thereby alleviating orbital degeneracy and suppressing Jahn-Teller distortions, which could be helpful to stabilize the structural integrity, which is confirmed by the higher capacity retention.

Additionally, the more symmetric TM-O environment caused by reduction of Jahn-Teller distortions, increasing the energetic barrier for O-O bond formation that can hinder Na^+ diffusion by inducing local structural distortion, blocking diffusion pathways, and increasing migration barriers. Consequently, the evolution toward localized O-O σ* configurations at high states of charge is suppressed, leading to improved reversibility and kinetics of the oxygen participation process. Thus, the GITT tests were employed to measure the kinetics of two samples shown in **Figure R3d** and **R3e**. It can be observed that at the highest voltage of 4.15V, the NLCMMTO exhibits higher Na^+ diffusion coefficient showing its superior ionic conductivity and kinetic properties. The result is consistent with the SXR spectra.

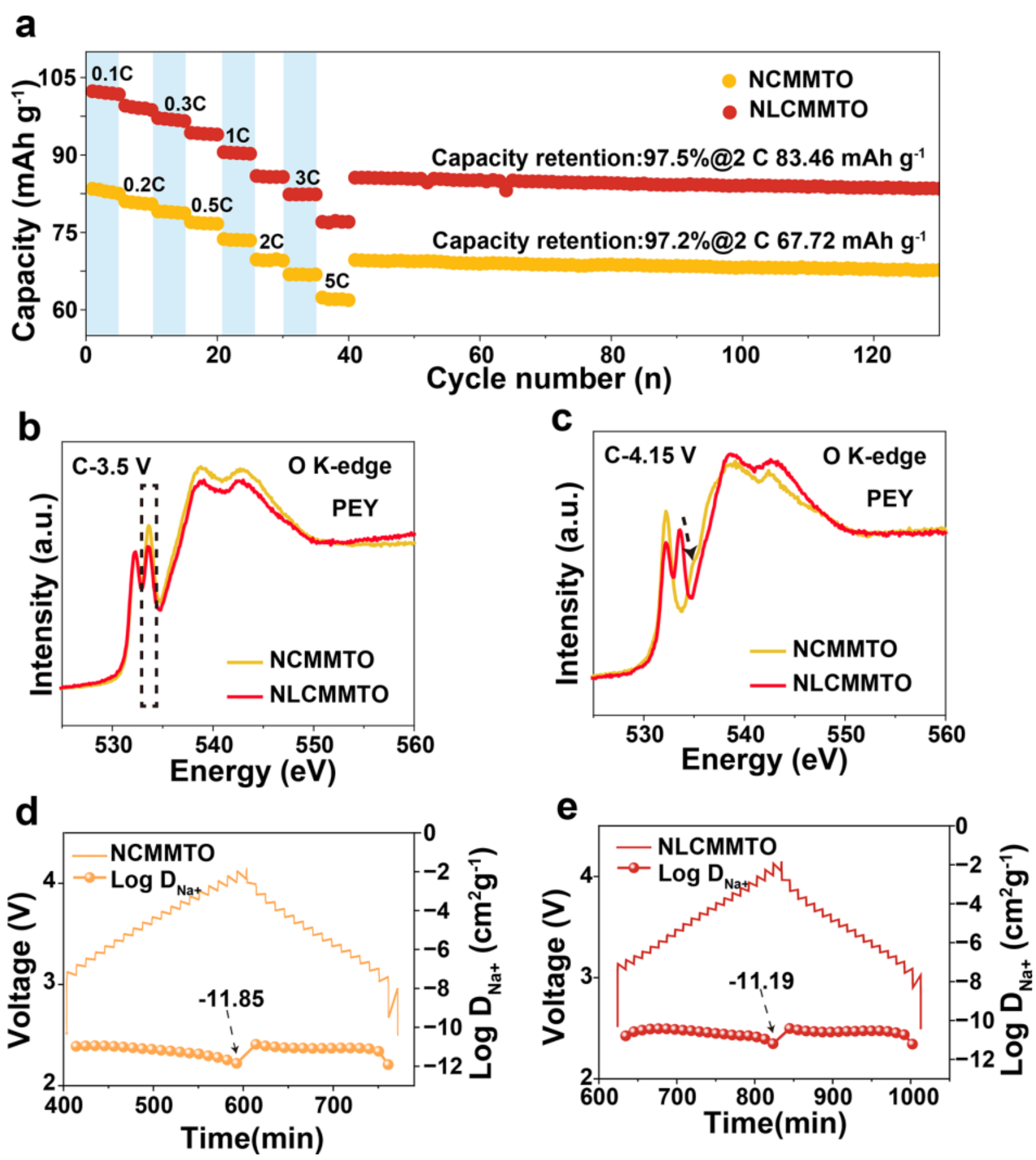

Figure R3 (Figure 4j, 4k, S21) a, electrochemical performance of NCMMTO and NLCMMTO. **b,c** O K-edge XAS of NCMMTO and NLCMMTO electrodes charging at 3.5V and 4.15V**. d,e** GITT results of NCMMTO and NLCMMTO**.**

3. The structural evolution during charging and discharging was tracked by in-situ synchrotron XRD offering rich and important information on microstructural changes of the material in this draft. In especial, the real-time tracking of structural changes under high current densities was also be studied, which offer valuable design principles for commercially viable cathode materials capable of fast charging and discharging. Thus, authors should clarify the organic connection between structure characterization and electrochemical performance improvement and be more sharply articulated in the discussion.

Response: Thanks for your valuable suggestions. We agree that microstructural characteristics are intimately linked to the observed macroscopic electrochemical

performance, and this relationship should be further strengthened in the discussion section to better present the practical implications of our work.

On the one hand, we further clarify how the structural evolution revealed by in situ synchrotron XRD correlates with the electrochemical behavior in the revised manuscript. Specifically, we emphasize that due to the self-adaptive behavior of the material, stress accumulation during repeated charge and discharge processes is effectively reduced, which largely preserves the structural stability under long-term cycling and consequently improves the capacity retention over prolonged cycles.

On the other hand, the fast charge compensation between Ni and Mn observed by in-situ XAS together with fast Na^+ transport kinetics enables rapid charge and discharge under high rate and large current densities, allowing the material to maintain stable electrochemical performance and high capacity even at high currents. This behavior highlights its strong potential for application in electronic devices requiring fast charging and discharging. This strengthened structure property relationship also could guide principle for the rational design of commercially viable cathode materials capable of high-rate operation.

We have revised and marked yellow the modification in the revised manuscript, please see lines 256-258.

4. The mechanism of charge compensation was directly observed by in-situ XAS, which is interesting and offers important scientific insights and serves as a guiding reference for other experiments seeking to probe the dynamic behavior of various elements. But it is not clear how the in-situ XAS measurements have been made. More experimental details on parameters are needed.

Response: Thanks for your comment. We sincerely thank the reviewer for pointing out that the description of the in-situ XAS measurements was insufficient. We agree that additional experimental details are necessary to ensure clarity and reproducibility. In the revised manuscript, we have now expanded the Experimental Section to provide a clearer description of the operando cell configuration and measurement parameters.

Synchrotron radiation X-ray absorption spectroscopy (XAS) measurements were conducted at the wiggler XAS Beamline (12ID) at the Australian Synchrotron in

Melbourne, using liquid N_2-cooled Si(111) monochromator crystals. In-situ XAS experiments were performed in transmission mode using a modified CR2032 coin cell configuration. Circular apertures 5 mm diameter were punched through both sides of the stainless-steel casing and sealed with Kapton film to provide X-ray transparent windows. The cathode composite was positioned within the X-ray beam path to enable direct probing of the active material during electrochemical cycling. The cell was galvanostatically cycled between 2.5-4.15V at a rate of 2C using a battery testing system LANDdt. XANES spectra at the Mn and Ni K-edges were collected at selected states of charge and discharge during the first cycle. Each scan required approximately 30 minutes for acquisition. Energy calibration was performed using reference metal foils measured simultaneously. No significant beam-induced spectral changes were observed during repeated scans. These additional details have now been included in the revised manuscript (Experimental Section lines 460-465). We appreciate the reviewer's comment, which has helped us improve the transparency and completeness of the experimental description.

5. To make it clearer for readers, some subtitles and figure captions should be described more accurately. For example, the Fig 6h, "adaptive stress distribution driven by charge reconfiguration diagram" didn't show the information on adaptive stress distribution; instead, it would be better to be described with "the fast charge transfer between the TM ions".

Response: Thanks for your invaluable advice. We agree that clearer and more precise subtitles and figure captions will improve the readability of the manuscript and help readers better understand the underlying physical meaning of the figures. In the draft manuscript, Figure 6h was titled "adaptive stress distribution driven by charge reconfiguration diagram" to qualitatively illustrate the coupling between charge redistribution and stress regulation. However, we acknowledge that this description may be too abstract and does not explicitly convey the key message of the schematic.

As correctly pointed out by the revised version, Figure 6h primarily emphasizes the rapid charge transfer and redistribution among transition metal ions, which underlies the adaptive mechanical response rather than directly visualizing stress

distribution itself. To avoid potential confusion and to make the figure more intuitive for readers, we have revised the subtitles and figure captions to better reflect the physical process illustrated in the diagram.

Similar refinements have also been applied to other related subtitles and figure captions throughout the manuscript where appropriate.

6. The manuscript is figure-heavy and risks overwhelming readers. Consolidation of adding some clearer mechanistic schematics would improve readability.

Response: Thanks for your comment. We have carefully revisited the organization and visual balance of the main figures with the aim of improving readability and reducing potential information overload. Specifically, we have reorganized the panels to streamline the presentation and improve the logical flow of the data.

Importantly, a mechanistic schematic has now been incorporated into revised **Figure R4l** to provide a clearer conceptual framework for interpreting the experimental results. This addition is intended to guide the reader through the key processes and relationships underlying the observed phenomena, thereby reducing the cognitive burden associated with interpreting multiple datasets.

More broadly, we have also refined figure layouts and adjusted the distribution of content between the main figures and the Supporting Information, ensuring that the central findings are presented in a more focused and accessible manner. We believe that these revisions have improved the clarity and readability of the manuscript, while preserving the completeness of the data presentation.

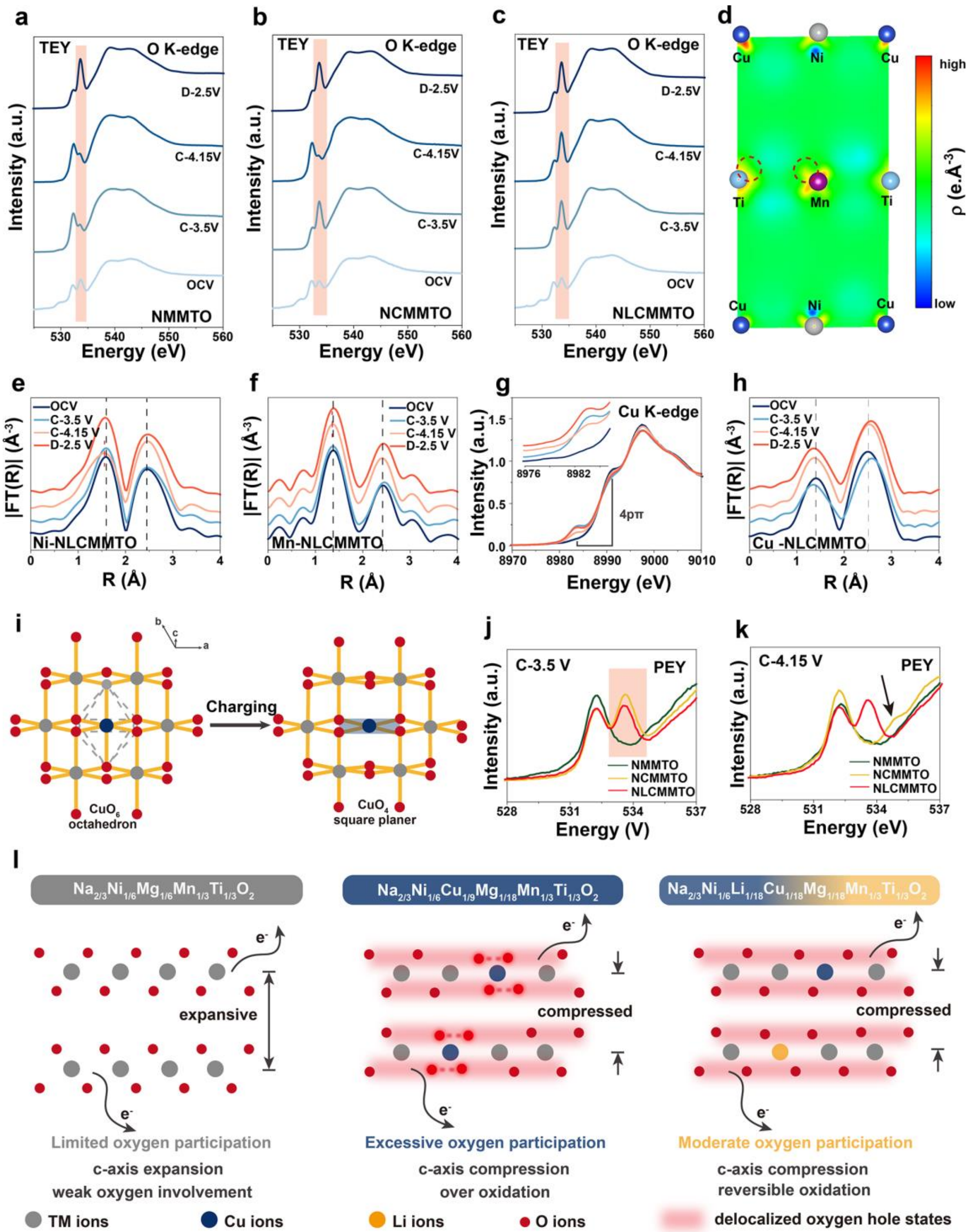

Figure R4 (Figure 4) TEY O K-edge XAS of **a,** NMMTO, **b,** NCMMTO, **c,** NLCMMTO electrodes from different charge states. **d,** Calculation of the charge density distribution of NCMMTO. **e,** Ni, **f,** Mn EXAFS in NLCMMTO at different charge state. **g,** Cu K-edge XANES in NLCMMTO at different charge state. **h,** Cu EXAFS in NLCMMTO at different charge state. **i,** Schematic image of structure evolution of Cu. PEY O K-edge XAS of NMMTO, NCMMTO, and NLCMMTO **j,** at

3.5V; **k,** at 4.15V. **l,** Schematic image of oxygen involved lattice evolution in layered oxides at the highest voltage (4.15V).

7. We observe that both ex-situ XAS and in-situ XAS are used in the study. Since both techniques can probe the K-edge of transition metal elements, what is the fundamental difference between them, and why did the authors conduct both experiments?

Response: Thanks for your comment. Although both ex-situ and in-situ XAS probe the K-edge of transition metals, the two techniques provide fundamentally different information and are therefore complementary rather than redundant.

Ex-situ XAS measurements are performed on electrodes extracted at selected states of charge, which allows for high signal-to-noise spectra and detailed quantitative analysis of oxidation states and local coordination environments. However, ex-situ measurements may be influenced by relaxation or partial charge redistribution during cell disassembly and sample handling.

In contrast, in-situ XAS probes the electrode under real electrochemical operating conditions, enabling direct observation of the continuous and dynamic evolution of the transition-metal electronic structure during Na extraction and insertion. This approach minimizes uncertainties associated with ex-situ sample preparation and allows identification of intermediate states that may not be preserved after cycling interruption. Therefore, we employed both techniques: ex-situ XAS to obtain high quality, quantitative information on the extent of transition metal redox, and in-situ XAS to verify that the observed redox processes indeed occur under operando conditions and to capture their evolution in real time. The combined use of ex-situ and in-situ XAS provides a more reliable and comprehensive understanding of the charge compensation mechanism in this system.

We greatly appreciate the reviewer's time and thoughtful feedback, which helped us improve both the presentation and the scientific content of our work.

Reviewer #2 (Remarks to the Author):

The manuscript presents a high-entropy layered oxide cathode (NLCMMTO) featuring "self-adaptive volume" behavior for Na-ion batteries, where Cu-driven O-O dimer formation and Ni-O-Mn superexchange are proposed to enhance stress management and cycling stability. The work leverages advanced characterization and aims to move beyond traditional high-entropy concepts by targeting specific electronic interactions. While the study is innovative and well-supported experimentally, strengthening the quantitative connection between mechanistic claims, structure-property relationships, and stress analysis would further clarify the manuscript's novelty and significance. Please see the below comments.

Response: We sincerely thank the reviewer for the overall positive evaluation of our work and for recognizing its novelty, which has helped us to further improve the clarity and quality of the manuscript. To better address your comments and further enrich the manuscript, we have added SXR data and quantitative analyses clarifying the roles of Li, refined several terms to avoid ambiguity and improve the rigor of the article meanwhile highlighted the originality of this work. With these revisions, we believe that the clarity of the mechanistic understanding, the robustness of data support, and the presentation of the originality of manuscript have all been significantly improved. Detailed point-by-point responses are provided below.

1. The long term cycling performance (82% capacity retention after 1000 cycles at 2C, from 88.6 to 72.8 mAh g^{-1}) is impressive in terms of stability, but the absolute capacity seems to be modest compared with recent state of the art layered Na cathodes in a similar voltage window. So. please place the performance of NLCMMTO in a more quantitative context by comparing specific capacity, average operating voltage, and energy density with relevant recent O3/P2 and high entropy Na layered oxides (including the works already cited in Refs. 1-4, 12, 18, 21, etc.). Also it would be helpful if the authors could calculate the theoretical capacity of NLCMMTO and discuss the fraction of capacity achieved at 0.1C and 2C.

Response: Thanks for your helpful suggestions. The theoretical capacity of NLCMMTO can be estimated based on the number of electrochemically active Na^{+} that

can be reversibly extracted from the layered structure. Considering that all samples in this manuscript use the P2 phase as the model for element substitution, the theoretical capacity of the P2 phase is therefore used to estimate the theoretical capacities of the series of synthesized materials. In general, P2 phase layered oxides with formula of $Na_{2/3}TMO_2$, not all Na^+ participate in reversible electrochemical reactions within the practical voltage window. Previous studies have shown that a portion of Na^+ remains in the structure to stabilize the layered framework and does not contribute to the reversible capacity. It is widely accepted that approximately 1/2 of Na^+ are electrochemically active in many P2-type cathodes within the voltage window of 2.5-4.15V (Wang et al., Sci. Adv., 2018, 4, eaar6018). Thus, NLCMMTO theoretical capacity is 92.4 mAh g^{-1}. The calculation is given by the following:

$$C_{theoretical} = \frac{n \cdot F}{3.6 \cdot M} = 92.4$$

where n represents transferred electrons, F represents Faraday constant (96485 C·mol^{-1}), M represents molecular weight (~96.6 g mol^{-1}). Accordingly, the experimentally achieved discharge capacity of 104 mAh g^{-1} at 0.1C corresponding to ~112% of the theoretical capacity. The higher specific capacity might originate from the additional compensation mechanism of reversible oxygen redox in Ni-O-Mn framework. In NLCMMTO, the stabilization of delocalized O 2p states enables reversible continuous electron extraction from the oxygen band, promoting additional Na insertion and extraction. As summarized in Table R1, while the capacity is moderate owing to the relatively narrow work voltage window, NLCMMTO demonstrates remarkable cycling stability, maintaining stable performance over 1000 cycles.

Table R1 Specific capacity comparison of different layered oxide cathodes

Sample	Phase	Specific capacity (mAh g^{-1})	Work voltage (V)	Ref.
$Na[Mn_{1/3}Fe_{1/3}Ni_{1/3}]O_2/NaCaPO_4$	O3@P2	148.2@0.1C 124@2C	2.0-4.2	3
$Na_{5/6}Li_{2/27}Ni_{8/27}Mn_{11/27}Ti_{6/27}O_2$	O3	152@0.1C 120@2C	2.0-4.4	4
$Na_{0.9}Ni_{0.32}Zn_{0.08}Fe_{0.1}Mn_{0.3}Ti_{0.2}O_2$	O3	144.9@at 5 mA g^{-1}	2.0-4.0	5
$Na_{2/3}Ni_{1/3}Mn_{1/3}Ti_{1/3}O_2$	P2	88@0.1C	2.5-4.15	6
$Na_{0.63}Mn_{0.4}Co_{0.15}Mg_{0.15}Ti_{0.1}Ru_{0.1}Cu_{0.05}Ca_{0.05}O_2$	P2	180.6@0.1C	1.5-4.5	7
$Na_{2/3}Ni_{1/6}Li_{1/18}Cu_{1/18}Mg_{1/18}Mn_{1/3}Ti_{1/3}O_2$	P2/O3	104@0.1C	2.5-4.15	This work

2. I think a key novelty of the manuscript is the claim that Cu substitution promotes O-O dimer formation, which then drives charge reconfiguration and self adaptive volume behavior. The main experimental evidence seems to be the O 1s XPS feature assigned to M-(O-O) species at C 3.5 V (Fig. 4b), along with changes in O K edge spectra. While this is suggestive, O-O dimer assignments are often debated. So I suggest that authors should provide a clear justification for assigning this feature to O-O dimers, e.g., by: 1) Comparing to reference spectra of well defined peroxo/superoxo species or to previous reports (quantitatively, not only qualitatively). 2) Providing DFT derived O-O distances and charge analysis demonstrating peroxo/superoxo like species and reporting O-O bond lengths in the computed structures and clarifying whether they are in the typical range expected for peroxo like dimers. In addition, the Cu K edge XANES/EXAFS analysis (Fig. 4g-i) is used to support a transition from CuO_6 octahedra to planar CuO_4. To more deeply understand this phenomenon, I suggest author do quantify the Cu-O bond length changes upon charging (from EXAFS fitting) and report coordination numbers and clearly present the models used for the CuO_6 to CuO_4 conversion, and describe how this geometrical change makes O-O dimer creation possible. At the current version, the Cu evolution is described qualitatively; a more quantitative EXAFS analysis would significantly strengthen the claim that Cu is the active driver of O O dimer formation rather than a passive "filler."

Response: Thanks for your constructive suggestions. We agree that assigning XPS peak uniquely to peroxo or superoxo dimers could be controversial without direct structural confirmation. So, to further clarify the nature of the oxygen species, we incorporated additional discussion of the SXR results at the O K-edge of three samples. The SXR measurements provide direct insight into the evolution of oxygen electronic states during the charging process. As shown in **Figure R5**, the appearance of the feature around ~533.6 eV corresponds to delocalized O 2p states associated with enhanced TM-O hybridization in both NLCMMTO and NCMMTO, indicating the introduction of Cu indeed enhances the redox activity of oxygen and facilitates the formation of delocalized oxygen hole distributed over the TM-O framework rather than localized on specific oxygen sites. Notably, these features evolve systematically with the state of

charge and are consistently observed across different detection modes: partial electron yield (PEY), total electron yield (TEY), and total fluorescence yield (TFY), indicating that the electronic state changes originate from intrinsic lattice oxygen rather than surface side reactions.

Besides, to investigate the changes in the reactivity of O under different desodium states, we calculated the density of states plots of O in the P2 and O3 structures with different sodium contents shown in **Figure R6**. The data in the figures show that in the P2 and O3 structures, as the sodium content decreases, the 2p orbital band center of O gradually shifts upward, indicating that the redox activity of O is enhanced.

Additionally, based on the reviewer's advice, we have expanded the EXAFS analysis to more clearly describe the structural change of the Cu coordination environment during charging in **Figure R7**. EXAFS fitting was performed in R space over the range of 1-3 Å, focusing on the first coordination shell corresponding to the Cu-O bond and offering information on coordination number (N) and bond distance. The fitting results indicate a reduction in the average Cu-O coordination number from 2.05 to 1.25, and the contraction of the in-plane Cu-O bond distance from 1.96 Å to 1.95 Å, which is consistent with the evolution from an octahedral CuO_6 environment toward a more planar CuO_4-like configuration. And then, when the Cu coordination geometry evolves toward a planar configuration, the oxygen atoms coordinated to Cu experience changes in orbital overlap and local electrostatic environment. These changes promote stronger TM-O hybridization and enable oxygen to act as an active electronic bridge between neighboring transition metal sites. As a result, oxygen participates directly in the charge compensation process and forms delocalized oxygen cluster like states, which in turn contribute to the observed self-adaptive structural response of the material.

According to the reminder of reviewers and after our careful consideration, to avoid overinterpreting the spectroscopic signatures as direct evidence of O-O dimers, we have updated throughout the manuscript to describe the observed oxygen species as delocalized oxygen clusters. This revised interpretation is supported by the combined evidence from O K-edge SXR measurements and DFT calculations, both of which

demonstrate strong oxygen redox activity and the involvement of oxygen in mediating charge redistribution. We believe this more conservative description more accurately reflects the available evidence while still supporting the central conclusion of the manuscript: that Cu substitution promotes oxygen-mediated charge reconfiguration, which contributes to the unique self-adaptive volume behavior of the material.

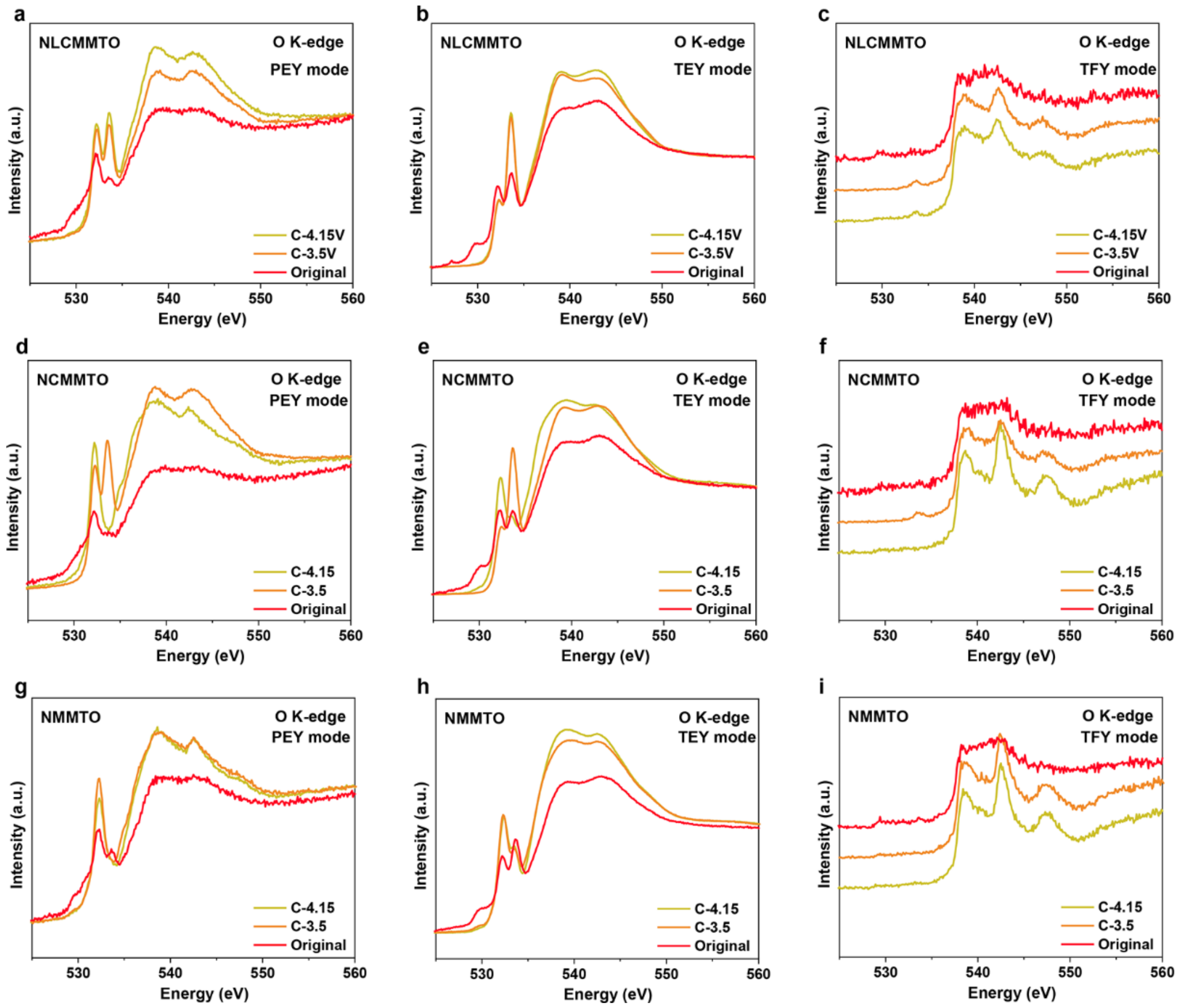

Figure R5 a-c, PEY, TEY, and TFY O K-edge XAS spectra of NLCMMTO electrodes collected in different states of charging. **d-f,** PEY, TEY, and TFY O K-edge XAS spectra of NCMMTO electrodes collected in different states of charging. **g-i,** PEY, TEY, and TFY O K-edge XAS spectra of NMMTO electrodes collected in different states of charging.

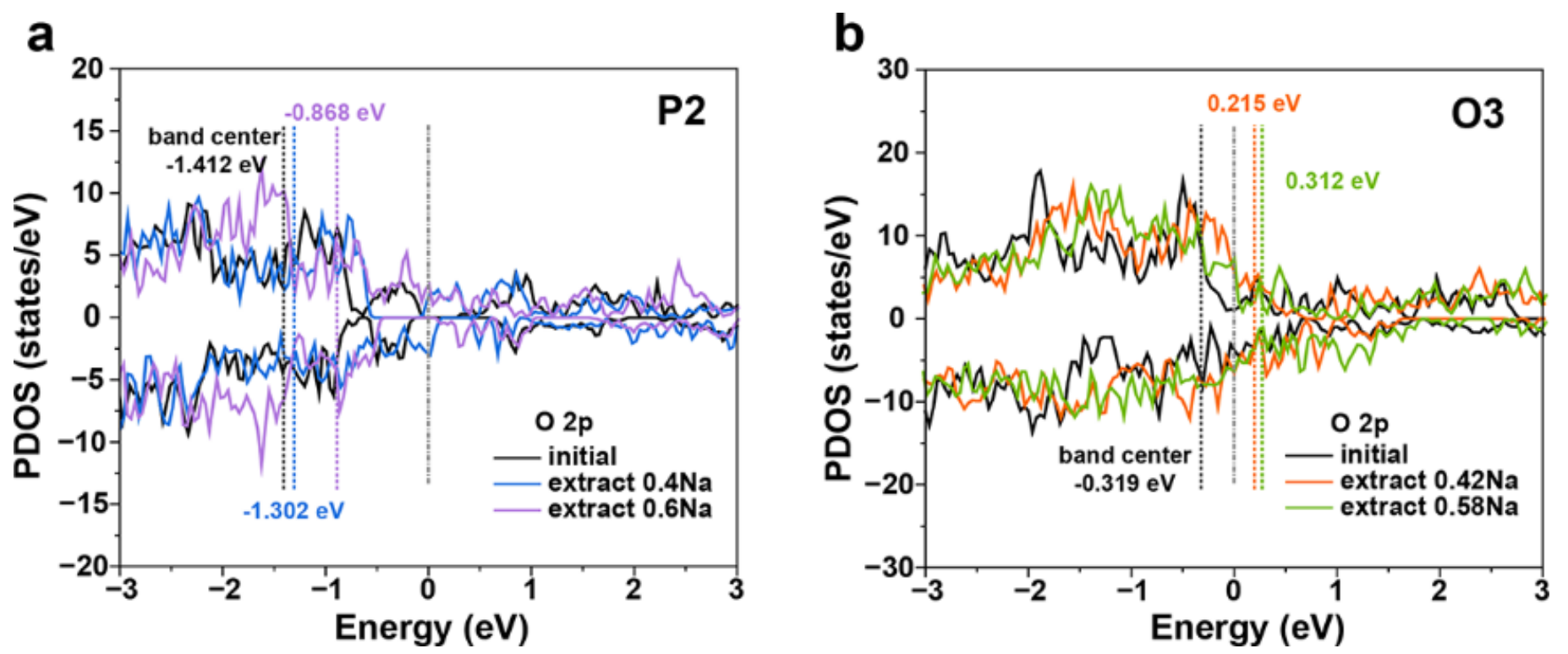

Figure R6 Density of states and band center diagram of O in **a,** P2 and **b,** O3 structures.

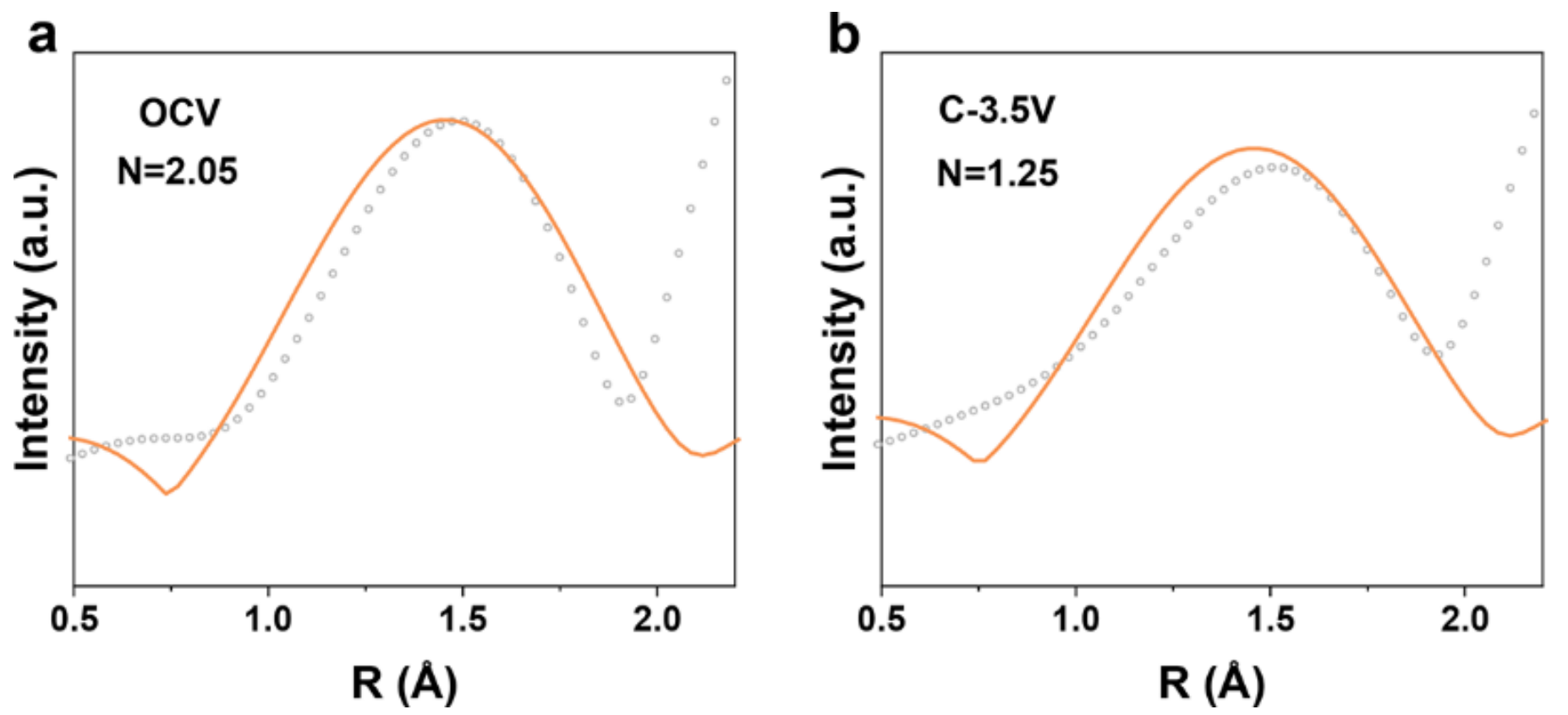

Figure R7 XAS refinement of Cu K-edge for NLCMMTO electrode at **a,** original state. **b,** charging to 3.5V.

3. NLCMMTO introduces both Cu and Li relative to NMMTO, and Cu relative to NMMTO in NCMMTO. To demonstrate that Cu is the key element responsible for O-O dimers and the related self adaptive behavior, the roles of Cu and Li need to be more carefully disentangled. For example, can the authors show that NCMMTO (Cu but no Li) already exhibits clear O-O dimer signatures, whereas Li primarily stabilizes the framework and mitigates Jahn-Teller distortions (as briefly suggested in the O K edge discussion)? A more systematic comparison of O K edge and O 1s spectra among NMTO, NMMTO, NCMMTO, and NLCMMTO at matched states of charge would be very helpful to understand the role of each element.

Response: Thanks for your helpful suggestions. We have collected the O K-edge of different samples with each charge of states via X-ray absorption spectroscopy using different hemispherical analyzer (HA) retardation voltages to clarify the origin of the

spectral feature at 533.6 eV (**Figure R8a** and **R8b**). With systematically increasing HA values, the intensity of the peak centered at 533.6 eV exhibited a monotonic enhancement, whereas the lower energy pre-edge features which are associated with TM-O hybridized states showed comparatively weaker dependence on HA. Since increasing HA preferentially enhances the contribution from higher kinetic energy emitted electrons and suppresses background signals, the pronounced HA dependence of the 533.6 eV feature indicates that it originates from the high energy O 2p hole states related to the anion redox activity which belongs to the generation of delocalized state oxygen species. From **Figure R8c** and **R8d**, in PEY mode, the NLCMMTO and NCMMTO with 3.5V charge-state exhibit pronounced feature at ~533.6 eV, indicating the introduction of Cu facilitates the generation of delocalized state oxygen hole species during charging. The formation of such oxygen species hosting delocalized hole states promotes the reconfiguration of electronic density within the lattice, which modulates the electrostatic repulsion between interlayer oxygen atoms and consequently leads to the observed starting of contraction of the interlayer spacing at 3.5V charging state. Such delocalized O 2p states could also act as a "charge buffer" within the TM-O network. This delocalization reduces the localization of electrons on d orbitals of transition metal, thereby alleviating orbital degeneracy and suppressing Jahn-Teller distortions, which could be helpful to stabilize the structural integrity, which is confirmed by the higher capacity retention. When the charging states up to 4.15V, the NLCMMTO still shows a distinct feature at 533.6 eV. In contrast, the peak around 533 eV in NCMMTO disappears and is replaced by a shoulder at ~534.9 eV, which is attributed to localized O-O configurations, suggesting a further oxidation of lattice oxygen. Such behavior is consequence of the introduction of Li, which stabilizes delocalized oxygen hole states by enhancing local electrostatic screening and TM-O hybridization, thereby increasing the energetic barrier for O-O bond formation. As a result, the evolution toward localized O-O σ* configurations at high states of charge are effectively suppressed and enhance reversibility. Notably, a similar evolution of the ~533.6 eV feature is consistently observed in TEY spectra shown in **Figure R8e** and **8f**. The agreement among these detection modes with different probing depths confirms

that the spectral change originates from intrinsic lattice oxygen electronic states rather than surface related side reactions. Thus, Li plays a crucial role in stabilizing delocalized oxygen vacancies and maintaining reversible oxygen redox activity, which contributes to enhanced structural stability upon long-term cycling and facilitates faster ion migration.

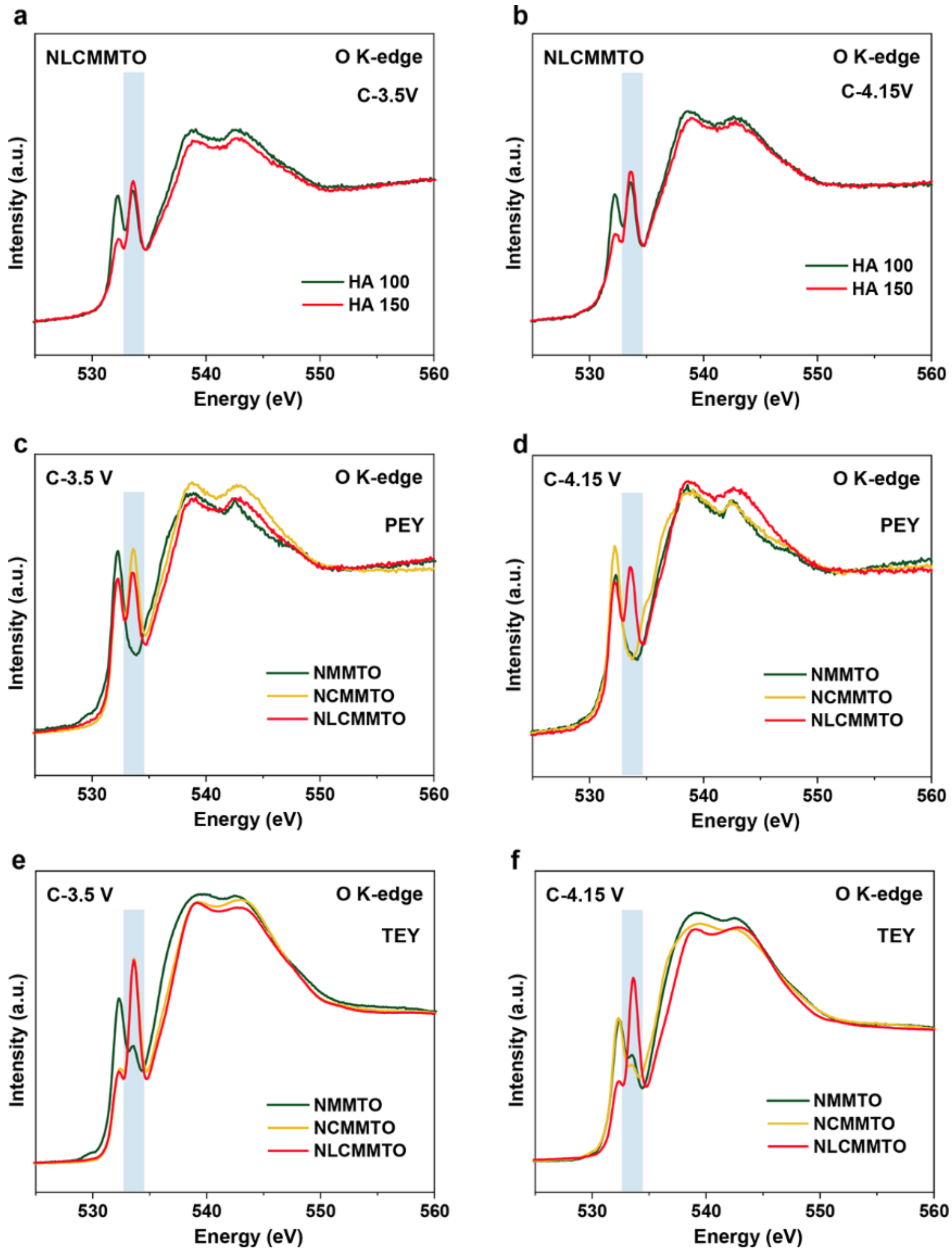

Figure R8 O K-edge XAS spectra of NLCMMTO with different hemispherical analyzer parameters at **a,** 3.5 V; **b,** 4.15V. PEY O K-edge spectra for NMMTO, NCMMTO, and NLCMMTO at **c,** 3.5 V; **d,** 4.15V. TEY O K-edge spectra for NMMTO, NCMMTO, and NLCMMTO at **e,** 3.5 V; **f,** 4.15V.

4. Authors state that the "self adaptive volume" and stress relief mechanism is attributed to Ni-O_2^{n-}-Mn superexchange mediated by Cu induced O dimers. It is a key point of the manuscript, but at current version, it seems to be based mainly on inference rather than direct evidence. Although conducting entirely new experiments may be difficult, the authors should either: 1) provide some experimental analysis that directly supports the observed changes in magnetic interactions upon Cu substitution and charging, or 2) considerably qualify their conclusion and indicate that superexchange remains a possible, but not fully confirmed, mechanism.

Response: Thanks for your helpful suggestions. we intended to use "superexchange" to describe the charge compensation behavior between Ni and Mn observed in the in-situ XAS. However, as pointed out by the reviewer, superexchange has a strict physical definition that requires direct magnetic exchange interactions. Therefore, we have revised this description accordingly.

Specifically, we have replaced the term superexchange with a more general expression, namely a bridged Ni-O-Mn configuration, which better reflects the experimentally accessible structural and electronic features without implying a rigorously established magnetic coupling mechanism. Despite the absence of direct magnetic evidence, the interaction between Ni and Mn is supported by multiple experimental observations. O K-edge spectra shown in **Figure R9a** confirms the involution of oxygen during charging process. Ex-situ (**Figure R9b and R9c**) and in-situ (**Figure R9b-R9j**) XAS spectra of Mn and Ni collected at different states of charging shows the valence changes under equilibrium and dynamic conditions. Both ex-situ and in-situ XAS and SXR measurements consistently reveal a coupled redox evolution of Ni and Mn during charging and discharging process. In particular, the oxidation of Ni is accompanied by a relative reduction tendency of Mn, indicating the presence of internal charge redistribution within the transition metal sublattice. Combined with emergence of the feature at 533.6 eV, this indicates enhanced TM-O hybridization during charging, accompanied by increased oxygen activity, suggesting an electronically interconnected TM-O framework. Furthermore, the spectroscopic features, together with the electrochemical response, point to a delocalized charge

compensation mechanism, likely involving hybridized TM-O states. Oxygen acts as an electronic mediator linking adjacent transition metal centers, enabling charge redistribution across the Ni-O-Mn network.

Accordingly, we have revised the manuscript to clearly state that the proposed mechanism should be regarded as a reasonable explanation based on experimental evidence and literature precedent, rather than a conclusively established interaction. We believe that this more cautious formulation better reflects the scope of the present work while preserving the underlying physical picture of a bridged Ni-O-Mn configuration with delocalized O hole states.

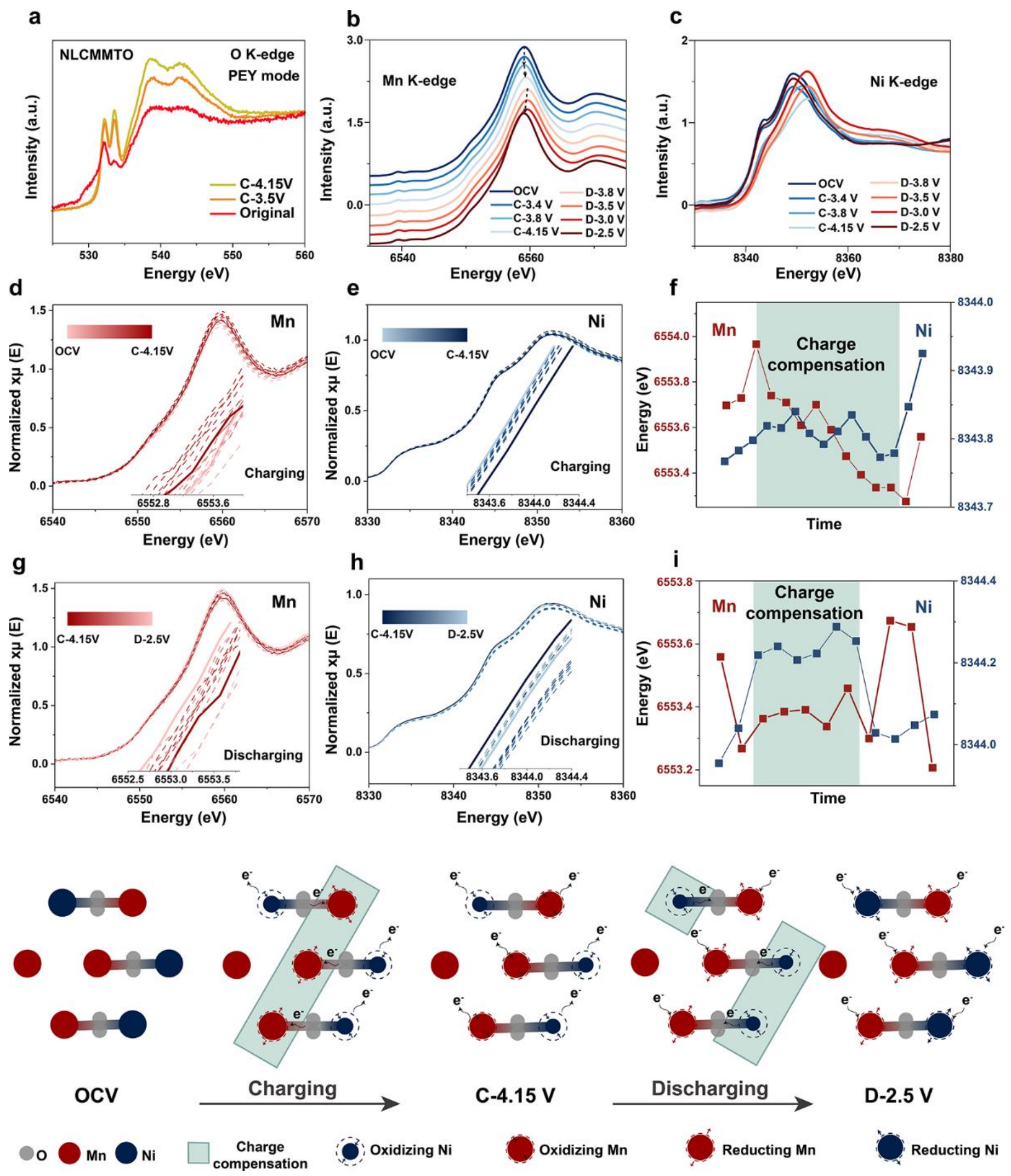

Figure R9 (Figure 5c, 5d, 6b-6h) a, PEY O K-edge XAS spectra of NLCMMTO with different states of charging. **b,** Ex-situ Mn XAS spectra collected at different states of charging. **c,** Ex-situ Ni XAS spectra collected at different states of charging. In-situ

XAS spectra of **d,** Mn K-edge; **e,** Ni K-edge of NLCMMTO during charging process. **f,** The variation of energy for NLCMMTO during charging process. In-situ XAS spectra of **g,** Mn K-edge; **h,** Ni K-edge of NLCMMTO during discharging process. **i,** The variation of energy for NLCMMTO during discharging process. **j,** Charge compensation between the TM ions diagram.

5. The Tresca (spelled "Treca" in the manuscript) stress calculations show that NLCMMTO has lower and more uniform stress than NMMTO at 4.15 V. Please clarify how the calculated structures used for stress analysis were chosen (with or without O-O dimers, with which Na content, etc.), and how the stress distribution correlates with the observed lattice parameter evolution and with the measured electrochemical cycling stability. If possible, show a direct correlation between calculated stress and experimental Δc/c or ΔV/V for the different compositions.

Response: Thanks for your comment regarding the Tresca stress analysis and its correlation with structural evolution and electrochemical stability.

Regarding the selection of calculated structures, the stress analysis was performed using a continuum scale chemo-mechanical finite element model. In this framework, the electrode is treated as a deformable solid in which Na transport is described by Fickian diffusion, and the local stress is calculated from the coupling between Na concentration and volumetric strain through the constitutive relations of the material. Therefore, the calculated structures correspond to representative Na contents at the charged state (4.15 V), rather than automictically reconstructed configurations. The oxygen cluster formation was not explicitly considered, as such processes are highly confined and stochastic at the atomic scale and cannot be straightforwardly incorporated into the governing equations of a continuum FEM model. Moreover, their contribution to the overall mechanical stress is expected to be minor compared to the dominant stress arising from concentration-induced volumetric deformation. Thus, neglecting local O-O bonding effects is a reasonable approximation for capturing the primary stress evolution at the electrode scale.

The Tresca stress distribution reflects the non-uniform deformation induced by Na extraction under mechanical constraints. At 4.15 V, NLCMMTO exhibits a lower and

more homogeneous stress distribution than NMMTO, indicating a more uniform accommodation of concentration induced strain. This behavior is consistent with the experimentally observed lattice parameter evolution, where NLCMMTO shows a smoother and less anisotropic change in lattice spacing compared with NMMTO. In particular, the reduced in-plane contraction and moderated c-axis evolution imply smaller strain gradients, which directly translate into lower equivalent shear (Tresca) stress. The lower and more uniform stress state of NLCMMTO provides a mechanistic explanation for its superior cycling stability. Reduced stress concentration mitigates crack initiation, intergranular decohesion, and irreversible structural degradation during repeated cycling, thereby contributing to the improved capacity retention observed experimentally.

$$\sigma_{Tresca} \approx |\varepsilon_c - \varepsilon_a|$$

$$\varepsilon_c = \frac{\Delta c}{c} \quad \varepsilon_a = \frac{\Delta a}{a}$$

The lattice strain anisotropy $|\varepsilon_c - \varepsilon_a|$ of NMMTO and NLCMMTO evaluated based on in-situ synchrotron XRD are 3.7% and 0.4%, respectively. The Tresca stress is found to scale with this anisotropic strain. A larger anisotropic strain induces higher shear stress concentration, whereas isotropic lattice evolution leads to a more homogeneous stress distribution.

6. The manuscript briefly states that the O3 phase provides mechanical support, but this is not substantiated. Please either: 1) Provide some evidence that varying O3 content (e.g., via different synthesis conditions) changes cycling stability and/or lattice evolution, or 2) Reframe the statement more cautiously, acknowledging that the precise role of the O3 fraction is not fully understood. Also, if the interfaces between P2 and O3 domains are believed to contribute to stress accommodation or Na ion transport (as often suggested in P2/O3 intergrowth systems), this should be discussed explicitly, possibly with schematic or DFT insights.

Response: Thanks for your comment. Shown as **Figure R10a**, 4D-STEM results show that the region with smaller interlayer spacing (~0.56 nm), assigned to the O3 phase, exhibits relatively uniform phase contrast and weaker charge modulation (**Figure**

R10b). This observation suggests a more homogeneous local electrostatic environment in O3 domains. Notably, the transition between P2 and O3 domains occurs over a finite region rather than an abrupt boundary. Within this intergrowth region, both phase and charge maps exhibit clear perturbations, including lattice distortion and enhanced, spatially heterogeneous charge distribution, indicating local structural reconstruction and charge redistribution at the interface. The interfacial region may accommodate local lattice mismatch and introduce variations in the electrostatic landscape that could enhance Na^+ transport.[8] By introducing Li, a higher fraction of the O3 phase can be induced. Rietveld-refined XRD results show that NLCMMTO contains more O3 phase than NCMMTO. As a result, the NLCMMTO exhibits highly stable long-cycling performance.

Related description has been added in the 4D-STEM section of revised manuscript: These observations suggest that the intergrowth interface acts as a structurally adaptive and electrostatically active region, which may accommodate lattice mismatch and facilitate Na^+ transport through locally modified potential landscapes.

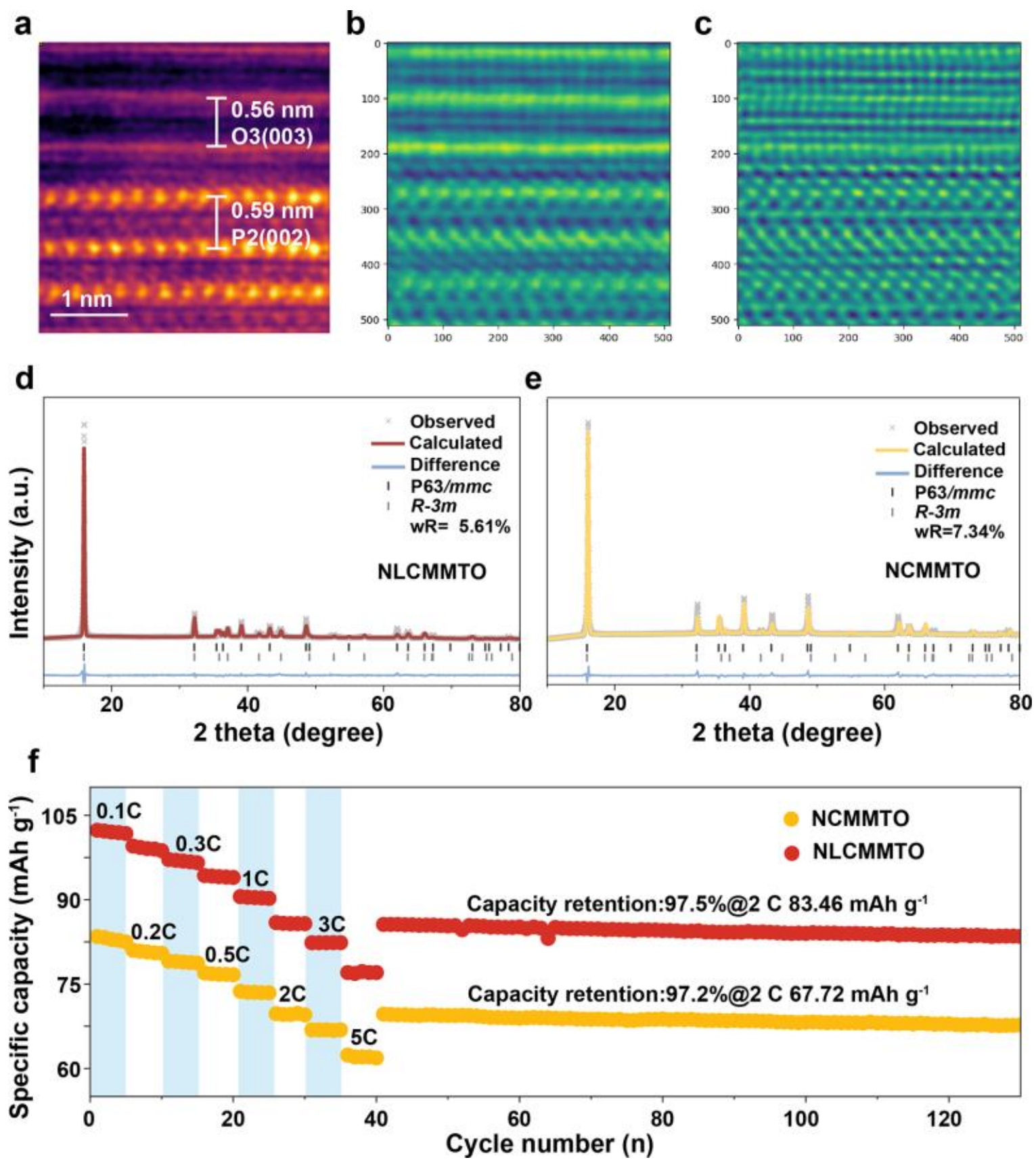

Figure R10 (Figure S9, 1a) a, BF-contrast inversion, **b,** phase-contrast image

(projected electrostatic potential) and **c,** calculated charge density of P2 and O3 intergrowth area in NLCMMTO. XRD patterns of **d,** NCMMTO and **e,** NLCMMTO. **f,** Rate and cycling performance of NCMMTO and NLCMMTO.

7. Ti constitutes a large fraction of the transition metal sublattice ($Ti_{1/3}$), yet its functional role is only briefly mentioned. Please clarify whether Ti is entirely electrochemically inactive in the investigated voltage range (2.5-4.15 V) and provide evidence (e.g., Ti K edge XAS or indirect arguments) if available.

Response: Thanks for your comment. In the investigated voltage window of 2.5-4.15 V, Ti is electrochemically inactive. This conclusion is directly supported by Ti K-edge XAS measurements collected at different states of charge. As shown in **Figure R11**, the Ti K-edge position and spectral features remain unchanged during charging and discharging, indicating that the oxidation state of Ti remains stable Ti^{4+} throughout the electrochemical process.

The absence of any detectable edge shift or pre-edge evolution confirms that Ti does not participate in charge compensation within this voltage range. Instead, Ti plays a structural stabilization role by remaining redox inactive, which helps suppress excessive lattice distortion and mitigates irreversible structural degradation during cycling. This stabilizing effect is consistent with the improved cycling stability and reduced mechanical stress observed for NLCMMTO.

Additionally, there are numerous studies on Ti-doped layered oxide cathodes for sodium ion batteries that confirm that Ti^{4+} acts as an inactive electrochemically inert stabilizer. For example, Liang et al. reported that Mg and Ti dopants in bulk layered cathode materials are electrochemically inactive and even reduce the achievable capacity by partially substituting electrochemically active Ni (Nat Commun. 2025, 16, 3505). Jia et al. also mentioned that Ti remains electrochemically inactive in the cathodes by DFT calculation that 3 d states were concentrated above the Fermi energy level (~2.0 eV) (Nat. Commun. 2025, 16, 10477). Meanwhile, the $Na_{2/3}[Ni_{1/4}Mn_{1/2}Ti_{1/6}Zn_{1/12}]O_2$ cathodes reported by Komaba et al. were found to 568 have no significant energy shift at the K-edge of Ti by in situ XANES spectroscopic

tests, suggesting that Ti is essentially uninvolved in redox reactions (Adv. Mater. 2023, 54, 202300714).

We have revised the manuscript to explicitly clarify the electrochemically inactive nature of Ti and to include the corresponding Ti K-edge XAS evidence (lines:329-332): Since Cu and Ti are electrochemically inactive at 2.5-4.15 V operating window evidenced by XAS showing no detectable energy shift across different states of charge (Figure S45), Ni and Mn redox couples have been the focus of investigation.

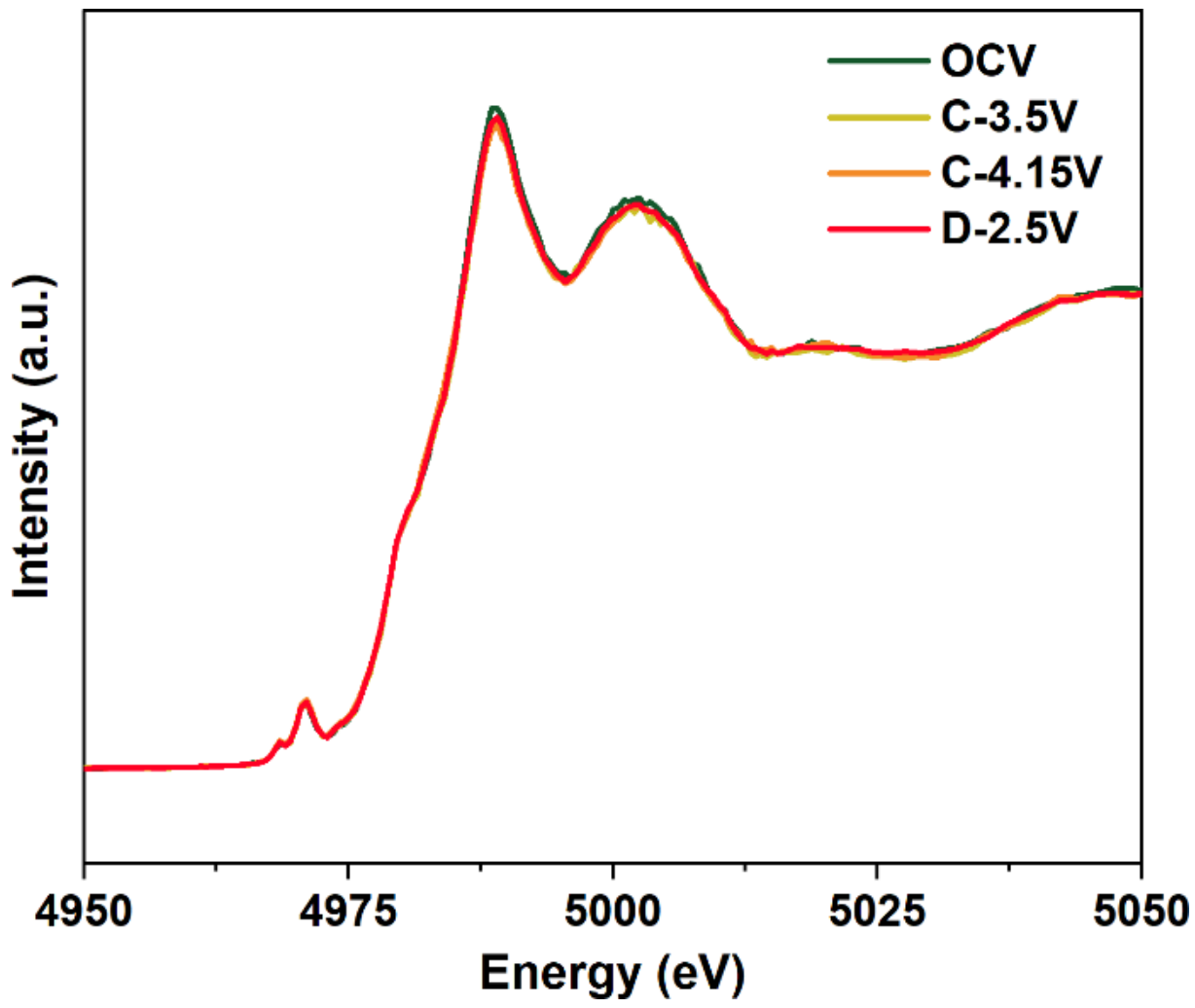

Figure R11 Ti K-edge XAS spectra of NLCMMTO at different states of charging.

8. The configurational entropy argument is used to rationalize improved stability, but the manuscript also emphasizes very specific roles of individual elements (Cu → O dimers, Li → relieve interlayer deformation, Ti → framework strengthening, etc.). It would be useful to distinguish between the general stabilizing effect from high configurational entropy and the specific electronic or structural influences of individual elements. For instance, could the authors provide configurational entropy values (S_conf) for NMTO, NMMTO, NCMMTO, and NLCMMTO, and analyze whether performance aligns with these entropic values or if the contributions from Cu, Li, or Ti are more significant than the entropy itself?

Response: Thanks for your comment. According to the suggestion of reviewer, we calculated the configurational entropy (S_conf) of the transition metal layers based on equal x.

$$\Delta S_{config}^{TM} = -R \sum x_i \, ln x_i$$

The S_conf of NMMTO is 9.13 J mol^{-1} K^{-1} (1.1R, low entropy); NMMTO is 11.06 J mol^{-1} K^{-1} (1.33R, middle entropy); NCMMTO is 11.94 J mol^{-1} K^{-1} (1.44R, middle entropy); NLCMMTO is 12.60 J mol^{-1} K^{-1} (1.52R, high entropy). This trend confirms a gradual increase in entropy with increasing compositional complexity shown in **Figure R12a**.

However, from **Figure R12b-R12d**, the electrochemical performance does not scale proportionally with S_conf. While the increase in entropy correlates with improved structural stability to some extent, the magnitude of performance enhancement cannot be explained by entropy alone. NMTO with the lowest entropy value achieves higher discharging capacity than NCMMTO (middle entropy value). We therefore propose that configurational entropy plays a secondary, background role, primarily contributing to global thermodynamic stabilization by suppressing phase separation and reducing the driving force for structural transformation[9].

In contrast, the dominant contribution arises from the synergistic effects of element specific functionalities enabled by rational compositional design. In specific, Cu regulates charge configuration in TM-O framework by enhancing oxygen activity. Li stabilizes the reversible oxygen redox enhancing the structural integrity.

Importantly, these effects are not independent but cooperative, leading to a cumulative gain that exceeds the contribution expected from entropy increase alone. Therefore, while configurational entropy contributes to stabilizing the overall structure, the enhanced electrochemical performance in this system is primarily governed by elemental synergy arising from deliberate substitution design, rather than by the monotonic increase of S_conf.

We have revised the manuscript (lines 208-211): This structural stability is attributed to the synergistic effects of element specific functionalities enabled by rational compositional design together with high-entropy effect.

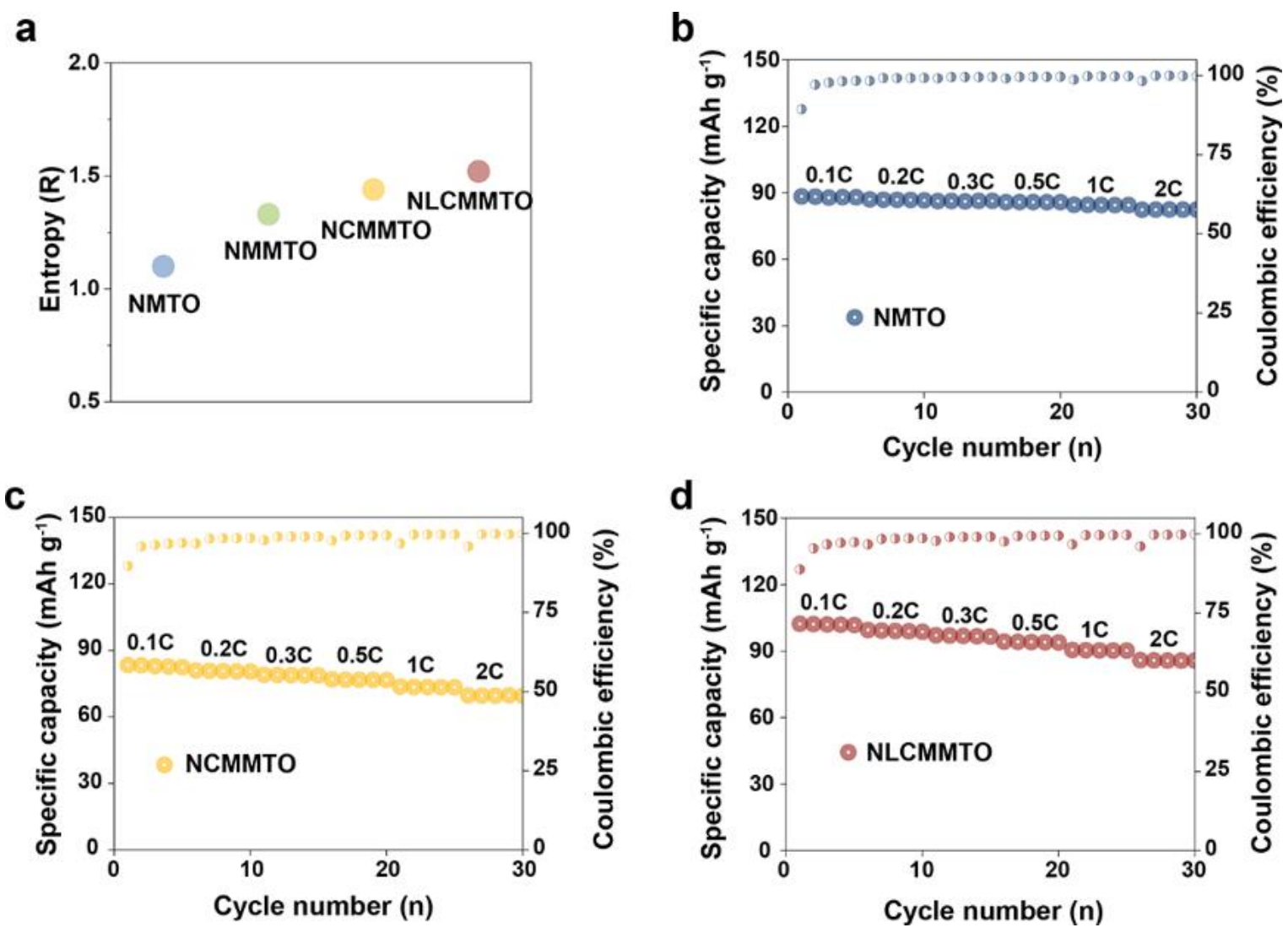

Figure R12 a, The configurational entropy value of NMTO, NMMTO, NCMMTO and NLCMMTO. Rate performance of **b,** NMTO, **c,** NCMMTO, and **d,** NLCMMTO.

9. Treca stress" should presumably be "Tresca stress." Please correct and briefly define the chosen stress criterion the first time it appears. The phrase 'self-adaptive volume behavior' is important to the manuscript but is somewhat vague. Providing a definition in the Introduction would make its meaning more precise.

Response: Thank you for your careful reading and constructive suggestions.

The term "Treca stress" has been corrected to "Tresca stress" throughout the manuscript. In addition, a brief definition of the Tresca yield criterion has been provided at its first occurrence. We agree that the phrase "self-adaptive volume behavior" was previously ambiguous. It has now been clearly defined in the Introduction as an adjustable structural evolution during Na extraction, where the interlayer spacing first expands due to O-O repulsion and then contracts at high voltage due to oxygen electronic structure rearrangement. This adaptive behavior helps mitigate stress accumulation and improve structural stability. These revisions improve the clarity and precision of the manuscript lines 79-81: Surprisingly, such NLCMMTO exhibits self-adaptive structural behavior, which could effectively accommodate volume fluctuations that interlayer spacing first expands due to O-O repulsion and then contracts at high voltage due to charge reconfiguration in TM-O framework.

10. In several main figures (e.g., in situ synchrotron XRD maps and in situ XAS), the scale bars, color bars, and labels are relatively small and difficult to read. And please carefully proofread the manuscript for minor grammatical errors and typos (e.g., "distorti00on", "Treca", "planer CuO4", etc.).

Response: Thanks for the helpful suggestion. The scale bars, color bars, and labels in the relevant main figures (including the in-situ synchrotron XRD maps and in-situ XAS figures) have been enlarged to improve readability. The revised figures are presented below.

In addition, we have carefully proofread the manuscript and corrected minor grammatical errors and typographical mistakes throughout the text. For example, "distorti00on", "Treca", and "planer CuO_4" have been corrected to "distortion", "Tresca", and "planar CuO_4", respectively.

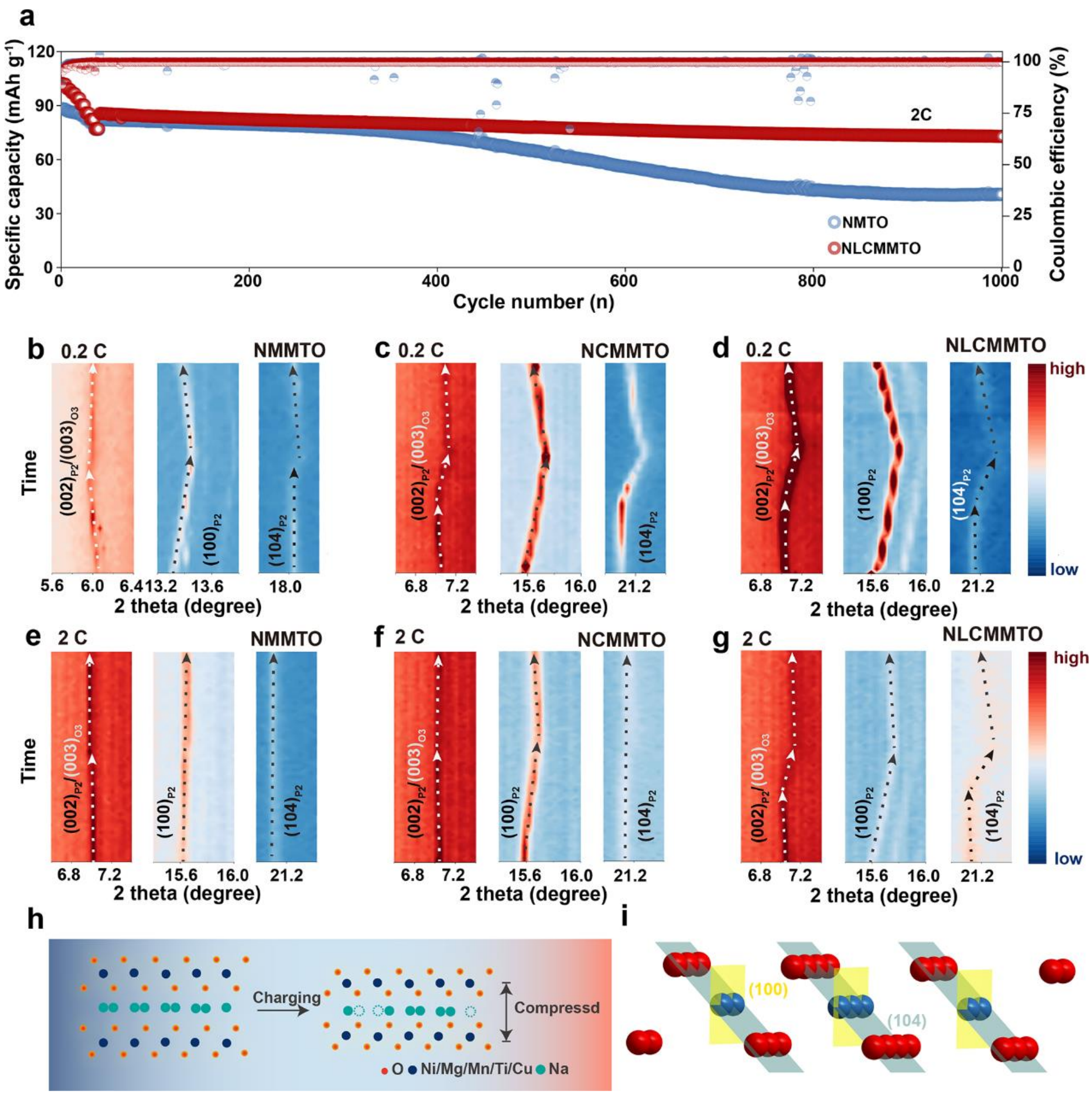

Figure R13 (Figure 3) a, electrochemical performance of NLCMMTO and NMTO **b-d,** selected in-situ synchrotron based XRD pattern collected during the first

charge/discharge in the voltage range of 2-4.15 V at 0.2C of NMMTO, NCMMTO, and NLCMMTO. **e-g,** selected in-situ synchrotron-based XRD pattern collected during the first charge/discharge in the voltage range of 2-4.15 V at 2C of NMMTO, NCMMTO, and NLCMMTO. **h,** Schematic picture of self-adaptive volume behavior in NLCMMTO. **i,** (100) and (104) crystal plane diagram.

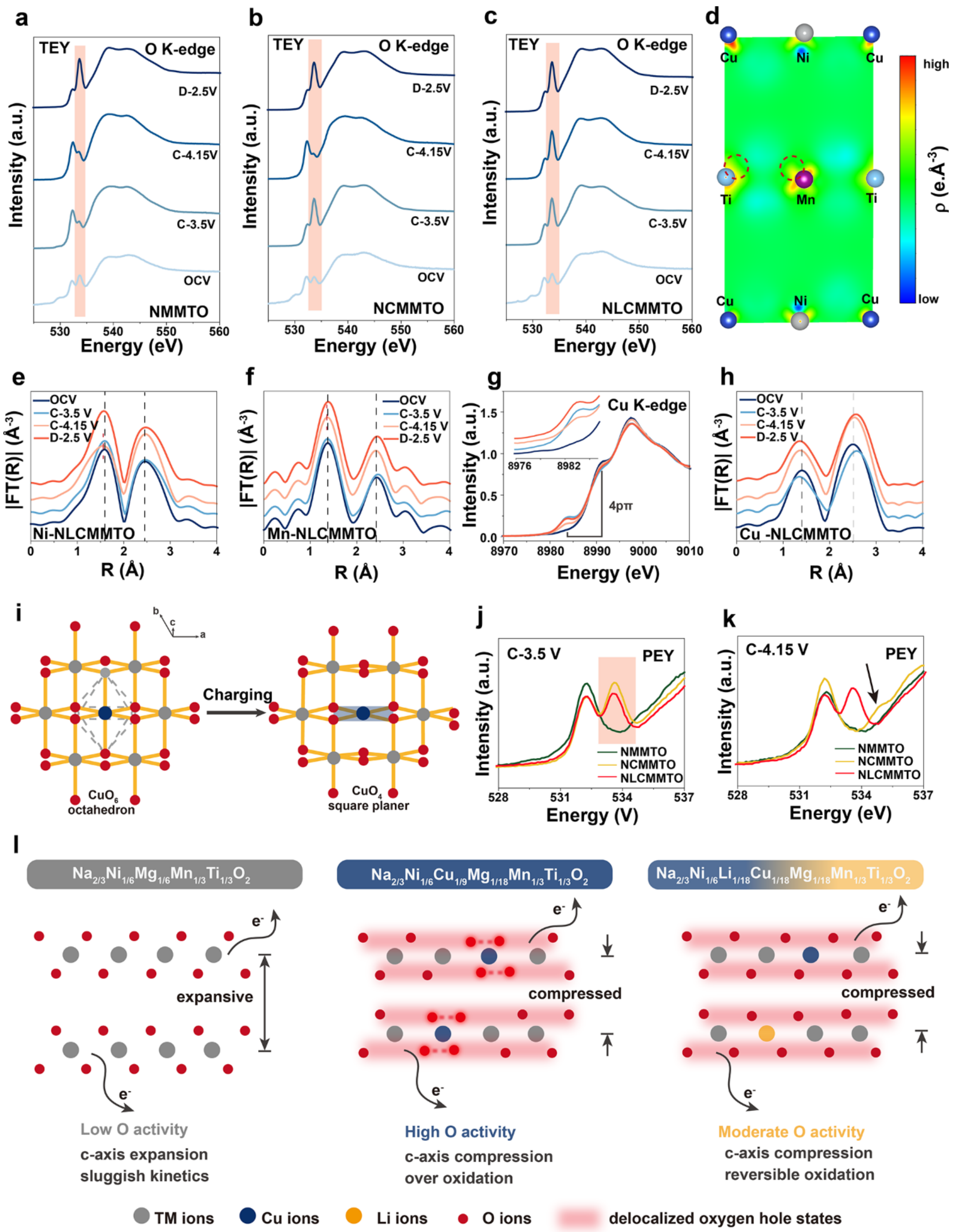

Figure R14 (Figure 4) TEY O K-edge XAS of **a,** NMMTO, **b,** NCMMTO, **c,** NLCMMTO electrodes from different charge states. **d,** Calculation of the charge density distribution of NCMMTO. **e,** Ni, **f,** Mn EXAFS in NLCMMTO at different charge state. **g,** Cu K-edge XANES in NLCMMTO at different charge state. **h,** Cu EXAFS in NLCMMTO at different charge state. **i,** Schematic image of structure

evolution of Cu. PEY O K-edge XAS of NMMTO, NCMMTO, and NLCMMTO **j,** at 3.5V; **k,** at 4.15V. **l,** Schematic image of oxygen involved lattice evolution in layered oxides at highest voltage (4.15V).

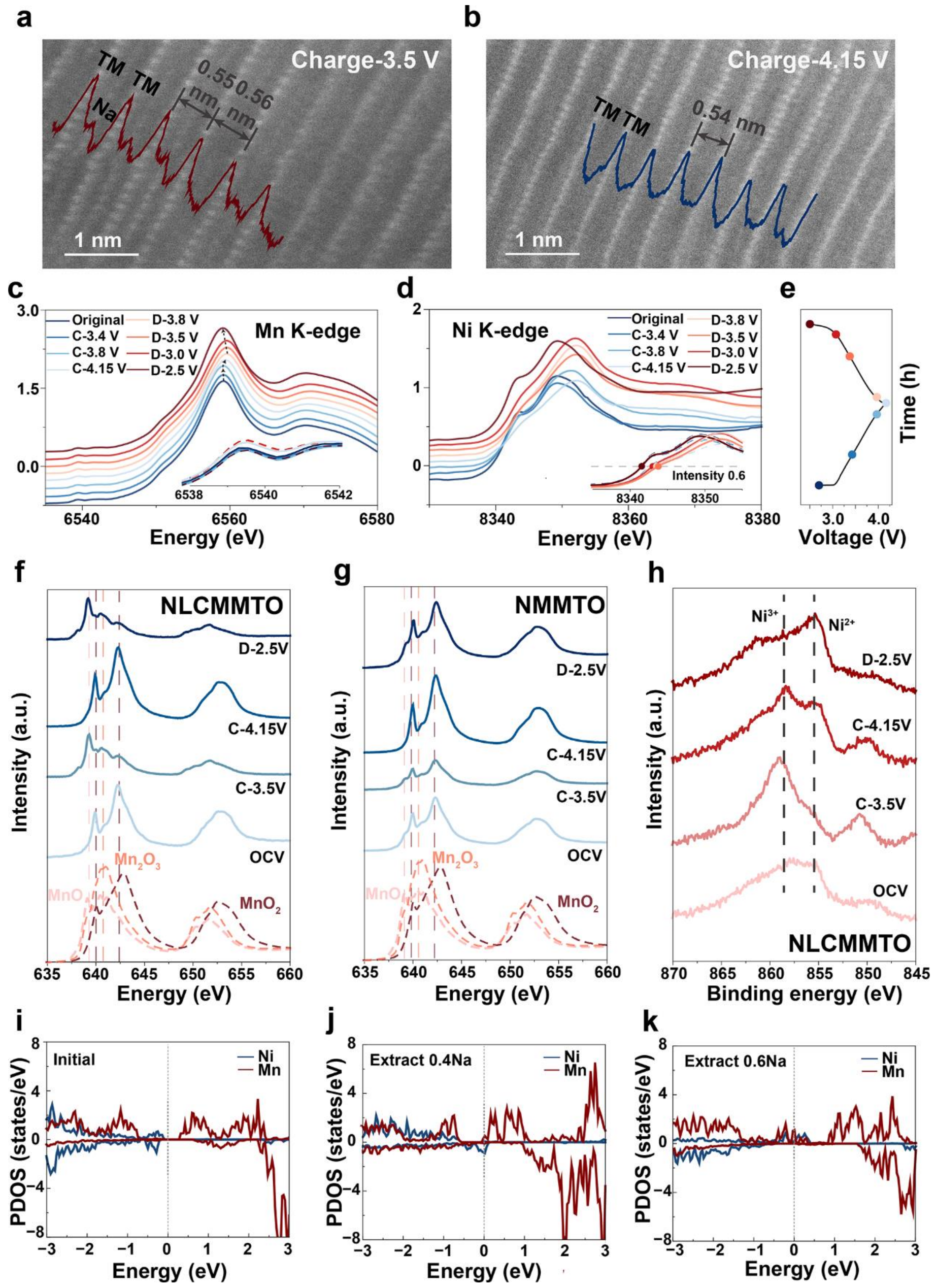

Figure R15 (Figure 5) HAADF-STEM pattern of NLCMMTO at charging **a,** 3.5V, **b,** 4.15V. **c,** Mn K-edge, **d,** Ni K-edge of NLCMMTO at different charge states. **e,** the profile of charge/discharge at 0.1 C. Mn L-edge of **f,** NLCMMTO and **g,** NMMTO at different states. **h,** Ni 2p XPS spectrum NLCMMTO. PDOS calculation of **i** initial state, **j,** extract 0.4 Na and **k,** extract 0.6 Na of P2 phase in NLCMMTO.

Reviewer #3 (Remarks to the Author):

The authors report the synthesis and advanced characterization of a layered oxide cathode material, $Na_{2/3}Ni_{1/6}Li_{1/18}Cu_{1/18}Mg_{1/18}Mn_{1/3}Ti_{1/3}O_2$ (NLCMMTO). They highlight NLCMMTO as a next-generation compound of interest for sustainable energy infrastructures, due to its electrochemical stability, resource abundance, and scalability. This material exhibits a self-adaptive volume regulation, with more attractive properties than the parent compound NMMTO, and NCMMTO. Several experimental techniques, some of which are supported by theoretical calculations, are used to provide a broad understanding of the structural and electronic properties of these compounds during charging and discharging, in line with their electrochemical properties. High quality X-ray absorption spectroscopy (ex situ and in situ), XPS, 4D-STEM, as well as high-resolution XRD provide a significant amount of information.

This is a thorough report, impressive by the large quantity of data reported, and the clear objective to systematically evaluate the evolution of the electrochemical properties through the structural, chemical , and electronic properties. Nevertheless, although the manuscript contains a very descriptive presentation of the results, the presentation and interpretation of some results could be clarified, and the discussion of the results in a broader context and their impact in the field is missing. One of the major issues in the manuscript is the lack of explanation for the role played by Li on the properties of NLCMMTO. Therefore, it is my understanding that the current version of the manuscript lacks the depth and impact expected to be considered for publication. I provide additional comments below.

Response: We sincerely thank the reviewer for the careful reading of our manuscript and for the constructive and insightful comments. Based on reviewer's suggestion, we further clarify the underlying mechanisms, and a stronger discussion of the broader impact are necessary, particularly regarding the role of Li in NLCMMTO. Combined with the results of SXR, the function of Li is studied systemically, which stabilizes delocalized O 2p states within the TM-O network, alters the charge compensation mechanism as the charge buffer between Ni and Mn, boosting electron and ion

movement during electrochemical cycling. Li plays a critical role in controlling the reversibility of oxygen redox, structural stability, and improving electrochemical performance. This insight provides a general design principle for advanced layered oxide cathodes that manipulate interlayer distance by building charge reconfiguration TM-O framework to achieve stable long term cycling performance. We believe these revisions significantly improve the depth, clarity, and impact of the manuscript, and we hope that the revised version meets the expectations for publication.

1 The manuscript would benefit from professional editing to correct English grammar, missing words and inconsistencies in the construction of sentences throughout the manuscript, including captions of figures in the main text and SI. Some examples:

- "However, in NLCMMTO that adding Li, the peak at 533 eV is slightly weaker demonstrating relieves the interlayer deformation stress in the TM layers."
- "The crystalline fact..."
- "… shell layer, the closely atomic…"
- "… all reflection peaks are kept combination with…"
- "… distorti00on…"
- "To gain the more direct the oxygen evolution behavior, the …"
- "Samples have also collected…"
- "A shape peak…"
- "was calculation in…"
- "planer CuO4…"
- "the function of Cu substitution"
- "…could partly present valence of Mn distinctly. From Figure 5c, the shape of Mn pre-edge shows broad and weak which represents that…"
- "desodium"

etc…

Response: We sincerely thank the reviewer for pointing out the issues related to English grammar and sentence construction. We fully agree that the language of the manuscript required improvement. In revised manuscript, including the main text, figure captions,

and Supporting Information, has been carefully revised to correct grammatical errors, missing words, and inconsistencies in sentence structure. In addition, the manuscript has been professionally edited by a native English speaker to ensure clarity, accuracy, and readability.

The specific examples provided by the reviewer have all been corrected in the revised version. These revisions have significantly improved the overall quality and readability of the manuscript. For example:

- "However, in NLCMMTO that adding Li, the peak at 533 eV is slightly weaker demonstrating relieves the interlayer deformation stress in the TM layers." change to "In contrast, Li incorporation in NLCMMTO slightly suppresses this peak, suggesting alleviated interlayer deformation stress in the TM layers." (lines 142-144)

- "The crystalline fact..." change to "crystalline plane" (line 123)

- "… shell layer, the closely atomic…" change to "…shell layer, respectively. Similar spectral features and close atomic distances…" (lines 129-133)

- "… all reflection peaks are kept combination with…" change to "All diffraction peaks are largely retained with slight shifts compared to the pristine state, indicating negligible structural changes during cycling." (lines 230-231)

- "To gain the more direct the oxygen evolution behavior, the …" change to "To obtain direct insight into the oxygen evolution behavior…" (line 269)

- "was calculation in…" change to "was calculated…" (line 283)

- "… distorti00on…" change to "distortion"; "planer CuO4…" change to "planar CuO_4"; "desodium" change to "desodiation"

2 The manuscript contains a lot of experimental data from multiple techniques covering various lengthscales, sometimes combined with theoretical calculations. The data provide evidence of different behaviours of NMMTO, NCMMTO, and NLCMMTO. This is factually underlined by the author at the end of the introduction (line 81-82): "The introduction of Li plays an important role in strengthening the structural framework and preserving mechanical integrity". The reported difference of electrochemical behaviour, and subsequent structural and chemical properties between NLCMMTO and NCMMTO is puzzling. Nevertheless, it is rather disappointing that

the authors do not provide an explanation for the effect induced by Li incorporation in the structure on the material properties. This is a major weakness of the manuscript.

Response: Thanks for your valuable suggestions. Electrochemical performance most directly reflects the function of Li. It can be seen from **Figure R16a** that NLCMMTO exhibits higher specific capacity and better stability. To better understand the role of Li, SXR was employed to the electrodes of NCMMTO and NLCMMTO at different states of charge. As shown in Figure R3b, NCMMTO and NLCMMTO exhibit a characteristic feature at ~533.6 eV when charged to 3.5 V, which is attributed to delocalized O 2p states associated with enhanced TM-O hybridization,[1] likely arising from the formation of Ni-O-Mn framework with delocalized O hole states, in which electron holes are distributed over the TM-O network rather than localized on specific sites.[2] Upon further charging, NLCMMTO retains this feature, indicating that the electron holes remain delocalized within the lattice. In contrast, the corresponding peak in NCMMTO gradually disappears and evolves into a higher energy shoulder at ~534.9 eV in **Figure R16c**, suggesting further oxidation of lattice oxygen accompanied by localized oxygen interactions which might be harmful to the structure integrity and Na^+ transfer kinetics due to the irreversibility. Such distinct behavior highlights the critical role of Li incorporation.

Specifically, such delocalized O 2p states could act as a “charge buffer” within the TM-O network. This delocalization reduces the localization of electrons on transition metal (Ni Mn) d orbitals, thereby alleviating orbital degeneracy and suppressing Jahn-Teller distortions, which could be helpful to stabilize the structural integrity, which is confirmed by the higher capacity retention.

Additionally, the more symmetric TM-O environment caused by reduction of Jahn-Teller distortions, increasing the energetic barrier for O-O bond formation that can hinder Na^+ diffusion by inducing local structural distortion, blocking diffusion pathways, and increasing migration barriers. Consequently, the evolution toward localized O-O σ* configurations at high states of charge is suppressed, leading to improved reversibility and kinetics of the oxygen participation process. Thus, the GITT tests were employed to measure the kinetics of two samples shown in **Figure R16d** and

R16e. It can be observed that at the highest voltage of 4.15V, the NLCMMTO exhibits higher Na^+ diffusion coefficient showing its superior ionic conductivity and kinetic properties. The result is consistent with the SXR spectra.

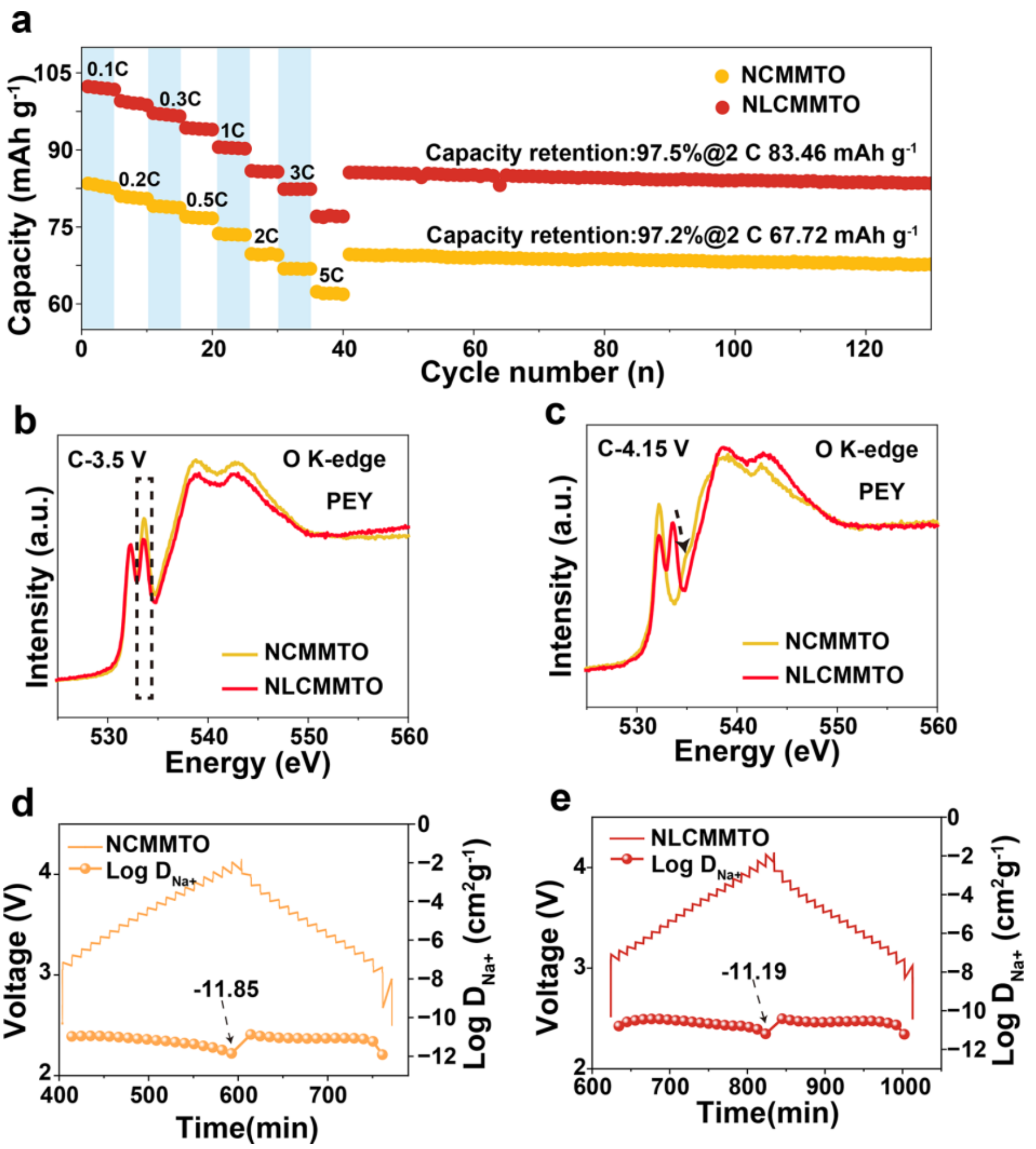

Figure R16(Figure 4j, 4k, S21) a, electrochemical performance of NCMMTO and NLCMMTO**. b,c** O K-edge XAS of NCMMTO and NLCMMTO electrodes charging at 3.5V and 4.15V**. d,e** GITT results of NCMMTO and NLCMMTO**.**

3 The Mn-K edge of NMMTO in Fig. 1c is different compared to NLCMMTO and NCMMTO. The peak at ~6562 eV is much less intense, so is the peak at ~6575 eV, and the ratio of the two peaks seems to differ from the other compounds, contrary to what is mentioned in the text (line 102-104). This is in apparent contradiction with the Mn-L23 edge shown in Fig. 1n. Please comment.

Response: Thanks for your careful examination of Mn K-edge XAS spectra and the detailed observation of difference in the intensity of the peaks at ~6562 eV and ~6575 eV. As noted by the reviewer, slight variations in the intensity can be observed. However, we would like to clarify that both peaks predominantly arise from 1s→4p dipole allowed transitions. The intensity is governed by the unoccupied Mn 4p density of states and highly sensitive to the local coordination geometry, Mn-O hybridization, and multiple scattering effects. In this case, enhanced intensity of the peaks at ~6562 eV and ~6575 eV originates from shortened Mn-O bond lengths, which strengthen Mn-O orbital hybridization and increase the dipole transition probability in NLCMMTO and NCMMTO shown in **Figure R17a**, rather than from changes in Mn oxidation state.

In addition, there is no obvious shift in the pre-edge position and change in intensity of pre-edge (**Figure R17b**). The similar SXR spectra of Mn L-edge also demonstrates that they have same oxidation state with similar valence. Therefore, the variation in the ~6562 eV and ~6575 eV peak intensity is more reasonably attributed to subtle structural and hybridization effects rather than to changes in Mn valence. To avoid possible misunderstanding, we have revised the corresponding description in the revised manuscript as "the nearly identical spectra present that there is no obvious valence change by element substitution in three samples".(lines 108-109)

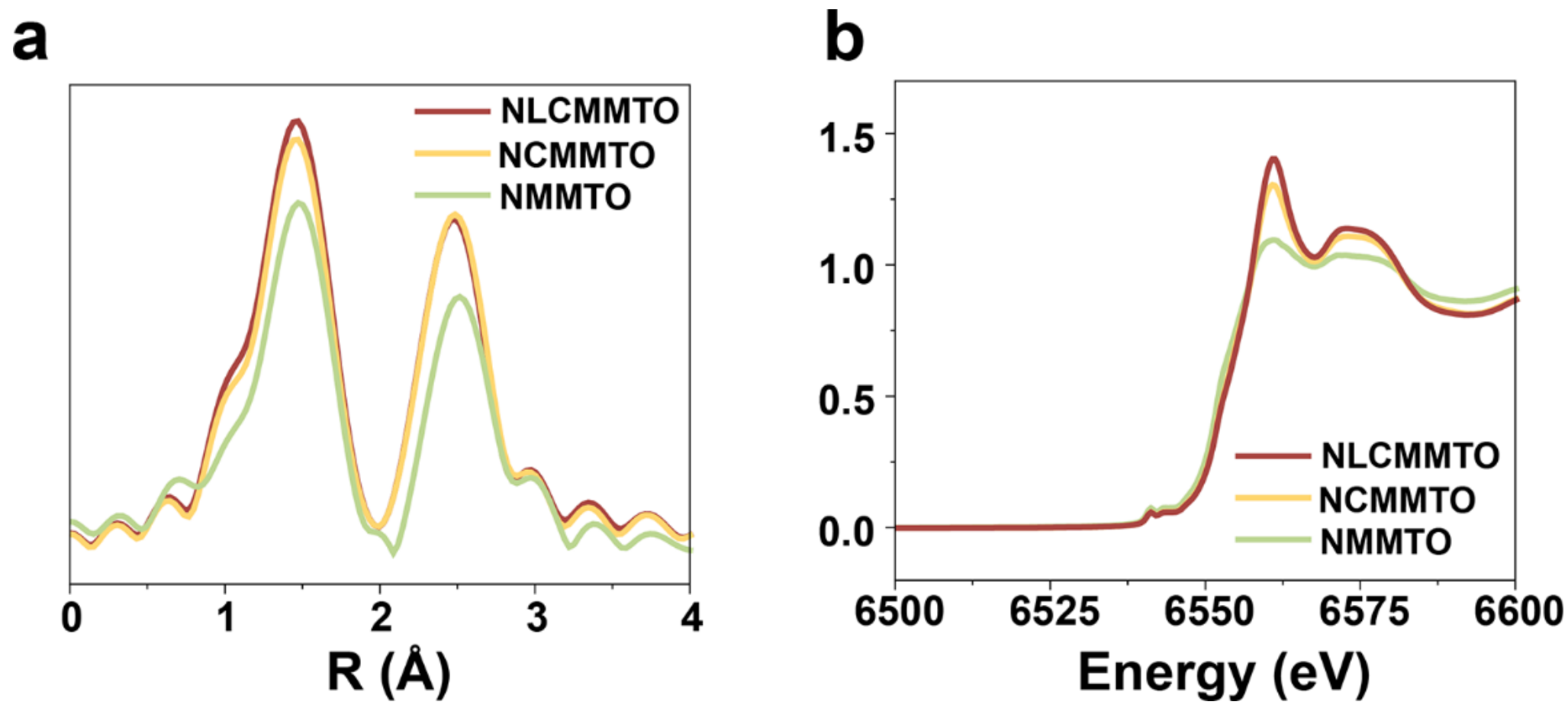

Figure R17 (Figure 1c) a, XAS refinement of Mn for NMMTO, NCMMTO and NLCMMTO. **b,** XAS Mn K-edge of NMMTO, NCMMTO and NLCMMTO**.**

4 The 4D stem data of the P2 and O3 structures leads to electric field and charge density distributions that are visually different. It is surprising that the authors do not point this

out. Specifically, the values of the charge density are much higher in O3 than P2, such difference seems unrealistic.

Furthermore, the authors claim (line 177-180) “The results indicate that, regardless of whether it is the P2 or O3 phase, there is no significant variation in the electric field intensity within the oxygen atom layers. This suggests that the Coulombic interactions between the oxide layers are relatively uniform.” It would be useful to overlay the atomic structure on the maps in Fig. 2f-m. The causal link between the two statements is also not obvious and should be explained further.

Response: Thanks for your comment. We acknowledge that the absolute values of the projected charge density in the P2 phase (**Figure R18a**) appear significantly smaller than those in the O3 phase (**Figure R18b**). This discrepancy is primarily due to variations in sample thickness between the two phases. In 4D-STEM Center-of-Mass (CoM) imaging, the signal scales with the projected potential integrated along the atomic columns; thus, a thicker area (O3 phase in this case) naturally yields higher integrated intensity values. We additionally supplemented the electron energy loss spectroscopy (EELS) spectra of these two phases to determine their thickness differences (**Figure R18c** and **R18d**).

However, our core finding relies on the in-plane spatial uniformity rather than the absolute magnitude. To support this, we have normalized the color scales in the revised Figure 2i and 2m according to their respective sample thicknesses. This removes the thickness artifact and allows for a valid visual comparison of the relative charge distribution. We apologize that the logical connection between “in-plane variation” and “inter-layer Coulombic interactions” was not explicitly detailed. Structural degradation in layered oxides is often initiated by local distortions or gliding of the transition metal oxide (TMO_2) slabs. In systems with charge disproportionation, localized charge variations on oxygen atoms create “hotspots” of uneven electrostatic repulsion between layers. These local fluctuations act as nucleation sites for layer gliding and structural collapse. Our 4D-STEM results reveal a highly uniform charge distribution within the oxygen layers (negligible variance). This implies that the interlayer electrostatic potential landscape is globally smooth and homogeneous. The absence of local charge

localization prevents the formation of stress concentration points that would otherwise trigger phase transitions, thereby enhancing the structural stability of the material during Na^+ extraction/insertion. Crucially, our normalized charge density maps (revised Figure 2i and 2m) reveal a remarkable uniformity in charge distribution across the oxygen sub-lattice for both phases. This lack of localized charge accumulation suggests that the electrostatic potential landscape experienced by the adjacent layers is globally homogeneous. In layered oxides, local variations in electrostatic repulsion often act as nucleation sites for layer gliding and phase transitions. Therefore, the observed uniform in-plane charge density minimizes local stress concentrations, effectively suppressing the driving force for deleterious phase transformations and ensuring the structural integrity of the cathode during cycling. We have revised the related description in revised manuscript (lines 183-194).

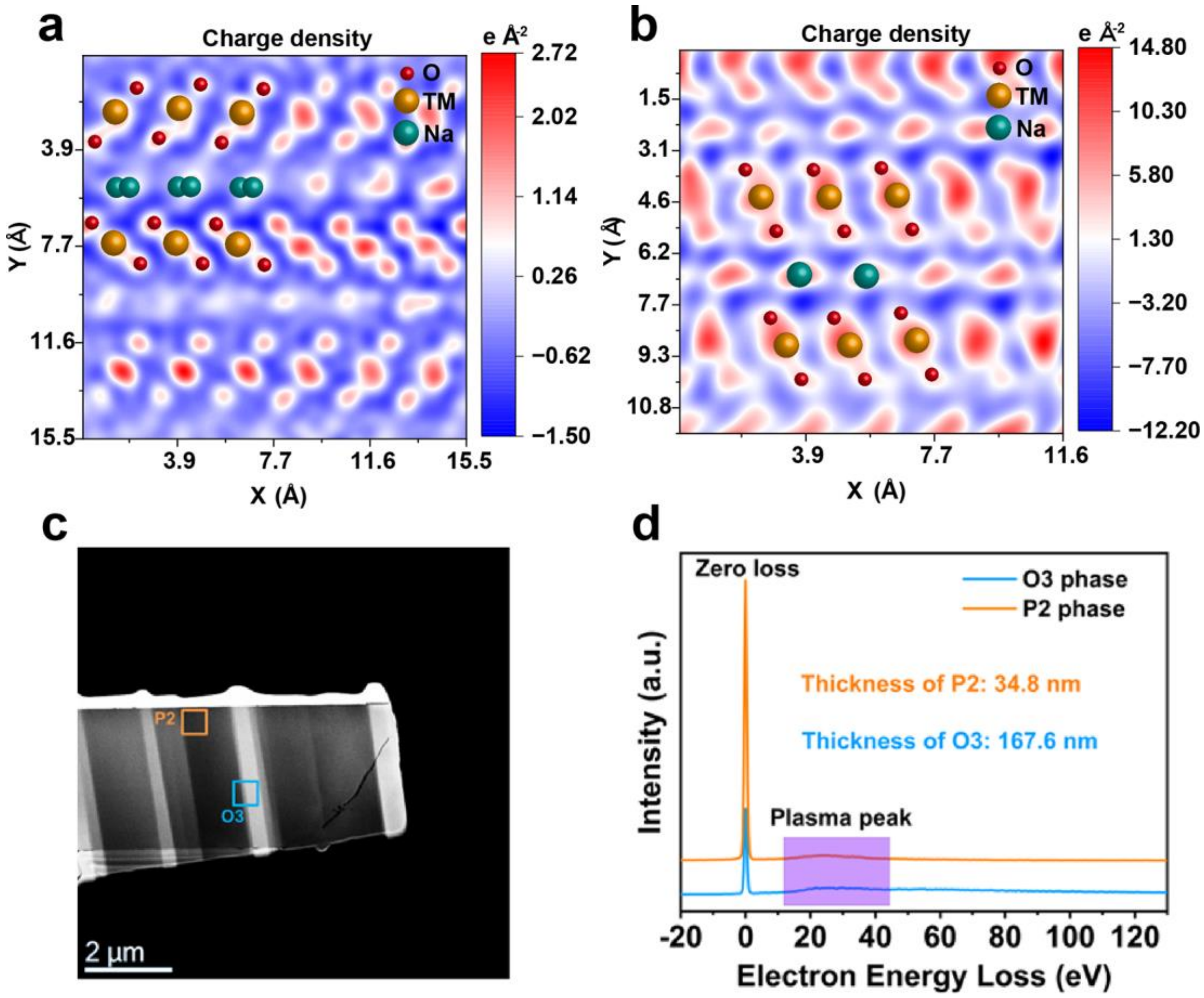

Figure R18 (Figure 2i and 2m) a, Atomic-level distribution of charge density of the P2 phase. **b,** Atomic-level distribution of charge density of the O3 phase. **c,** Cross-

sectional TEM image of NLCMMTO. **d,** Electron energy loss spectroscopy (EELS) spectra collected from the P2 and O3 regions.

5 From Fig. S22, the authors indicate that (line 206-207) The sodium ions all show the characteristics of three-dimensional diffusion… It is unclear why a planar geometry of the diffusion paths of Na ions shown in this figure is described as three-dimensional, as opposed to 2D.

Response: We sincerely thank the reviewer for pointing out this issue. Upon Figure S22, Na^+ diffusion in O3 phase proceeds through an octahedral to tetrahedral to octahedral hopping mechanism, forming a zigzag transfer pathway (Adv. Mater. 2022, 34, 2202137); however, they are indeed confined within the layer. Thus, the diffusion pathways shown correspond to planar Na^+ migration within the layer and therefore represent a two-dimensional diffusion network rather than a three-dimensional one. We have corrected the text in the revised manuscript to describe the Na^+ diffusion as two-dimensional.

We appreciate the reviewer's careful reading, which has helped us improve the clarity and accuracy of the manuscript: The sodium ions all show the characteristics of two-dimensional diffusion, and the migration energy barriers are 0.133eV and 0.115eV, respectively, indicating that the material has good ion transport performance in both phases. (lines 217-219)

6 For all the XRD maps in Fig. 3, and in SI, please indicate the meaning of the vertical direction. One could guess that it corresponds to charging from the bottom, up to 4.15 V in the middle, then discharging up to the top, but this should be clearly indicated. In SI, "intensity" should not be indicated as the vertical axis label in the same type of maps since these are contour plots.

Response: Thanks for your helpful suggestions. In revised manuscript, we have revised all in-situ synchrotron based XRD patterns.

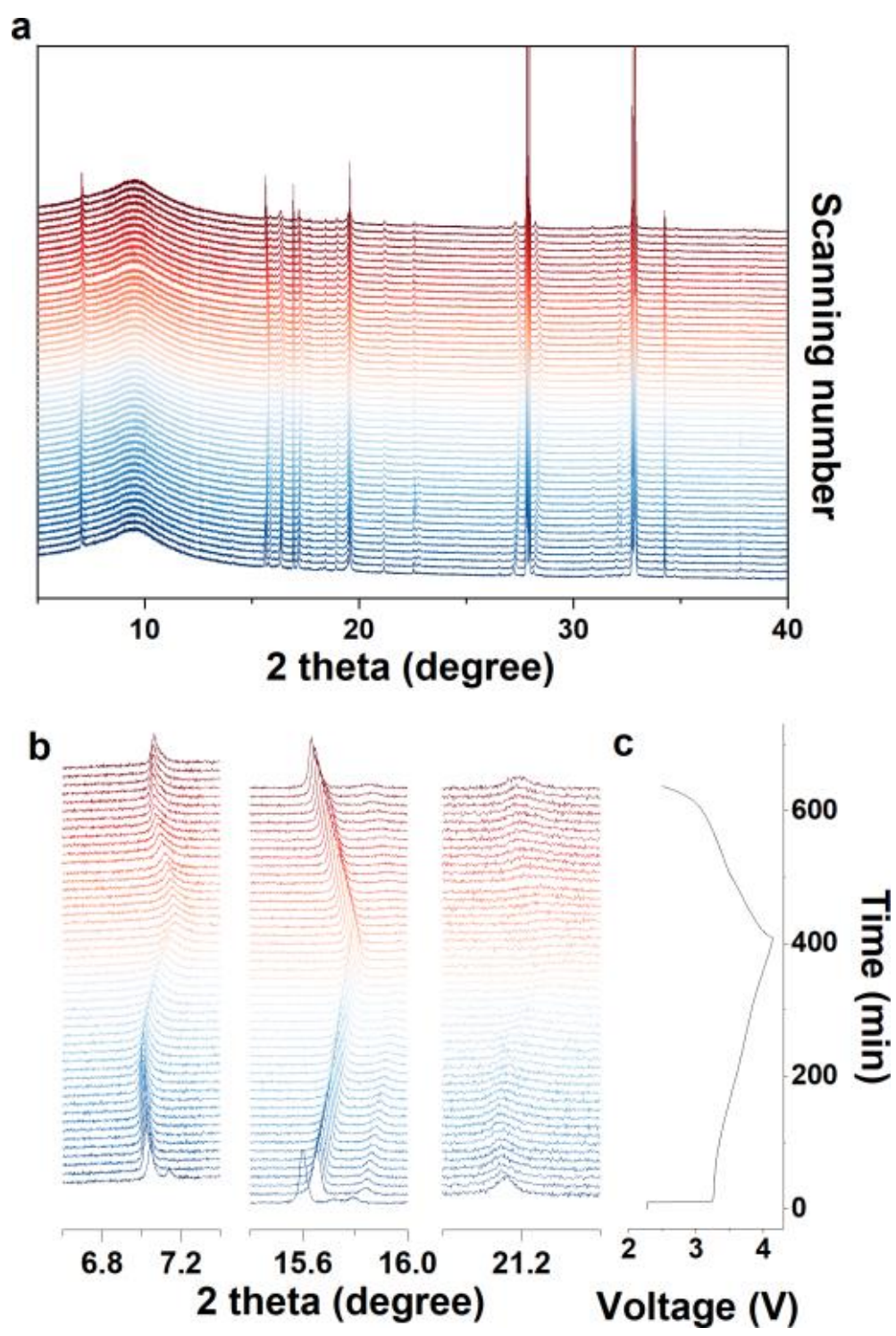

Figure R19 (Figure S32) a, In-situ synchrotron-based XRD pattern of NLCMMTO during the first charge/discharge at 0.2 C in the voltage range of 2.5- 4.15 V. **b,** The selected area in Figure R19a. **c,** Profile of charging/discharging of NLCMMTO at 0.2C.

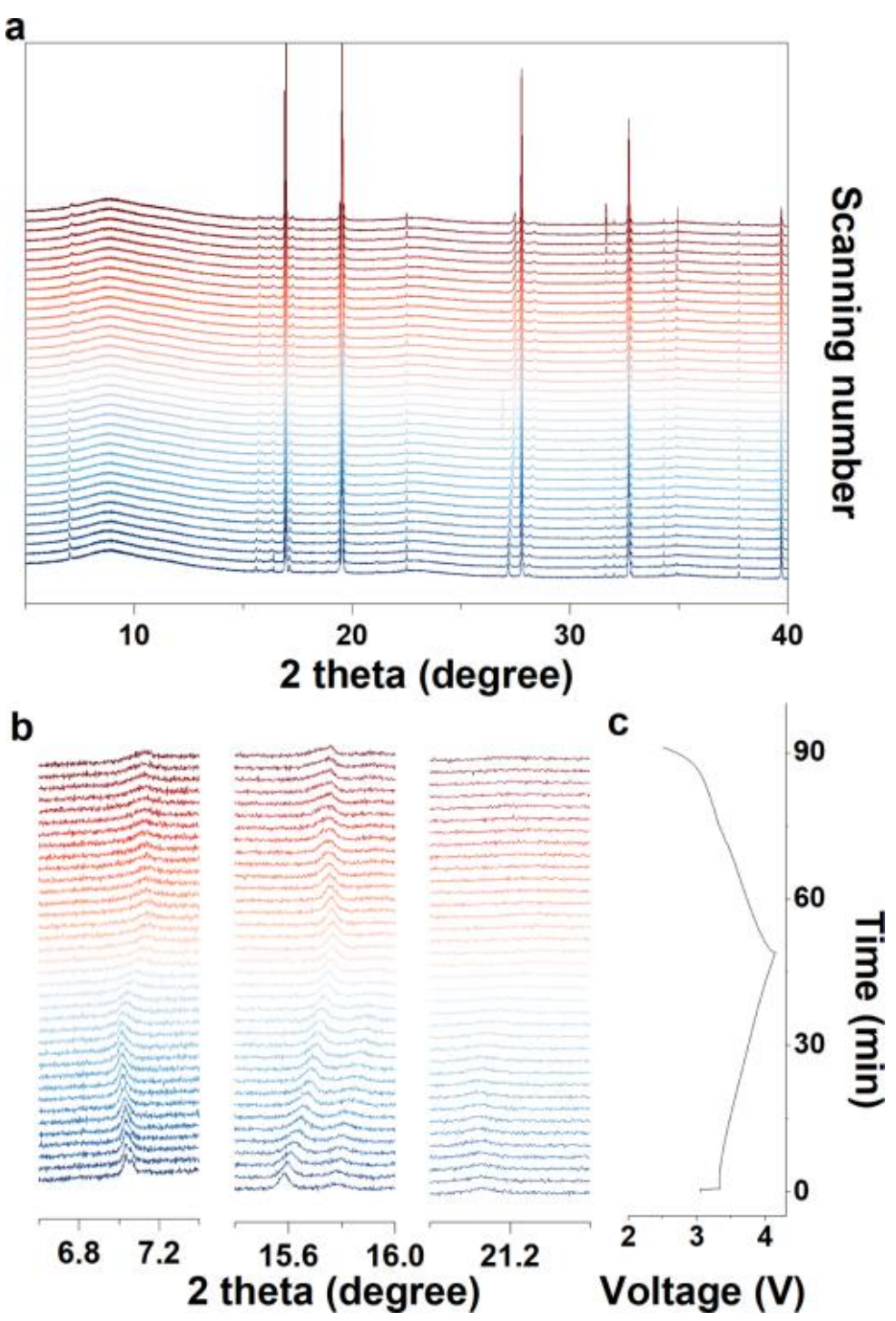

Figure R20 (Figure S34) a, In-situ synchrotron-based XRD pattern of NLCMMTO during the first charge/discharge at 2 C in the voltage range of 2.5- 4.15 V. **b,** The selected area in Figure R20a. **c,** Profile of charging/discharging of NLCMMTO at 2C.

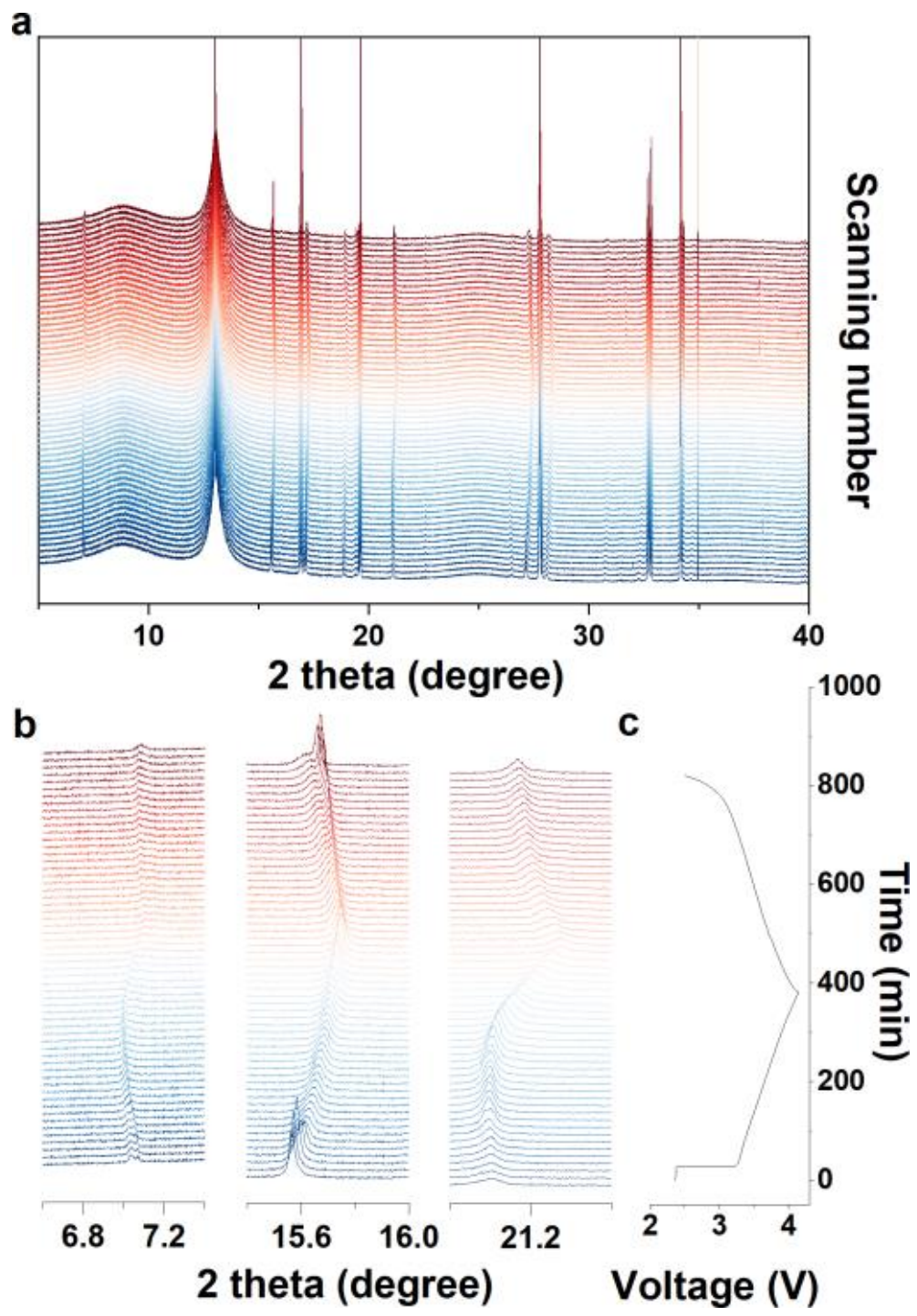

Figure R21 (Figure S28) a, In-situ synchrotron-based XRD pattern of NCMMTO during the first charge/discharge at 0.2 C in the voltage range of 2.5- 4.15 V. **b,** The selected area in Figure R21a. **c,** Profile of charging/discharging of NCMMTO at 0.2C.

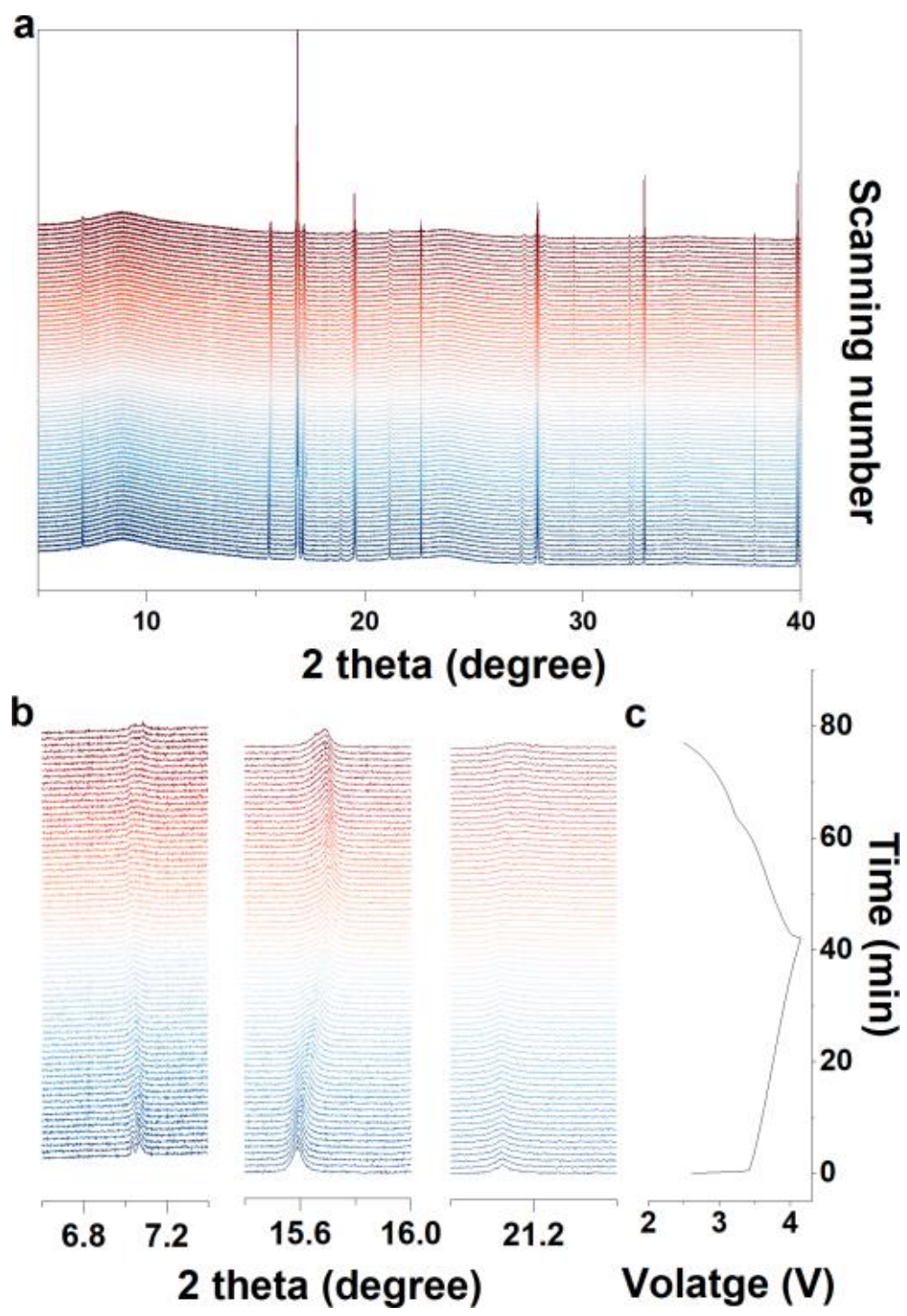

Figure R22 (Figure S30) a, In-situ synchrotron-based XRD pattern of NCMMTO during the first charge/discharge at 2 C in the voltage range of 2.5- 4.15 V. **b,** The selected area in Figure R22a. **c,** Profile of charging/discharging of NCMMTO at 2C.

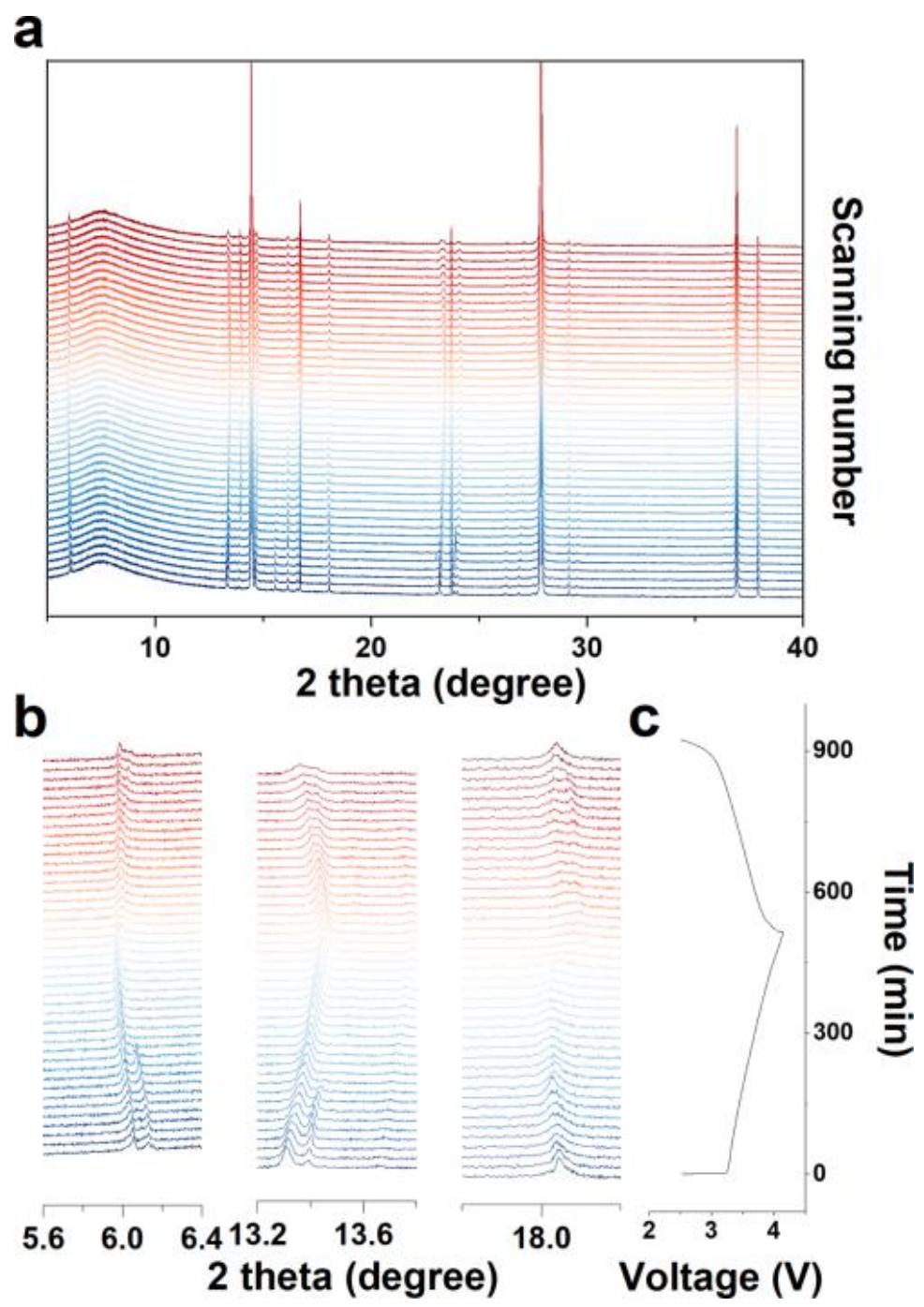

Figure R23 (Figure S24) a, In-situ synchrotron-based XRD pattern of NMMTO during the first charge/discharge at 0.2 C in the voltage range of 2.5- 4.15 V. **b,** The selected area in Figure R23a. **c,** Profile of charging/discharging of NMMTO at 0.2C.

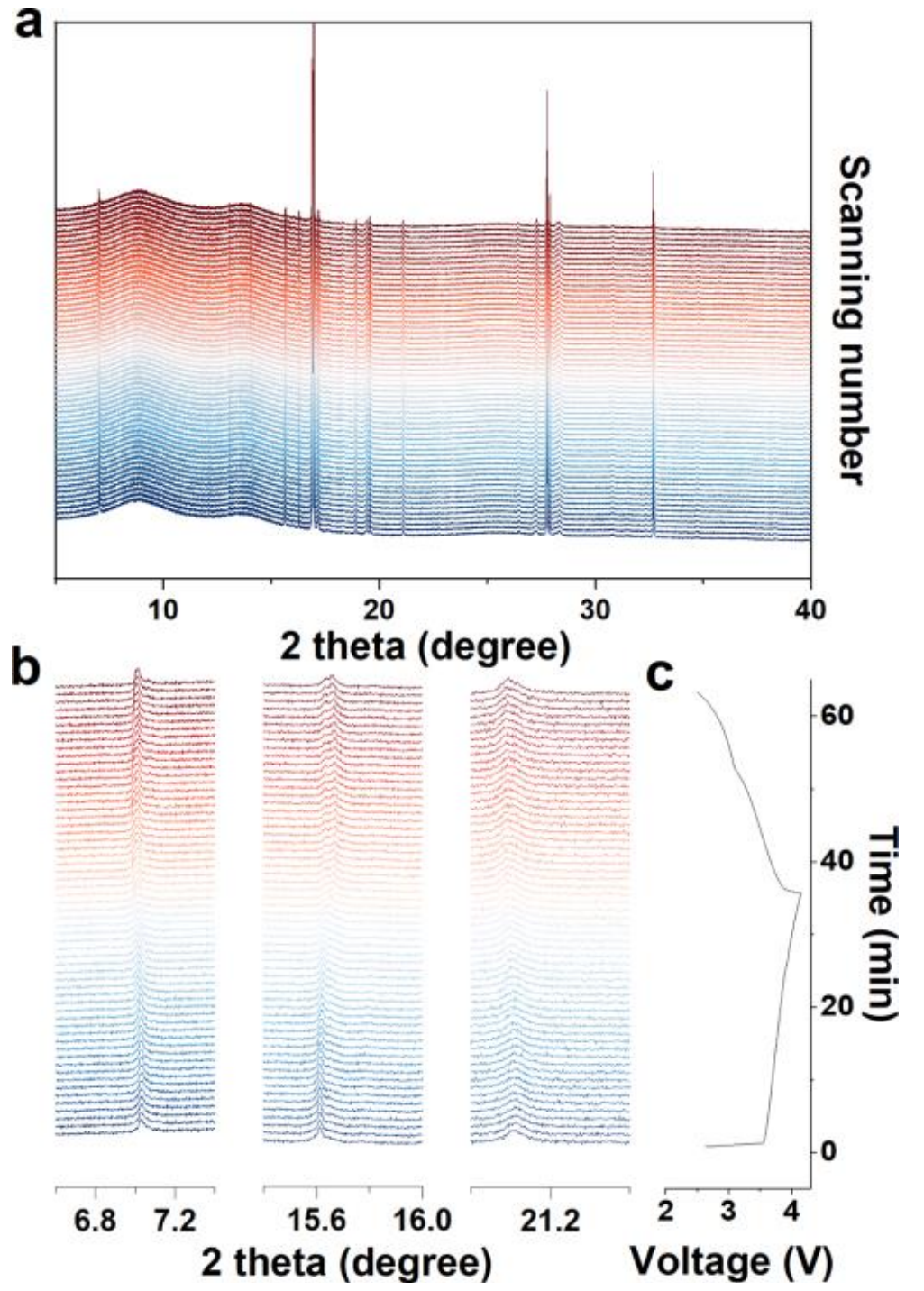

Figure R24 (Figure S26) a, In-situ synchrotron-based XRD pattern of NMMTO during the first charge/discharge at 2 C in the voltage range of 2.5- 4.15 V. **b,** The selected area in Figure R24a. **c,** Profile of charging/discharging of NMMTO at 2C.

7 The discussion of XRD peak positions from Fig. 3 maps and SI would benefit from maps over a narrower range of 2theta values to emphasize the variations of peaks. Thus far, the shifts are sometimes difficult to visualize. Furthermore, the data of the (104) peak could be included in the main text.

Response: Thanks for your helpful suggestions. In revised manuscript, we have revised Figure 3 by narrowing the 2θ range of XRD maps, which significantly enhances the visualization of peak position variations. Moreover, the (104) peak data have been added to the main text and discussed accordingly. We believe these changes make the peak shift analysis clearer and more convincing.

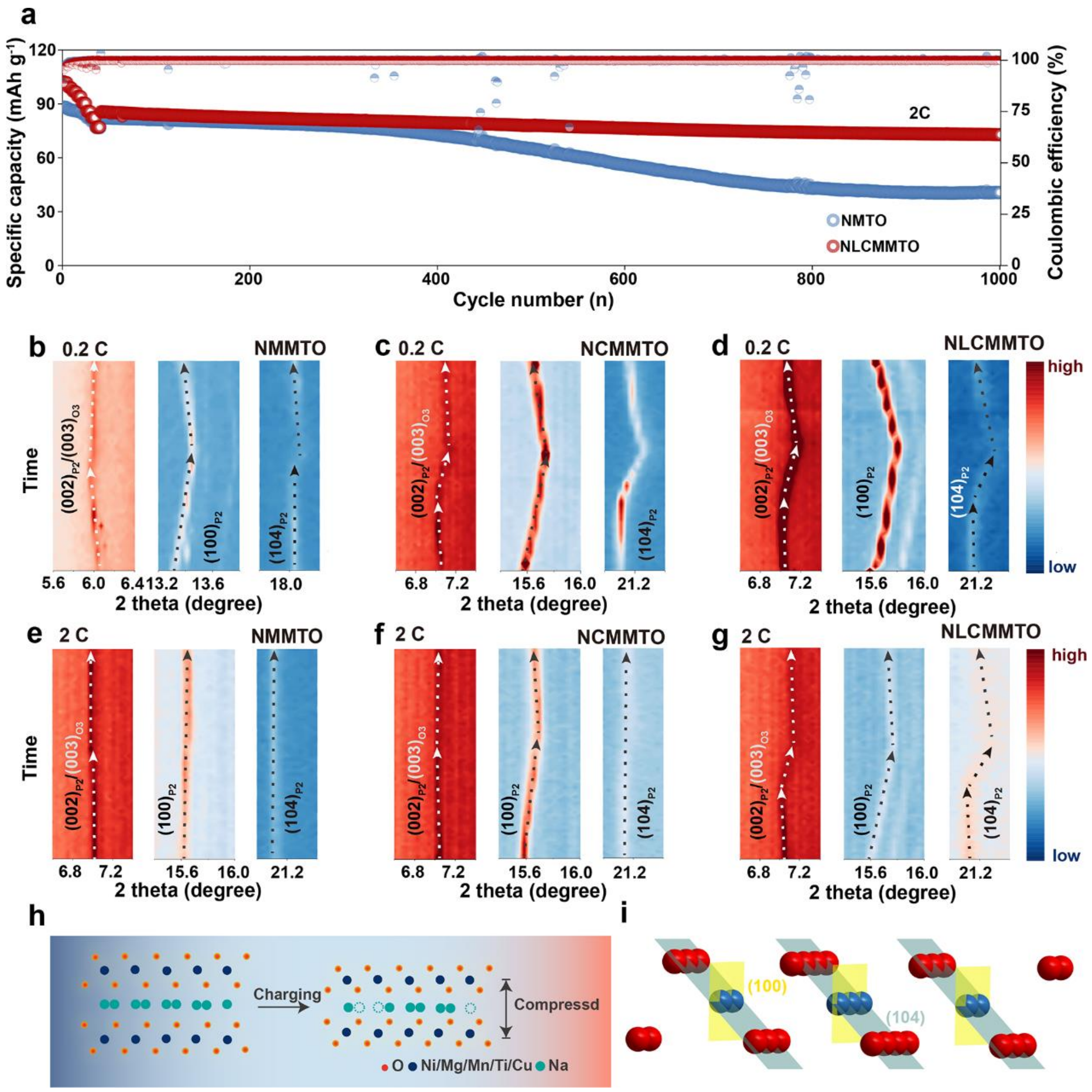

Figure R25 (Figure 3) a, electrochemical performance of NLCMMTO and NMTO **b-d,** selected in-situ synchrotron-based XRD pattern collected during the first charge/discharge in the voltage range of 2-4.15 V at 0.2C of NMMTO, NCMMTO, and

NLCMMTO. **e-g,** selected in-situ synchrotron-based XRD pattern collected during the first charge/discharge in the voltage range of 2-4.15 V at 2C of NMMTO, NCMMTO, and NLCMMTO. **h,** Schematic picture of self-adaptive volume behavior in NLCMMTO. **i,** (100) and (104) crystal plane diagram.

8 Fig. 4i is not discussed at all in the text. What is its purpose?

Response: Thank you for your careful reading and for pointing this out. We apologize for the lack of explanation of Figure 4i in the original manuscript. The original Figure 4i intent to highlight the advantage of wavelet transform analysis, which distinguish coordination environments with enhanced resolution in both k- and R-space. In response to the constructive suggestions from the first two reviewers, additional experiments have been conducted, and new data have been incorporated into the revised Figure 4 (**Figure R26**). Notably, SXR measurements have been introduced to further probe the electronic structure evolution. These results provide additional evidence for the role of Li in facilitating the reversible evolution of oxygen states.

On this basis, Figure 4 (**Figure R26**) has been substantially reorganized to integrate the newly acquired data and to improve the coherence of the presentation. A schematic illustration has been included to consolidate the mechanistic interpretation and to provide a clearer conceptual framework linking the experimental observations.

To maintain clarity and focus on the main text, the figure has been streamlined, with certain parts of the original content relocated to the Supporting Information (Figure S42 and S43). All associated figure captions and relevant sections of the manuscript have been revised to reflect these updates (lines 269-280).

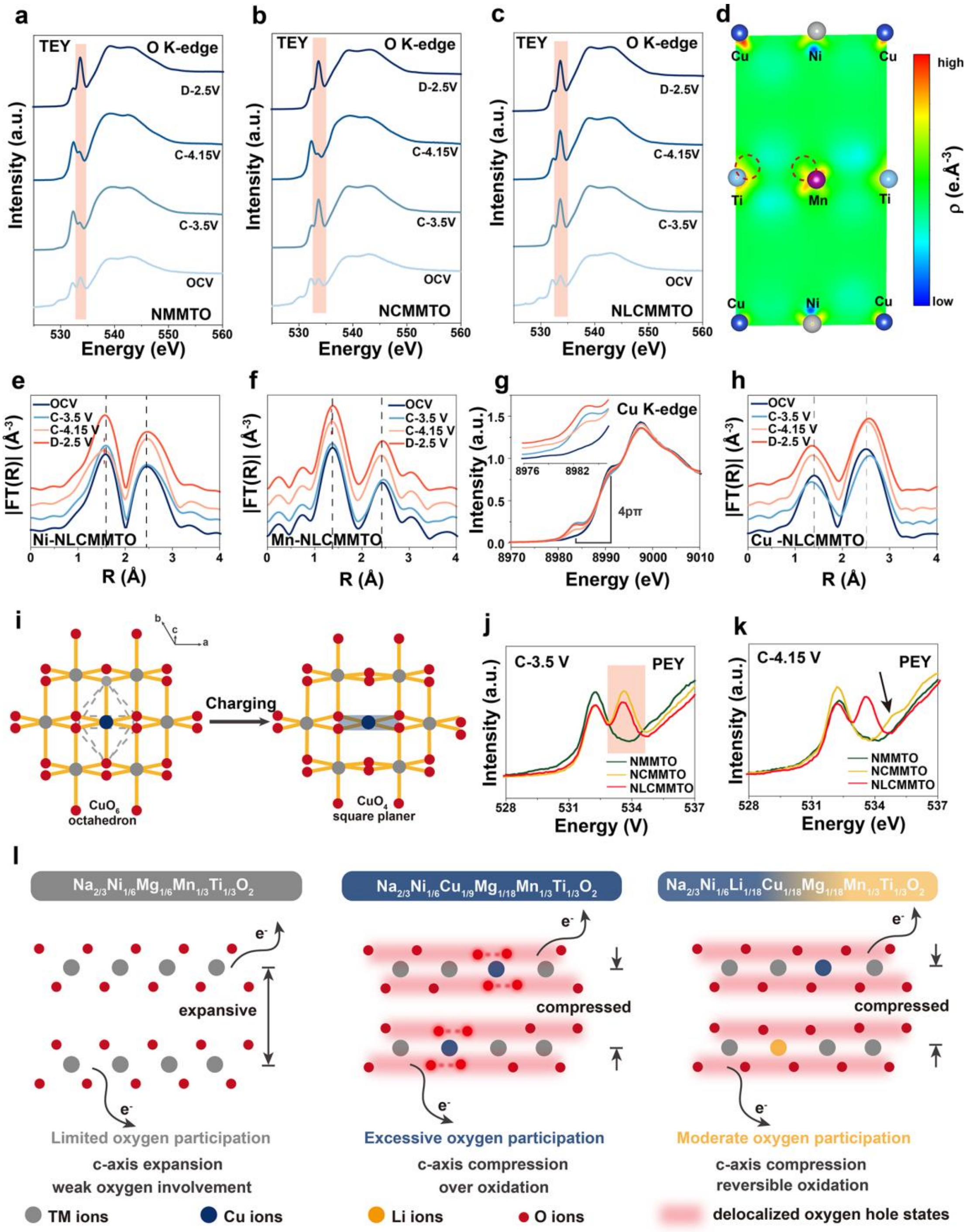

Figure R26 (Figure 4) TEY O K-edge XAS of **a,** NMMTO, **b,** NCMMTO, **c,** NLCMMTO electrodes from different charge states. **d,** Calculation of the charge density distribution of NCMMTO. **e,** Ni, **f,** Mn EXAFS in NLCMMTO at different charge state. **g,** Cu K-edge XANES in NLCMMTO at different charge state. **h,** Cu EXAFS in NLCMMTO at different charge state. **i,** Schematic image of structure evolution of Cu. PEY O K-edge XAS of NMMTO, NCMMTO, and NLCMMTO **j,** at

3.5V; **k,** at 4.15V. **l,** Schematic image of oxygen involved lattice evolution in layered oxides at highest voltage (4.15V).

9 The O-K ELNES in Fig. 4 is discussed line 259-261: "Such a broad 3d peak caused by an integration of the octahedral crystal field, exchange and the Jahn-Teller distortion from MnO6 has been maintained during the whole charging and discharging process in NMMTO demonstrating the valence change is Mn^{3+}/Mn^{4+}." The meaning of this sentence and the rationale behind the demonstration of valence change is difficult to comprehend. Please clarify

Response: We thank the reviewer for pointing out the lack of clarity in this sentence. The intention of this statement is to describe the origin of the broad feature in the O K-edge and its implication for the subtle variation in the Mn valence state. The peak at 529 eV in **Figure R27a** highlighted by dashed box related to O 2p states hybridized with Mn 3d orbitals, which remain during charging process suggesting the local electronic structure of Mn does not undergo a drastic transformation. This is consistent with Mn L-edge SXR spectra shown in **Figure R27b**. All results suggest that Mn valence state shows negligible variation during cycling.

In the revised manuscript, this part is modified in combination with the newly measured data (lines 270-280): From Figure 4a-4c, NMMTO, NCMMTO, and NLCMMTO show similar O K-edge SXR spectra (TEY mode) at the original state. Upon charging to 3.5V, a distinct peak emerges at ~533.6 eV in NCMMTO and NLCMMTO, which is associated with delocalized O 2p states that transitions arising from O 1s → π*, indicating the oxygen redox activity increases. Such specific O 2p states could act as a "charge buffer" within the TM-O network. This delocalization reduces the localization of electrons on transition metal (Ni Mn) d orbitals, thereby alleviating orbital degeneracy and suppressing Jahn-Teller distortions, which could be helpful to stabilize the structural integrity. As the increasing the voltage to 4.15V, the 533.6 eV feature could remain in NLCMMTO, indicating the stabilization of that state upon further oxidation. In contrast, the peak at 533.6 eV disappears for NCMMTO at the highest voltage.

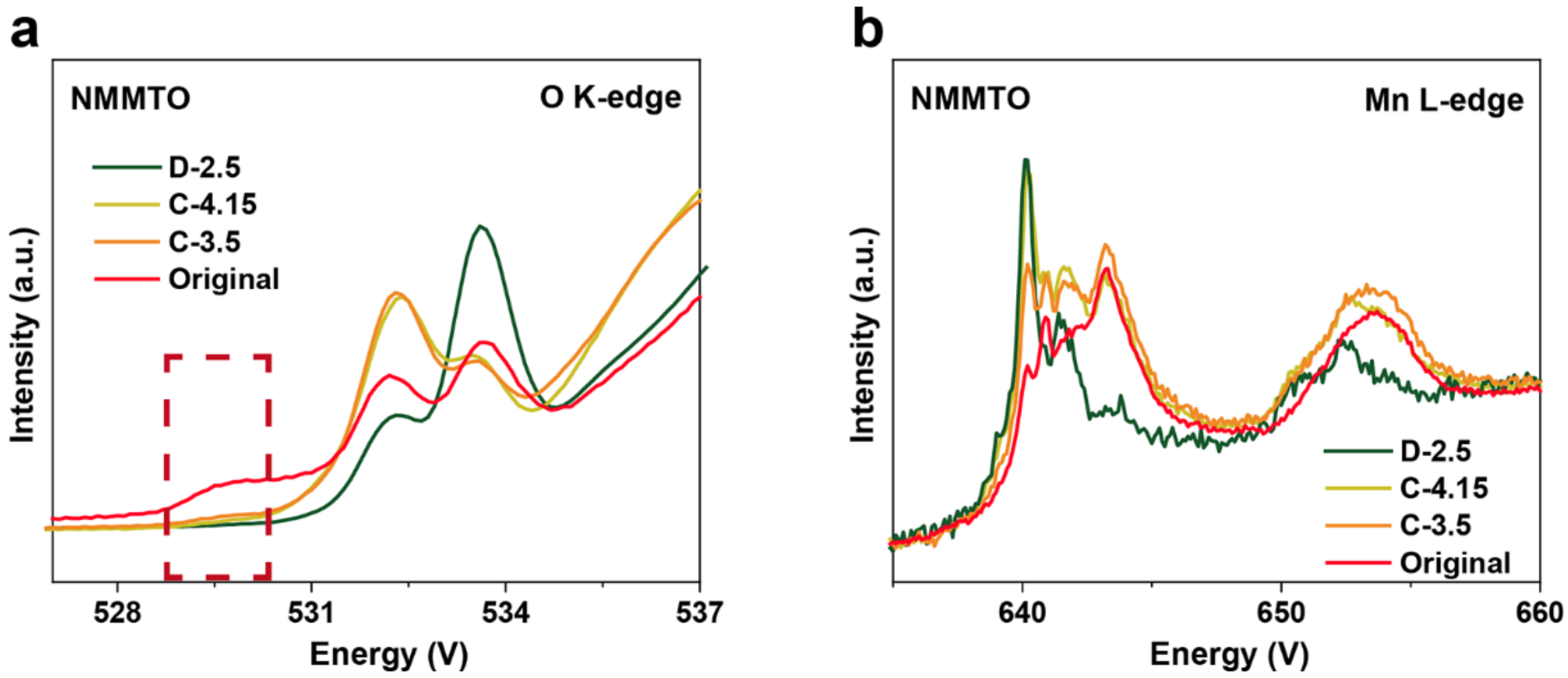

Figure R27 (Figure 5j) a, SXR spectra O K-edge of NMMTO electrodes at different states of charging. **b,** Mn L-edge of NMMTO electrodes at different states of charging.

10 In Fig. 5, atomic resolution HAADF images are shown at two charge states. Interplanar distances of 0.55-0.56 and 0.54 nm are indicated and mentioned later in the text. How meaningful are these values given that the cationic planes appear fuzzy and not as straight lines. It seems that these values are within the error.

Response: We thank the reviewer for this insightful comment regarding the interpretation of the interplanar distances derived from the HAADF images. We agree that there is an intrinsic uncertainty associated with directly measuring interplanar distances from these images. Therefore, we do not rely on HAADF analysis as a standalone quantitative method for determining subtle lattice changes.

Importantly, the values extracted from the HAADF images are primarily intended to provide a qualitative visualization of the structural evolution at different states of charge. The observed slight contraction of the interlayer spacing at high voltage is consistently supported by our in-situ synchrotron based XRD results (**Figure R28**), which provide statistically averaged and more precise structural information. The XRD analysis clearly indicates a reduction in the interlayer distance upon charging to the highest voltage, in good agreement with the trend observed in the HAADF images.

Thus, rather than serving as an isolated measurement, the HAADF observations and XRD results are complementary and mutually reinforcing. The agreement between these two independent techniques strengthens the reliability of the structural interpretation.

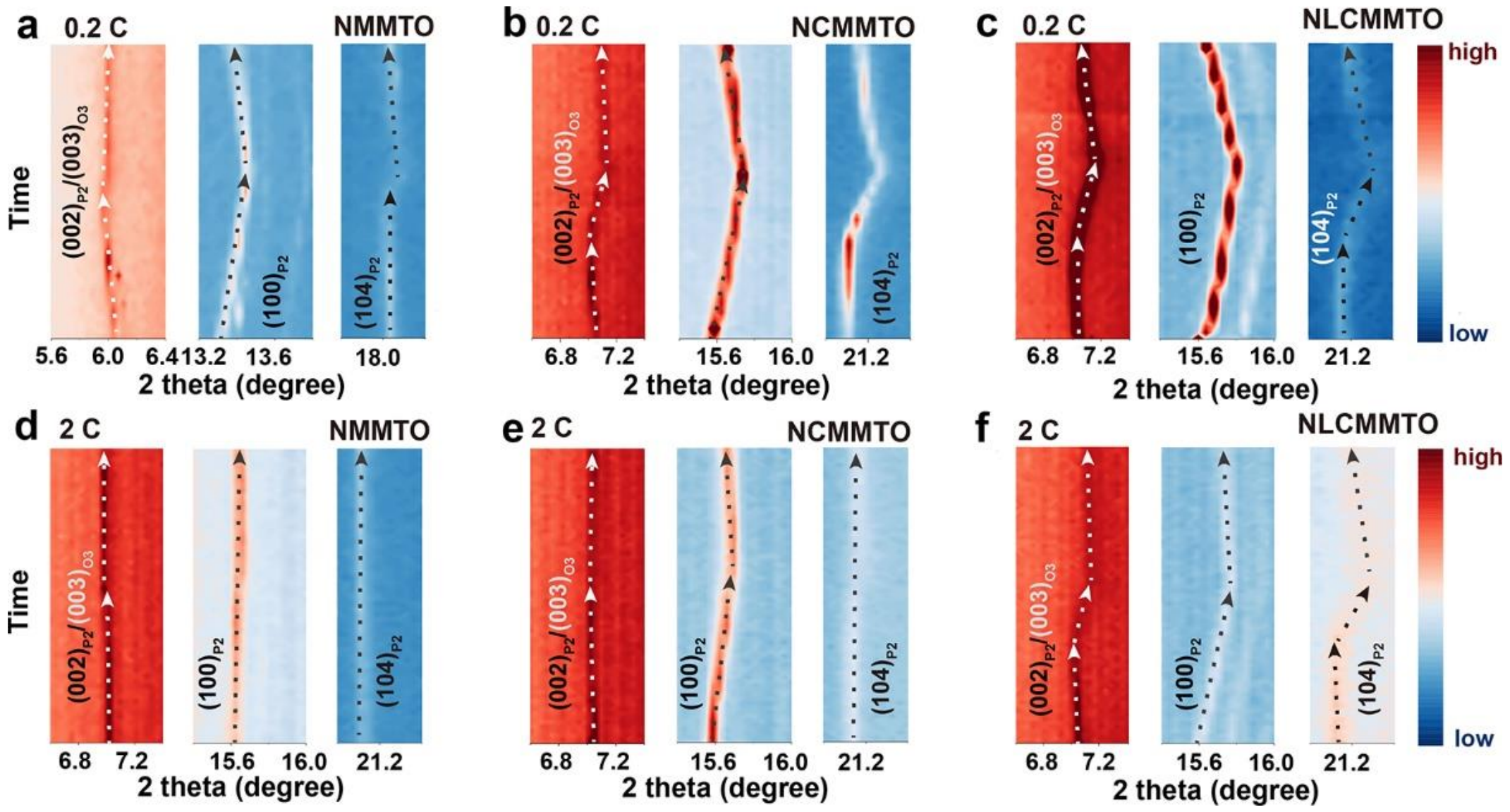

Figure R28 (Figure 2b-2g) a-c, selected in-situ synchrotron-based XRD pattern collected during the first charge/discharge in the voltage range of 2-4.15 V at 0.2C of NMMTO, NCMMTO, and NLCMMTO. **d-f,** selected in-situ synchrotron-based XRD pattern collected during the first charge/discharge in the voltage range of 2-4.15 V at 2C of NMMTO, NCMMTO, and NLCMMTO.

11 The Mn-K and Ni-K edges in Fig. 5 are difficult to visualize for all states of charge given that they overlap, which makes the description of the results line 307-337 difficult to follow. Please make sure that all spectra are visible.

Response: Thanks for your valuable suggestions. In revised manuscript, we have carefully revised the figure to improve its clarity and readability. Specifically, the spectra have been reprocessed and replotted to ensure that all states of charge are clearly distinguishable. We have adjusted the presentation by optimizing the offset and contrast between individual spectra, allowing the evolution of both Mn and Ni edges to be more readily visualized in Figure 5 (**Figure R29**). In addition, key features relevant to the discussion (lines 307-337 in the original manuscript) have now been explicitly highlighted in the revised Figure 5. Guiding markers and annotations have been introduced to draw attention to the spectral regions where the most significant changes occur, thereby facilitating a more straightforward interpretation of the redox behavior.

The related description has been revised (lines 329-341): From Figure 5c, the shape of Mn pre-edge shows broad and weak which represents that Mn is primarily

located in octahedral coordination sites. At the beginning of charging duration, there is slightly left shift of peak at 6559 eV together with decreased intensity of C-3.4V and C-3.8V pre-edge, suggesting the subtle reduction of Mn, until charging to 4.15V, the intensity of pre-edge peak is increased. At the following discharging process, the intensity of peak decreases with reducing Mn valence. During the entire Na^+ insertion and extraction process, the coordination environment of Mn remains stable evidenced by the similar shape of pre-edge features observed at different charge states. Ni K-edge XANES spectra reveal a change in the Ni environment at 3.8V, evidenced by a continuous change in the shape of the absorption edge. Additionally, Ni XANES spectrum at the end of discharge, returns to exactly the same position as in that of the pristine material, showing the highly reversible ability of Ni local environment. Notedly, the spectrum of Ni of D-3.5V seems like be in a higher oxidation state than its D-3.8V in reduction process due to its slightly right shift.

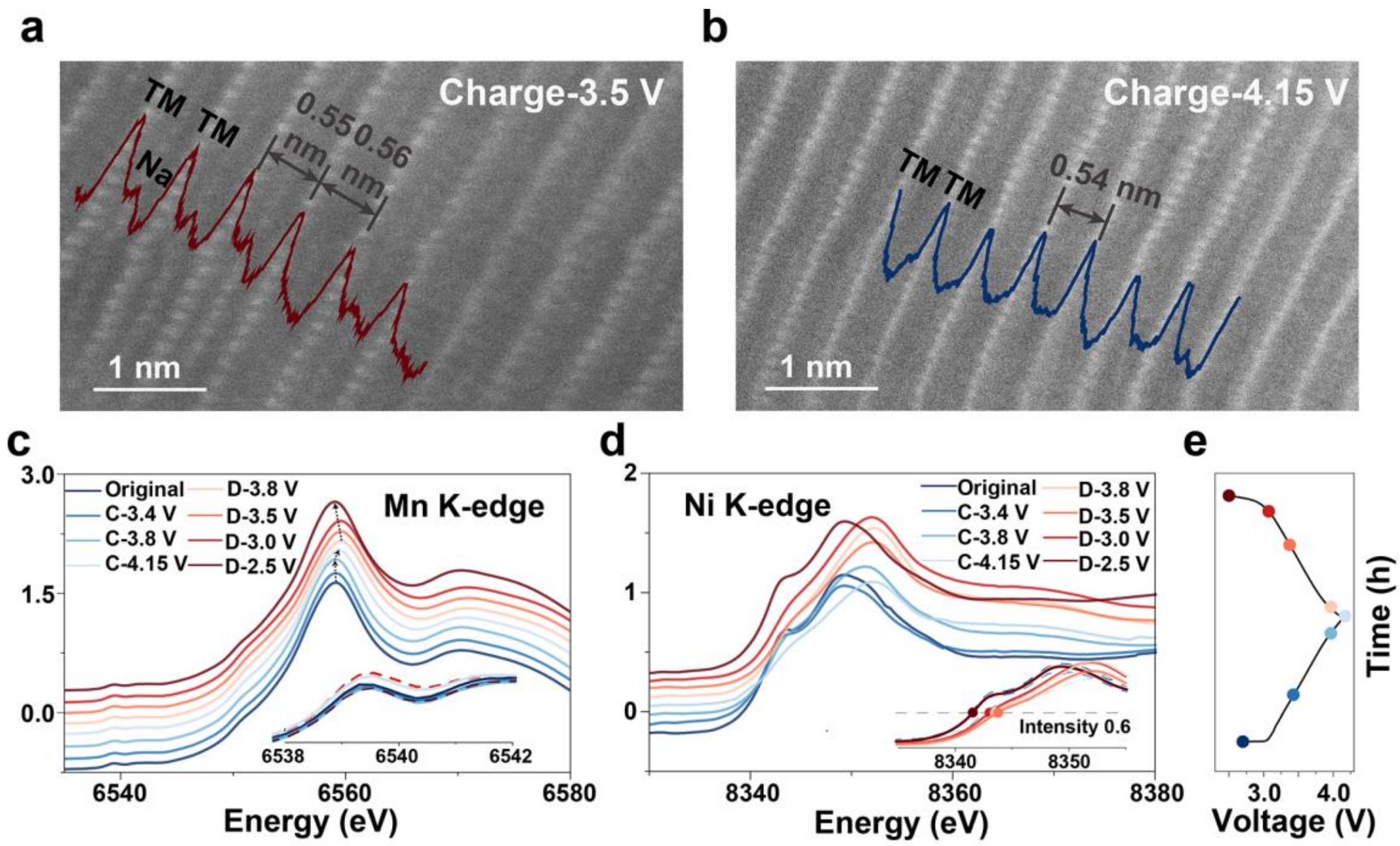

Figure R29 (Figure 5a-5e) HAADF-STEM pattern of NLCMMTO at charging **a,** 3.5V, **b,** 4.15V. **c,** Mn K-edge, **d,** Ni K-edge of NLCMMTO at different charge states. **e,** the profile of charge/discharge at 0.1 C.

12 Line 332, it is indicated that the NLCMMTO exhibits an unexpected reduction of Mn from the +4 (OCV) to the +3(C-3.5V) upon charging to 3.5 V… Is it a typo for +2

valence at 3.5 V? The Mn-L3 edge in Fig. 5f shows a rather typical Mn2+ fine structures at 3.5 V, which is also indicated line 268.

Response: We thank the reviewer for the careful analysis of the Mn L-edge and for pointing out this inconsistency.

We agree that the valence of Mn is ambiguous, thus the standard samples of MnO, Mn_2O_3 and MnO_2 have been added to revised Figure 5 (**Figure R30**). Although there is ~639 eV associated to the Mn^{2+} feature in NLCMMTO at charging to 3.5V, the peaks at 640.5 eV, 640 eV and 642.3 eV corresponding to Mn^{3+} and Mn^{4+} still exist at 3.5V, suggesting the Mn is mixed valence and undergoes a slight reduction. In the revised manuscript, we clarify that Mn is reduced from Mn^{4+} toward lower valence states, and likely exists in a mixed-valence state (Mn^{2+}/ Mn^{3+}/Mn^{4+}) at this stage.

The relevant discussion has been revised (lines 346-349): Compared with NMMTO, where the Mn peak shape and intensity remain seldom changed, NLCMMTO exhibits an unexpected reduction of Mn from the +4 (OCV) to the mixed valence of +2/+3/+4 upon charging to 3.5 V, which indicates a reduction process, followed by oxidation to the highest valence state at 4.15V.

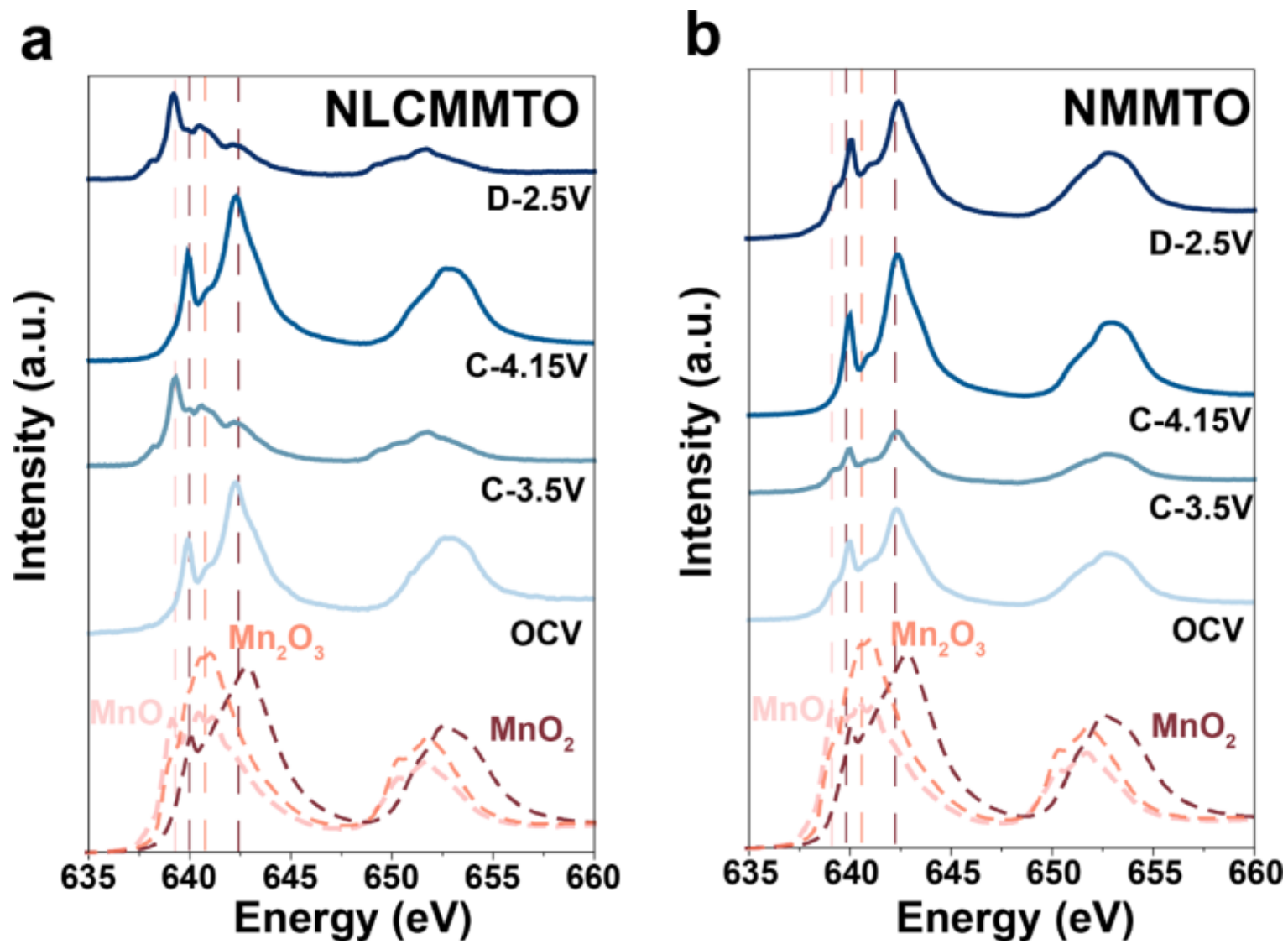

Figure R30 (Figure 5f and 5g) Mn L-edge of **a,** NLCMMTO, **b,** NMMTO electrodes at different states of charging.

13 The Mn-K edge shown in Fig. 5 shows limited potential to be interpreted in terms of valence and structural change. The Ni-K edge is better in this respect, but XPS is

provided. It is surprising that the authors have not included data for the SXR Ni-L23 edge and XPS Mn 2p (was it recorded?), which would be useful to discuss the valence changes, complementarily to SXR Mn-L23, XPS Ni 2p and the XAS K edges, and support the discussion about the participation of Mn and Ni in the charge compensation mechanism.

Response: Thanks for your helpful suggestions. We agree that Ni $L_{2,3}$-edge and XPS Mn 2p can provide valuable insight into the electronic structure of transition metal ions in cathode materials.

Following this suggestion, we carefully examined the Ni $L_{2,3}$-edge spectra that were collected for NLCMMTO at different states of charge. These spectra show systematic and reproducible evolution during charging. Specifically, the higher energy feature of the Ni L_3-edge shifts progressively with increasing voltage (**Figure R31a**), while the intensity of higher energy peak of Ni L_2-edge exhibits a similar trend. These changes indicate a modification of the Ni electronic structure as the electrode is charged. Notably, the low-energy main peak of the Ni L_3-edge is less pronounced than that of the high-energy peak within the experimental resolution. The Ni L-edge spectra reflect transitions into unoccupied Ni 3d states and are strongly influenced by crystal field interactions, multiplet effects, and metal-ligand hybridization. As a result, the spectral line shape typically represents a superposition of multiple electronic configurations and local environments. During charging process, Ni may evolve through heterogeneous local coordination environments rather than undergoing a uniform valence shift. Under these conditions, the dominant L_3 component can remain nearly constant in position, while higher energy multiplet features which are more sensitive to ligand-field strength and charge transfer energy show detectable changes. In addition, the strong covalency between Ni 3d and O 2p orbitals in oxide cathodes can lead to charge compensation mechanisms that involve not only changes in Ni oxidation state but also modifications of transition metal and oxygen hybridization. These effects primarily influence the higher energy spectral features of the L_3 and L_2 edges rather than producing a simple rigid shift of the main peak. Thus, these observations combined with change of Ni K-edge support the conclusion that Ni participates in the charge compensation process.

Regarding the reviewer's suggestion to include Mn 2p XPS, we note that Mn 2p spectra in transition metal oxides typically exhibit strong multiplet splitting and satellite features, which can make small valence changes difficult to quantify reliably, particularly for composite electrodes where surface sensitivity may influence the spectra shown in **Figure R31c**. In contrast, Mn L-edge spectroscopy provides a more direct probe of the Mn 3d electronic configuration and is widely used to monitor Mn valence evolution in cathode materials. To clarify these points, we have added a discussion of the Ni L-edge results and their implications for the charge compensation mechanism in the revised manuscript.

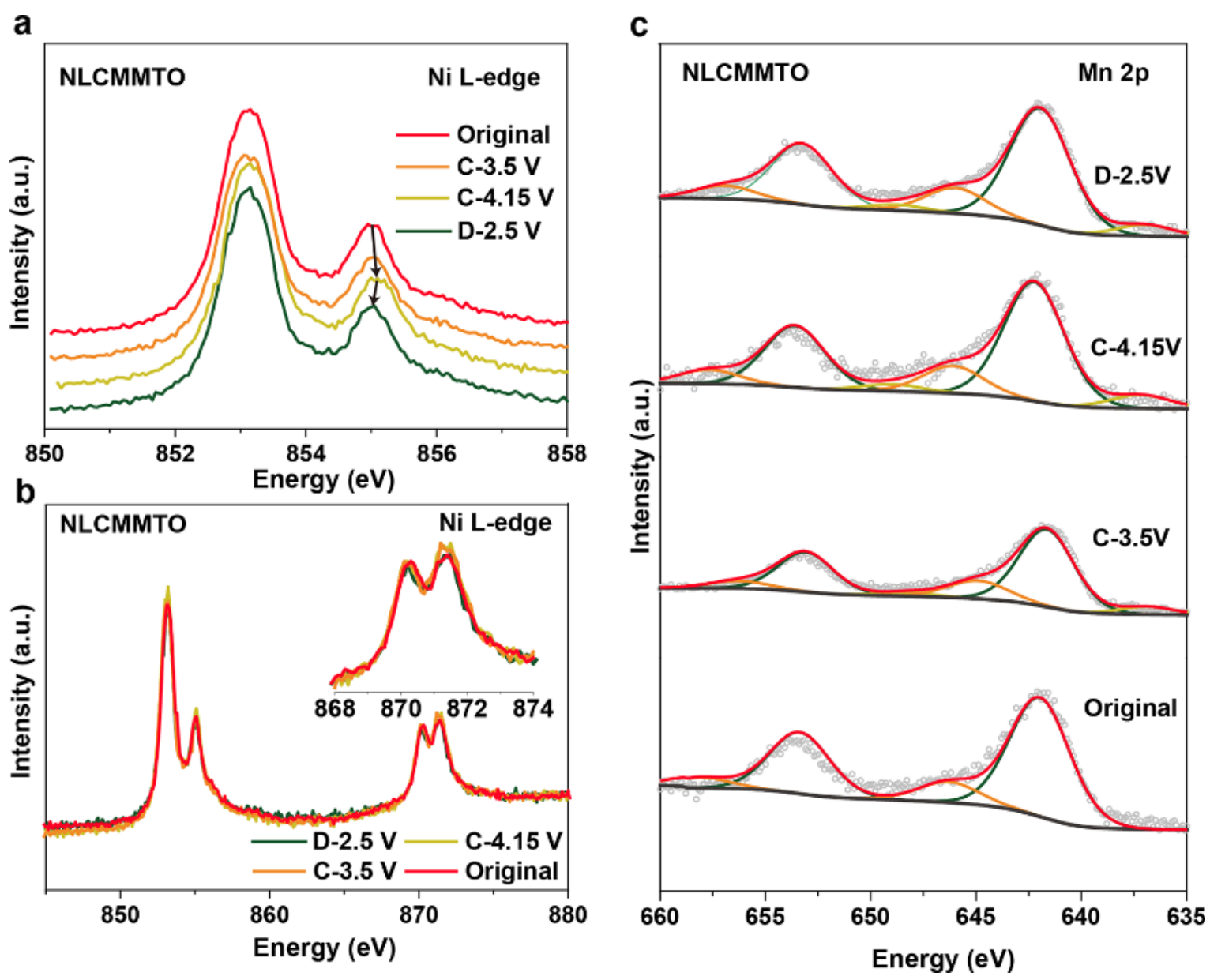

Figure R31 (Figure 5h and S46) a, SXR spectra Ni L_2-edge of NLCMMTO electrodes at different states of charging. **b,** SXR spectra Ni L-edge of NLCMMTO electrodes at different states of charging. **c,** Mn 2p XPS spectrum of NLCMMTO electrodes at different states of charging.

14 Line 341: "This shift partially reverses after discharging to 2.5 V, suggesting a quasi-reversible Ni redox process." The reversal is clearly partial and therefore the redox process cannot hardly be called quasi-reversible…

Response: Thanks for your insightful comment. The partial irreversibility of Ni 2p XPS results likely arises from surface reactions with the electrolyte, as XPS is inherently surface sensitive. To further clarify the intrinsic redox behavior, we refer to the ex-situ XAS experiment in which the electrochemical process was stopped at different voltages,

and the XAS spectra of the electrode sheets were directly collected from the battery. The Ni K-edge spectra are shown in **Figure R32**. In this case, after completing the full charge-discharge process, the Ni XAS spectral features (red line), including both the line shape and edge position, return to their initial state and nearly overlap with those of the pristine electrode (blue line). This recovery indicates that the electronic structure and local coordination environment of Ni are largely restored after cycling, suggesting that the Ni redox process proceeds in a highly reversible manner under near equilibrium conditions. So, we would describe this behavior "Ni reversible redox".

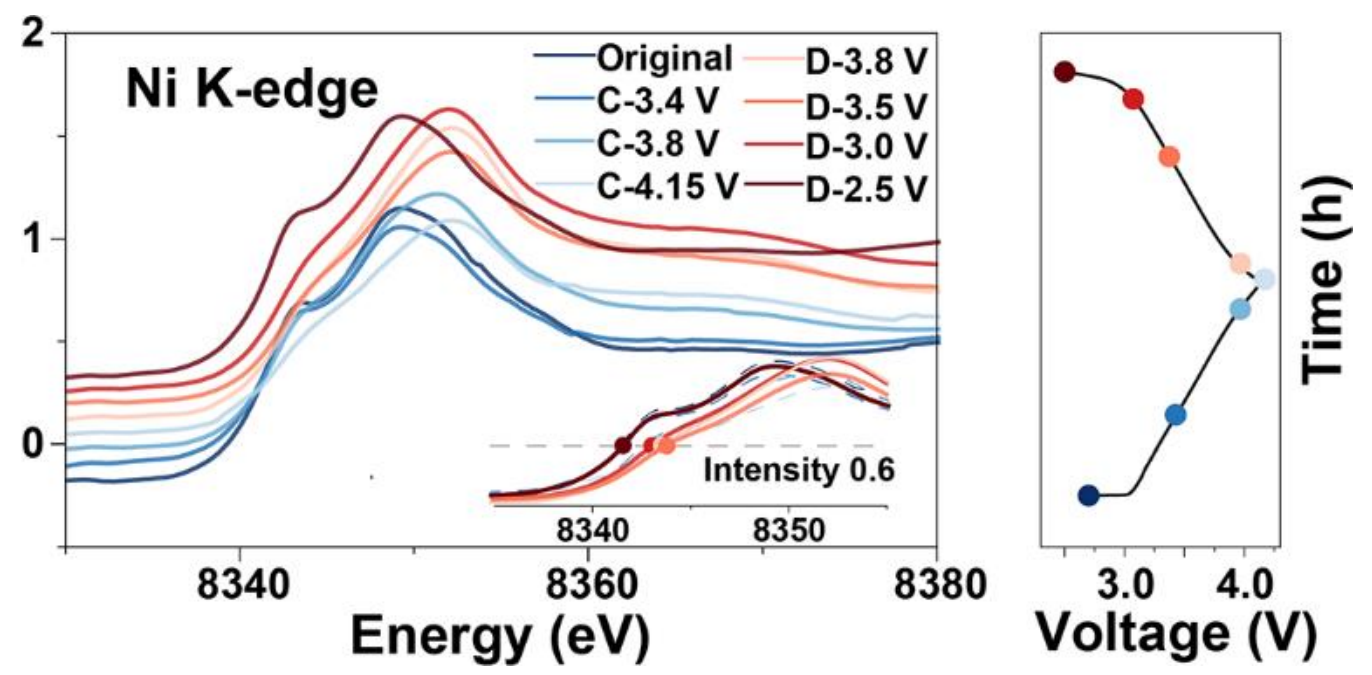

Figure R32 (Figure 5d) ex-situ XAS spectra of Ni K-edge of NLCMMTO electrodes at different voltage in battery.

15 The discussion about the DOS leads to the statement "the reduction activity of Ni ions is greater than that of Mn ions." I miss a justification for this interpretation from the DOS presented.

Response: We thank the reviewer for this valuable comment. In the original manuscript, the term "reduction activity" was used to describe the relative tendency of transition-metal states to participate in electron exchange, which is used in a broader sense to describe the ability of transition-metal states to accommodate electron transfer, and it is more rigorously interpreted as redox activity in the context of desodiation.

In the desodiation process, the DOS near the Fermi level reflects the availability of electronically accessible states that can participate in charge compensation. The Ni 3d states, being closer to the Fermi level, can more readily undergo electron depletion and reoccupation, indicating higher electronic (redox) activity compared to Mn. To improve clarity and avoid ambiguity, we have revised the manuscript to replace "reduction activity" with "redox activity" (or "electronic activity") in line 369.

16 Fig. 6c: the fine structure at ~8335 eV is not present in the Ni-K edges shown in Fig. 5. Why such difference?

Response: Thanks for your insightful comment. The spectra in Figure 5d and Figure 6c were acquired under different measurement conditions. In Figure 5d, the spectra were collected after interrupting the electrochemical process at selected voltages, representing near equilibrium states. In contrast, Figure 6c was obtained under continuous cycling, where the spectra reflect the instantaneous response under dynamic conditions. A weak feature around ~8335 eV is only resolved under operando conditions. While its exact origin cannot be unambiguously determined from the present data, such features are commonly associated with pre-edge or shoulder contributions that are sensitive to local symmetry and 3d-4p hybridization. One possible explanation is that, under operando conditions, the system is continuously driven out of equilibrium, which may enhance subtle electronic or local structural fluctuations that are not captured in quasi in situ measurements. These dynamic effects could make such weak features more discernible.

We also note that such subtle features can be influenced by experimental conditions and data processing. Importantly, this feature does not evolve with electrochemical state, indicating that it is not directly associated with the redox evolution. Such subtle features are known to be sensitive to experimental conditions and are often attributed to minor pre-edge or shoulder contributions related to local symmetry and hybridization. We therefore do not rely on this feature for interpretation. Instead, the analysis focuses on the systematic shift of the Ni K-edge position, which is consistently observed under both measurement conditions and reflects the continuous evolution of the average Ni oxidation state during cycling.

17 How is the oxidation state quantified from the Ni-K edge in Fig. 6 (line 363)?

Response: Thanks for the important comment. We would like to clarify that the interpretation of the Ni K-edge evolution in Figure 6 is based on the observed shift of the absorption edge, rather than a direct assignment of oxidation states. As shown in Figure 6c, the spectra mainly exhibit a set of shifts in edge position, with increasing voltage, the absorption edge gradually shifts toward higher energy, indicating an

increase in the average oxidation state of Ni. Given that the spectral line shape remains essentially unchanged, the description in the manuscript has been revised to better reflect this observation (revised manuscript lines:383-384):

"During charging, the Ni K-edge position continues to shift to higher energy at the 4.15 V cut-off (Figure 6c), indicating a continuous increase in the average oxidation state of Ni."

18 The link between covalency and bond strength could be explicited further (line 370).

Response: Thanks for this valuable comment. We have revised the manuscript to further clarify the relationship between covalency and bond strength.

Specifically, increased covalency reflects enhanced orbital hybridization between metal d states and ligand p states, strengthening the chemical bonds. This relationship has been widely discussed in previous studies (e.g., J. Am. Chem. Soc. 2024, 10.1021/jacs.4c07459; Nat. Rev. Chem. 2024, 10.1038/s41570-024-00641-y; Nat. Commun. 2025, 10.1038/s41467-025-58654-0). In this study, this enhances the covalency of the TM-O bonds through stronger hybridization between TM d states and O 2p states. The increased covalency promotes electron delocalization and reduces the localization of TM-O interactions, thereby weakening the effective ionic character of the bond. As a result, the lattice becomes more flexible, which lowers the activation energy barrier for Na^+ migration.

The corresponding discussion has been added to the revised manuscript (lines 387-393): This enhances the covalency of the TM-O bonds through stronger hybridization. Such increased covalency originates from enhanced orbital overlap, which governs the strength and nature of chemical bonding. Consequently, the modified bonding character facilitates Na^+ migration by reducing the energy barrier, and then achieving the improvement of rate performance.

Minor comments:

19 Line 270: "…shows the oxygen going through structural change". The material can undergo structural changes, but not the oxygen atoms. Please rephrase.

Response: Thanks for pointing this out. We have modified and rephrase the description of oxygen evolution in the revised manuscript lines 269-277.

20 Line 274: the electron is more delocalized…" The electrons (or the electronic density) are more delocalized.

Response: Thanks for pointing this out. We have revised the sentence to clarify that it is the electrons that become more delocalized, rather than referring to a single electron. The description has been corrected accordingly in the revised manuscript lines 284: The electron density is more delocalized.

21 In the schematic in Fig. 6h, a proper description of the corresponding states of charge is necessary to comprehend the meaning of the schematic.

Response: Thank you for this helpful and constructive comment. We appreciate the reviewer's careful reading and valuable suggestions, which has helped improve the clarity of our manuscript. In response, we have added a more detailed description of the corresponding states of charge in the schematic shown in Figure 6h, in order to better guide the reader and enhance the interpretability of the figure. The relevant information has been incorporated into the revised manuscript.

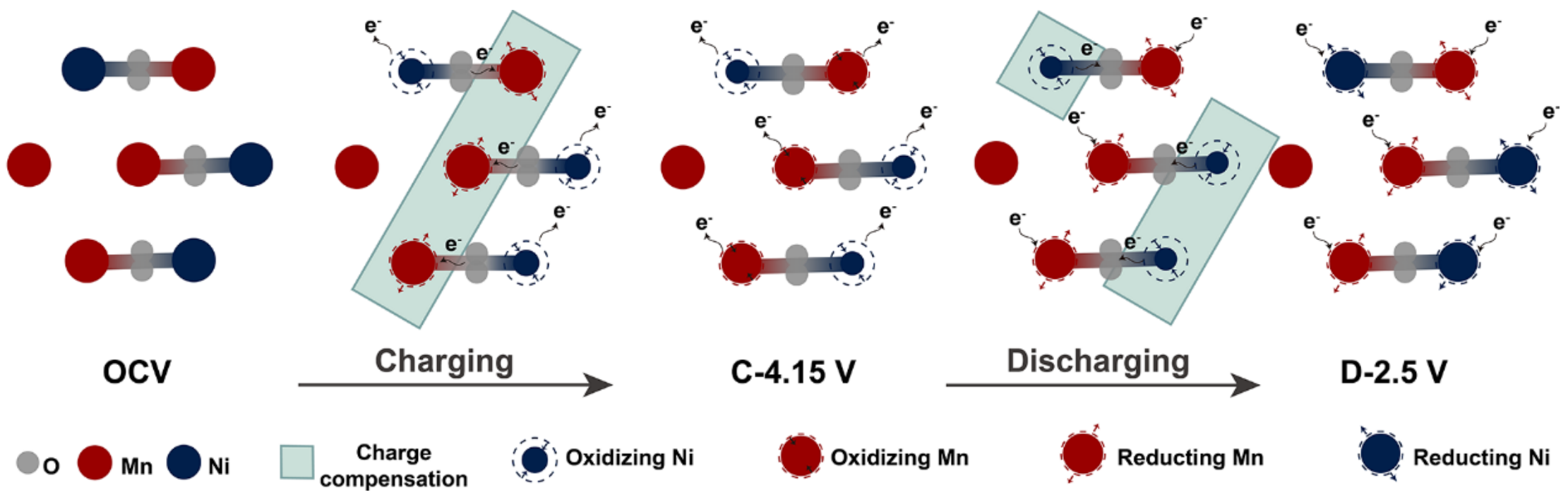

Figure R33 (Figure 6h) Charge compensation between the TM ions diagram.

22 The colors of spectra in insets of Mn-K and Ni-K in Fig. 6 are difficult to distinguish. Maybe the dashes should be kept instead of full lines, and arrows indicating the shift of the spectra upon charging / discharging could be added.

Response: Thank you for valuable suggestions. we have revised the figure by reintroducing dashed line styles to improve the visual distinction between the spectra. In addition, arrows have been added to clearly indicate the spectral shifts during the charging and discharging processes. These improvements enhance both the clarity and interpretability of the figure. The updated figure has been incorporated into the revised manuscript.

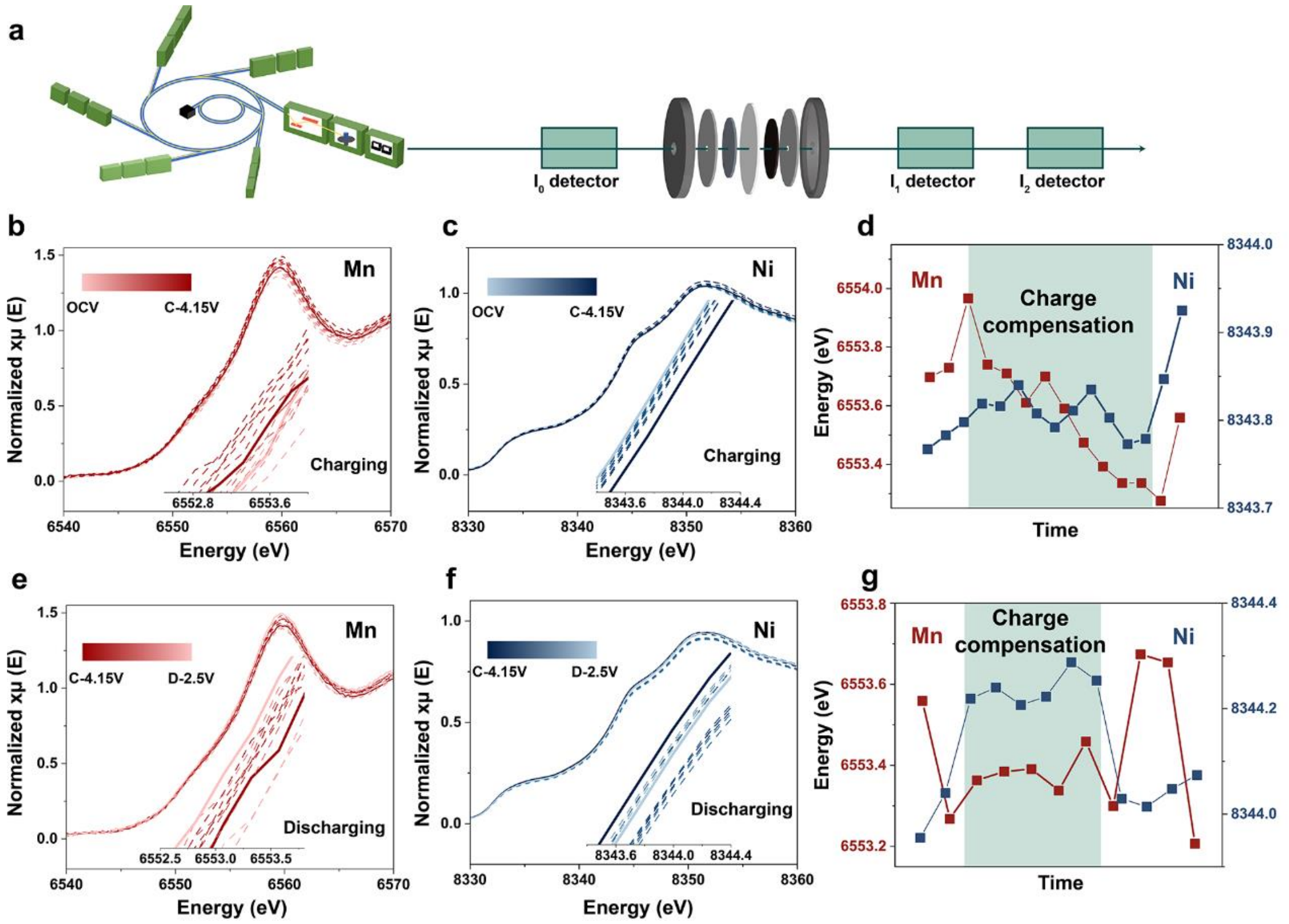

Figure R34 (Figure 6a-6g) a, Schematic of the in-situ battery XAS experiment set-up in ANSTO. In-situ XANES spectrum of **b,** Mn K-edge; **c,** Ni K-edge of NLCMMTO during charging process. **d,** The variation of energy for NLCMMTO during charging process. In-situ XANES spectrum of **e,** Mn K-edge; **f,** Ni K-edge of NLCMMTO during discharging process. **g,** The variation of energy for NLCMMTO during discharging process.

Reference

1. Hu, E.; Yu, X.; Lin, R.; Bi, X.; Lu, J.; Bak, S.; Nam, K.-W.; Xin, H. L.; Jaye, C.; Fischer, D. A.; Amine, K.; Yang, X.-Q., Evolution of redox couples in Li- and Mn-rich cathode materials and mitigation of voltage fade by reducing oxygen release. *Nat. Energy* **2018,** *3* (8), 690-698.

2. House, R. A.; Rees, G. J.; McColl, K.; Marie, J.-J.; Garcia-Fernandez, M.; Nag, A.; Zhou, K.-J.; Cassidy, S.; Morgan, B. J.; Saiful Islam, M.; Bruce, P. G., Delocalized electron holes on oxygen in a battery cathode. *Nat. Energy* **2023,** *8* (4), 351-360.

3. Liang, X.; Song, X.; Sun, H. H.; Kim, H.; Kim, M.-C.; Sun, Y.-K., High-energy and long-life O3-type layered cathode material for sodium-ion batteries. *Nat. Commun.* **2025,** *16* (1), 3505.

4. Wang, Q.; Zhou, D.; Zhao, C.; Wang, J.; Guo, H.; Wang, L.; Yao, Z.; Wong, D.; Schuck, G.; Bai, X.; Lu, J.; Wagemaker, M., Fast-charge high-voltage layered cathodes for sodium-ion batteries. *Nature Sustainability* **2024,** *7* (3), 338-347.

5. Zhang, T.; Ren, M.; Huang, Y.; Li, F.; Hua, W.; Indris, S.; Li, F., Negative lattice expansion in an O3-type transition-metal oxide cathode for highly stable sodium-ion batteries. *Angew. Chem.* **2024,** *136* (8), e202316949.

6. Wang, P.-F.; Yao, H.-R.; Liu, X.-Y.; Yin, Y.-X.; Zhang, J.-N.; Wen, Y.; Yu, X.; Gu, L.; Guo, Y.-G., Na^+/vacancy disordering promises high-rate Na-ion batteries. *Science Advances 4* (3), eaar6018.

7. Liu, S.; Liu, F.; Zhao, S.; Zhuo, Z.; Xiao, D.; Cui, Z.; Wang, Y.; Wang, B.; Wu, T.; Li, Y.; Liang, L.; Huang, H.; Yang, W.; Yu, H., A high-entropy engineering on sustainable anionic redox Mn-based cathode with retardant stress for high-rate sodium-ion batteries. *Angew. Chem. Int. Ed.* **2025,** *64* (10), e202421089.

8. Wang, X.; Zhang, Q.; Zhao, C.; Li, H.; Zhang, B.; Zeng, G.; Tang, Y.; Huang, Z.; Hwang, I.; Zhang, H.; Zhou, S.; Qiu, Y.; Xiao, Y.; Cabana, J.; Sun, C.-J.; Amine, K.; Sun, Y.; Wang, Q.; Xu, G.-L.; Gu, L.; Qiao, Y.; Sun, S.-G., Achieving a high-performance sodium-ion pouch cell by regulating intergrowth structures in a layered oxide cathode with anionic redox. *Nat. Energy* **2024,** *9* (2), 184-196.

9. Li, K.; Shi, L.; An, J.; Zhang, M.; Du, Y.; Ma, Y.; Lou, S.; Yin, G.; Yu, Z.; Hua, X.; Huo, H., Stabilizing configurational entropy in spinel-type high entropy oxides during discharge-charge by overcoming kinetic sluggish diffusion. *Angew. Chem.* **2025,** *137* (51), e202518569.

For Reviewer #1:

My concerns have been well addressed, and I now recommend its publication.

Response: We sincerely thank the reviewer for the positive evaluation and recommendation for publication.

For Reviewer #2:

I appreciate the authors' detailed responses. Overall, most of my concerns have been addressed, particularly the more cautious interpretation of the O–O dimer and Ni–O–Mn superexchange mechanisms, as well as the additional discussion of Cu, Li, Ti, and configurational entropy.

However, the response to the role of the O3 phase and the P2/O3 intergrowth region remains only partially satisfactory. The added 4D-STEM phase and charge maps suggest local electrostatic/structural differences between P2 and O3 domains, but they do not directly demonstrate that the O3 phase provides mechanical support. Likewise, charge redistribution at the P2/O3 interface may indicate interfacial activity, but it is still indirect evidence for stress accommodation or enhanced Na^+ transport.

The statement that the higher O3 fraction in NLCMMTO leads to improved long-term cycling stability is also not fully justified, because Li incorporation simultaneously affects the O3 fraction, oxygen-redox behavior, local electronic structure, and charge redistribution. Therefore, the role of the O3 phase should be described more cautiously. I suggest that the authors either provide quantitative P2/O3 phase fractions with uncertainties and correlate them with lattice strain parameters such as $\Delta c/c$, $\Delta V/V$, or strain anisotropy, or explicitly acknowledge that the precise contribution of the O3 phase remains unresolved. In the absence of such evidence, the P2/O3 interface should be described as a possible contributor to stress accommodation and Na^+ transport, rather than as a demonstrated origin of the improved cycling stability.

Response: Thanks for your advisable suggestion. We agree that the provided 4D-STEM phase and charge maps of response letter reveal local structural and electrostatic differences between P2 and O3 domains, but they do not directly demonstrate that the

O3 phase provides mechanical support. We also agree that Li incorporation simultaneously modifies the P2/O3 phase distribution, oxygen-redox behavior, local electronic structure, and interfacial charge redistribution, and the improved cycling stability might be consequence of their combined effects.

Following the advice, we have revised the manuscript to describe the role of the O3 phase and the P2/O3 intergrowth region more cautiously after careful consideration. The previous statements imply that the higher O3 fraction directly leads to improved long-term cycling stability have been weakened. Besides, to gain a clearer understanding of the contribution of the O3 phase, stress calculations were also conducted between the P2 phase and the P2/O3 heterostructure, thereby evaluating the influence of O3 incorporation. In the revised manuscript, we have added a comparison of the calculated stress distribution between the pure P2 structure and the P2/O3 intergrowth structure of NLCMMTO shown as **Figure R1** (Supplementary Figure S37). Compared with the pure P2 structure, the P2/O3 intergrowth structure shows lower calculated stress throughout the simulated process, suggesting that the P2/O3 intergrowth may contribute to alleviating local stress accumulation.

In addition, considering the absence of direct evidence for fast ion conduction within the interfacial region, we have revised the relevant discussion in a more cautious manner. The updated description avoids assigning a definitive ion transport function to the interface and instead highlights its possible contribution to mechanical support and structural stabilization. We also have revised the corresponding description in the revised manuscript (lines 258-263) as:

In addition, the Tresca stress calculation further shows that the P2/O3 intergrowth structure has lower stress accumulation than the pure P2 structure (Figure S37), suggesting that the intergrowth region may act as a possible contributor to stress accommodation during cycling. The spontaneous contraction of the interlayer spacing, which may be related to oxygen involved charge redistribution, could further reduce stress accumulation and contribute to structural stabilization during cycling.

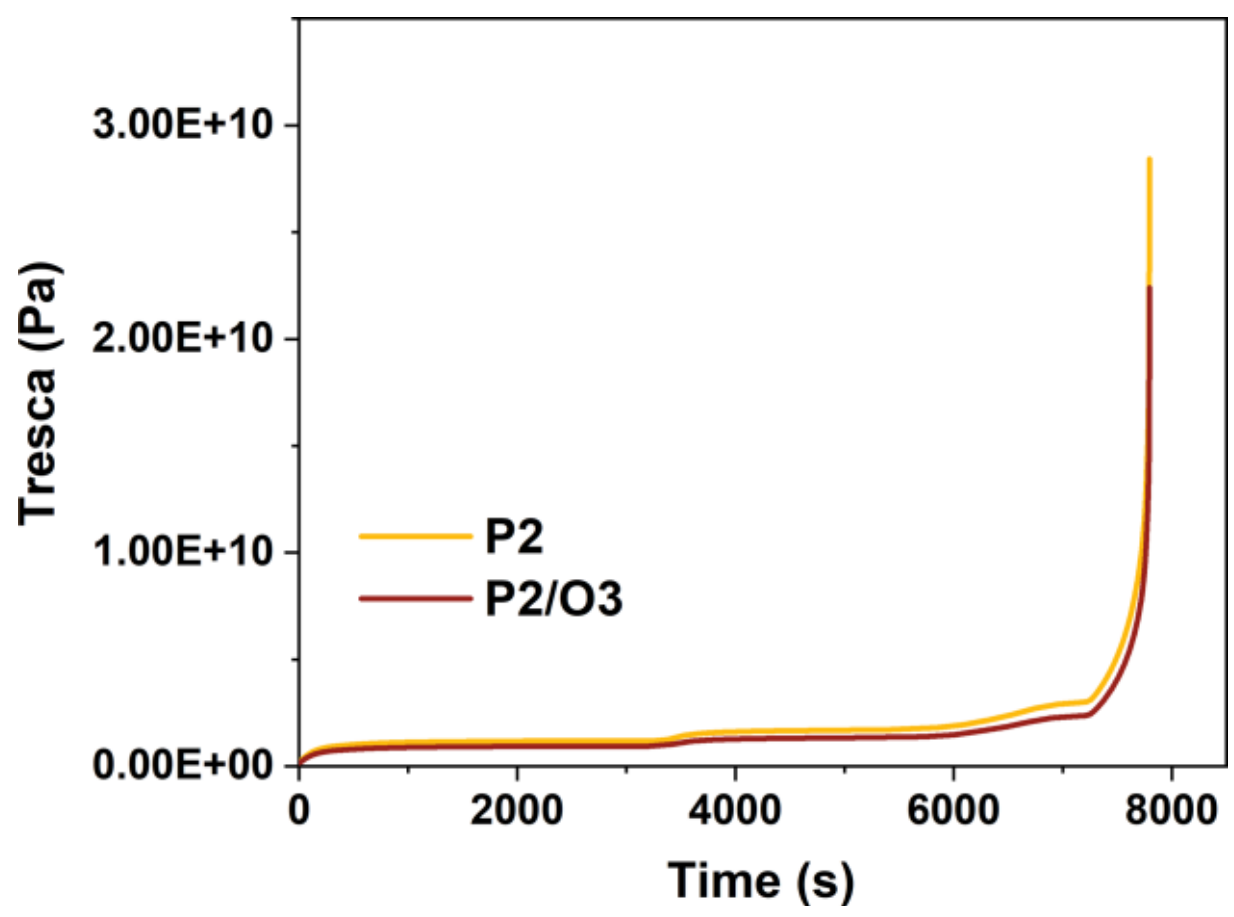

Figure R1 (Figure S37) Averaged stress calculation simulation results for different phase compositions of NLCMMTO, including pure P2 and P2/O3 mixed phases.

For Reviewer #3:

I thank the authors for providing detailed answers to my comments. Several aspects of the study have been clarified in the revised version. Even though most of my comments have been addressed convincingly, some answers require further clarifications, as detailed below.

• Comment 3)

The authors provide an explanation for the Mn-K XANES intensity difference between NMMTO and the two other compounds NLCMMTO and NCMMTO. I suggest the authors consider including the proof of shortened Mn-O bond length (Figure R17 a) in the revised manuscript or at least in SI.

As it stands, the current modification to the revised manuscript is limited to changing "the well overlapped spectrum " to "the nearly identical spectra", which is a very light modification.

Response: Thanks for your valuable suggestion. We have added Mn Fourier-transform EXAFS spectra for NMMTO, NCMMTO and NLCMMTO to the revised Supplementary Information Figure S1 shown as **Figure R2**. We also have revised the corresponding description in the revised manuscript (lines 105-110) as:

X-ray absorption near-edge structure (XANES) has been used to analyze the oxidation state of Ni, Mn and Cu in three samples, the nearly unchanged absorption

edge positions present that there is no obvious valence change by element substitution in three samples (Figure 1b-1d). However, a slight difference in the Mn spectral feature, particularly in peak intensity, is observed for NMMTO. This variation is attributed to the subtle change of local coordination environment induced by the shortened Mn-O bond length (Figure S1).

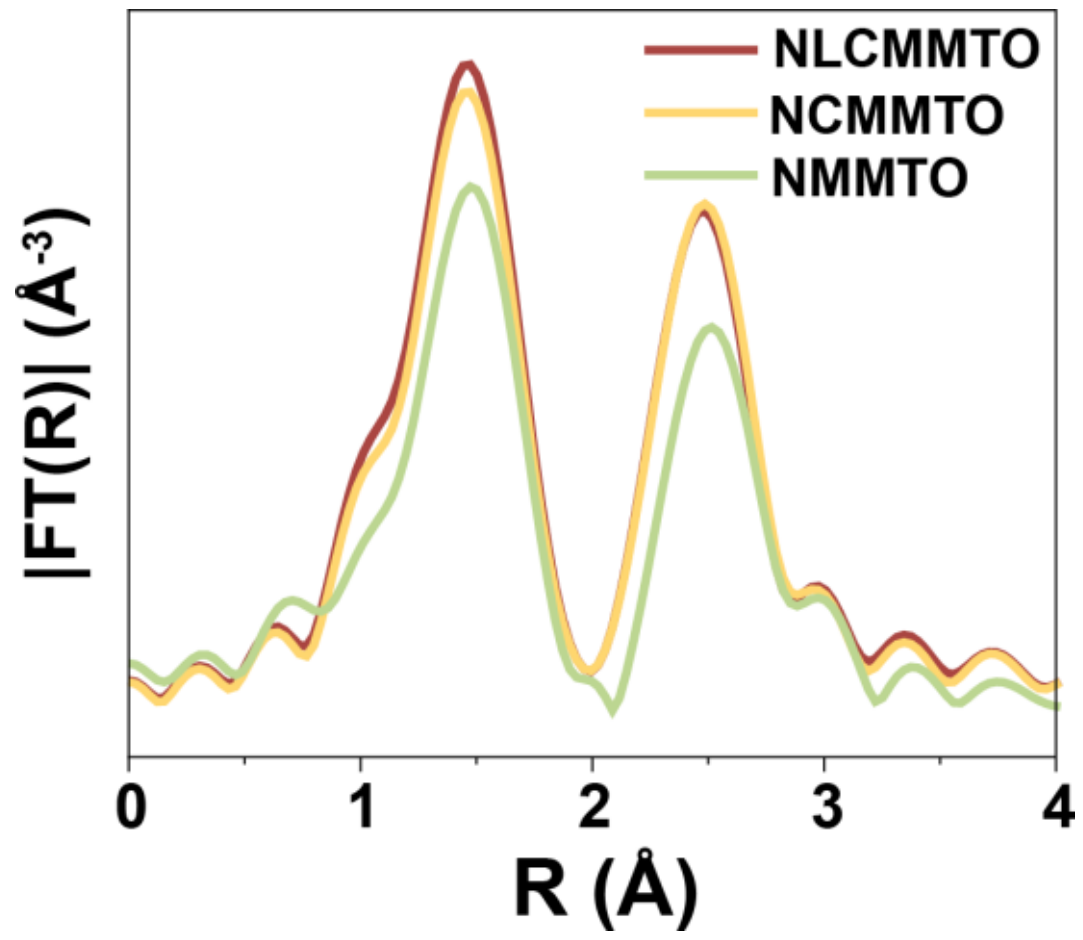

Figure R2 (Figure S1) Fourier-transform EXAFS spectra in R space in NMMTO, NCMMTO and NLCMMTO.

• Comment 4)

Here as well, the data regarding the specimen thickness, shown in the answer, would be of interest to the reader, at least in SI.

Response: Thank you for this helpful suggestion. We agree that the specimen thickness information is valuable for readers in evaluating the reliability of the STEM/HAADF analysis. Therefore, the corresponding thickness data previously provided in the response has now been included in the Supplementary Information as Figure S10. The related description has also been revised in revised manuscript (lines 155-157):

A 4D-STEM dataset is acquired by scanning a focused electron probe across a sample and using a pixelated detector to image the scattered electron beam at each probe position (Figure 2a), the FIB-STEM sample preparation process and thickness of samples were shown in Figure S9 and S10.

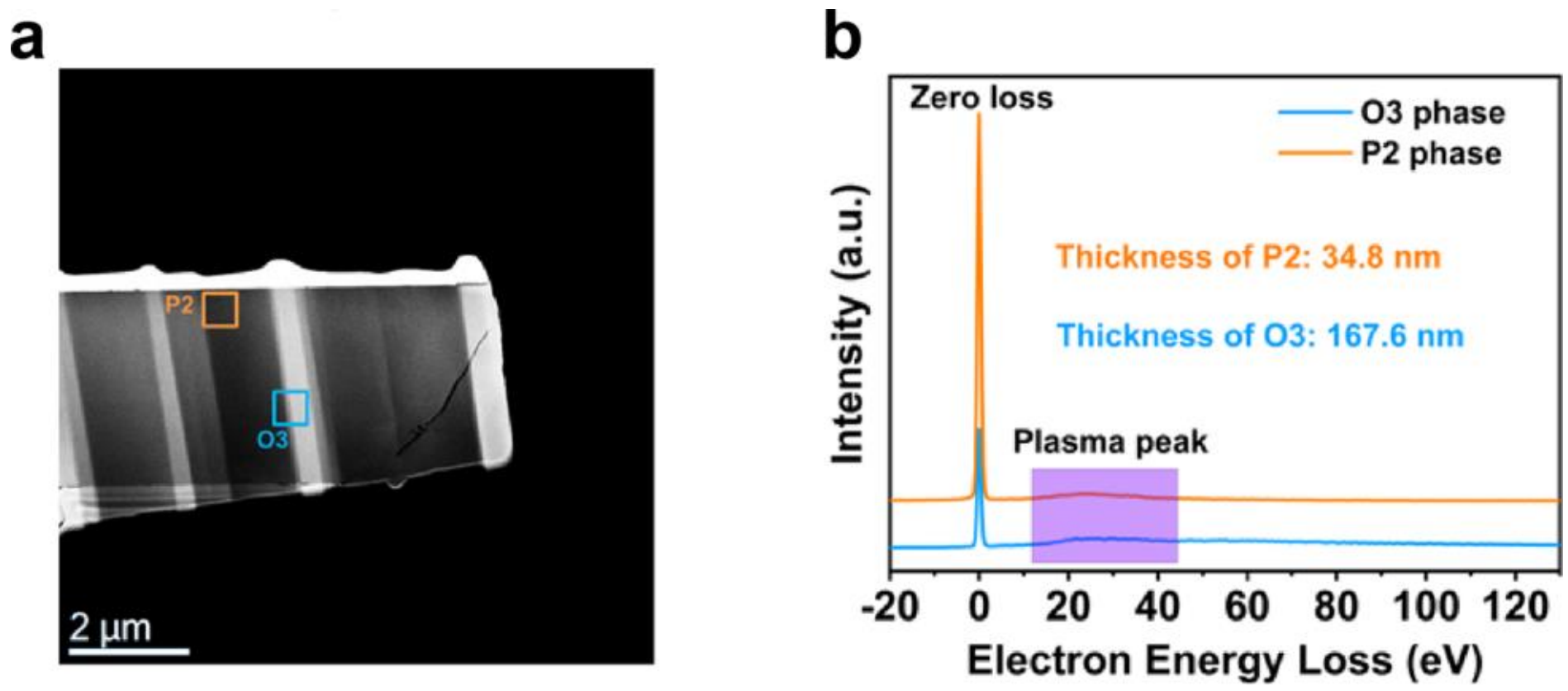

Figure R3 (Figure S10) **a,** Cross-sectional TEM image of NLCMMTO. **b,** Electron energy loss spectroscopy (EELS) spectra collected from the P2 and O3 regions.

• Comment 9)

The spectra for NLCMMTO in the revised Figure 4c are drastically different of those in the original Figure 4a. Why? Have the data been acquired again?

Furthermore, various statements in the answer to this comment raise further questions. First, referring to Figure R27a the peak at 529 eV in the O-K edge of NMMTO seems to disappear during the charging process, which is opposite to the authors' answer. Second, the authors indicate that this remaining pre-peak at 529 eV of the O-K edge and the Mn-L edges shown in Figure R27b suggest that the Mn valence state shows negligible variation during cycling. This also appears very unlikely from the spectra given in Figure R27. A clear change of spectral weight is apparent in the Mn-L3 edge upon cycling, with a drastic red-shift at C-2.5 giving a Mn-L23 edge characteristic of low valence (~2+). Local geometrical effects, such as distortion, bond lengths, etc., can modify near-edge fine structures at L23 edges of transition metals. However, associating the spectral shifts in Figure R27b to a negligible variation of the Mn valence is surprising and requires solid proof (through e.g. multiplet calculations).

Response: We thank the reviewer for the careful observation. The spectra shown in the revised Figure 4c was reacquired under optimized measurement conditions to improve the signal-to-noise ratio. We have collected the O K-edge of different samples using different hemispherical analyzer (HA) retardation voltages. The HA setting mainly modifies the energy filtered electron yield detection condition rather than the intrinsic

absorption process. Changing the HA setting changes the detected electron energy window and the relative contributions from Auger, energy loss Auger, secondary electron, and background signals. (Sci. Rep. 2016, 6, 24659; Appl. Surf. Sci., 2022, 575, 151610.) As shown in **Figure R4a** and **R4b**, increasing the HA value from 100 to 150 selectively enhances the feature centered at approximately 533.6 eV in both the C-3.5 V and C-4.15 V samples. The feature around 533.6 eV at charging 3.5V remains observable in both revised Figure 4c and original Figure 4a, suggesting the persistent involvement of oxygen related electronic states in charge compensation. Therefore, HA 150 was selected as the optimized condition for resolving the high energy O-related SXR feature. This accounts for the apparent difference in the detailed line shape compared with the original spectra. We apologize that this reacquisition and optimization of the measurement conditions were not clearly stated in our previous response.

We agree with the reviewer that the O K-edge spectra alone should not be used to determine the Mn valence state. In our previous response, we attempted to interpret the Mn valence evolution based partly on the O K-edge spectra presented in the original manuscript. However, we acknowledge that this approach has intrinsic limitations, especially considering that the O K-edge reflects the hybridization between O 2p states and multiple enabled redox transition metal species rather than Mn alone. Therefore, in the revised discussion, the O K-edge is only described as reflecting changes in TM-O hybridization and local electronic structure during cycling. Moreover, we agree that Mn L-edge features can be affected by both Mn valence and local geometrical/electronic effects, including ligand-field changes, Mn-O bond distortion, and covalency, a quantitative assignment of Mn valence would require more rigorous analysis, such as multiplet calculations. As such calculations were not performed in this work, we have avoided making a quantitative claim about Mn valence evolution and have limited the discussion to qualitative changes in the Mn local electronic structure during cycling. And we have carefully checked the revised manuscript and confirmed that the O K-edge is not used there to determine the specific Mn valence state.

We sincerely thank the reviewer for the careful examination of our response and for correcting these inaccuracies. These comments have helped us improve the clarity and accuracy of the revised manuscript and response.

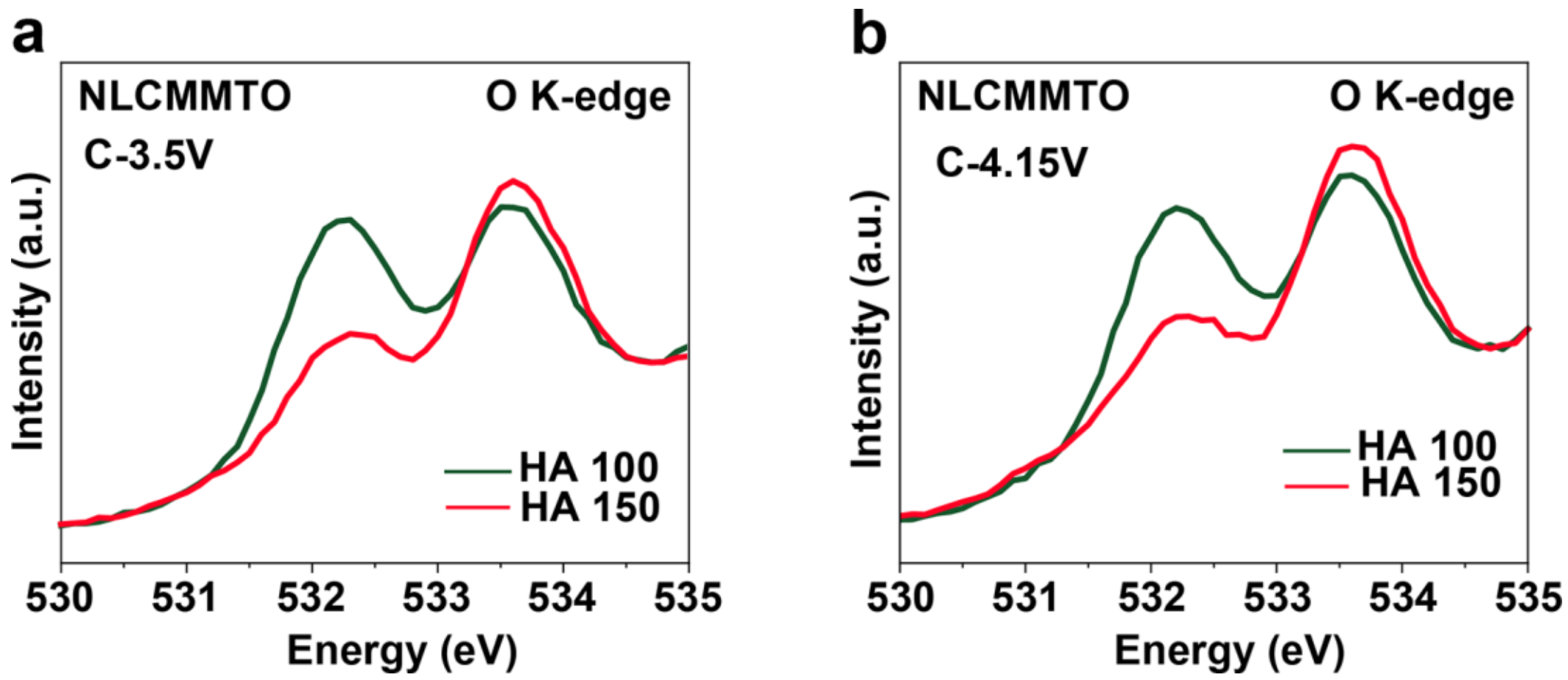

Figure R4 O K-edge XAS spectra of NLCMMTO with different hemispherical analyzer parameters at **a,** 3.5 V; **b,** 4.15V.

• Comment 10)

The authors indicate that the HAADF analysis is not used as a standalone quantitative method for determining subtle lattice changes. They agree that intrinsic uncertainty is is associated with directly measuring interplanar distances from these images. My initial question was about this uncertainty: how precise are the values 0.54, 0.55 and 0.56 nm? Thus far, the text has not been changed, and the statement (p. 324-326) does not reflect the uncertainty associated with such measurements: "[…] the interlayer distance still maintains 0.55 nm as pristine state, otherwise the distance could be enlarged to 0.56 nm after extraction Na+. When charging to 4.15V, the distance reduces to 0.54 nm without TM slabs sliding or disordering." Such statement does not indicate nor suggest any uncertainty to these values, which is misleading.

Response: Thanks for your constructive comment. We agree that direct measurement of interplanar distances from HAADF-STEM images inherently involves experimental uncertainty and therefore should not be interpreted as highly precise quantitative values. The interlayer spacings were estimated by averaging measurements taken from multiple regions in the HAADF-STEM images, as shown in **Figure R5** (Revised Figure 5a and 5b). Our intention was mainly to describe the overall trend of interlayer spacing

evolution during cycling rather than to claim exact lattice parameters from the HAADF images alone. According to your suggestions, we have revised the corresponding description in the manuscript to avoid overinterpretation of the measured distances. Rather than emphasizing the exact values of 0.54, 0.55, and 0.56 nm, the revised text now describes the interlayer spacing as exhibiting only slight variation during cycling. In addition, the annotations of the interlayer spacings in the corresponding figure have also been revised accordingly. We have revised the corresponding description in the revised manuscript as:

From Figures 5a and 5b, the interlayer distance increase slightly after extraction Na^{+} at charging 3.5V. And then when charging to 4.15V, the distance reduces. (Lines 326-328)

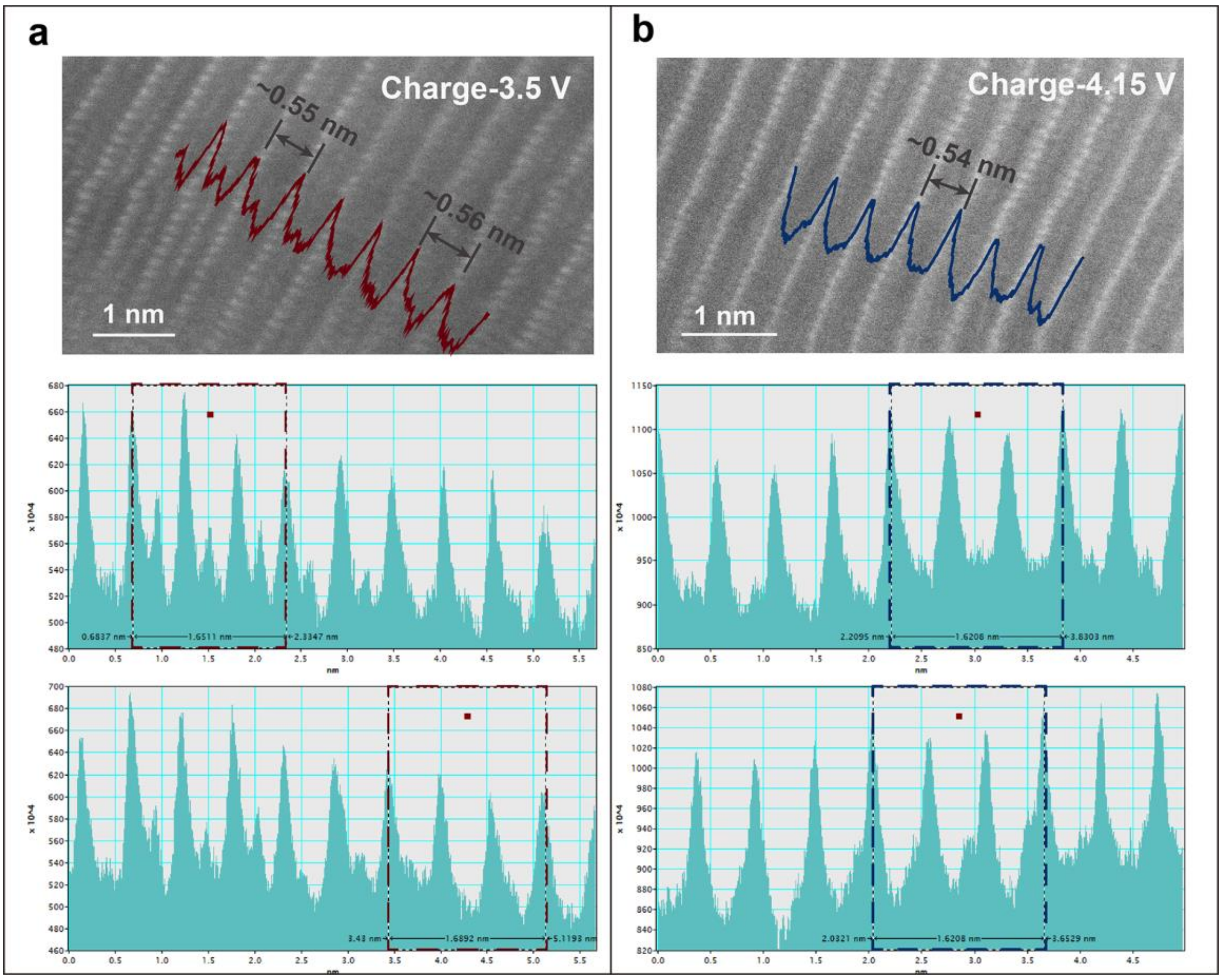

Figure R5 (Figure 5a and 5b) O K-edge XAS spectra of NLCMMTO with different hemispherical analyzer parameters at **a,** 3.5 V; **b,** 4.15V.

• Comment 12)

I disagree with the answer provided by the authors here. Subtle effects like mixed-valence variations at the Mn-L23 XANES spectra of a complex structure like NLCMMTO cannot be interpreted solely by fingerprinting standard references like MnO, Mn2O3 and MnO2. Matching the energy of peaks in NLCMMTO with these standards does not mean that Mn2+, 3+ and 4+ are present in NLCMMTO. For example, pure Mn2+ compounds like MnO also have a Mn L-edge with intensity where the 3+ and 4+ "reference" peaks are marked, but it does not make MnO a mixed valence system.
The main spectral weight of the spectrum can be linked to the valence of Mn. But this is not the case for all the peaks and shoulders, which are associated with both the valence and local geometry effects. To discriminate between valence and geometry effects, multi-electronic calculations (e.g. multiplet, etc.) will provide a solid and quantitative answer, but not comparison with standards (with different local geometries and chemistries). Therefore, from the evidence provided and without appropriate calculations, only the overall valence and its trend could be mentioned. The Mn valence seems to go from an overall 4+ (OCV and -4.15) to 2+ (C-3.5 and C-2.5). Other valences cannot be excluded but cannot be supported either.
As it stands, the following statement is not supported: "[…] to the mixed valence of +2/+3/+4 upon charging to 3.5 V, which [...]".

Response: We thank the reviewer for this important and insightful comment. We agree that comparison with MnO, Mn_2O_3 and MnO_2 reference spectra alone cannot rigorously demonstrate the coexistence of $Mn^{2+}/Mn^{3+}/Mn^{4+}$ in NLCMMTO, since accurate decomposition of mixed Mn oxidation states from Mn L-edge XAS/EELS can be affected by instrumental resolution and energy-axis calibration, making simple reference fingerprinting insufficient for rigorous quantification (Sci Rep. 2023 13, 14132). Although Mn L-edge XAS has been commonly used to track Mn redox evolution through changes in the main spectral weight and edge position (ACS Nano 2026, 20, 1357-1369; Adv. Mater. 2021, 33, 2004280). We acknowledge that individual peaks and shoulders are also strongly influenced by local coordination geometry,

ligand-field effects, and Mn-O hybridization. Detailed analysis of transition metal L-edge spectra generally requires multiplet-based simulations because the line shape is governed by electron-electron interactions, spin-orbit coupling and local structural distortions (Inorg. Chem. 2023, 62, 3738-3760).

Thus, we have removed the unsupported statement assigning the spectra to "mixed valence of +2/+3/+4." In the revised manuscript, the MnO, Mn_2O_3 and MnO_2 reference spectra are used only as qualitative guides to track the overall Mn valence evolution trend. We also have revised the corresponding description in the revised manuscript (Lines 352-354) as:

NLCMMTO exhibits an Mn reduction trend during charging. Compared with the relatively high Mn oxidation state at OCV, the spectral weight redistribution at C-3.5V suggests an overall evolution of Mn toward lower oxidation states, followed by oxidation to higher oxidation state at 4.15V.

• Comment 15)

I thank the authors for their answer. Maybe the point could be made for the reader, with a clear mention that "the DOS near the Fermi level reflects the availability of electronically accessible states that can participate in charge compensation."

Response: We thank the reviewer for this helpful suggestion. We agree that explicitly clarifying the physical meaning of the DOS near the Fermi level would improve the readability of the discussion. Based on suggestion, we have revised the manuscript (Lines 370-371):

The DOS near the Fermi level reflects the availability of electronically accessible states that can participate in charge compensation.

We greatly appreciate the reviewer's time and thoughtful feedback, which helped us improve both the presentation and the scientific content of our work.

For Reviewer #2:

The authors have adequately addressed my remaining concern. Although the additional stress calculation does not fully isolate the independent contribution of the O3 phase, the revised manuscript now presents the role of the P2/O3 intergrowth region more cautiously and appropriately describes it as a possible contributor to stress accommodation rather than as a demonstrated origin of improved cycling stability. Therefore, I am satisfied with the revision and recommend acceptance, subject to minor editorial checks such as figure numbering and language polishing. For example, the stress calculation is referred to as Supplementary Figure S37 in the response letter, whereas it appears as Figure S36 in the revised manuscript and Supporting Information. In addition, several Figure 2 panel references appear inconsistent with the figure caption, especially the assignments of the electric-field and charge-density maps. The caption of Figure R5 in the response letter should also be corrected, as it appears to describe O K-edge XAS rather than the HAADF-STEM/line-profile analysis shown.

Response: Thanks for your careful reading. We have carefully checked all figure numbers and captions throughout the main text to ensure consistency and accuracy.

We have carefully checked and corrected the panel references associated with Figure 2, particularly those corresponding to charge density maps, to ensure consistency among the figure, figure caption, and main text. We also have revised the corresponding description in the revised manuscript (lines 155-159) as:

…the experimental projected electric field maps in Figure 2h and 2l…

…we calculated the projected charge density (Figure 2i and 2m)…

The caption of Figure R1 (original Figure R5) has been revised to "High-angle annular dark-field scanning transmission electron microscopy images and corresponding intensity line profiles of NLCMMTO charged to **a,** 3.5V, **b,** 4.15 V, respectively."

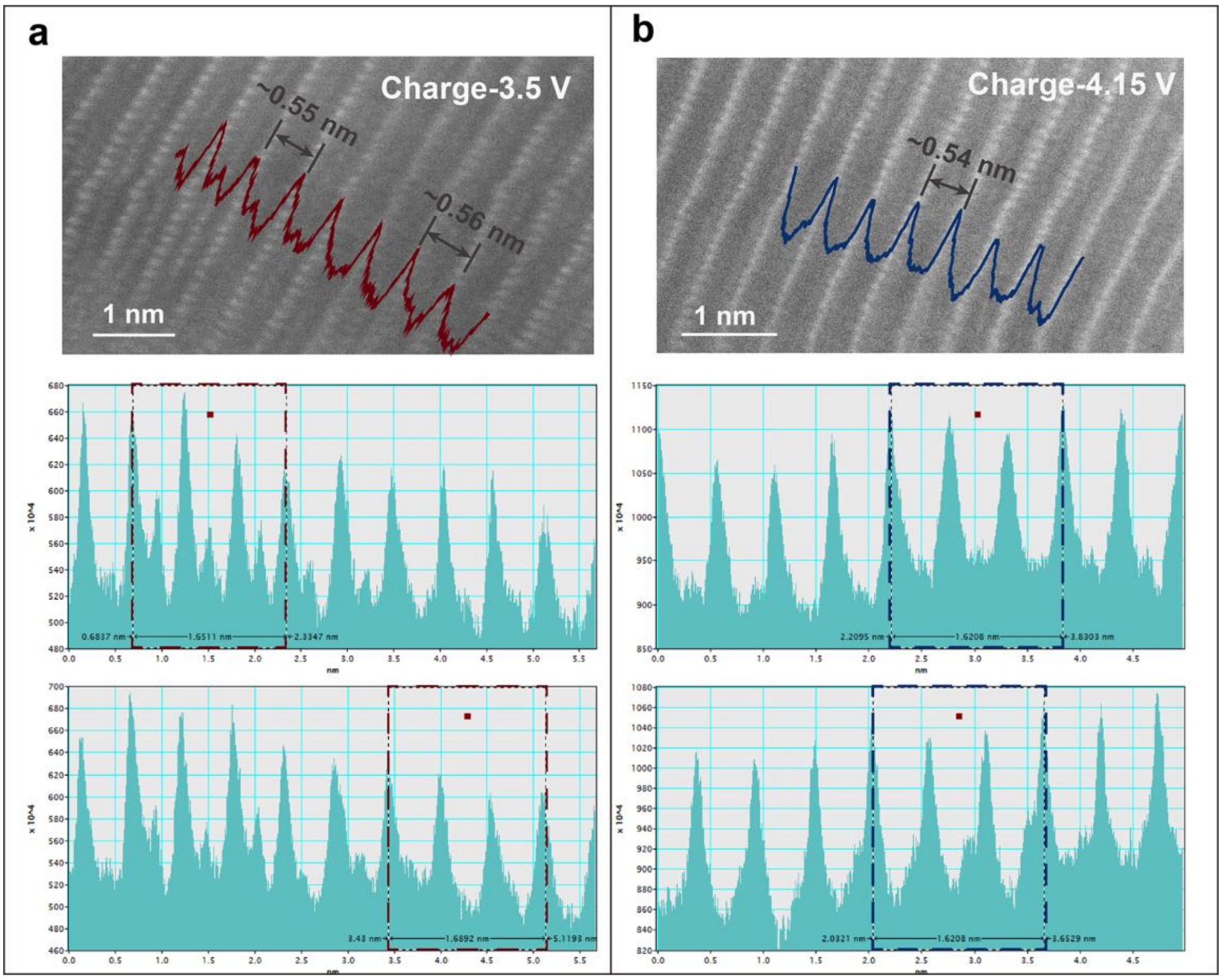

Figure R1 (original Figure R5) High-angle annular dark-field scanning transmission electron microscopy images and corresponding intensity line profiles of NLCMMTO charged to **a,** 3.5V, **b,** 4.15 V, respectively.

For Reviewer #3:

The authors have addressed all of my concerns. I now recommend this work for publication in Nature Communications.

Response: We sincerely thank the reviewer for the positive evaluation and recommendation for publication.

All revisions requested by the reviewer have been fully implemented. We greatly appreciate the reviewer's time and thoughtful feedback, which helped us improve both the presentation and the scientific content of our work.

.
